# Incidence, Morbidity and years Lived With Disability due to Type 2 Diabetes Mellitus in 204 Countries and Territories: Trends From 1990 to 2019

**DOI:** 10.3389/fendo.2022.905538

**Published:** 2022-07-11

**Authors:** Rongrong Zhu, Shan Zhou, Liang Xia, Xiaoming Bao

**Affiliations:** ^1^ Department of Pharmacy, Hwa Mei Hospital, University of Chinese Academy of Sciences, Ningbo, China; ^2^ Department of Endocrinology, Hwa Mei Hospital, University of Chinese Academy of Sciences, Ningbo, China; ^3^ Department of Gynecology, Hwa Mei Hospital, University of Chinese Academy of Sciences, Ningbo, China; ^4^ Department of Cardiology, Hwa Mei Hospital, University of Chinese Academy of Sciences, Ningbo, China

**Keywords:** type 2 diabetes mellitus, incidence, mortality, disability-adjusted life-years, global burden of disease (GBD)

## Abstract

**Background:**

We aimed to examine the descriptive epidemiology and trends in the burden of type 2 diabetes mellitus (T2DM).

**Methods:**

Data were extracted from the Global Burden of Disease 2019 dataset. Estimated annual percentage changes (EAPCs) were calculated to assess the trends in incidence rate, mortality and disability-adjusted life-years (DALYs) associated with T2DM. Measures were stratified by sex, region, country, age and social development index (SDI) value.

**Results:**

The global age-standardized incidence rate of T2DM increased from 1990 to 2019, with an EAPC of 1.25 (95% CI, 1.19 to 1.31). In 2019, the highest age-standardized incidence rate of T2DM was observed in high-SDI regions, and the largest increase in this rate from 1990 to 2019 was also in high-SDI regions (EAPC, 1.74;95% CI, 1.57 to 1.90). At the regional level, Central Asia (EAPC, 2.53;95% CI, 2.45 to 2.61) had the largest increase in the age-standardized incidence rate of T2DM from 1990 to 2019. At the national level, Luxembourg (EAPC, 4.51;95% CI, 4.37 to 4.65) and Uzbekistan (EAPC, 3.63; 95% CI, 3.44 to 3.82) had the largest increases in the age-standardized incidence rate of T2DM from 1990 to 2019. The global age-standardized death and DALY rates increased from 1990 to 2019, with EAPCs of 0.26 (95% CI, 0.16 to 0.37) and 0.81 (95% CI, 0.77 to 0.85), respectively. The age-standardized death and DALY rates showed the largest increases in Central Asia, South Asia and Southern Sub-Saharan Africa.

**Conclusions:**

Globally, the age-standardized incidence, death and DALY rates increased from 1990 to 2019. Central Asia, South Asia and Southern Sub-Saharan Africa were found to have the greatest burden of T2DM. Future strategies should focus on these high-risk regions and other high-risk populations.

## Introduction

Type 2 diabetes mellitus (T2DM) is a great public health chanllenge globally, imposing a heavy burden on public health and socioeconomic development (1, 2). In 2021, it is estimated that 537 million people have diabetes, and this number is projected to reach 643 million by 2030, and 783 million by 2045 (3). T2DM is the most regular type of diabetes (3).

Currently, no study has calculated the global estimated annual percentage change (EAPC) in T2DM burden from the Global Burden of Diseases, Injuries, and Risk Factors Study (GBD) 2019 datasets. The most recent study of T2DM used data from the GBD 2017 to obtain the total percentage change in disease burden from 1990 to 2017 (4).

The GBD 2019 assessed the burden of 369 human diseases and injuries in 204 countries and territories worldwide (5). GBD data are updated annually, with changes in methods made appropriately (5). Therefore, we assessed the T2DM burden based on GBD 2019 data by determining the temporal trends in T2DM incidence rates, death rates and T2DM-related DALYs at the global, regional and national levels according to sex, age and socio-development index (SDI) value.

## Methods

### Overview

We extracted data from the latest edition of the 2019 GBD research report, according to the GBD’s operational guidelines. T2DM disease burden was analyzed using the Global Health Data Exchange. We obtained T2DM-related morbidity, mortality and DALY data. The official GBD research website details the general approach adopted by the GBD team in 2019 (5). We also analyzed global trends in T2DM according to the following 2019 GBD age stratifications: 10–14, 15–19, 20–24, 25–29, 30–34, 35–39, 40–44, 45–49, 50–54, 55–59, 60–64, 65–69, 70–74, 75–79, 80–84, 85–89, 90–94 and greater than 95 years.

The SDI value is based on educational attainment, per-capita income level and fertility rate. SDI values range from 0 to 1, with larger values indicating higher levels of social and economic development (6). In the GBD 2019, 204 countries or regions were divided into the following five groups based on SDI values: high-SDI group (>0.81) medium-high-SDI group (0.70–0.81), medium-SDI group (0.61–0.70), medium-low-SDI group (0.46–0.61) and low-SDI group (< 0.46) (6).

This study was conducted in accordance with the recommendations of the Guidelines for Accurate and Transparent Health Assessment Reporting (7). The Ethics Committee of Hwa Mei Hospital reviewed and approved this study.

### Data Sources and Estimation Framework

A systematic review of diabetes prevalence, morbidity, and mortality using relevant search terms for GBD 2019 identified 717 records (5). An additional 281 records were identified, and 998 records were identified through the reference list of the aforementioned articles.In addition, the Global Health Data Exchange website was searched for multinational surveys, national surveys, and longitudinal studies on measuring diabetes or fasting blood glucose.

T2DM incidence was estimated using DisMod-MR 2.1, a Bayesian meta-regression disease modeling tool (8). The non-specific codes of all available data on mortality were corrected and used to estimate mortality rates for T2DM (8). The Cause of Death Ensemble model (CODEm) was used to estimate death rates (data from verbal autopsy and vital registration data) (8). DALYs due to T2DM were calculated as the sum of years lived with disability and the years of life lost (9). All estimates were generated with 95% uncertainty intervals (UIs).

### Case Definition of T2DM

T2DM was defined as fasting plasma glucose (FPG) ≥ 126 mg/dL (7 mmol/L) or reporting to be on drug or insulin treatment for type 2 diabetes (5). Determination of T2DM in the GBD study is based on the International Classification of Diseases ICD-10 codes E11.2, E11.21, E11.22, E11.29 (5).

### Statistical Analyses

The EAPCs were determined using the formula Y = α + βX + ε, where Y is ln (age-standardized rate), X is the calendar year, ε is the error term and β is the positive or negative age-standardized rate trend. Assuming that Y is linear with time, EAPC = 100 × [exp (*β*) - 1]. The 95% confidence intervals (CIs) of the EAPCs were calculated from the linear model. When the EAPC and the lower limit of its 95% CI were both positive, the corresponding age-standardized rate was considered to show an upward trend. In contrast, when the EAPC and the upper limit of its 95% CI were both negative, the corresponding age-standardized rate was considered to show a downward trend. Otherwise, the age-standardized rate was regarded as stable (10).

The correlations between EAPCs and age-standardized rates, as well as SDIs and EAPCs were calculated using Gaussian process regression and Pearson’s correlation coefficient (ρ). All calculations were performed using R software (version 3.5.1).

## Results

### Change in the Incidence of T2DM

**Figure 1:**
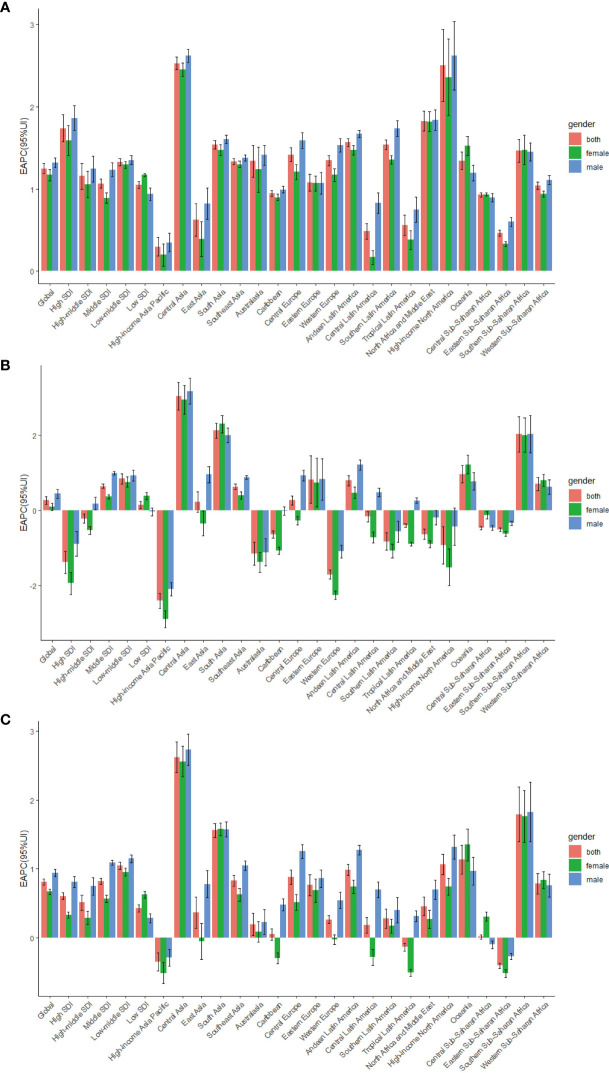
The EAPC of T2DM age-standardized rates from 1990 to 2019, by sex and region. **(A)** The EAPC of ASIR. **(B)** The EAPC of ASDR. **(C)** The EAPC of age-standardized DALY rate; EAPC, estimated annual percentage change.ASIR, age standardized incidence rate; ASDR, age standardized death rate; DALY, disability adjusted life-year.

In 2019, there were 21,669,944.39 cases of T2DM globally (95% uncertainty interval, 20,020,894.99 to 23,513,486.10). There was also an increase in the global number of cases of T2DM from 1990 to 2019, corresponding to a total increase of 157.63%. The age-standardized incidence rate of T2DM increased from 184.55 per 100,000 people (95% UI, 170.91 to 199.70) in 1990 to 259.94 per 100,000 people (95% UI, 240.35 to 281.44) in 2019, corresponding to an EAPC of 1.25 (95% CI, 1.19 to 1.31; **Table 1**; **Figure 1A**; **Supplementary Figure 1A**). However, the age-standardized incidence rate of T2DM from 1990 to 2019 was higher in males than in females(**Table 1**). Globally, the male-to-female ratio of T2DM incidence peaked in the 35–39-year age group (**Supplementary Figure 2A**).

**Table 1:** The age-standardized incidence rate of T2DM in 1990 and 2019 and its temporal trends.

Characteristics	1990		2019		1990-2019	
	ASIR (per 100000)		ASIR (per 100000)		Percent Change(%)	EAPC
	No. (95% UI)	Male/female ratio	No. (95% UI)	Male/female ratio		No. (95% CI)
**Global**	184.55(170.91,199.70)	1.059854422	259.94(240.35,281.44)	1.112174643	1.58(1.53,1.62)	1.25(1.19,1.31)
**Sex**	–	–	–	–	–	–
Male	190.00(175.94,205.59)	–	273.74(253.52,296.19)	–	1.62(1.57,1.68)	1.32(1.26,1.38)
Female	179.27(166.03,194.19)	–	246.13(227.46,266.78)	–	1.53(1.48,1.57)	1.17(1.10,1.24)
**Sociodemographic index**	–	–	–	–	–	–
Low SDI	170.91(156.91,186.54)	1.156314099	227.31(208.31,249.57)	1.024336283	2.13(2.06,2.19)	1.05(1.01,1.09)
Low-middle SDI	182.66(168.05,199.72)	1.069244632	275.61(253.24,299.98)	1.086123148	2.12(2.06,2.19)	1.32(1.28,1.37)
Middle SDI	193.22(178.15,210.54)	0.980565009	254.75(236.70,275.80)	0.990237613	1.67(1.59,1.76)	1.07(1.01,1.12)
High-middle SDI	175.53(162.55,189.43)	1.036521417	231.46(213.85,250.88)	1.09989705	1.13(1.08,1.19)	1.16(1.00,1.31)
High SDI	193.16(178.84,207.74)	1.176700618	287.90(265.51,310.46)	1.272865947	1.17(1.11,1.23)	1.74(1.57,1.90)
**Region**	–	–	–	–	–	–
Andean Latin America	151.84(141.00,161.99)	0.955048127	236.33(218.04,255.89)	1.01061053	2.82(2.69,2.98)	1.57(1.52,1.62)
Australasia	125.62(115.95,135.33)	1.263377824	190.54(172.40,209.26)	1.255897739	1.61(1.41,1.84)	1.34(1.15,1.54)
Caribbean	277.52(260.50,294.18)	1.021845554	366.65(340.50,393.77)	1.04892498	1.32(1.23,1.41)	0.95(0.91,0.98)
Central Asia	131.25(121.75,141.13)	0.904414761	255.39(237.94,274.41)	0.969375556	2.30(2.17,2.46)	2.53(2.45,2.61)
Central Europe	191.93(177.96,207.20)	1.113838675	286.04(263.18,309.88)	1.258910009	0.68(0.63,0.72)	1.42(1.33,1.50)
Central Latin America	358.99(334.03,385.16)	0.965395008	418.88(388.57,448.70)	1.102113451	1.80(1.71,1.89)	0.49(0.39,0.58)
Central sub-Saharan Africa	199.55(184.00,218.56)	1.487396241	257.96(235.99,282.15)	1.474182065	2.40(2.27,2.52)	0.93(0.91,0.95)
East Asia	174.11(158.92,191.40)	0.970609176	202.08(186.94,220.01)	1.119699037	0.98(0.86,1.11)	0.63(0.43,0.82)
Eastern Europe	105.37(96.68,114.91)	1.011570056	142.59(130.96,155.98)	1.055685813	0.45(0.41,0.50)	1.08(0.97,1.18)
Eastern sub-Saharan Africa	153.53(142.18,166.41)	1.219965189	176.12(162.28,191.41)	1.307459187	1.75(1.69,1.82)	0.46(0.42,0.50)
High-income Asia Pacific	164.50(150.93,178.00)	1.44973887	194.88(178.63,212.90)	1.436319019	0.56(0.49,0.63)	0.30(0.18,0.41)
High-income North America	227.94(209.17,247.31)	1.174267455	342.28(317.75,368.26)	1.306909127	1.40(1.30,1.52)	2.50(2.06,2.94)
North Africa and middle East	196.60(180.65,213.67)	0.958764224	353.19(326.05,383.38)	1.00809146	3.55(3.41,3.68)	1.83(1.70,1.95)
Oceania	345.01(321.57,369.47)	1.337580341	506.06(472.95,542.44)	1.218260749	2.58(2.47,2.71)	1.34(1.24,1.45)
South Asia	189.86(173.27,209.29)	1.11828423	299.75(273.78,329.19)	1.123035261	2.43(2.34,2.51)	1.54(1.49,1.59)
Southeast Asia	183.74(169.49,199.23)	0.994150434	273.35(254.04,295.57)	0.998916596	2.23(2.12,2.34)	1.33(1.30,1.37)
Southern Latin America	170.11(156.87,182.41)	0.973435731	274.59(249.87,298.35)	1.108251858	1.61(1.47,1.77)	1.54(1.48,1.60)
Southern sub-Saharan Africa	219.12(203.14,236.97)	0.930863701	326.08(304.70,349.16)	0.920718823	2.03(1.93,2.14)	1.47(1.33,1.60)
Tropical Latin America	242.19(224.18,261.30)	1.032590633	268.57(247.31,292.76)	1.099980084	1.46(1.38,1.54)	0.56(0.44,0.68)
Western Europe	187.90(173.16,202.46)	1.062362694	276.41(252.22,301.22)	1.180594095	0.84(0.76,0.90)	1.35(1.28,1.41)
Western sub-Saharan Africa	144.74(133.27,157.60)	1.010006437	197.56(181.67,214.62)	1.041990279	2.27(2.19,2.35)	1.04(1.00,1.09)

ASIR, age-standardized incidence rate; EAPC, estimated annual percentage change; NA, not available; UI, uncertainty interval.

As shown in **Table 1**, in 2019, the highest age-standardized incidence rates of T2DM (287.90 per 100,000 people; 95% UI, 265.51 to 310.46) were observed in high-SDI regions. The age-standardized incidence rate of T2DM increased the least in low-SDI regions, in which the EAPC was lowest (1.05; 95% CI, 1.01 to 1.09). In contrast, the age-standardized incidence rate of T2DM showed the largest increase in high-SDI regions, in which the EAPC was highest (1.74; 95% CI, 1.57 to 1.90)(**Table 1**; **Figure 1A**).

A negative correlation was found between the EAPC in the age-standardized incidence rate and the age-standardized incidence rate (ρ = −0.188, *P* = 0.007), whereas a positive correlation was found between the EAPC in the age-standardized incidence rate and the SDI value (ρ = 0.072, *P* = 0.305, **Supplementary Figures 3A, B**). Regions with a higher SDI value were also found to have a higher proportion of incident cases of T2DM in young people, and regions with higher SDI values in 1990 and 2019 had a higher proportion of incident cases of T2DM in people aged 50–69 years (**Supplementary Figures 4A, B**). However, the annual proportions of T2DM incidence in elderly people decreased from year to year (**Supplementary Figure 5A**). The incidence of T2DM was markedly higher in people aged 60–64 years than in people of other age groups in all SDI regions (**Supplementary Figure 8A**).

At the regional level, in 2019, the highest age-standardized incidence rates of T2DM were observed in Oceania (506.06 per 100,000 people; 95% UI, 472.95 to 542.44), Central Latin America (418.88 per 100,000 people; 95% UI, 388.57 to 448.70) and the Caribbean (366.65 per 100,000 people; 95% UI, 340.50 to 393.77), whereas the lowest age-standardized incidence rate of T2DM in 2019 was in Eastern Europe (142.59 per 100,000 people; 95% UI, 130.96 to 155.98; **Table 1**; **Supplementary Table 2**). From 1990 to 2019, the age-standardized incidence rate of T2DM showed the largest increase in Central Asia (EAPC, 2.53; 95% CI, 2.45 to 2.61) and the smallest increase in the high-income Asia Pacific region (EAPC, 0.30; 95% CI, 0.18 to 0.41) (**Table 1**, **Figure 1A**, **Supplementary Table 2**).

**Figure 2:**
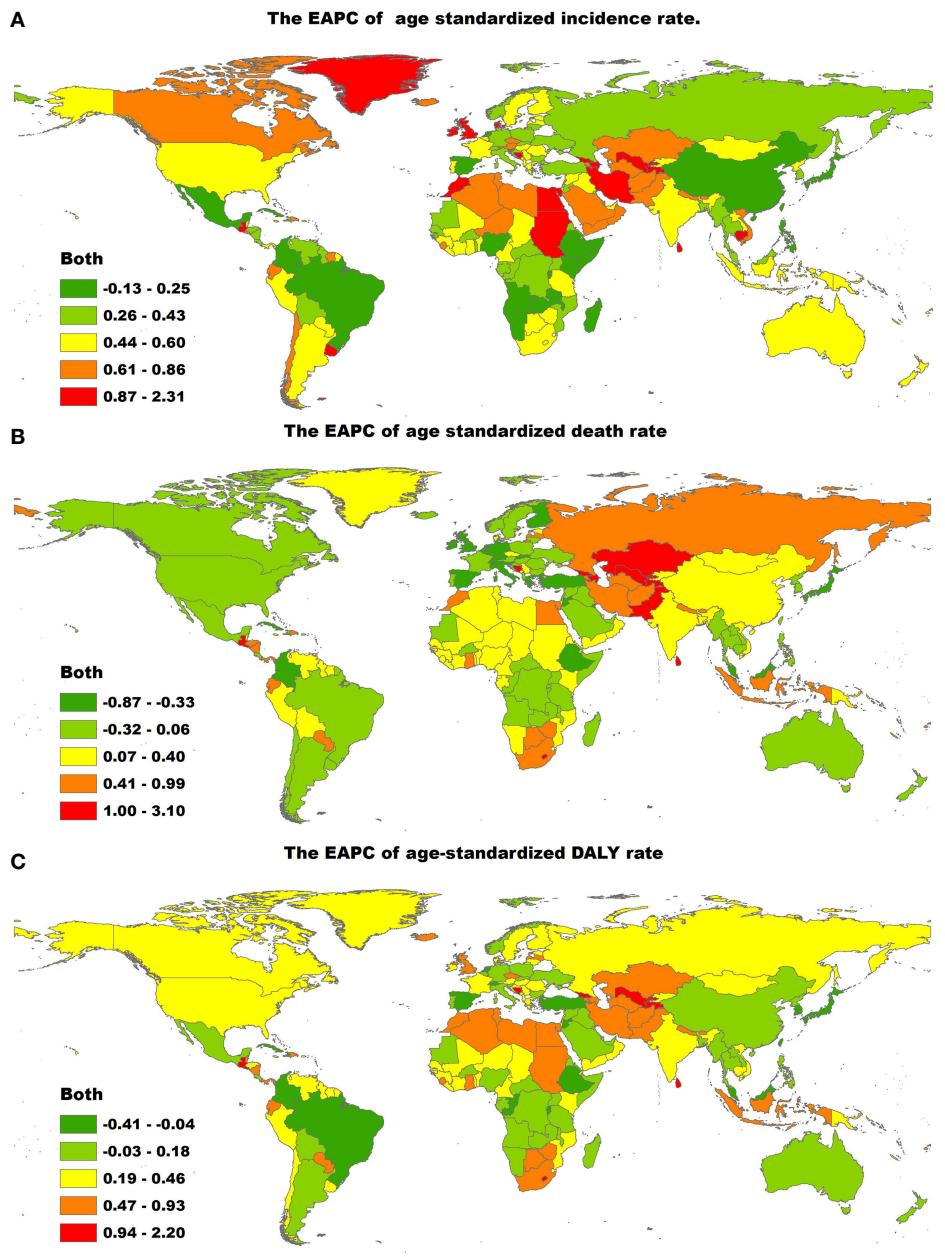
The global EAPC of T2DM age-standardized rates from 1990 to 2019, by countries. **(A)** The EAPC of age-standardized incidence rate. **(B)** The EAPC of age-standardized death rate. **(C)** The EAPC of age-standardized DALY rate. EAPC, estimated annual percentage change; ASIR, age-standardized incidence rate; ASDR, age-standardized death rate; DALY, disability adjusted life-year.

At the country level, in 2019, the highest age-standardized incidence rates of T2DM were in American Samoa (819.43 per 100,000 people; 95% UI, 762.75 to 882.16), Qatar (818.03 per 100,000 people; 95% UI, 773.89 to 868.70) and Fiji (797.04 per 100,000 people; 95% UI, 763.50 to 835.80), whereas the lowest age-standardized incidence rates of T2DM were in Mongolia (104.83 per 100,000 people; 95% UI, 94.17 to 116.66) (**Supplementary Table 1**, **3**). The largest increases in the age-standardized incidence rate of T2DM were in Luxembourg (EAPC, 4.51; 95% CI, 4.37 to 4.65), Uzbekistan (EAPC, 3.63; 95% CI, 3.44 to 3.82) and Ireland (EAPC, 3.54; 95% CI, 3.43 to 3.65) (**Figure 2A**; **Supplementary Table 1**, **3**). Age distributions of incidence rate (per 100,000) for T2DM in different countries in 2019 are shown in **Supplementary Table 6**.

### Change in the T2DM-Related Death Rate

As presented in **Table 2**, the number of global deaths caused by T2DM increased by 142.90% over a 30-year period, from 606,407.24 (95% UI, 573,068.74 to 637,507.97) in 1990 to 1,472,933.98 (95% UI, 1,371,939.55 to 1,565,860.35) in 2019. In contrast, the age-standardized T2DM-related death rate over the same period increased from 16.69 per 100,000 people (95% UI, 15.70 to 17.55) in 1990 to 18.49 per 100,000 people (95% UI, 17.18 to 19.66) in 2019, corresponding to an EAPC of 0.26 (95% CI, 0.16 to 0.37; **Table 2**; **Figure 1B**; **Supplementary Figure 1B**). The age-standardized death rate was slightly higher in males than in females (**Table 2**; **Figure 1B**; **Supplementary Figure 1B**).

**Table 2:** The age-standardized death rate (ASDR) of T2DM in 1990 and 2019 and its temporal trends.

Characteristics	1990		2019		1990-2019	
	ASDR (per 100000)		ASDR (per 100000)		Percent Change(%)	EAPC
	No. (95% UI)	Male/female ratio	No. (95% UI)	Male/female ratio		No. (95% CI)
**Global**	16.69(15.70,17.55)	1.042843144	18.49(17.18,19.66)	1.152488764	1.43(1.28,1.58)	0.26(0.16,0.37)
**Sex**	–	–	–	–	–	–
Male	17.10(16.06,18.07)	–	19.94(18.50,21.32)	–	1.62(1.42,1.83)	0.44(0.32,0.56)
Female	16.40(15.17,17.72)	–	17.30(15.62,18.70)	–	1.27(1.07,1.47)	0.09(0.00,0.18)
**Sociodemographic index**	–	–	–	–	–	–
Low SDI	30.50(26.93,33.72)	1.294156531	31.89(28.95,35.05)	1.168340652	1.29(1.03,1.57)	0.13(0.04,0.23)
Low-middle SDI	22.75(20.33,25.31)	1.069059538	29.05(26.46,31.48)	1.119848591	2.09(1.75,2.47)	0.84(0.70,0.97)
Middle SDI	19.98(18.75,21.23)	0.862254403	23.74(21.97,25.52)	1.03563898	2.06(1.81,2.30)	0.64(0.59,0.69)
High-middle SDI	13.27(12.50,13.88)	0.9682855	12.65(11.58,13.53)	1.153420905	0.94(0.81,1.08)	-0.22(-0.35,-0.10)
High SDI	11.72(10.94,12.14)	1.102113001	9.05(8.29,9.55)	1.471307813	0.51(0.44,0.56)	-1.39(-1.69,-1.09)
**Region**	–	–	–	–	–	–
Andean Latin America	19.39(17.64,21.63)	0.75544493	24.03(20.07,28.30)	0.884998411	2.50(1.86,3.19)	0.79(0.65,0.93)
Australasia	10.90(10.15,11.44)	1.356055044	8.74(7.80,9.45)	1.569637801	0.91(0.76,1.06)	-1.16(-1.47,-0.86)
Caribbean	40.96(38.12,43.67)	0.639232987	35.51(29.88,42.08)	0.86167106	0.79(0.53,1.10)	-0.64(-0.74,-0.55)
Central Asia	8.93(8.52,9.43)	1.04478539	23.56(21.42,26.09)	1.080937852	3.02(2.61,3.48)	3.03(2.67,3.40)
Central Europe	11.73(11.20,12.20)	0.929878747	11.92(10.36,13.59)	1.259891271	0.58(0.38,0.78)	0.26(0.14,0.39)
Central Latin America	43.45(41.19,44.73)	0.79875096	44.63(39.28,50.22)	1.124953332	2.02(1.67,2.38)	-0.17(-0.30,-0.04)
Central sub-Saharan Africa	42.99(37.19,49.20)	2.119535395	38.29(32.08,46.20)	1.979969854	1.09(0.64,1.64)	-0.47(-0.51,-0.44)
East Asia	9.15(8.19,10.27)	0.871110646	9.64(8.35,10.96)	1.230257331	1.59(1.14,2.08)	0.22(-0.05,0.49)
Eastern Europe	4.12(3.92,4.26)	0.85340176	6.11(5.40,6.82)	0.836872131	0.90(0.70,1.12)	0.82(0.19,1.45)
Eastern sub-Saharan Africa	41.20(36.25,46.70)	1.425251676	36.52(32.46,40.90)	1.535649052	0.84(0.50,1.13)	-0.52(-0.56,-0.47)
High-income Asia Pacific	7.79(7.31,8.19)	1.318340938	4.22(3.74,4.59)	1.606327957	0.43(0.24,0.56)	-2.41(-2.61,-2.21)
High-income North America	13.25(12.38,13.72)	1.110224983	12.29(11.39,12.86)	1.488918083	0.68(0.62,0.74)	-0.93(-1.42,-0.44)
North Africa and middle East	24.81(22.46,27.66)	0.722365636	25.23(22.39,28.18)	0.827072141	1.62(1.30,1.98)	-0.64(-0.78,-0.50)
Oceania	87.72(73.85,107.45)	1.547130367	121.02(100.23,146.50)	1.386460507	2.27(1.53,3.10)	0.96(0.73,1.19)
South Asia	22.07(19.08,25.84)	1.135962828	28.15(25.00,31.61)	1.082377922	2.55(1.99,3.17)	2.12(1.91,2.32)
Southeast Asia	32.44(29.10,35.45)	0.920035166	38.06(34.12,41.66)	1.047462238	1.84(1.49,2.24)	0.62(0.55,0.70)
Southern Latin America	19.56(18.51,20.32)	1.205223302	17.38(16.08,18.45)	1.430923983	0.71(0.61,0.81)	-0.83(-1.06,-0.60)
Southern sub-Saharan Africa	42.64(37.69,47.30)	0.925536309	68.50(63.21,73.76)	1.030536205	2.19(1.87,2.54)	2.02(1.54,2.50)
Tropical Latin America	31.83(29.78,33.28)	0.77064611	28.02(25.28,29.60)	1.039717953	1.51(1.36,1.63)	-0.41(-0.46,-0.36)
Western Europe	13.06(12.12,13.56)	0.97491343	8.55(7.64,9.07)	1.339815651	0.19(0.11,0.25)	-1.71(-1.83,-1.59)
Western sub-Saharan Africa	29.45(25.56,33.61)	1.178990272	35.59(30.83,40.23)	1.126231958	1.51(1.11,1.94)	0.70(0.52,0.88)

ASDR, age-standardized death rate; EAPC, estimated annual percentage change; NA, not available; UI, uncertainty interval.

The age-standardized death rate due to T2DM increased in most SDI quintiles, except in high- and high-middle-SDI quintiles (**Table 2**; **Figure 1B**; **Supplementary Figure 1B**). In 2019, low-SDI (31.89 per 100,000 people; 95% UI, 28.95 to 35.05) had the highest age-standardized T2DM-related death rates, whereas high-SDI regions had the lowest (9.05 per 100,000 people; 95% UI, 8.29 to 9.55). The age-standardized T2DM-related death rate showed the largest decrease in high-SDI regions (EAPC, −1.39; 95% CI, −1.69 to −1.09; **Table 2**; **Figure 1B**; **Supplementary Figure 1B**).

Of note, regions with higher SDI values had lower proportions of young people dying from T2DM. However, the proportion of elderly people dying from T2DM increased in regions whose SDI values increased from 1990 to 2019 (**Supplementary Figures 4C, D**). From 1990 to 2019, the annual proportion of elderly people dying from T2DM was relatively stable (**Supplementary Figure 5B**). There was also an increase in mortality due to T2DM with increasing age in both sexes, especially in people aged greater than 60 years (**Supplementary Figure 9A**). Globally, the male-to-female ratio peaked in the 30–34-year age group (**Supplementary Figure 6A**). In addition, the EAPC in the age-standardized death rate due to T2DM was positively correlated with the age-standardized death rate (ρ = 0.073, *P* = 0.299, **Supplementary Figure 3C**) and negatively correlated with the SDI value (ρ = −0.370, *P*=0.001, **Supplementary Figure 3D**).

At the regional level, the highest age-standardized death rates due to T2DM in 2019 were in Oceania (121.02 per 100,000 people; 95% UI, 100.23 to 146.50), Southern Sub-Saharan Africa (68.50 per 100,000 people; 95% UI, 63.21 to 73.76) and Central Latin America (44.63 per 100,000 people; 95% UI, 39.28 to 50.22), whereas the lowest age-standardized death rates due to T2DM were in the high-income Asia Pacific region (4.22 per 100,000 people; 95% UI, 3.74 to 4.59; **Table 2**; **Supplementary Table 2**). The age-standardized death rate due to T2DM showed the largest increase in Central Asia (EAPC, 3.03; 95% CI, 2.67 to 3.40), South Asia (EAPC, 2.12; 95% CI, 1.91 to 2.32) and Southern Sub-Saharan Africa (EAPC, 2.02; 95% CI, 1.54 to 2.50) and the largest decrease in the high-income Asia Pacific region (EAPC, −2.41; 95% CI, −2.61 to −2.21; **Table 2**; **Supplementary Table 2**; **Figure 1B**).

The countries with the highest age-standardized death rates due to T2DM in 2019 were Fiji (257.38 per 100,000 people; 95% CI, 210.35 to 309.25), Kiribati (203.99 per 100,000 people; 95% UI, 158.28 to 252.68) and the Federated States of Micronesia (169.13 per 100,000 people; 95% UI, 126.79 to 226.11), whereas those with the lowest age-standardized death rates due to T2DM in 2019 were Japan (1.96 per 100,000 people, 95% CI, 1.68 to 2.13) (**Supplementary Table 1**, **4**). The age-standardized death rate due to T2DM showed the largest decrease in Singapore (EAPC, −7.24; 95% CI, −8.40 to −6.07) and the largest increase in Mauritius (EAPC, 5.09; 95% CI, 4.07 to 6.11), Uzbekistan (EAPC, 4.73; 95% CI, 4.04 to 5.43) and Bosnia and Herzegovina (EAPC, 4.36; 95% CI, 3.66 to 5.06; **Figure 2B**; **Supplementary Table 1**, **4**). Age distributions of death rate (per 100,000) for T2DM in different countries in 2019 are shown in **Supplementary Table 7**.

### Change in the Number of DALYs due to T2DM

The total global number of DALYs due to T2DM in 1990 was 25,478,098.44 (95% UI, 21,701,410.79 to 29,776,366.95), and this increased to 66,299,750.62 (95% UI, 55,477,041.86 to 79,005,166.07) in 2019. The age-standardized DALY rate of T2DM increased from 1990 to 2019, with an EAPC of 0.81 (95% CI, 0.77 to 0.85; **Table 3**; **Figure 1C**; **Supplementary Figure 1C**). From 1990 to 2019, the age-standardized DALY rate of T2DM was higher in males than in females (**Table 3**; **Figure 1C**; **Supplementary Figure 1C**).

**Table 3:** The age-standardized DALY rate of T2DM in 1990 and 2019 and its temporal trends.

Characteristics	1990		2019		1990-2019	
	age-standardized DALY rate (per 100000)		age-standardized DALY rate (per 100000)		Percent Change(%)	EAPC
	No. (95% UI)	Male/female ratio	No. (95% UI)	Male/female ratio		No. (95% CI)
**Global**	628.33(537.22,730.86)	1.071866729	801.55(670.58,954.43)	1.163296672	1.43(1.28,1.58)	0.81(0.77,0.85)
**Sex**	–	–	–	–	–	–
Male	651.72(556.48,759.65)	–	865.16(721.20,1030.70)	–	1.62(1.42,1.83)	0.94(0.89,1.00)
Female	608.02(519.01,706.84)	–	743.71(621.81,888.99)	–	1.27(1.07,1.47)	0.67(0.63,0.71)
**Sociodemographic index**	–	–	–	–	–	–
Low SDI	920.91(801.64,1049.52)	1.257938069	1064.45(915.04,1236.74)	1.163245643	1.29(1.03,1.57)	0.43(0.37,0.48)
Low-middle SDI	756.32(647.54,870.30)	1.067903066	1049.75(891.23,1231.42)	1.130162977	2.09(1.75,2.47)	1.05(0.99,1.10)
Middle SDI	719.82(620.48,836.39)	0.929619279	915.14(780.40,1075.78)	1.084685968	2.06(1.81,2.30)	0.82(0.78,0.86)
High-middle SDI	534.06(449.26,634.04)	1.024492903	610.16(492.12,743.69)	1.162916792	0.94(0.81,1.08)	0.51(0.40,0.62)
High SDI	483.95(401.58,579.52)	1.216394038	584.69(454.63,735.02)	1.385521543	0.51(0.44,0.56)	0.61(0.56,0.65)
**Region**	–	–	–	–	–	–
Andean Latin America	653.63(562.86,763.69)	0.853139953	873.52(722.30,1045.26)	0.966648679	2.50(1.86,3.19)	0.98(0.90,1.06)
Australasia	362.06(306.08,426.59)	1.290648939	419.59(320.65,531.67)	1.387093295	0.91(0.76,1.06)	0.19(0.03,0.35)
Caribbean	1407.93(1222.53,1622.46)	0.777979851	1483.07(1226.00,1800.55)	0.969760961	0.79(0.53,1.10)	0.05(-0.03,0.13)
Central Asia	460.41(375.28,556.80)	0.959629766	1013.76(841.93,1219.13)	1.023540275	3.02(2.61,3.48)	2.62(2.40,2.84)
Central Europe	580.37(470.41,703.66)	1.065310188	730.22(558.96,923.08)	1.310355713	0.58(0.38,0.78)	0.88(0.77,0.98)
Central Latin America	1575.57(1375.76,1799.38)	0.908553899	1746.50(1485.31,2073.88)	1.167406709	2.02(1.67,2.38)	0.18(0.07,0.29)
Central sub-Saharan Africa	1248.94(1071.94,1453.26)	1.942568266	1265.08(1049.07,1533.16)	1.774475131	1.09(0.64,1.64)	0.01(-0.02,0.04)
East Asia	447.15(361.61,546.90)	0.940112917	487.64(387.78,602.69)	1.198660239	1.59(1.14,2.08)	0.37(0.14,0.59)
Eastern Europe	281.46(221.94,347.76)	0.941146843	375.97(295.07,468.17)	0.994039906	0.90(0.70,1.12)	0.76(0.61,0.92)
Eastern sub-Saharan Africa	1122.45(985.24,1286.48)	1.369907452	1026.82(889.81,1182.42)	1.461407358	0.84(0.50,1.13)	-0.41(-0.44,-0.37)
High-income Asia Pacific	383.01(312.67,469.06)	1.517578162	383.16(285.32,495.52)	1.533542846	0.43(0.24,0.56)	-0.35(-0.48,-0.22)
High-income North America	589.79(492.51,705.35)	1.18922324	748.23(596.04,931.14)	1.389588043	0.68(0.62,0.74)	1.06(0.92,1.21)
North Africa and middle East	808.33(696.87,944.32)	0.821574609	1060.79(872.05,1279.05)	0.935482533	1.62(1.30,1.98)	0.46(0.32,0.59)
Oceania	2592.69(2186.09,3065.54)	1.460893916	3703.43(3060.00,4399.34)	1.32881183	2.27(1.53,3.10)	1.13(0.92,1.34)
South Asia	732.00(616.47,856.63)	1.138218448	1049.71(869.25,1244.94)	1.122106164	2.55(1.99,3.17)	1.56(1.46,1.66)
Southeast Asia	1002.91(885.23,1132.40)	0.967301997	1273.42(1103.92,1452.41)	1.079967333	1.84(1.49,2.24)	0.83(0.75,0.91)
Southern Latin America	606.31(528.93,700.23)	1.148906166	722.37(588.21,878.55)	1.278581482	0.71(0.61,0.81)	0.28(0.14,0.42)
Southern sub-Saharan Africa	1226.53(1085.25,1368.28)	0.927130268	1877.98(1679.95,2098.75)	1.029491984	2.19(1.87,2.54)	1.79(1.39,2.19)
Tropical Latin America	1082.24(952.21,1232.62)	0.914918457	1020.44(868.03,1194.54)	1.121786308	1.51(1.36,1.63)	-0.13(-0.19,-0.08)
Western Europe	458.39(381.20,551.61)	1.098109628	515.75(389.52,663.92)	1.307180948	0.19(0.11,0.25)	0.27(0.21,0.32)
Western sub-Saharan Africa	794.34(686.62,911.39)	1.129114465	994.82(851.01,1148.43)	1.100176005	1.51(1.11,1.94)	0.79(0.64,0.93)

DALY, disability adjusted life-years; NA, not available; UI, uncertainty interval.

In 2019, the highest age-standardized number of DALYs due to T2DM was observed in countries in the low-SDI quintile (1,064.45 per 100,000 people; 95% UI, 915.04 to 1,236.74); **Table 3**). The age-standardized number of DALYs due to T2DM increased in all SDI regions, with the largest increase occurring in low-middle-SDI regions (1.05; 95% CI, 0.99 to 1.10; **Table 3**; **Figure 1C**; **Supplementary Figure 1C**).

In addition, the EAPC in the age-standardized DALY rate due to T2DM was negatively correlated with the age-standardized DALY rate (ρ = −0.127, *P* = 0.071, **Supplementary Figure 3E**) and the SDI value (ρ = −0.142, *P* = 0.042, **Supplementary Figure 3F**). In all regions, regardless of the SDI value, the DALY rate due to T2DM increased with increasing age (**Supplementary Figure 10**). Globally, the male-to-female ratio of the number of DALYs due to T2DM in various age groups peaked in the 45–49-year age group (**Supplementary Figure 7A**).

In 2019, the regions with the highest age-standardized number of DALYs due to T2DM were Oceania (3,703.43 per 100,000 people; 95% UI, 3,060.00 to 4,399.34), Southern Sub-Saharan Africa (1,877.98 per 100,000 people; 95% UI, 1,679.95 to 2,098.75) and Central Latin America (1,746.50 per 100,000 people; 95% UI, 1,485.31 to 2,073.88). The regions with the lowest age-standardized number of DALYs due to T2DM were Eastern Europe (375.97 per 100,000 people; 95% UI, 295.07 to 468.17; **Table 3**, **Supplementary Table 2**). The age-standardized number of DALYs due to T2DM showed the largest increase in Central Asia (EAPC, 2.62; 95% CI, 2.40 to 2.84), Southern Sub-Saharan Africa (EAPC, 1.79; 95% CI, 1.39 to 2.19) and South Asia (EAPC, 1.56; 95% CI, 1.46 to 1.66; **Table 3**; **Figure 1C**; **Supplementary Table 2**).

The countries with the highest age-standardized DALY rates of T2DM in 2019 were Fiji (6,884.30, 95% UI, 5,667.75 to 8,214.82), Kiribati (6,161.41, 95% UI, 4,876.73 to 7,611.34) and the Federated States of Micronesia (4,896.31, 95% UI, 3,682.18 to 6,620.37). The countries with the lowest age-standardized DALY rates of T2DM in 2019 were France (278.20, 95% UI, 220.64 to 345.74). The age-standardized DALY rate of T2DM showed the largest decrease in Cyprus (EAPC, −2.18; 95% CI, −2.37 to −1.99) and the largest increase in Mauritius (EAPC, 4.04, 95% CI, 3.29 to 4.80) (**Figure 2C**; **Supplementary Table 1**, **5**). Age distributions of death rate (per 100,000) for T2DM in different countries in 2019 are shown in **Supplementary Table 8**.

## Discussion

By analyzing data from the GBD 2019 database, we estimated the age-standardized incidence, death and DALY rates of T2DM in 204 countries and 21 regions from 1990 to 2019. Moreover, we evaluated regional differences in T2DM burden and investigated how this burden has changed over the past 30 years. The results of this study may help policymakers in different countries understand the burden of T2DM in their region and in other countries or regions.

We found that the age-standardized incidence, death and DALY rates of T2DM increased globally from 1990 to 2019. With rapid economic development, globalization has made low-cost, high-calorie, over-processed foods more accessible and cost-effective. Thus, traditional low-fat, high-fiber diets comprising lightly processed foods have been replaced with high-calorie, high-saturated fatty acid, high-sugar, high-salt diets comprising highly processed foods (11, 12). In addition, industrialization and urbanization have led to changes in work styles, increase in sedentary behavior, reduction in activity levels and increase in air pollution and environmental pollution. Consequently, the risk of T2DM has also increased (11, 13, 14).

Notably, in 2019, the highest age-standardized incidence rate of T2DM was observed in high-SDI regions, and the largest increase in this rate from 1990 to 2019 was also in high-SDI regions. Residents of developed countries have higher educational levels than those in lower-SDI countries. A multi-national, prospective cohort study indicated that primary education level or less was identified in the majority of participants from low-income countries (54.0%), in 43.8% from middle-income countries, and 13.2% in high-income countries. By contrast, the proportion of participants with a college was highest in high-income countries (58.0%), and lowest in low-income countries (12.7%) (15). Moreover, countries with higher SDI values have more adequate medical and health resources and commonly have T2DM screening programs; thus, the early diagnosis rate of T2DM is high in these countries (4, 15, 16).

However, the age-standardized death rate and number of DALYs due to T2DM were highest in countries in the low-SDI quintile in 2019, and the age-standardized DALY rate showed the largest increase in low-middle-SDI regions, suggesting that T2DM is a major issue in countries with less economic development. These findings show that while low-SDI countries and regions have made progress in economic and social development, chronic diseases, such as T2DM, have also begun to become more prevalent in these countries and regions, resulting in a large disease burden (17, 18). This may also be related to factors such as limited healthcare resources and medical and health technology in countries and regions with low SDI values (4). Therefore, countries and regions with low SDI values urgently need to improve their medical and healthcare systems, increase the availability of medical and health technology, and establish a sound healthcare system to help reduce the burden of T2DM.

At the regional level, the disease burden of T2DM varied widely. In 2019, the regions with the highest age-standardized incidence rates of T2DM were Oceania, Central Latin America and the Caribbean. From 1990 to 2019, the age-standardized incidence rate of T2DM showed the largest increase in Central Asia, whereas the age-standardized number of DALYs and age-standardized death rates due to T2DM showed the largest increases in Central Asia, Southern Sub-Saharan Africa and South Asia. As developing countries have experienced economic globalization and economic transformation, living and eating behaviors, such as dietary patterns (high intake of fast foods, refined grains, sugar-sweetened soft drinks and processed meats) and levels of physical inactivity have also changed and life expectancy has increased. These factors are thought to play key roles in determining the prevalence of T2DM (11, 12, 19). Moreover, obesity is closely related to T2DM, and the obesity rates in the aforementioned regions are generally high (20).

Notably, the age-standardized incidence, death and DALY rates of T2DM from 1990 to 2019 were higher in males than in females. The sex distribution of T2DM is closely related to lifestyle, educational level, socio-economic status and cultural factors, in addition to the physiological and metabolic differences between males and females (21, 22). Women generally pay more attention to the disease, have high treatment compliance and have a greater awareness of T2DM self-management, which help improve the prognosis of T2DM and reduce the incidence of complications (23). However, the difference in disease burden between men and women was found to differ between different countries or regions (16, 24, 25). Thus, targeted interventions, based on the distribution characteristics of T2DM, should be considered when formulating prevention and control measures.

The incidence of T2DM was found to peak in the 60–64-year age group, and the burden of T2DM was found to increase with age (26), with death and DALY rates peaking in the 90–94-year age group. Age is an independent risk factor for T2DM (27–29). The characteristics of the age distribution of T2DM also suggest that the burden of disease is increasing as the population ages.

The aim of this study was to comprehensively evaluate the distribution of the disease burden of T2DM and determine the relationship between T2DM and the level of socio-demographic development. Moreover, we aimed to analyze the characteristics of the disease burden of T2DM in each country and region, including those with limited medical and health resources, to facilitate more reasonable and targeted policy development and resource allocation. By comparing and analyzing the relationship between the SDI value and T2DM morbidity, mortality and DALY rates, the differences in T2DM disease burden in different countries or regions were comprehensively assessed.

There are some shortcomings of this study that should be noted. First, there may be some deficiencies in the statistical methods used in the GBD 2019. For example, the inclusion of original data may have introduced bias, data may be incomplete for some countries and the data quality is relatively low. Second, internal economic development is unbalanced and does not fully reflect the differences between countries or regions.

From 1990 to 2019, there was a significant global increase in the disease burden of T2DM, and the age-standardized incidence, death and DALY rates of T2DM also increased globally during that period. Current prevention strategies need to be recalibrated and more targeted and specific strategies need to be established in countries with high age-standardized incidence, mortality or DALY rates, to prevent an increase in the burden of T2DM.

## Data Availability Statement

The original contributions presented in the study are included in the article/**Supplementary Material**. Further inquiries can be directed to the corresponding author.

## Author Contributions

Conceptualization, RZ and XB. Methodology and statistical analysis, RZ, SZ and LX. Data curation, RZ and XB. Writing—original draft preparation, RZ. Writing—review and editing, RZ and XB. All authors have read and agreed to the published version of the manuscript.

## Funding

This work was supported by the Ningbo Health Branding Subject Fund (PPXK2018-01).

## Conflict of Interest

The authors declare that the research was conducted in the absence of any commercial or financial relationships that could be construed as a potential conflict of interest.

## Publisher’s Note

All claims expressed in this article are solely those of the authors and do not necessarily represent those of their affiliated organizations, or those of the publisher, the editors and the reviewers. Any product that may be evaluated in this article, or claim that may be made by its manufacturer, is not guaranteed or endorsed by the publisher.
