# Supplementary material for: Incidence, Morbidity and years Lived With Disability due to Type 2 Diabetes Mellitus in 204 Countries and Territories: Trends From 1990 to 2019
Source: Front Endocrinol (Lausanne). 2022 Jul 11;13:905538. doi: 10.3389/fendo.2022.905538 (PMC9309695; doi:10.3389/fendo.2022.905538)
Supplement: Supplementary file 1 [file Image_1.pdf]

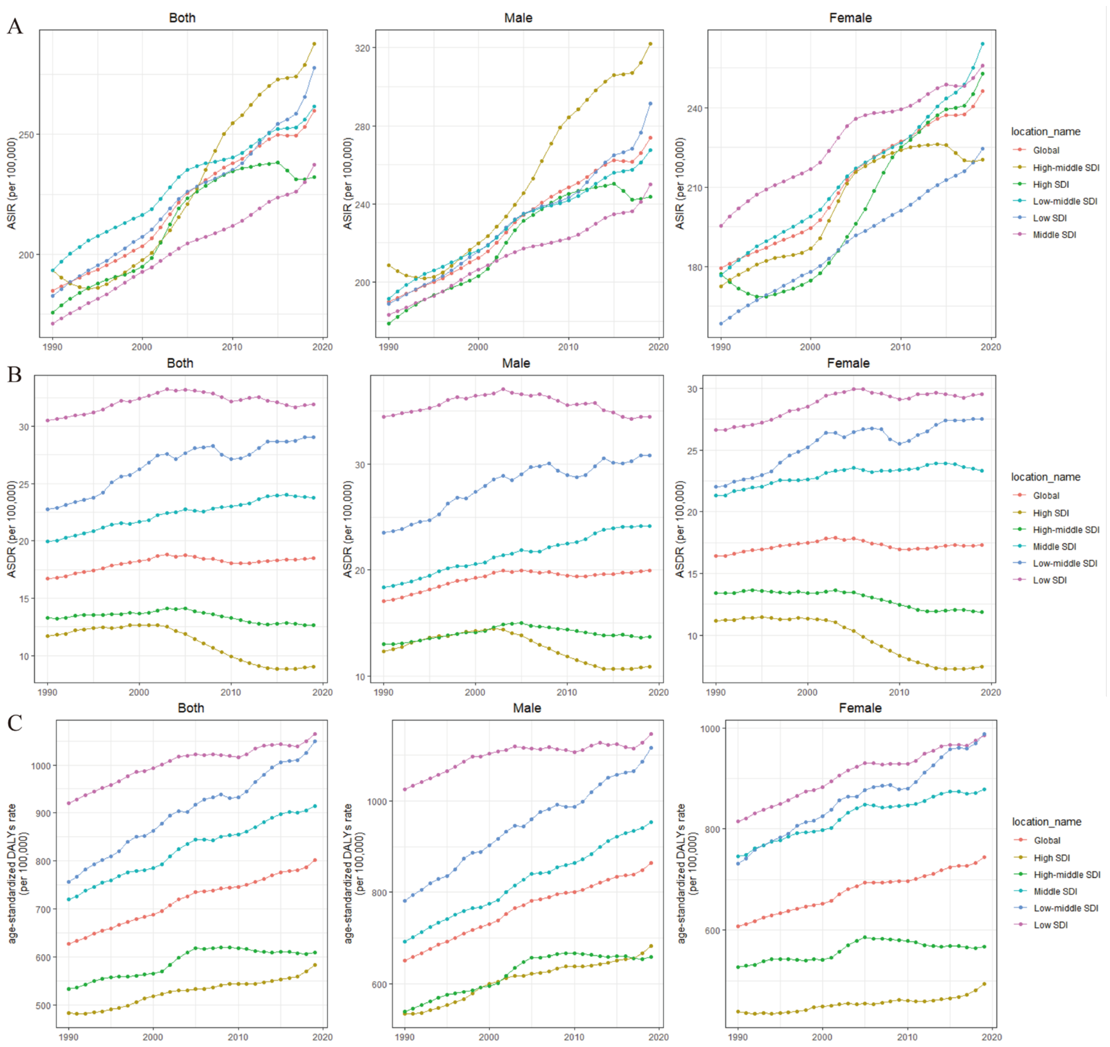

Supplementary Figure 1. The change trends of age-standardized T2DM incidence, death, and DALY rate among different SDI quintiles and gender from 1990 to 2019. (A) ASIR, age standardized incidence rate. (B) ASDR, age standardized death rate. (C) age-standardized DALY rate. Abbreviations: DALY = disability adjusted life-year.

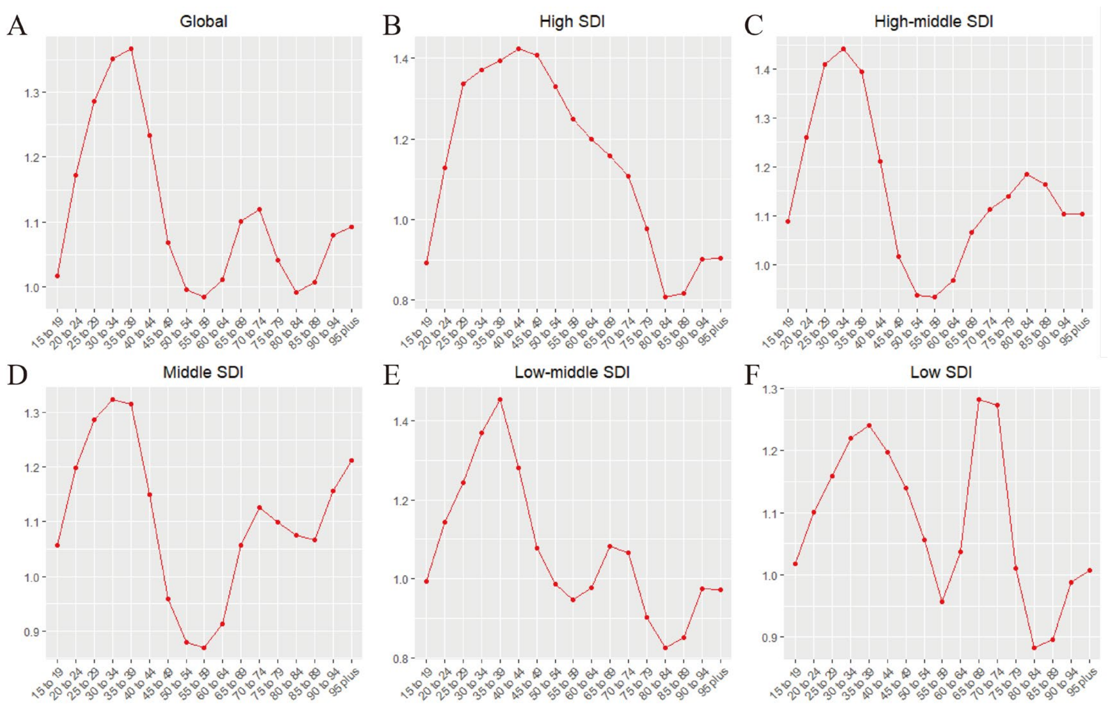

Supplementary Figure 2. The ratio of male to female incidence among different age groups in 2019. (A) Global. (B) High SDI. (C) High-middle SDI. (D) Middle SDI. (E) Middle-low SDI. (F) Low SDI. Abbreviations: SDI = socio-demographic index.

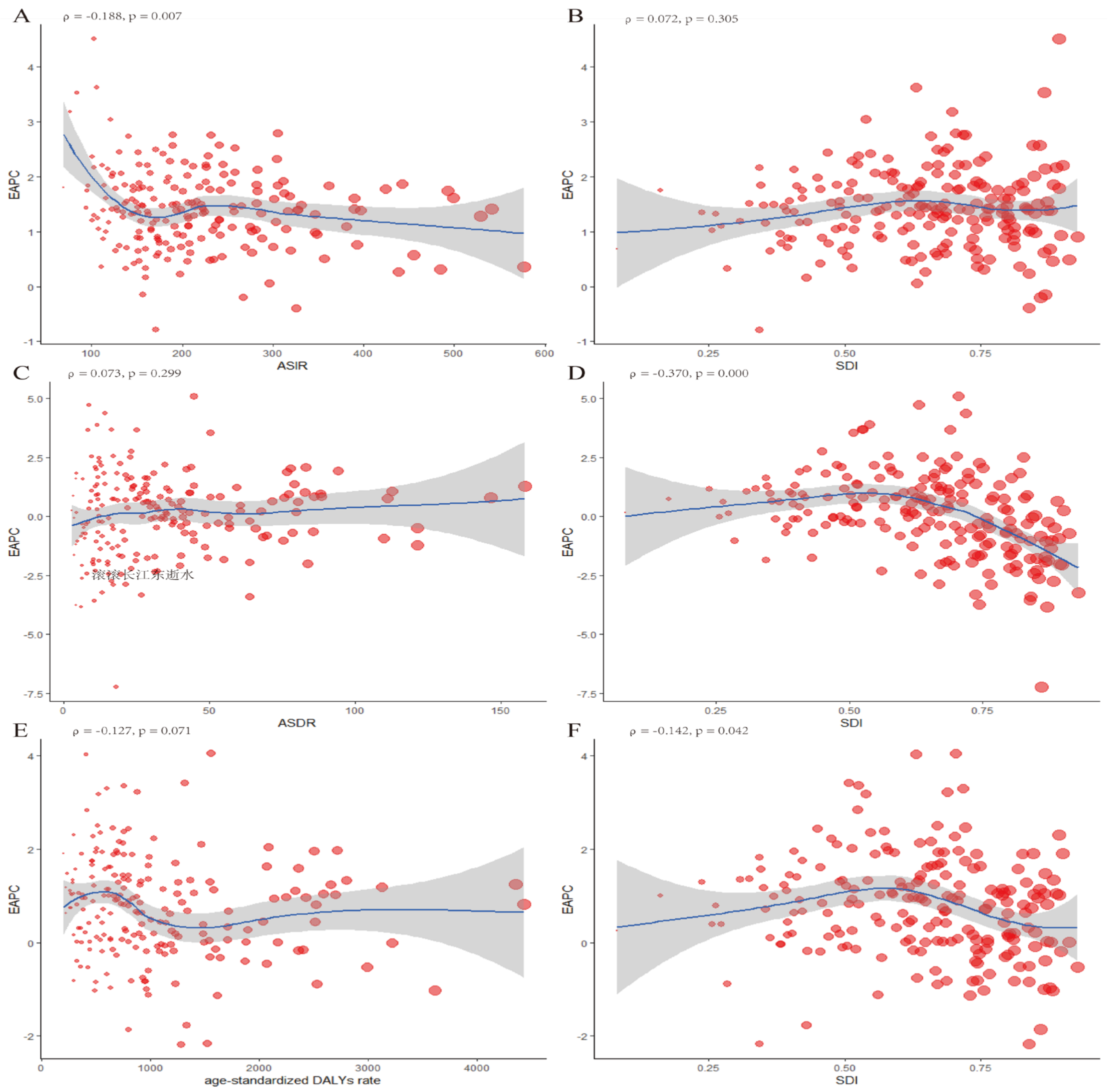

Supplementary Figure 3. The correlation between EAPC and T2DM age-standardized rates in 1990 and SDI in 2019. The circles represent countries that were available on SDI data. The size of circle is increased with the cases of T2DM. The ρ indices Pearson's correlation coefficient and p values were derived from Pearson's correlation analysis. (A) EAPC and ASIR. (B) EAPC and SDI in incidence. (C) EAPC and ASDR. (D) EAPC and SDI in death. (E) EAPC and age-standardized DALY rate. (F) EAPC and SDI in DALYs. Abbreviations: EAPC = estimated annual percentage change. SDI = socio-demographic index. ASIR = age standardized incidence rate. ASDR = age standardized death rate. DALY = disability adjusted life-year.

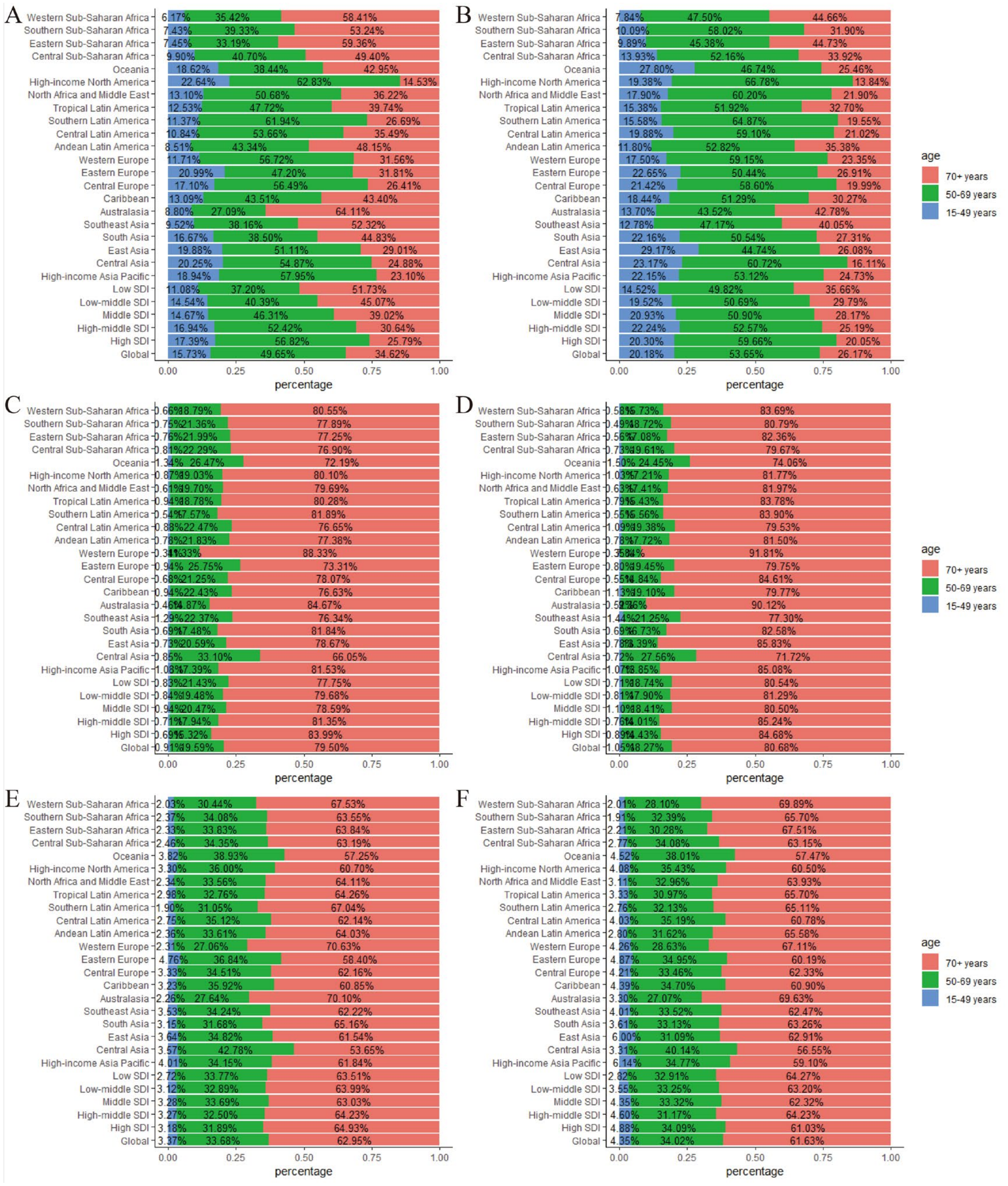

supplementary Figure 4. The incidence, death, and DALY rates of T2DM in different age groups. (A) incidence in 1990. (B) incidence in 2019. (C) Death rate in 1990. (D) Death rate in 2019. (E) DALY rate in 1990. (F) DALY rate in 2019. Abbreviations: DALY = disability adjusted life-year.

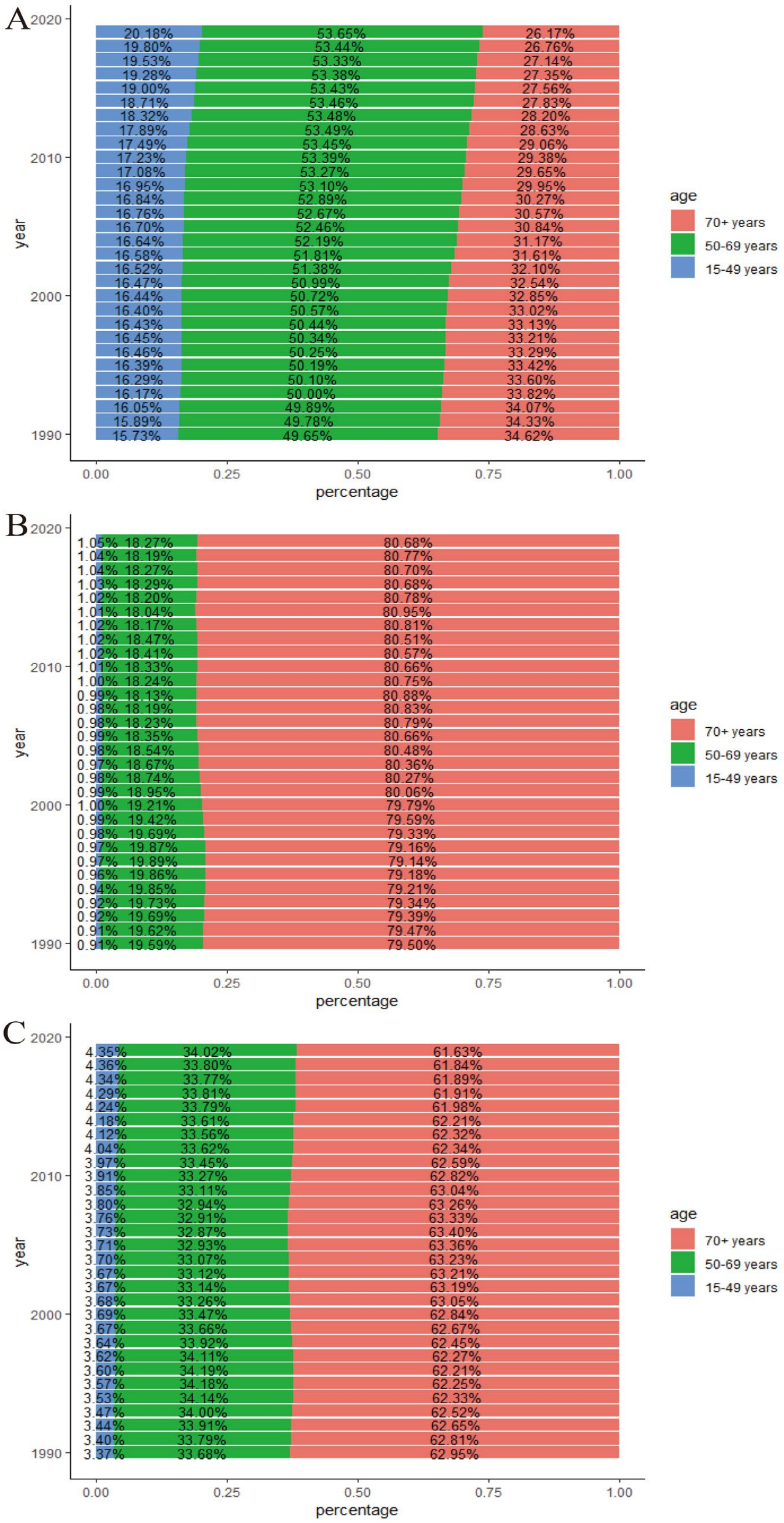

Supplementary Figure 5. The proportion of different ages in T2DM incidence (A) and death (B) and age-standardized DALY (C) by years. Abbreviations: DALY = disability adjusted life-year.

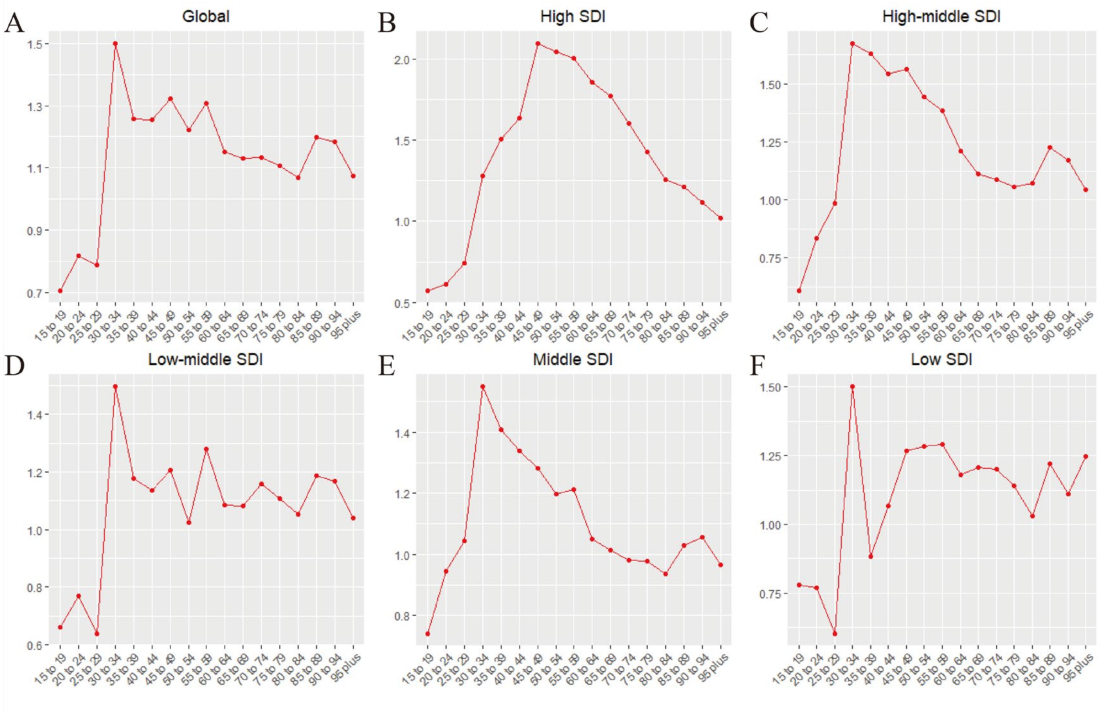

Supplementary Figure 6. The ratio of male to female death among different age groups in 2019. (A) Global. (B) High SDI. (C) High-middle SDI. (D) Middle SDI. (E) Middle-low SDI. (F) Low SDI. Abbreviations: SDI = socio-demographic index.

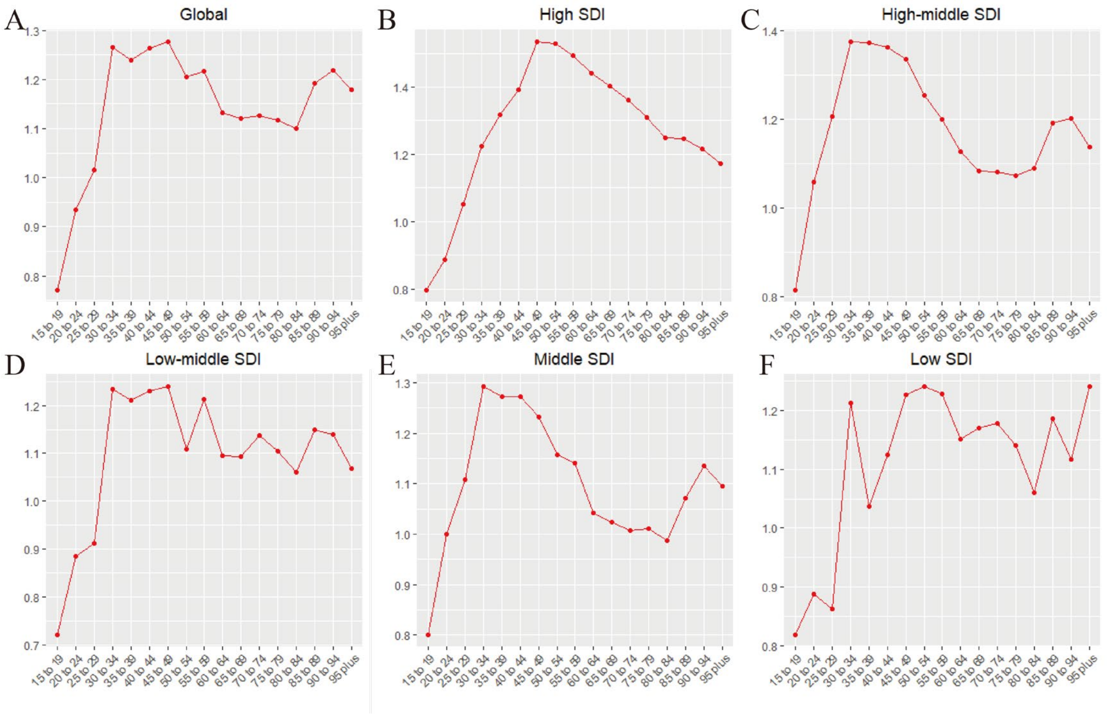

Supplementary Figure 7. The ratio of male to female age standardized DALY rate among different age groups in 2019. (A) Global. (B) High SDI. (C) High-middle SDI. (D) Middle SDI. (E) Middle-low SDI. (F) Low SDI. Abbreviations: SDI = socio-demographic index.

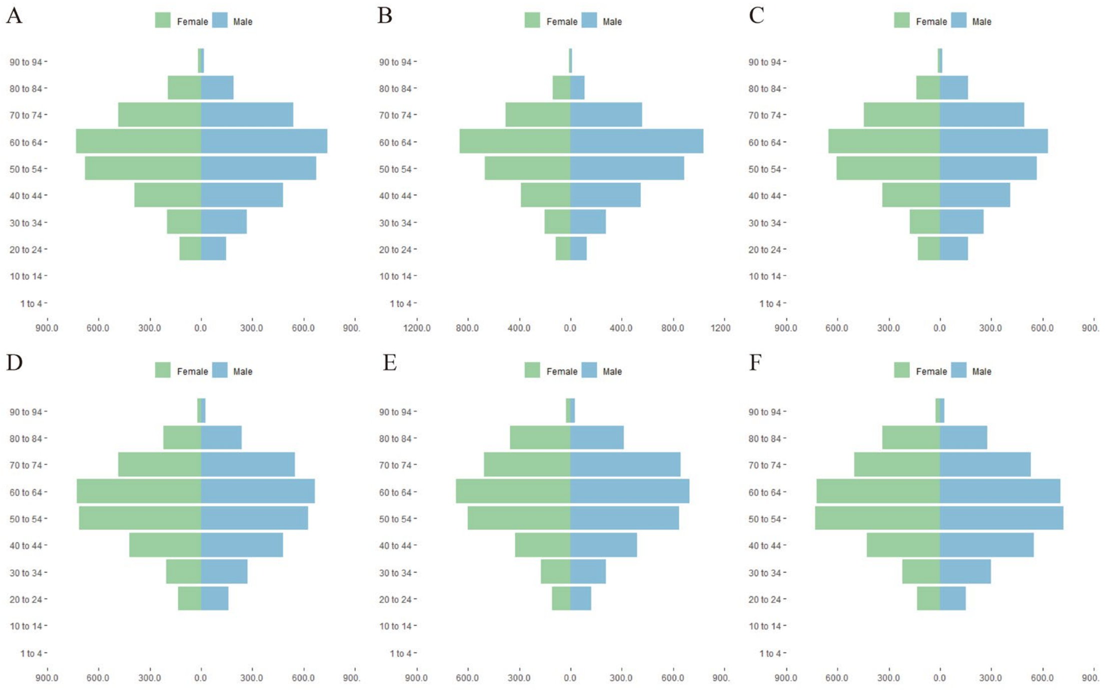

Supplementary Figure 8. Distribution of different ages in T2DM incidence in global (A), high SDI (B), high-middle SDI (C), middle SDI (D), middle-low SDI (E), low SDI (F). Abbreviations: SDI, socio-demographic index.

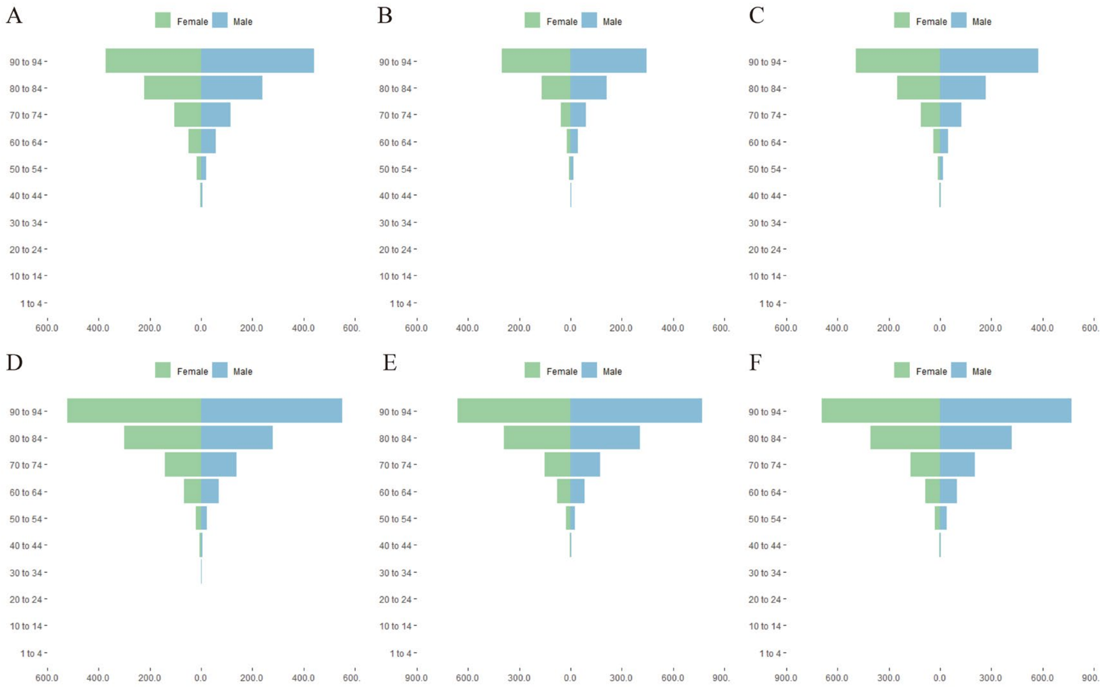

Supplementary Figure 9. Distribution of different ages in T2DM death in global (A), high SDI (B), high-middle SDI (C), middle SDI (D), middle-low SDI (E), low SDI (F). Abbreviations: SDI, socio-demographic index.

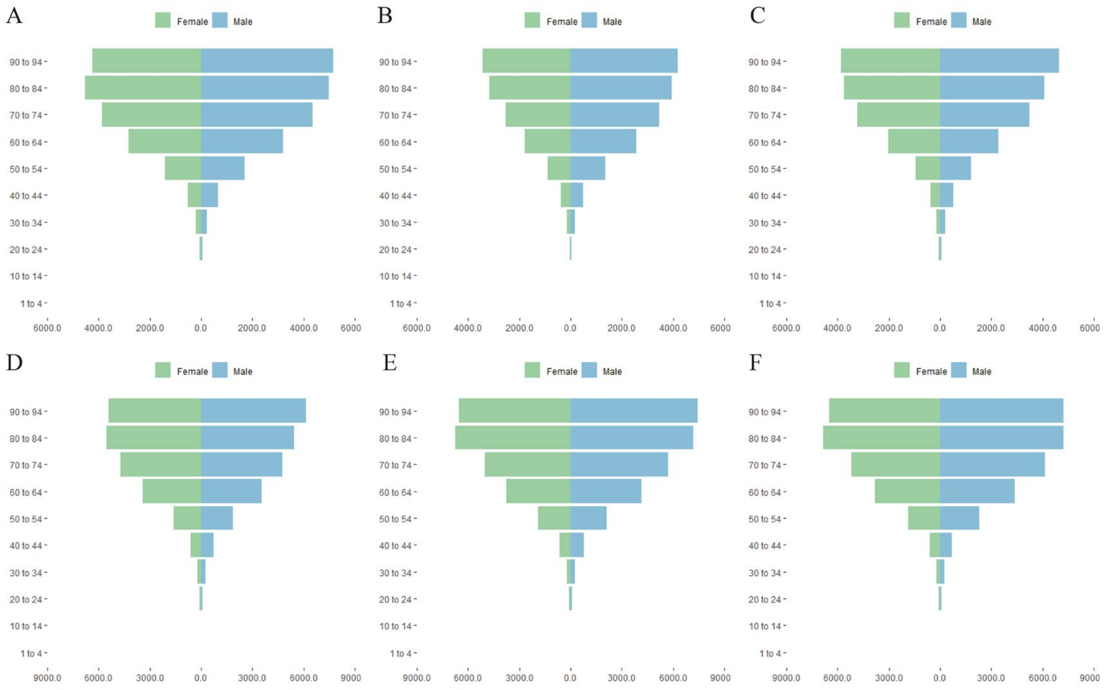

Supplementary Figure 10. Distribution of different ages in T2DM DALYs in global (A), high SDI (B), high-middle SDI (C), middle SDI (D), middle-low SDI (E), low SDI (F). Abbreviations: Abbreviations: SDI, socio-demographic index.
