# Supplementary material for: Incidence, Morbidity and years Lived With Disability due to Type 2 Diabetes Mellitus in 204 Countries and Territories: Trends From 1990 to 2019
Source: Front Endocrinol (Lausanne). 2022 Jul 11;13:905538. doi: 10.3389/fendo.2022.905538 (PMC9309695; doi:10.3389/fendo.2022.905538)
Supplement: Supplementary file 2 [file Table_1.docx]

**Supplementary Table 1.** The top three and the bottom three countries of type 2 diabetes incidence, death, or DALY.

| **Measure** | **sex** | **Top three countries** | | | **Bottom three countries** | | |
| --- | --- | --- | --- | --- | --- | --- | --- |
| **2019 ASR (per 100,000 people)** | | | | | | | |
| **ASIR** |  |  |  |  |  |  |  |
|  | both | Fiji(797.04) | Qatar(818.03) | American Samoa(819.43) | Mongolia(104.83) | Belarus(126.90) | France(140.55) |
|  | female | Marshall Islands(796.35) | American Samoa(805.05) | Qatar(821.66) | Mongolia(99.37) | Belarus(121.12) | Kenya(124.64) |
|  | male | Qatar(816.78) | American Samoa(835.09) | Fiji(840.99) | Mongolia(110.90) | Belarus(133.67) | Sierra Leone(136.33) |
| **ASDR** |  |  |  |  |  |  |  |
|  | both | Micronesia (Federated States of)(169.13) | Kiribati(203.99) | Fiji(257.38) | Japan(1.96) | Belarus(2.21) | Singapore(2.39) |
|  | female | Qatar(173.19) | Kiribati(180.06) | Fiji(245.35) | Japan(1.43) | Belarus(1.99) | Iceland(2.13) |
|  | male | Eswatini(170.78) | Kiribati(237.78) | Fiji(280.13) | Japan(2.55) | Singapore(2.64) | Belarus(2.69) |
| **Age-standardized DALY rate** |  |  |  |  |  |  |  |
|  | both | Micronesia (Federated States of)(4896.31) | Kiribati(6161.41) | Fiji(6884.30) | France(278.20) | Belarus(278.74) | Japan(313.75) |
|  | female | Marshall Islands(4840.33) | Kiribati(5329.06) | Fiji(6667.04) | France(231.74) | Japan(241.26) | Belarus(268.30) |
|  | male | Micronesia (Federated States of)(4933.05) | Fiji(7216.35) | Kiribati(7223.14) | Belarus(294.95) | France(332.30) | Russian Federation(368.82) |
| **1990-2019 increase times** | | | | | | | |
| **Incidence (cases)** | | | | | | | |
|  | both | Bahrain(10.95) | United Arab Emirates(15.93) | Qatar(18.13) | Lithuania(1.17) | Ukraine(1.20) | Belarus(1.20) |
|  | female | Bahrain(9.52) | United Arab Emirates(12.43) | Qatar(15.59) | Lithuania(1.10) | Belarus(1.12) | Ukraine(1.14) |
|  | male | Bahrain(11.77) | United Arab Emirates(17.25) | Qatar(19.01) | Japan(1.20) | Lithuania(1.24) | Ukraine(1.27) |
| **Death (cases)** |  |  |  |  |  |  |  |
|  | both | Bahrain(7.32) | Uzbekistan(7.38) | Guatemala(11.66) | Singapore(0.48) | Belarus(0.66) | United Kingdom(0.82) |
|  | female | Sri Lanka(7.18) | Uzbekistan(7.36) | Guatemala(12.57) | Singapore(0.40) | Belarus(0.59) | Finland(0.72) |
|  | male | Bahrain(7.63) | United Arab Emirates(9.33) | Guatemala(10.54) | Singapore(0.61) | Belarus(0.80) | Japan(0.90) |
| **DALY (cases)** |  |  |  |  |  |  |  |
|  | both | Bahrain(8.59) | Qatar(11.12) | United Arab Emirates(11.42) | Belarus(1.10) | Ethiopia(1.15) | Ukraine(1.20) |
|  | female | Bahrain(7.42) | Qatar(8.13) | Guatemala(9.21) | Belarus(1.02) | Netherlands(1.11) | Ukraine(1.11) |
|  | male | Bahrain(9.43) | Qatar(12.68) | United Arab Emirates(13.69) | Ethiopia(1.11) | Belarus(1.22) | Bulgaria(1.25) |
| **EAPC** | | | | | | | |
| **Incidence** |  |  |  |  |  |  |  |
|  | both | Ireland(3.54) | Uzbekistan(3.63) | Luxembourg(4.51) | Ethiopia(-0.79) | Cyprus(-0.39) | Singapore(-0.20) |
|  | female | Ireland(3.33) | Uzbekistan(3.76) | Luxembourg(4.11) | Ethiopia(-0.63) | Cyprus(-0.54) | Singapore(-0.48) |
|  | male | Ireland(3.71) | Uruguay(3.90) | Luxembourg(4.82) | Ethiopia(-0.87) | Cyprus(-0.18) | Japan(-0.03) |
| **Death** |  |  |  |  |  |  |  |
|  | both | Bosnia and Herzegovina(4.36) | Uzbekistan(4.73) | Mauritius(5.09) | Singapore(-7.24) | Japan(-3.84) | Belarus(-3.74) |
|  | female | Lesotho(4.12) | Mauritius(4.66) | Uzbekistan(4.97) | Singapore(-7.92) | Japan(-4.55) | Slovenia(-4.48) |
|  | male | Uzbekistan(4.51) | Bosnia and Herzegovina(5.42) | Mauritius(5.52) | Singapore(-6.41) | Japan(-3.39) | Malaysia(-3.33) |
| **DALY** |  |  |  |  |  |  |  |
|  | both | Lesotho(3.42) | Uzbekistan(4.02) | Mauritius(4.04) | Cyprus(-2.18) | Ethiopia(-2.16) | Singapore(-1.85) |
|  | female | Mauritius(3.59) | Lesotho(3.89) | Uzbekistan(4.26) | Cyprus(-2.75) | Singapore(-2.43) | Ethiopia(-2.14) |
|  | male | Uzbekistan(3.81) | Bosnia and Herzegovina(3.83) | Mauritius(4.45) | Ethiopia(-2.15) | Rwanda(-1.81) | Cyprus(-1.62) |

**Supplementary Table 2.** The top three and the bottom three regions of type 2 diabetes incidence, death, or DALY.

| **Measure** | **sex** | **Top three regions** | | | **Bottom three regions** | | |
| --- | --- | --- | --- | --- | --- | --- | --- |
| **2019 ASIR (per 100,000 people)** | | | | | | | |
| **ASIR** | | | | | | | |
|  | both | Caribbean(366.65) | Central Latin America(418.88) | Oceania(506.06) | Eastern Europe(142.59) | Eastern Sub-Saharan Africa(176.12) | Australasia(190.54) |
|  | female | Caribbean(358.42) | Central Latin America(399.43) | Oceania(454.82) | Eastern Europe(138.83) | Eastern Sub-Saharan Africa(153.34) | High-income Asia Pacific(159.46) |
|  | male | High-income North America(389.17) | Central Latin America(440.21) | Oceania(554.09) | Eastern Europe(146.56) | Eastern Sub-Saharan Africa(200.48) | Western sub-Saharan Africa(201.63) |
| **ASDR** |  |  |  |  |  |  |  |
|  | both | Central Latin America(44.63) | Southern sub-Saharan Africa(68.50) | Oceania(121.02) | High-income Asia Pacific(4.22) | Eastern Europe(6.11) | Western Europe(8.55) |
|  | female | Central Latin America(42.10) | Southern sub-Saharan Africa(67.28) | Oceania(101.60) | High-income Asia Pacific(3.26) | Eastern Europe(6.43) | Australasia(6.92) |
|  | male | Central sub-Saharan Africa(54.10) | Southern sub-Saharan Africa(69.34) | Oceania(140.87) | High-income Asia Pacific(5.24) | Eastern Europe(5.38) | Western Europe(9.87) |
| **Age-standardized DALY rate** |  |  |  |  |  |  |  |
|  | both | Central Latin America(1746.50) | Southern sub-Saharan Africa(1877.98) | Oceania(3703.43) | Eastern Europe(375.97) | High-income Asia Pacific(383.16) | Australasia(419.59) |
|  | female | Central Latin America(1619.12) | Southern sub-Saharan Africa(1849.51) | Oceania(3170.56) | High-income Asia Pacific(304.05) | Australasia(354.43) | Eastern Europe(374.52) |
|  | male | Central Latin America(1890.17) | Southern sub-Saharan Africa(1904.06) | Oceania(4213.08) | Eastern Europe(372.29) | High-income Asia Pacific(466.27) | Australasia(491.63) |
| **1990-2019 increase times** |  |  |  |  |  |  |  |
| **Incidence (cases)** | | | | | | | |
|  | both | Oceania(3.58) | Andean Latin America(3.82) | North Africa and Middle East(4.55) | Eastern Europe(1.45) | High-income Asia Pacific(1.56) | Central Europe(1.68) |
|  | female | Andean Latin America(3.74) | Oceania(3.77) | North Africa and Middle East(4.35) | Eastern Europe(1.41) | High-income Asia Pacific(1.56) | Central Europe(1.56) |
|  | male | Central Asia(3.48) | Andean Latin America(3.92) | North Africa and Middle East(4.74) | Eastern Europe(1.51) | High-income Asia Pacific(1.55) | Central Europe(1.79) |
| **Death (cases)** |  |  |  |  |  |  |  |
|  | both | Andean Latin America(3.50) | South Asia(3.55) | Central Asia(4.02) | Western Europe(1.19) | High-income Asia Pacific(1.43) | Central Europe(1.58) |
|  | female | Oceania(3.46) | Central Asia(3.75) | South Asia(3.95) | Western Europe(1.02) | High-income North America(1.38) | High-income Asia Pacific(1.42) |
|  | male | Central Latin America(3.41) | Andean Latin America(3.71) | Central Asia(4.40) | High-income Asia Pacific(1.45) | Western Europe(1.49) | Central Europe(1.78) |
| **DALY (cases)** |  |  |  |  |  |  |  |
|  | both | South Asia(3.53) | Central Asia(3.58) | Andean Latin America(3.58) | Eastern Europe(1.61) | Western Europe(1.63) | Central Europe(1.73) |
|  | female | Andean Latin America(3.43) | Oceania(3.60) | South Asia(3.77) | Western Europe(1.40) | Eastern Europe(1.54) | Central Europe(1.57) |
|  | male | North Africa and Middle East(3.65) | Andean Latin America(3.77) | Central Asia(3.90) | Eastern Europe(1.73) | High-income Asia Pacific(1.86) | Central Europe(1.91) |
| **EAPC** | | | | | | | |
| **Incidence** |  |  |  |  |  |  |  |
|  | both | North Africa and Middle East(1.83) | High-income North America(2.50) | Central Asia(2.53) | High-income Asia Pacific(0.30) | Eastern Sub-Saharan Africa(0.46) | Central Latin America(0.49) |
|  | female | North Africa and Middle East(1.82) | High-income North America(2.36) | Central Asia(2.45) | Central Latin America(0.17) | High-income Asia Pacific(0.20) | Eastern Sub-Saharan Africa(0.33) |
|  | male | North Africa and Middle East(1.84) | Central Asia(2.62) | High-income North America(2.62) | High-income Asia Pacific(0.35) | Eastern Sub-Saharan Africa(0.60) | Tropical Latin America(0.75) |
| **Death** |  |  |  |  |  |  |  |
|  | both | Southern sub-Saharan Africa(2.02) | South Asia(2.12) | Central Asia(3.03) | High-income Asia Pacific(-2.41) | Western Europe(-1.71) | Australasia(-1.16) |
|  | female | Southern sub-Saharan Africa(2.00) | South Asia(2.29) | Central Asia(2.94) | High-income Asia Pacific(-2.90) | Western Europe(-2.26) | High-income North America(-1.52) |
|  | male | South Asia(1.99) | Southern sub-Saharan Africa(2.03) | Central Asia(3.17) | High-income Asia Pacific(-2.10) | Australasia(-1.12) | Western Europe(-1.09) |
| **DALY** |  |  |  |  |  |  |  |
|  | both | South Asia(1.56) | Southern sub-Saharan Africa(1.79) | Central Asia(2.62) | Eastern Sub-Saharan Africa(-0.41) | High-income Asia Pacific(-0.35) | Tropical Latin America(-0.13) |
|  | female | South Asia(1.57) | Southern sub-Saharan Africa(1.76) | Central Asia(2.56) | Eastern Sub-Saharan Africa(-0.52) | High-income Asia Pacific(-0.51) | Tropical Latin America(-0.51) |
|  | male | South Asia(1.57) | Southern sub-Saharan Africa(1.82) | Central Asia(2.73) | High-income Asia Pacific(-0.29) | Eastern Sub-Saharan Africa(-0.27) | Central sub-Saharan Africa(-0.10) |

**Supplementary Table 3.** The Incident cases and age-standardized Incidence rate of type 2 diabetes in 1990 and 2019, and its temporal trends from 1990 to 2019.

| **Nation** | **Sex** | **Incident Cases No. (95% UI)** | | **Change in absolute number (%)** | **ASIR per 100,000 No.(95% UI)** | | **1990-2019 EAPC No. (95%CI)** |
| --- | --- | --- | --- | --- | --- | --- | --- |
|  |  | **1990** | **2019** |  | **1990** | **2019** |  |
| Afghanistan | both | 18397.72(16505.94,20383.51) | 96530.70(85594.99,109547.89) | 4.25 | 226.88(204.75,251.76) | 412.01(371.66,458.70) | 2.17(2.13,2.21) |
| Albania | both | 2769.44(2479.55,3068.62) | 5820.43(5190.89,6508.20) | 1.10 | 106.83(96.09,117.50) | 161.15(145.29,178.98) | 1.25(1.19,1.30) |
| Algeria | both | 33339.77(30192.10,36817.37) | 159510.25(144387.79,177237.03) | 3.78 | 211.89(191.35,234.05) | 386.38(350.31,428.79) | 2.20(2.12,2.27) |
| American Samoa | both | 197.60(181.80,215.75) | 444.16(414.29,479.71) | 1.25 | 541.12(501.30,586.69) | 819.43(762.75,882.16) | 1.41(1.24,1.58) |
| Andorra | both | 76.96(68.89,85.72) | 266.28(236.57,302.95) | 2.46 | 126.55(113.49,140.97) | 212.86(190.68,239.85) | 1.80(1.78,1.82) |
| Angola | both | 11851.12(10734.75,13059.69) | 44938.74(40695.39,49617.80) | 2.79 | 207.59(191.16,225.80) | 253.23(230.59,276.44) | 0.78(0.73,0.84) |
| Antigua and Barbuda | both | 163.00(152.75,175.08) | 469.55(429.27,514.97) | 1.88 | 313.99(292.29,338.96) | 430.82(396.76,468.32) | 1.05(1.00,1.10) |
| Argentina | both | 56117.99(51738.48,60548.75) | 130475.36(118349.72,142445.81) | 1.33 | 172.35(159.20,185.96) | 262.98(238.07,287.53) | 1.22(1.10,1.34) |
| Armenia | both | 5023.08(4588.98,5487.83) | 9986.36(9144.46,11103.86) | 0.99 | 156.94(144.56,169.94) | 265.75(244.34,290.17) | 1.81(1.57,2.05) |
| Australia | both | 24800.56(22709.23,26869.59) | 65363.60(58193.83,72209.44) | 1.64 | 127.71(117.16,138.35) | 193.74(174.11,214.62) | 1.39(1.15,1.64) |
| Austria | both | 12232.17(11115.43,13402.89) | 28550.77(25256.93,31460.79) | 1.33 | 122.47(111.29,133.95) | 215.32(191.56,236.79) | 2.02(1.89,2.14) |
| Azerbaijan | both | 8100.14(7332.34,8821.84) | 30691.81(27620.89,33891.26) | 2.79 | 130.57(119.34,141.48) | 254.84(231.00,280.01) | 2.45(2.32,2.59) |
| Bahrain | both | 1313.16(1191.69,1434.10) | 14375.57(13280.92,15587.77) | 9.95 | 442.46(409.23,476.74) | 757.62(721.30,795.85) | 1.87(1.65,2.10) |
| Bangladesh | both | 107761.26(99213.01,116932.55) | 336852.12(312028.90,364331.35) | 2.13 | 166.27(154.03,179.23) | 223.64(207.62,241.34) | 1.05(0.88,1.22) |
| Barbados | both | 832.10(789.89,873.99) | 1660.49(1531.87,1802.05) | 1.00 | 319.00(301.15,339.21) | 397.94(368.85,427.85) | 0.66(0.62,0.69) |
| Belarus | both | 13426.48(12089.80,14914.71) | 16103.24(14439.61,17949.10) | 0.20 | 110.86(100.31,121.94) | 126.90(113.76,140.16) | 0.36(0.19,0.52) |
| Belgium | both | 19156.24(17305.11,21185.05) | 35423.48(31613.54,39402.57) | 0.85 | 146.25(132.01,161.37) | 220.94(198.09,243.45) | 1.33(1.30,1.36) |
| Belize | both | 234.74(217.61,252.27) | 1187.75(1088.44,1284.55) | 4.06 | 210.92(195.72,227.50) | 333.36(306.63,359.95) | 1.53(1.39,1.66) |
| Benin | both | 3689.40(3351.84,4041.94) | 15789.65(14400.06,17356.57) | 3.28 | 155.87(142.06,170.57) | 239.14(217.87,262.95) | 1.48(1.41,1.55) |
| Bermuda | both | 130.63(120.11,142.23) | 257.88(233.51,284.82) | 0.97 | 198.66(182.34,215.97) | 255.83(233.73,280.68) | 0.72(0.66,0.78) |
| Bhutan | both | 575.21(519.47,636.47) | 1746.89(1597.49,1918.91) | 2.04 | 152.47(138.77,167.68) | 246.80(226.67,270.08) | 1.77(1.72,1.83) |
| Bolivia (Plurinational State of) | both | 7524.42(6830.42,8307.57) | 26959.64(24553.83,29611.07) | 2.58 | 191.66(173.61,211.47) | 265.30(241.43,292.03) | 1.10(1.04,1.16) |
| Bosnia and Herzegovina | both | 9187.77(8287.52,10167.82) | 18868.20(17063.12,20683.46) | 1.05 | 189.29(171.58,208.07) | 376.29(343.11,411.96) | 2.76(2.57,2.96) |
| Botswana | both | 1536.91(1401.53,1673.96) | 6134.63(5643.87,6696.30) | 2.99 | 218.41(200.61,237.78) | 338.31(313.53,366.87) | 1.81(1.71,1.91) |
| Brazil | both | 271175.53(250783.85,290968.38) | 659934.64(606399.98,721235.45) | 1.43 | 243.71(225.57,262.99) | 268.27(247.06,292.61) | 0.54(0.42,0.67) |
| Brunei Darussalam | both | 918.55(860.94,987.44) | 3048.36(2845.41,3265.88) | 2.32 | 577.25(544.61,611.19) | 639.58(600.74,678.81) | 0.36(0.17,0.55) |
| Bulgaria | both | 20814.68(18736.76,22866.42) | 25671.63(23203.23,28646.77) | 0.23 | 181.77(165.00,197.48) | 253.87(231.15,281.40) | 1.19(1.07,1.31) |
| Burkina Faso | both | 8515.46(7728.16,9328.15) | 27297.92(24789.06,29730.77) | 2.21 | 165.92(152.00,181.17) | 220.98(202.48,242.09) | 1.02(1.00,1.05) |
| Burundi | both | 4757.91(4331.36,5192.29) | 11772.93(10668.34,13002.56) | 1.47 | 159.25(145.98,174.13) | 178.57(162.84,195.34) | 0.34(0.32,0.36) |
| Cambodia | both | 8650.28(7971.06,9418.20) | 43589.68(39773.07,47961.27) | 4.04 | 151.34(139.87,164.71) | 303.03(275.88,333.38) | 2.45(2.26,2.64) |
| Cameroon | both | 8047.09(7322.20,8756.12) | 37666.63(34505.42,41242.91) | 3.68 | 150.22(138.18,163.06) | 234.88(215.65,255.71) | 1.45(1.07,1.84) |
| Canada | both | 35507.87(33275.66,37642.59) | 98931.31(86594.83,110860.25) | 1.79 | 112.42(105.19,119.71) | 182.48(160.58,204.32) | 1.39(1.31,1.47) |
| Cabo Verde | both | 349.40(316.73,384.50) | 1373.39(1241.22,1513.11) | 2.93 | 152.25(138.29,168.43) | 280.89(253.59,309.24) | 2.22(2.01,2.43) |
| Central African Republic | both | 3406.56(3086.03,3742.26) | 9729.26(8828.87,10737.63) | 1.86 | 206.49(189.70,225.04) | 276.68(253.98,301.99) | 1.11(1.05,1.16) |
| Chad | both | 4900.66(4456.05,5370.92) | 17021.76(15372.52,18661.82) | 2.47 | 151.85(138.25,167.61) | 223.36(201.20,246.03) | 1.34(1.18,1.51) |
| Chile | both | 21572.60(19666.35,23492.82) | 71717.16(64824.38,80238.91) | 2.32 | 191.14(174.03,208.28) | 311.62(283.50,345.59) | 1.80(1.63,1.98) |
| China | both | 1902459.20(1729904.71,2095091.84) | 3739121.08(3440391.93,4096656.94) | 0.97 | 174.27(158.91,191.69) | 201.06(185.71,219.17) | 0.61(0.41,0.82) |
| Colombia | both | 66264.67(59692.65,72801.64) | 171273.38(156813.19,188371.96) | 1.58 | 296.36(266.18,325.29) | 325.85(298.82,356.62) | 0.06(-0.08,0.20) |
| Comoros | both | 386.11(355.67,415.76) | 947.83(870.55,1034.61) | 1.45 | 146.44(135.70,158.96) | 165.96(152.93,180.57) | 0.43(0.41,0.45) |
| Congo | both | 3190.21(2896.57,3490.23) | 11055.84(10012.11,12245.15) | 2.47 | 220.82(202.63,241.18) | 282.89(259.54,310.49) | 0.88(0.85,0.91) |
| Costa Rica | both | 5584.33(5071.73,6109.68) | 18348.86(16350.01,20395.85) | 2.29 | 270.02(244.13,296.68) | 348.37(311.35,385.81) | 0.71(0.62,0.81) |
| C么te d'Ivoire | both | 9013.95(8183.00,9924.26) | 36376.20(33128.25,39750.07) | 3.04 | 161.83(149.17,176.47) | 241.05(222.23,262.78) | 1.37(1.24,1.49) |
| Croatia | both | 12983.58(11760.85,14248.03) | 18661.31(16815.46,20602.65) | 0.44 | 206.90(187.92,225.15) | 295.89(267.26,327.28) | 1.12(1.06,1.18) |
| Cuba | both | 29979.80(27519.14,32223.29) | 53720.71(48313.42,59137.69) | 0.79 | 276.82(253.75,298.92) | 337.18(305.86,370.69) | 0.63(0.47,0.78) |
| Cyprus | both | 2717.33(2525.80,2911.88) | 5969.97(5478.73,6499.51) | 1.20 | 326.04(301.52,352.89) | 337.29(308.83,368.20) | -0.39(-0.58,-0.20) |
| Czechia | both | 31697.02(28909.77,35058.85) | 67426.87(61137.13,73482.23) | 1.13 | 254.07(231.25,280.59) | 445.30(404.62,485.86) | 1.88(1.67,2.08) |
| Democratic Republic of the Congo | both | 41261.10(37486.00,45465.17) | 137513.91(124163.90,152047.93) | 2.33 | 194.07(178.26,212.99) | 254.34(231.96,279.44) | 0.96(0.92,0.99) |
| Denmark | both | 6762.65(6111.60,7362.30) | 15885.21(14445.80,17528.39) | 1.35 | 102.78(92.84,112.50) | 193.21(176.05,211.08) | 2.17(2.06,2.27) |
| Djibouti | both | 327.94(297.27,365.01) | 1667.65(1513.49,1843.67) | 4.09 | 150.72(138.53,163.91) | 193.78(177.13,211.62) | 0.89(0.84,0.94) |
| Dominica | both | 211.48(198.16,226.39) | 403.72(371.56,438.60) | 0.91 | 327.73(305.91,352.33) | 496.07(458.52,537.15) | 1.39(1.28,1.50) |
| Dominican Republic | both | 6404.98(5921.25,6941.83) | 23281.63(21457.67,25422.83) | 2.63 | 132.22(122.24,143.10) | 223.74(206.66,244.50) | 2.06(1.92,2.20) |
| Ecuador | both | 11707.71(10746.89,12619.78) | 49903.78(45805.11,53961.08) | 3.26 | 183.29(168.88,197.42) | 305.67(280.96,331.45) | 1.91(1.71,2.11) |
| Egypt | both | 53140.44(48970.17,57588.46) | 251706.51(229320.14,278450.43) | 3.74 | 140.81(130.44,151.68) | 295.24(268.25,325.18) | 2.74(2.62,2.86) |
| El Salvador | both | 7274.02(6605.27,7882.00) | 21952.39(20168.85,23790.80) | 2.02 | 205.96(186.97,224.09) | 366.85(336.08,397.97) | 2.03(1.90,2.15) |
| Equatorial Guinea | both | 522.79(476.50,576.67) | 2284.93(2057.31,2523.51) | 3.37 | 205.25(188.47,224.86) | 283.08(258.91,310.50) | 1.28(1.23,1.33) |
| Eritrea | both | 2310.14(2094.14,2551.52) | 8165.19(7415.38,9003.76) | 2.53 | 153.93(141.91,167.99) | 195.24(178.96,213.56) | 0.87(0.84,0.91) |
| Estonia | both | 2265.45(2057.54,2486.15) | 3092.63(2781.01,3425.28) | 0.37 | 122.39(111.24,134.30) | 179.11(162.50,197.36) | 1.42(1.15,1.69) |
| Ethiopia | both | 46033.80(42126.80,50367.68) | 86011.80(77830.69,94228.90) | 0.87 | 170.71(157.68,185.61) | 148.03(135.68,161.45) | -0.79(-0.89,-0.68) |
| Micronesia (Federated States of) | both | 203.36(187.92,219.71) | 557.84(515.95,603.91) | 1.74 | 304.31(283.28,327.29) | 580.29(540.53,623.06) | 2.32(1.98,2.67) |
| Fiji | both | 2816.25(2618.18,3040.47) | 7362.72(6995.00,7761.28) | 1.61 | 529.21(499.14,561.17) | 797.04(763.50,835.80) | 1.29(1.10,1.48) |
| Finland | both | 12253.67(11238.75,13380.97) | 22191.26(19926.24,24461.59) | 0.81 | 193.63(177.84,209.38) | 287.80(260.72,315.64) | 1.50(1.40,1.60) |
| France | both | 65978.51(59874.82,71104.35) | 131459.17(118732.12,144826.26) | 0.99 | 93.26(85.30,100.71) | 140.55(127.36,154.56) | 1.44(1.34,1.54) |
| Gabon | both | 1548.67(1415.37,1689.08) | 4482.76(4107.35,4919.28) | 1.89 | 237.36(217.78,258.81) | 320.22(294.15,348.27) | 1.11(0.99,1.23) |
| Georgia | both | 8522.70(7755.54,9387.14) | 13888.84(12694.23,15123.85) | 0.63 | 139.90(127.35,152.24) | 305.48(279.85,330.19) | 2.04(0.52,3.58) |
| Germany | both | 264252.46(239358.50,294405.44) | 432005.05(386586.37,473253.15) | 0.63 | 242.26(219.32,269.84) | 340.97(306.09,372.52) | 0.93(0.70,1.16) |
| Ghana | both | 13147.75(11927.69,14574.96) | 55316.73(50510.19,60885.45) | 3.21 | 164.97(151.40,181.84) | 260.66(238.67,285.56) | 2.06(1.72,2.39) |
| Greece | both | 19803.79(17848.57,21660.50) | 33519.89(29558.54,36956.65) | 0.69 | 144.49(130.62,158.13) | 227.35(201.88,251.17) | 1.59(1.50,1.69) |
| Greenland | both | 47.95(43.30,53.37) | 152.23(135.61,171.76) | 2.17 | 100.61(91.78,110.46) | 200.43(180.34,222.96) | 2.37(2.23,2.51) |
| Grenada | both | 216.82(203.05,231.99) | 577.19(532.15,626.28) | 1.66 | 327.37(305.13,353.03) | 481.18(447.55,520.83) | 1.40(1.35,1.45) |
| Guam | both | 295.59(270.15,326.45) | 631.75(578.09,694.04) | 1.14 | 255.86(236.28,278.55) | 349.01(320.25,383.34) | 1.08(0.98,1.19) |
| Guatemala | both | 10943.97(10074.89,11897.94) | 60917.03(56317.39,65619.43) | 4.57 | 228.59(210.70,249.95) | 437.86(404.61,472.74) | 2.37(2.10,2.65) |
| Guinea | both | 5019.28(4612.53,5467.43) | 14728.30(13519.63,16053.12) | 1.93 | 136.08(125.05,148.24) | 214.25(196.64,233.23) | 1.52(1.41,1.62) |
| Guinea-Bissau | both | 859.10(790.82,936.66) | 2484.23(2259.19,2735.02) | 1.89 | 167.64(155.10,183.06) | 235.41(215.41,257.70) | 1.16(1.10,1.21) |
| Guyana | both | 1978.37(1835.12,2133.54) | 4073.83(3775.73,4381.51) | 1.06 | 381.62(356.34,408.87) | 533.58(495.70,571.16) | 1.09(0.97,1.21) |
| Haiti | both | 13102.08(11962.89,14346.63) | 42456.22(38466.98,46653.55) | 2.24 | 303.33(277.96,330.52) | 429.87(392.78,470.16) | 1.18(1.16,1.20) |
| Honduras | both | 7472.61(6756.73,8245.32) | 30318.05(27424.57,33556.36) | 3.06 | 274.42(247.41,303.15) | 380.91(343.37,421.06) | 1.09(1.03,1.14) |
| Hungary | both | 26088.95(23502.05,28747.80) | 41730.79(37921.20,45775.85) | 0.60 | 198.56(179.96,218.39) | 298.35(270.87,326.41) | 1.42(1.17,1.66) |
| Iceland | both | 309.75(280.80,345.16) | 972.50(875.91,1083.89) | 2.14 | 118.29(106.74,132.31) | 219.96(197.74,243.15) | 2.15(2.13,2.16) |
| India | both | 1227211.51(1106448.04,1366380.99) | 4206580.10(3821838.07,4647331.08) | 2.43 | 193.09(175.30,213.29) | 309.20(281.51,340.93) | 1.39(1.31,1.47) |
| Indonesia | both | 204200.19(186765.01,224002.64) | 631701.22(579046.31,692115.22) | 2.09 | 156.92(143.87,171.77) | 236.88(217.73,257.70) | 1.46(1.40,1.52) |
| Iran (Islamic Republic of) | both | 61449.26(55483.84,68015.96) | 291482.36(265076.85,320392.56) | 3.74 | 170.51(154.95,188.36) | 323.31(295.73,354.45) | 2.50(2.38,2.62) |
| Iraq | both | 30355.33(27797.54,33219.51) | 145023.94(133029.78,156843.13) | 3.78 | 286.87(262.83,312.74) | 424.91(392.39,460.20) | 1.34(1.29,1.39) |
| Ireland | both | 3148.98(2869.15,3449.46) | 14149.71(12613.47,15827.48) | 3.49 | 83.59(76.42,91.78) | 221.61(198.45,246.16) | 3.54(3.43,3.65) |
| Israel | both | 7666.45(7028.03,8301.69) | 25822.02(23597.64,28017.82) | 2.37 | 169.26(154.54,183.81) | 258.62(235.74,281.12) | 1.59(1.18,2.00) |
| Italy | both | 162282.66(147514.94,178504.37) | 271054.87(243690.56,299154.42) | 0.67 | 212.97(193.31,232.80) | 289.31(258.82,318.89) | 1.29(0.82,1.77) |
| Jamaica | both | 5046.89(4757.20,5356.27) | 13416.24(12367.62,14450.25) | 1.66 | 282.00(264.85,300.33) | 446.09(411.83,481.10) | 1.52(1.37,1.68) |
| Japan | both | 253640.23(230438.74,278890.14) | 308827.73(278939.30,341503.58) | 0.22 | 156.25(142.30,171.34) | 168.41(152.53,186.33) | -0.15(-0.35,0.06) |
| Jordan | both | 5950.36(5503.15,6444.36) | 38420.98(35067.33,41788.03) | 5.46 | 300.40(277.93,323.26) | 395.59(363.36,428.41) | 0.72(0.54,0.90) |
| Kazakhstan | both | 24178.13(22044.82,26465.67) | 57268.15(52123.62,63133.70) | 1.37 | 161.66(147.60,177.08) | 287.69(263.00,315.38) | 2.20(2.11,2.30) |
| Kenya | both | 14020.36(12726.12,15438.10) | 51889.76(47004.06,57093.11) | 2.70 | 130.05(119.65,142.13) | 161.74(148.02,176.76) | 0.76(0.71,0.80) |
| Kiribati | both | 221.67(205.19,238.93) | 640.68(591.01,695.94) | 1.89 | 389.63(365.12,417.42) | 610.54(568.73,655.39) | 1.61(1.44,1.78) |
| Kuwait | both | 4253.42(3832.05,4697.85) | 24116.96(21491.57,27092.39) | 4.67 | 346.89(317.74,378.89) | 495.49(451.90,542.72) | 0.99(0.75,1.22) |
| Kyrgyzstan | both | 3614.20(3272.49,3969.86) | 9209.55(8248.68,10139.59) | 1.55 | 102.97(93.73,113.05) | 151.50(137.01,166.17) | 1.30(1.26,1.34) |
| Lao People's Democratic Republic | both | 5617.53(5096.07,6164.32) | 19522.04(17770.14,21390.62) | 2.48 | 215.78(197.62,237.26) | 340.57(310.47,372.38) | 1.56(1.54,1.58) |
| Latvia | both | 3591.59(3260.26,3925.70) | 4641.63(4191.73,5124.17) | 0.29 | 113.82(103.55,124.47) | 181.39(164.91,198.66) | 1.84(1.67,2.01) |
| Lebanon | both | 6091.86(5580.59,6711.86) | 20236.57(18365.17,22297.03) | 2.32 | 231.30(211.83,255.18) | 383.50(347.67,424.07) | 1.77(1.72,1.82) |
| Lesotho | both | 2219.29(2037.46,2434.58) | 4802.81(4426.48,5223.50) | 1.16 | 200.15(185.07,217.99) | 300.61(278.69,325.71) | 1.74(1.63,1.85) |
| Liberia | both | 2338.56(2127.42,2559.48) | 7914.46(7133.81,8815.50) | 2.38 | 188.88(171.60,207.18) | 272.79(245.28,302.14) | 1.49(1.38,1.60) |
| Libya | both | 6199.65(5615.33,6843.68) | 33010.15(29472.36,37015.58) | 4.32 | 244.75(221.33,271.05) | 454.79(409.66,506.50) | 2.22(2.12,2.32) |
| Lithuania | both | 4537.80(4137.89,4980.86) | 5286.85(4787.01,5852.77) | 0.17 | 108.19(98.94,118.36) | 141.66(129.26,155.45) | 1.05(0.88,1.21) |
| Luxembourg | both | 506.58(466.52,552.61) | 2933.50(2649.73,3241.95) | 4.79 | 102.57(94.29,112.21) | 339.15(307.85,372.70) | 4.51(4.37,4.65) |
| North Macedonia | both | 4710.80(4250.08,5163.07) | 11682.88(10557.69,12854.84) | 1.48 | 226.18(204.75,246.57) | 384.21(349.38,420.79) | 1.91(1.75,2.08) |
| Madagascar | both | 9024.09(8201.45,9868.33) | 27755.83(25157.76,30627.05) | 2.08 | 138.43(127.56,150.38) | 168.92(153.99,184.21) | 0.71(0.69,0.73) |
| Malawi | both | 8778.88(7963.50,9680.48) | 22635.36(20582.76,24845.02) | 1.58 | 175.10(160.49,191.50) | 216.58(198.49,237.05) | 0.78(0.65,0.92) |
| Malaysia | both | 29352.71(27401.83,31377.66) | 104178.37(93573.93,114357.55) | 2.55 | 241.03(225.26,257.25) | 334.02(300.79,365.38) | 1.20(1.09,1.30) |
| Maldives | both | 232.27(211.07,254.88) | 1154.06(1042.78,1279.23) | 3.97 | 200.81(183.45,220.42) | 271.15(243.98,297.35) | 0.93(0.81,1.06) |
| Mali | both | 6980.66(6317.11,7655.55) | 23595.35(21472.15,25919.88) | 2.38 | 143.38(130.97,157.26) | 208.96(190.88,229.07) | 1.32(1.31,1.34) |
| Malta | both | 981.98(893.47,1076.68) | 2062.14(1839.39,2283.87) | 1.10 | 233.83(212.79,257.14) | 314.06(283.85,345.61) | 1.02(0.96,1.08) |
| Marshall Islands | both | 142.62(129.07,157.07) | 441.00(405.71,482.68) | 2.09 | 499.79(458.93,542.80) | 795.18(735.96,858.57) | 1.62(1.50,1.73) |
| Mauritania | both | 1444.33(1326.96,1571.82) | 4163.06(3802.97,4558.08) | 1.88 | 126.02(116.46,137.52) | 168.02(154.15,183.63) | 0.67(0.53,0.81) |
| Mauritius | both | 2795.22(2608.85,2987.53) | 10356.42(9476.63,11157.18) | 2.71 | 304.98(283.37,327.30) | 591.43(547.96,632.70) | 2.80(2.51,3.09) |
| Mexico | both | 240621.91(223507.44,258050.45) | 620193.70(572870.12,663975.29) | 1.58 | 438.90(406.04,471.82) | 477.63(442.19,511.45) | 0.27(0.11,0.43) |
| Republic of Moldova | both | 7076.05(6452.80,7807.70) | 9630.03(8763.47,10608.74) | 0.36 | 152.34(139.23,166.74) | 197.43(180.60,216.60) | 1.00(0.83,1.17) |
| Mongolia | both | 993.72(883.90,1107.75) | 3525.93(3149.28,3972.04) | 2.55 | 68.83(61.98,76.86) | 104.83(94.17,116.66) | 1.81(1.67,1.94) |
| Montenegro | both | 1463.03(1326.42,1612.84) | 2842.90(2580.43,3126.76) | 0.94 | 222.32(202.00,244.23) | 335.34(304.94,365.55) | 1.34(1.29,1.39) |
| Morocco | both | 31322.23(28198.31,34673.53) | 129305.04(116580.83,143166.16) | 3.13 | 178.73(161.37,198.14) | 345.08(311.46,378.66) | 2.41(2.37,2.46) |
| Mozambique | both | 11356.99(10425.60,12400.30) | 33745.79(30843.49,36901.06) | 1.97 | 154.44(142.80,168.27) | 210.50(194.00,228.19) | 1.19(1.14,1.24) |
| Myanmar | both | 68867.30(63625.13,74693.31) | 180723.00(168115.76,195078.53) | 1.62 | 240.94(222.64,261.24) | 341.32(318.31,368.88) | 1.22(1.08,1.35) |
| Namibia | both | 1907.70(1753.70,2071.13) | 4565.50(4177.68,4979.37) | 1.39 | 230.57(213.49,250.51) | 266.09(244.46,289.71) | 0.48(0.38,0.58) |
| Nepal | both | 18800.64(17015.64,20759.06) | 67947.43(61563.21,74869.25) | 2.61 | 145.86(133.25,160.67) | 250.46(226.77,276.51) | 1.75(1.64,1.85) |
| Netherlands | both | 27570.43(25424.53,29580.23) | 45827.76(41474.39,50473.58) | 0.66 | 152.70(139.67,164.37) | 182.06(163.76,200.15) | 0.47(0.42,0.51) |
| New Zealand | both | 4341.89(3932.30,4752.02) | 10817.35(9897.49,11715.01) | 1.49 | 115.73(105.30,127.13) | 173.84(159.39,188.20) | 1.00(0.68,1.32) |
| Nicaragua | both | 5812.47(5324.43,6356.83) | 21685.83(19849.76,23471.88) | 2.73 | 280.54(258.25,305.93) | 377.88(344.47,408.78) | 1.04(0.98,1.11) |
| Niger | both | 3177.84(2885.87,3482.61) | 16543.97(14914.20,18235.24) | 4.21 | 94.61(87.39,103.20) | 159.06(144.46,174.85) | 1.75(1.58,1.93) |
| Nigeria | both | 70477.53(64273.39,77042.39) | 187357.40(169703.84,206780.38) | 1.66 | 137.46(126.18,150.29) | 163.70(149.82,178.78) | 0.34(0.22,0.46) |
| Democratic People's Republic of Korea | both | 26561.93(24073.06,29506.54) | 63541.06(56979.91,70986.64) | 1.39 | 134.07(121.90,148.64) | 198.95(179.74,220.27) | 1.36(1.35,1.37) |
| Northern Mariana Islands | both | 114.31(103.28,127.98) | 259.75(235.63,287.32) | 1.27 | 301.20(277.79,328.23) | 468.47(432.02,509.81) | 1.60(1.48,1.72) |
| Norway | both | 9711.50(8842.27,10694.54) | 17962.97(16209.37,19944.93) | 0.85 | 184.48(167.32,204.18) | 246.87(223.73,273.05) | 0.49(0.35,0.63) |
| Oman | both | 2706.76(2449.67,2990.75) | 14921.19(13301.30,16609.09) | 4.51 | 241.99(222.13,264.43) | 410.33(374.42,447.78) | 1.75(1.65,1.85) |
| Pakistan | both | 136426.59(123543.36,150278.10) | 494938.49(446207.05,549456.21) | 2.63 | 185.04(168.45,203.73) | 302.59(275.30,333.27) | 1.99(1.86,2.12) |
| Palestine | both | 2768.74(2536.87,3023.96) | 15518.01(14192.94,16743.86) | 4.60 | 258.18(237.51,281.69) | 452.68(414.55,485.39) | 2.08(1.98,2.17) |
| Panama | both | 4458.57(4070.70,4874.73) | 16087.02(14665.00,17514.35) | 2.61 | 257.39(234.06,282.17) | 380.47(347.14,414.03) | 1.36(1.33,1.39) |
| Papua New Guinea | both | 9394.22(8627.42,10247.15) | 38621.16(35459.31,42256.94) | 3.11 | 316.34(292.76,340.35) | 463.54(430.14,502.71) | 1.37(1.32,1.42) |
| Paraguay | both | 4677.82(4285.55,5082.04) | 17759.65(16331.42,19297.21) | 2.80 | 177.73(162.73,193.40) | 283.64(260.49,307.46) | 1.47(1.32,1.62) |
| Peru | both | 17794.77(16265.77,19315.73) | 64732.28(59013.09,70968.21) | 2.64 | 126.63(115.95,137.49) | 193.60(176.53,213.30) | 1.44(1.35,1.52) |
| Philippines | both | 77478.61(71074.38,84331.24) | 235025.96(217252.20,254475.34) | 2.03 | 205.96(190.01,223.90) | 248.84(229.63,269.54) | 0.47(0.40,0.54) |
| Poland | both | 87566.50(80471.63,96134.64) | 148470.95(134836.59,163673.14) | 0.70 | 206.04(189.08,225.08) | 280.66(256.15,306.31) | 1.24(0.98,1.50) |
| Portugal | both | 27796.69(25044.19,30796.83) | 53819.34(48510.11,59405.98) | 0.94 | 220.47(198.79,242.50) | 333.22(300.16,367.74) | 1.50(1.28,1.72) |
| Puerto Rico | both | 12577.47(11706.99,13584.28) | 22339.92(20615.21,24153.37) | 0.78 | 349.31(323.84,379.12) | 461.36(426.42,498.78) | 0.95(0.78,1.12) |
| Qatar | both | 1319.43(1169.11,1489.19) | 23919.52(21586.08,26587.13) | 17.13 | 493.12(453.74,534.08) | 818.03(773.89,868.70) | 1.74(1.43,2.05) |
| Romania | both | 36232.97(32747.56,40327.26) | 52756.33(47338.94,58413.07) | 0.46 | 134.38(121.66,148.40) | 197.04(176.75,216.76) | 1.27(1.22,1.31) |
| Russian Federation | both | 176510.92(160768.15,194507.91) | 280615.79(256305.35,308403.35) | 0.59 | 101.09(92.40,110.81) | 141.06(129.29,154.25) | 1.21(1.10,1.31) |
| Rwanda | both | 6003.33(5447.18,6563.09) | 13997.56(12679.25,15391.10) | 1.33 | 159.35(146.28,173.03) | 172.39(157.16,189.27) | 0.16(0.10,0.23) |
| Saint Lucia | both | 439.70(411.75,469.23) | 1192.37(1103.97,1289.14) | 1.71 | 456.23(425.06,489.42) | 546.46(507.16,587.16) | 0.57(0.53,0.61) |
| Saint Vincent and the Grenadines | both | 304.62(286.23,325.89) | 675.69(627.28,731.79) | 1.22 | 392.90(368.08,420.99) | 505.89(473.04,545.47) | 0.76(0.71,0.80) |
| Samoa | both | 378.89(351.03,410.42) | 935.19(859.24,1019.50) | 1.47 | 331.84(307.71,358.99) | 513.87(473.37,557.77) | 1.48(1.34,1.62) |
| Sao Tome and Principe | both | 116.11(104.24,128.86) | 324.56(291.45,359.19) | 1.80 | 162.26(146.12,179.64) | 232.74(208.47,257.46) | 1.31(1.18,1.44) |
| Saudi Arabia | both | 27124.18(24772.30,29574.90) | 169200.49(151543.69,189934.35) | 5.24 | 282.68(260.43,306.36) | 462.07(420.77,506.93) | 1.74(1.68,1.79) |
| Senegal | both | 8443.67(7660.64,9314.45) | 28322.31(25602.95,31159.73) | 2.35 | 213.72(193.80,236.71) | 297.24(269.18,329.13) | 1.45(1.23,1.66) |
| Serbia | both | 26032.31(23563.73,28538.66) | 40877.13(36819.32,44718.64) | 0.57 | 222.66(202.96,243.17) | 336.63(303.97,365.01) | 1.41(1.35,1.48) |
| Seychelles | both | 137.27(124.87,150.17) | 615.02(559.93,670.94) | 3.48 | 231.95(210.49,254.03) | 504.42(463.28,544.34) | 2.75(2.59,2.92) |
| Sierra Leone | both | 2035.71(1877.49,2205.17) | 7141.04(6519.04,7794.40) | 2.51 | 95.39(88.37,103.13) | 154.37(142.00,168.86) | 1.84(1.75,1.93) |
| Singapore | both | 8241.92(7642.55,8807.49) | 20354.01(18481.78,22476.79) | 1.47 | 267.89(247.91,286.53) | 258.76(236.32,284.51) | -0.20(-0.37,-0.02) |
| Slovakia | both | 10021.58(9105.31,11028.44) | 17622.80(15946.23,19556.71) | 0.76 | 173.56(157.33,190.82) | 230.07(207.69,253.01) | 0.84(0.80,0.88) |
| Slovenia | both | 4685.16(4287.59,5125.57) | 7101.77(6404.37,7845.00) | 0.52 | 197.10(180.79,214.89) | 232.73(211.05,254.67) | 0.23(0.08,0.38) |
| Solomon Islands | both | 618.23(566.35,676.34) | 2600.27(2403.78,2820.03) | 3.21 | 283.04(261.62,306.12) | 493.91(460.57,528.67) | 2.12(2.06,2.19) |
| Somalia | both | 5641.11(5113.01,6191.50) | 20461.97(18526.43,22721.99) | 2.63 | 159.27(145.95,173.86) | 194.07(178.18,211.07) | 0.68(0.66,0.71) |
| South Africa | both | 54623.78(50481.65,59258.09) | 170174.08(158714.03,182003.25) | 2.12 | 221.61(205.96,239.89) | 329.64(308.84,352.97) | 1.44(1.28,1.60) |
| Republic of Korea | both | 76067.73(70543.96,81875.58) | 195279.28(178361.61,213857.88) | 1.57 | 187.47(174.21,201.66) | 242.40(224.41,263.45) | 0.69(0.56,0.82) |
| South Sudan | both | 4564.51(4158.80,4990.56) | 9800.78(8900.73,10811.32) | 1.15 | 151.15(139.37,164.56) | 183.25(168.45,200.69) | 0.65(0.60,0.70) |
| Spain | both | 115530.70(105768.15,124944.64) | 195916.39(176420.84,216841.06) | 0.70 | 235.06(216.49,252.90) | 288.93(262.26,319.29) | 0.49(0.25,0.73) |
| Sri Lanka | both | 33415.02(30707.35,36013.00) | 136200.94(125501.09,147279.50) | 3.08 | 258.79(237.36,280.42) | 514.72(476.31,555.21) | 2.52(2.41,2.63) |
| Sudan | both | 22705.92(20519.37,25116.96) | 100585.57(90376.42,112084.08) | 3.43 | 186.55(168.72,206.62) | 359.63(323.43,399.64) | 2.29(2.25,2.32) |
| Suriname | both | 947.63(867.36,1031.62) | 3141.72(2897.86,3417.78) | 2.32 | 305.29(280.15,333.12) | 491.46(453.91,533.13) | 1.85(1.77,1.93) |
| Eswatini | both | 944.15(871.01,1028.02) | 2730.59(2521.95,2940.83) | 1.89 | 256.57(237.94,276.92) | 368.09(342.88,396.76) | 1.46(1.25,1.66) |
| Sweden | both | 17097.65(15443.12,18959.01) | 31088.47(27745.15,34534.21) | 0.82 | 148.50(133.95,165.03) | 221.49(197.85,244.58) | 1.54(1.41,1.68) |
| Switzerland | both | 15281.66(13990.43,16699.66) | 28821.03(25659.44,32019.17) | 0.89 | 172.98(158.22,188.99) | 226.79(202.79,252.51) | 0.91(0.85,0.97) |
| Syrian Arab Republic | both | 16250.54(14798.21,17839.20) | 51641.69(46246.60,56782.20) | 2.18 | 224.53(204.77,245.98) | 345.74(312.53,378.21) | 1.36(1.30,1.41) |
| Taiwan (Province of China) | both | 38972.62(36688.08,41429.90) | 91671.49(85930.77,97412.31) | 1.35 | 208.76(196.61,221.34) | 259.23(244.67,273.49) | 0.62(0.49,0.75) |
| Tajikistan | both | 4392.22(4001.26,4789.61) | 21870.43(19958.54,23970.38) | 3.98 | 120.93(110.65,131.31) | 268.25(245.32,293.59) | 3.04(2.94,3.13) |
| United Republic of Tanzania | both | 15188.29(13968.66,16470.03) | 54995.60(50199.69,60409.16) | 2.62 | 115.66(107.96,124.54) | 167.46(153.45,183.02) | 1.61(1.48,1.73) |
| Thailand | both | 81537.11(74759.86,88516.36) | 252286.97(229492.51,279826.01) | 2.09 | 181.65(165.86,198.36) | 256.32(235.36,282.93) | 0.79(0.62,0.95) |
| Bahamas | both | 568.78(523.20,615.91) | 1673.52(1517.83,1828.96) | 1.94 | 287.53(266.15,310.54) | 381.88(348.61,415.37) | 0.88(0.84,0.91) |
| Gambia | both | 632.86(573.22,700.35) | 2664.63(2427.29,2917.06) | 3.21 | 138.67(126.58,153.54) | 213.80(194.59,234.50) | 1.55(1.50,1.60) |
| Timor-Leste | both | 629.76(571.00,696.01) | 2652.98(2394.98,2905.98) | 3.21 | 152.25(138.74,166.76) | 275.66(249.58,301.85) | 1.88(1.74,2.01) |
| Togo | both | 1891.28(1712.41,2071.39) | 8118.61(7412.38,8901.16) | 3.29 | 118.56(108.32,129.39) | 167.33(154.29,182.59) | 1.17(1.10,1.24) |
| Tonga | both | 236.28(219.59,255.06) | 442.48(411.62,475.62) | 0.87 | 346.85(323.26,374.26) | 501.81(467.88,538.06) | 1.31(1.26,1.36) |
| Trinidad and Tobago | both | 4649.79(4370.52,4949.15) | 9702.78(8971.23,10493.22) | 1.09 | 485.61(455.10,517.86) | 554.52(515.98,597.08) | 0.30(0.26,0.34) |
| Tunisia | both | 13746.88(12476.69,15114.20) | 55021.75(49388.75,60979.97) | 3.00 | 226.12(205.28,249.65) | 408.64(369.92,451.90) | 2.22(2.06,2.38) |
| Turkey | both | 88856.72(82307.84,96372.18) | 258309.21(234931.64,281494.65) | 1.91 | 202.48(188.56,219.42) | 273.83(248.82,298.61) | 2.27(1.77,2.77) |
| Turkmenistan | both | 3179.43(2853.59,3494.82) | 11029.11(9992.54,12056.49) | 2.47 | 122.60(111.04,133.85) | 217.05(196.73,236.55) | 2.06(1.99,2.13) |
| Uganda | both | 14569.05(13243.61,15944.41) | 47291.29(42551.58,52270.86) | 2.25 | 173.06(158.30,189.38) | 222.43(201.17,245.91) | 0.86(0.77,0.96) |
| Ukraine | both | 70034.01(62800.31,77781.82) | 83890.10(75646.99,93092.76) | 0.20 | 112.59(101.22,125.06) | 144.46(131.36,159.29) | 0.86(0.71,1.02) |
| United Arab Emirates | both | 4008.08(3560.82,4554.97) | 63860.27(55883.76,73094.27) | 14.93 | 397.24(364.66,431.78) | 589.90(545.98,641.76) | 1.39(1.16,1.62) |
| United Kingdom | both | 136375.06(123970.61,150897.48) | 306416.58(278952.88,336529.53) | 1.25 | 188.86(171.35,208.41) | 361.33(328.91,396.54) | 2.57(2.43,2.72) |
| United States of America | both | 657498.36(604168.13,714864.96) | 1566210.17(1452048.19,1695662.43) | 1.38 | 241.18(220.84,262.31) | 360.83(335.35,387.37) | 2.58(2.10,3.05) |
| Uruguay | both | 2748.77(2580.57,2942.75) | 8004.48(7202.44,8825.29) | 1.91 | 75.87(70.75,81.23) | 188.87(169.96,208.06) | 3.18(2.73,3.63) |
| Uzbekistan | both | 15751.03(14261.91,17214.00) | 85869.36(79265.73,92988.99) | 4.45 | 106.15(97.43,114.64) | 265.43(245.32,286.65) | 3.63(3.44,3.82) |
| Vanuatu | both | 283.26(256.11,313.49) | 1141.18(1049.08,1246.49) | 3.03 | 282.19(257.02,308.84) | 473.20(435.05,514.52) | 1.86(1.84,1.89) |
| Venezuela (Bolivarian Republic of) | both | 34086.03(31062.10,37089.40) | 111387.76(102869.80,121456.13) | 2.27 | 277.16(253.47,303.06) | 360.09(333.62,392.97) | 1.01(0.91,1.10) |
| Viet nam | both | 75184.78(68831.44,81886.82) | 280427.33(257046.32,305442.27) | 2.73 | 164.93(150.26,180.07) | 271.41(249.76,294.87) | 1.77(1.68,1.87) |
| Virginia | both | 16160.19(14721.38,17683.09) | 42170.98(38535.60,45763.08) | 1.61 | 234.82(213.44,256.49) | 363.46(334.68,392.83) | 2.69(2.24,3.15) |
| Yemen | both | 10044.87(9006.59,11156.34) | 48533.70(43651.48,54068.89) | 3.83 | 145.90(131.49,161.79) | 241.73(217.44,268.59) | 1.83(1.73,1.93) |
| Zambia | both | 6603.44(6018.51,7235.15) | 20513.54(18587.29,22549.97) | 2.11 | 171.51(157.84,186.93) | 204.04(188.45,222.40) | 0.55(0.50,0.60) |
| Zimbabwe | both | 10690.12(9704.57,11719.57) | 29454.07(26766.23,32520.09) | 1.76 | 206.32(187.69,225.16) | 309.69(282.94,341.29) | 1.54(1.49,1.58) |
| Monaco | both | 54.03(48.12,60.54) | 123.99(110.42,140.08) | 1.29 | 111.94(100.15,124.99) | 209.58(188.85,233.88) | 2.21(2.20,2.23) |
| San Marino | both | 40.11(36.07,45.09) | 106.63(95.72,119.42) | 1.66 | 136.14(122.63,152.32) | 231.43(207.90,258.20) | 1.85(1.83,1.87) |
| Saint Kitts and Nevis | both | 121.58(113.57,130.42) | 321.50(294.65,352.87) | 1.64 | 356.69(333.13,383.31) | 429.24(395.73,467.29) | 0.50(0.46,0.55) |
| Cook Islands | both | 58.98(54.89,63.50) | 122.86(115.54,131.65) | 1.08 | 390.07(364.46,417.65) | 591.63(556.04,631.45) | 1.41(1.27,1.54) |
| Nauru | both | 21.15(19.35,23.10) | 44.30(40.50,48.53) | 1.09 | 318.93(298.08,342.47) | 517.47(480.26,557.06) | 1.65(1.60,1.69) |
| Niue | both | 8.64(7.94,9.42) | 12.89(11.95,13.91) | 0.49 | 424.00(389.36,464.62) | 703.98(653.63,759.99) | 1.78(1.65,1.90) |
| Palau | both | 47.98(44.45,52.04) | 140.95(129.48,153.47) | 1.94 | 362.71(338.16,393.10) | 607.88(565.49,657.07) | 1.83(1.68,1.98) |
| Tokelau | both | 4.11(3.81,4.43) | 6.78(6.26,7.38) | 0.65 | 311.90(287.53,337.01) | 505.30(467.83,553.05) | 1.70(1.62,1.78) |
| Tuvalu | both | 25.34(23.30,27.49) | 60.71(55.93,65.98) | 1.40 | 311.50(286.61,337.35) | 528.08(486.39,573.61) | 1.93(1.83,2.02) |
| Afghanistan | male | 8104.85(7216.62,9084.77) | 43073.95(37716.56,49029.23) | 4.31 | 206.34(184.30,230.70) | 373.92(335.46,419.30) | 2.13(2.09,2.17) |
| Albania | male | 1523.72(1357.85,1705.12) | 3052.59(2720.46,3426.58) | 1.00 | 113.60(101.71,126.73) | 174.18(155.92,194.17) | 1.24(1.17,1.31) |
| Algeria | male | 16193.49(14593.93,18020.77) | 77391.41(69248.50,86927.68) | 3.78 | 208.43(188.36,232.78) | 372.31(333.53,415.36) | 2.10(1.99,2.21) |
| American Samoa | male | 109.30(100.14,120.55) | 223.91(207.64,242.39) | 1.05 | 583.74(538.50,635.91) | 835.09(775.61,902.04) | 1.21(1.04,1.38) |
| Andorra | male | 45.42(40.44,50.91) | 152.45(134.87,175.04) | 2.36 | 140.05(124.82,157.24) | 232.95(207.29,263.37) | 1.78(1.76,1.80) |
| Angola | male | 7013.37(6362.25,7775.65) | 25717.23(23155.54,28531.21) | 2.67 | 245.11(224.76,269.30) | 310.79(282.58,341.98) | 0.92(0.85,1.00) |
| Antigua and Barbuda | male | 76.84(70.92,83.54) | 231.35(209.77,252.80) | 2.01 | 319.29(293.83,347.38) | 442.41(403.34,481.14) | 1.13(1.09,1.16) |
| Argentina | male | 26296.73(24226.54,28310.06) | 66443.13(59486.10,73616.30) | 1.53 | 170.46(157.35,183.26) | 280.37(251.22,310.47) | 1.49(1.34,1.63) |
| Armenia | male | 2127.83(1917.64,2370.06) | 4395.75(3951.43,4914.30) | 1.07 | 142.55(130.43,157.32) | 255.86(232.36,282.88) | 2.00(1.77,2.24) |
| Australia | male | 13266.22(12021.03,14412.02) | 35711.82(31336.12,40129.78) | 1.69 | 145.63(131.92,158.19) | 217.25(191.07,245.40) | 1.44(1.29,1.60) |
| Austria | male | 5613.43(5004.22,6232.53) | 15105.74(13074.57,16937.31) | 1.69 | 124.74(111.20,138.59) | 234.43(203.75,261.41) | 2.27(2.08,2.47) |
| Azerbaijan | male | 3675.03(3322.23,4021.00) | 14201.40(12612.07,15882.25) | 2.86 | 126.90(116.27,137.82) | 245.70(220.06,273.76) | 2.31(2.22,2.41) |
| Bahrain | male | 832.82(748.68,918.85) | 9800.77(8921.12,10809.08) | 10.77 | 464.52(426.01,505.79) | 771.02(731.79,813.20) | 1.73(1.49,1.97) |
| Bangladesh | male | 59558.36(54548.76,64808.95) | 166394.21(152518.73,180794.77) | 1.79 | 174.00(160.87,189.04) | 221.78(204.05,240.52) | 0.92(0.72,1.11) |
| Barbados | male | 349.11(324.44,373.91) | 760.17(684.59,836.96) | 1.18 | 294.94(272.76,317.00) | 387.52(352.93,421.76) | 0.88(0.85,0.92) |
| Belarus | male | 5924.73(5274.85,6551.49) | 7700.53(6832.73,8656.31) | 0.30 | 110.42(99.29,121.76) | 133.67(119.40,148.87) | 0.52(0.33,0.71) |
| Belgium | male | 9144.06(7958.05,10339.28) | 18331.51(16207.51,20539.25) | 1.00 | 147.80(129.22,166.73) | 234.11(208.99,260.47) | 1.56(1.54,1.58) |
| Belize | male | 101.40(92.83,110.02) | 520.82(475.61,571.68) | 4.14 | 180.69(165.46,196.90) | 297.05(272.25,324.68) | 1.58(1.47,1.69) |
| Benin | male | 1815.97(1650.52,2002.31) | 7566.84(6829.49,8391.73) | 3.17 | 162.10(146.91,179.40) | 241.28(216.11,266.95) | 1.37(1.29,1.46) |
| Bermuda | male | 69.13(62.47,76.38) | 139.74(125.96,153.85) | 1.02 | 227.04(205.33,248.67) | 286.80(259.98,312.52) | 0.70(0.65,0.75) |
| Bhutan | male | 294.25(262.36,330.97) | 939.04(851.84,1039.41) | 2.19 | 148.38(134.30,164.55) | 250.28(227.62,276.12) | 1.97(1.91,2.03) |
| Bolivia (Plurinational State of) | male | 3500.48(3156.75,3873.15) | 13603.55(12230.57,14992.70) | 2.89 | 187.52(169.36,208.18) | 273.45(245.18,302.20) | 1.25(1.16,1.34) |
| Bosnia and Herzegovina | male | 4441.65(3979.37,4984.29) | 9614.14(8563.06,10646.33) | 1.16 | 193.83(174.69,215.59) | 405.39(363.87,444.67) | 2.95(2.71,3.19) |
| Botswana | male | 710.36(643.44,777.52) | 2757.57(2508.22,3031.45) | 2.88 | 221.51(203.08,241.50) | 336.43(310.21,366.69) | 1.71(1.58,1.84) |
| Brazil | male | 133373.25(122901.39,143863.05) | 329152.33(301956.48,360633.55) | 1.47 | 248.28(229.13,268.19) | 281.60(259.14,308.33) | 0.73(0.58,0.89) |
| Brunei Darussalam | male | 558.96(518.53,603.74) | 1843.57(1718.09,1974.92) | 2.30 | 643.06(605.25,678.87) | 738.24(696.09,780.06) | 0.58(0.35,0.81) |
| Bulgaria | male | 10343.69(9208.52,11611.62) | 13242.73(11846.67,14785.45) | 0.28 | 189.82(171.27,210.67) | 274.70(246.68,304.86) | 1.38(1.26,1.50) |
| Burkina Faso | male | 3937.33(3558.62,4337.50) | 12764.70(11658.22,13943.73) | 2.24 | 166.89(152.25,183.27) | 228.48(208.41,250.08) | 1.09(1.07,1.12) |
| Burundi | male | 2448.38(2219.48,2682.83) | 6616.66(5961.53,7344.97) | 1.70 | 180.25(164.47,197.09) | 198.83(180.95,219.11) | 0.27(0.25,0.30) |
| Cambodia | male | 3786.15(3462.33,4153.06) | 20786.60(18763.77,23217.90) | 4.49 | 151.15(138.72,165.43) | 319.56(288.60,353.95) | 2.69(2.55,2.83) |
| Cameroon | male | 4135.93(3735.06,4532.41) | 19130.98(17403.14,20921.11) | 3.63 | 159.65(146.18,174.86) | 244.26(222.52,268.16) | 1.35(0.96,1.75) |
| Canada | male | 18915.43(17653.98,20206.16) | 54734.83(46984.91,62100.58) | 1.89 | 125.43(116.47,134.33) | 206.32(179.44,233.91) | 1.54(1.48,1.59) |
| Cabo Verde | male | 150.16(135.32,166.63) | 627.10(559.61,692.07) | 3.18 | 151.75(137.39,168.49) | 269.71(240.19,298.49) | 2.05(1.87,2.24) |
| Central African Republic | male | 1930.28(1746.78,2117.08) | 5535.90(4991.72,6168.25) | 1.87 | 247.95(226.50,270.46) | 326.78(296.90,359.53) | 1.04(0.98,1.10) |
| Chad | male | 2385.52(2155.80,2621.51) | 8411.67(7490.10,9229.81) | 2.53 | 154.23(139.35,170.77) | 215.60(191.45,238.58) | 1.12(0.94,1.30) |
| Chile | male | 10053.70(9031.88,11068.09) | 34877.61(30862.07,40450.01) | 2.47 | 186.50(166.85,205.13) | 317.39(282.46,363.67) | 1.86(1.74,1.99) |
| China | male | 981777.85(889757.57,1082763.78) | 1925546.64(1775614.04,2115927.45) | 0.96 | 172.39(156.27,190.24) | 212.66(196.26,232.11) | 0.81(0.61,1.01) |
| Colombia | male | 33787.01(30274.24,37325.03) | 88376.87(79840.97,98597.44) | 1.62 | 307.52(274.17,339.52) | 356.33(322.94,397.37) | 0.34(0.20,0.47) |
| Comoros | male | 205.32(188.97,223.22) | 514.05(471.17,564.55) | 1.50 | 160.02(147.17,172.83) | 187.76(172.47,206.62) | 0.56(0.53,0.59) |
| Congo | male | 1780.44(1608.86,1948.76) | 6165.05(5514.70,6889.75) | 2.46 | 269.06(246.71,294.43) | 319.80(290.49,353.38) | 0.56(0.53,0.60) |
| Costa Rica | male | 3004.04(2699.15,3317.73) | 9940.71(8656.62,11131.31) | 2.31 | 294.60(263.88,325.78) | 398.74(349.54,444.74) | 1.03(0.92,1.13) |
| C么te d'Ivoire | male | 4911.67(4439.56,5421.29) | 18269.82(16616.58,20208.49) | 2.72 | 167.31(152.85,183.34) | 233.09(211.71,256.21) | 1.05(0.93,1.16) |
| Croatia | male | 6413.19(5726.04,7187.30) | 9885.69(8857.08,10894.96) | 0.54 | 224.53(202.04,247.30) | 330.50(296.94,365.83) | 1.24(1.14,1.34) |
| Cuba | male | 16323.60(14758.13,17907.29) | 30512.36(27239.36,33835.63) | 0.87 | 302.76(272.27,332.17) | 389.57(351.11,430.30) | 0.81(0.68,0.95) |
| Cyprus | male | 1309.10(1206.76,1418.73) | 3120.60(2846.51,3432.10) | 1.38 | 339.66(307.29,373.88) | 364.63(329.87,401.55) | -0.18(-0.33,-0.02) |
| Czechia | male | 16507.81(14962.61,18618.50) | 37336.84(33522.43,41227.58) | 1.26 | 284.82(257.86,320.50) | 507.32(451.17,559.24) | 1.95(1.77,2.13) |
| Democratic Republic of the Congo | male | 23315.77(21027.76,25769.55) | 79878.13(72042.86,88465.92) | 2.43 | 235.65(214.10,259.24) | 304.93(278.46,335.67) | 0.90(0.82,0.99) |
| Denmark | male | 3440.58(3089.23,3803.73) | 8251.96(7446.69,9124.23) | 1.40 | 109.86(99.00,122.33) | 203.99(184.82,222.97) | 2.22(2.07,2.36) |
| Djibouti | male | 183.73(164.76,204.85) | 977.99(882.73,1081.49) | 4.32 | 161.70(147.94,176.42) | 213.72(194.96,233.58) | 0.99(0.93,1.05) |
| Dominica | male | 97.47(89.54,105.85) | 231.19(210.40,253.36) | 1.37 | 333.37(305.79,363.47) | 554.68(505.15,606.41) | 1.82(1.71,1.93) |
| Dominican Republic | male | 3036.01(2758.26,3331.89) | 11683.92(10672.85,12833.22) | 2.85 | 129.24(117.84,141.30) | 225.63(206.59,246.94) | 2.16(2.03,2.28) |
| Ecuador | male | 5542.48(5017.05,6055.08) | 23949.47(21618.73,26112.41) | 3.32 | 175.03(159.00,192.05) | 300.52(270.89,327.91) | 1.97(1.79,2.14) |
| Egypt | male | 22573.26(20568.25,24609.19) | 126407.60(113543.78,140843.55) | 4.60 | 114.36(104.77,124.21) | 281.06(251.37,313.02) | 3.33(3.17,3.49) |
| El Salvador | male | 3775.80(3397.91,4141.37) | 10605.44(9663.70,11581.89) | 1.81 | 227.11(203.19,251.09) | 407.67(370.81,447.09) | 2.31(2.00,2.62) |
| Equatorial Guinea | male | 289.27(264.27,318.69) | 1282.12(1150.34,1422.47) | 3.43 | 253.82(233.10,278.65) | 336.73(305.58,371.53) | 1.09(1.03,1.16) |
| Eritrea | male | 1149.49(1029.02,1278.64) | 4293.24(3824.64,4798.91) | 2.73 | 169.03(156.04,184.85) | 220.81(200.97,243.29) | 1.00(0.95,1.05) |
| Estonia | male | 1040.85(929.80,1154.03) | 1563.36(1409.39,1743.32) | 0.50 | 128.45(115.81,141.43) | 194.03(175.41,214.91) | 1.50(1.21,1.79) |
| Ethiopia | male | 24688.31(22554.10,27061.06) | 45661.32(41468.65,50234.02) | 0.85 | 187.29(172.70,204.50) | 158.75(145.70,174.02) | -0.87(-0.96,-0.78) |
| Micronesia (Federated States of) | male | 107.75(98.55,116.83) | 286.67(264.56,310.49) | 1.66 | 321.35(297.67,345.48) | 593.53(552.92,636.33) | 2.21(1.88,2.54) |
| Fiji | male | 1417.75(1310.94,1530.24) | 3863.46(3643.54,4100.72) | 1.73 | 554.80(520.51,590.01) | 840.99(802.97,881.59) | 1.27(1.12,1.43) |
| Finland | male | 5542.38(5101.61,6058.65) | 10938.79(9717.02,12136.84) | 0.97 | 190.41(175.59,207.68) | 293.34(261.54,325.70) | 1.65(1.55,1.74) |
| France | male | 33818.84(30473.08,37212.88) | 68045.77(60855.49,76919.44) | 1.01 | 102.51(92.64,112.57) | 152.60(137.10,170.21) | 1.28(1.19,1.37) |
| Gabon | male | 936.35(852.52,1022.53) | 2673.60(2440.31,2949.46) | 1.86 | 302.99(277.72,329.39) | 391.67(359.16,428.96) | 0.91(0.83,1.00) |
| Georgia | male | 3927.03(3508.24,4376.57) | 6933.46(6263.38,7618.22) | 0.77 | 142.64(128.45,157.29) | 329.44(297.85,360.10) | 2.31(0.52,4.13) |
| Germany | male | 118606.08(104607.94,133566.48) | 220819.56(197026.21,243888.19) | 0.86 | 237.19(209.80,265.35) | 356.86(319.22,392.28) | 1.18(0.97,1.38) |
| Ghana | male | 5929.90(5347.82,6612.13) | 27069.62(24680.20,29759.79) | 3.56 | 156.98(142.44,173.66) | 283.50(258.07,311.08) | 2.48(2.10,2.86) |
| Greece | male | 9926.09(8880.94,11198.04) | 18135.39(15798.89,20307.51) | 0.83 | 151.93(136.16,169.80) | 256.65(225.45,287.42) | 1.88(1.80,1.97) |
| Greenland | male | 34.31(30.69,38.62) | 101.72(89.65,115.37) | 1.96 | 127.66(115.70,142.04) | 245.67(220.28,274.62) | 2.31(2.15,2.48) |
| Grenada | male | 97.31(89.57,105.16) | 311.93(286.44,342.15) | 2.21 | 319.78(292.48,347.40) | 511.01(474.42,554.31) | 1.71(1.61,1.81) |
| Guam | male | 150.90(136.06,168.05) | 335.72(305.18,368.59) | 1.22 | 242.69(221.14,266.73) | 360.69(328.91,395.49) | 1.43(1.39,1.48) |
| Guatemala | male | 5319.25(4780.60,5796.04) | 27841.58(25424.59,30070.56) | 4.23 | 223.92(201.36,244.89) | 438.00(398.41,476.26) | 2.62(2.23,3.02) |
| Guinea | male | 2422.04(2204.22,2678.06) | 7019.56(6382.69,7737.31) | 1.90 | 133.83(121.75,147.75) | 208.22(189.94,229.63) | 1.42(1.25,1.58) |
| Guinea-Bissau | male | 417.40(381.80,460.26) | 1125.39(1019.95,1244.65) | 1.70 | 173.86(159.62,191.34) | 233.50(211.50,256.91) | 0.95(0.89,1.00) |
| Guyana | male | 906.65(832.35,988.82) | 1985.98(1810.79,2160.01) | 1.19 | 356.30(329.09,387.65) | 535.69(491.14,580.57) | 1.36(1.23,1.48) |
| Haiti | male | 5601.37(5053.53,6226.76) | 17390.26(15709.00,19273.73) | 2.10 | 278.61(253.36,307.34) | 382.57(346.16,420.98) | 1.09(1.05,1.13) |
| Honduras | male | 4073.14(3658.27,4534.49) | 15510.91(13907.79,17192.13) | 2.81 | 305.64(274.15,341.70) | 414.48(370.59,459.47) | 1.15(1.02,1.27) |
| Hungary | male | 12800.98(11386.35,14222.25) | 22366.58(19921.43,24761.35) | 0.75 | 214.03(191.57,236.74) | 343.79(306.97,379.72) | 1.62(1.28,1.95) |
| Iceland | male | 162.74(144.84,183.44) | 545.99(485.86,614.89) | 2.36 | 126.40(112.32,143.06) | 244.16(217.61,273.25) | 2.35(2.31,2.39) |
| India | male | 683693.21(618272.01,759212.38) | 2295107.17(2086917.86,2538349.79) | 2.36 | 203.80(185.81,225.52) | 329.90(300.42,363.68) | 1.39(1.29,1.49) |
| Indonesia | male | 103169.88(94149.32,113547.21) | 324545.11(296714.94,357505.48) | 2.15 | 165.29(151.17,181.22) | 245.13(225.22,266.67) | 1.42(1.33,1.50) |
| Iran (Islamic Republic of) | male | 32573.23(29443.48,36266.36) | 140611.48(127861.57,154214.04) | 3.32 | 173.83(158.21,192.35) | 308.53(282.46,338.07) | 2.32(2.18,2.45) |
| Iraq | male | 14914.78(13530.31,16430.34) | 71786.46(64517.72,78771.93) | 3.81 | 281.84(255.11,309.75) | 410.00(370.72,450.00) | 1.26(1.22,1.29) |
| Ireland | male | 1643.82(1482.20,1821.52) | 7895.46(6946.39,8920.25) | 3.80 | 90.75(81.85,100.74) | 251.29(221.55,283.47) | 3.71(3.64,3.79) |
| Israel | male | 3487.47(3198.42,3778.43) | 12572.13(11351.89,13953.89) | 2.60 | 163.96(149.48,178.30) | 260.99(235.41,290.00) | 1.84(1.41,2.27) |
| Italy | male | 76596.80(69253.73,84618.28) | 141900.21(127591.48,157291.71) | 0.85 | 216.28(195.53,237.67) | 317.54(284.62,349.62) | 1.62(1.10,2.15) |
| Jamaica | male | 2220.15(2046.00,2408.18) | 6540.37(5951.53,7178.19) | 1.95 | 258.04(236.75,280.87) | 440.68(401.48,481.92) | 1.78(1.61,1.94) |
| Japan | male | 148470.01(134630.66,162965.38) | 178865.56(161324.99,198114.14) | 0.20 | 189.48(172.69,207.50) | 203.70(184.19,225.70) | -0.03(-0.22,0.16) |
| Jordan | male | 3179.08(2896.76,3491.19) | 23112.56(20758.85,25476.50) | 6.27 | 294.03(268.92,322.79) | 427.58(386.65,467.44) | 1.17(1.06,1.27) |
| Kazakhstan | male | 9284.04(8299.31,10223.46) | 23119.97(20805.59,25899.79) | 1.49 | 133.75(120.04,146.53) | 248.76(224.85,276.79) | 2.32(2.25,2.40) |
| Kenya | male | 7583.91(6895.22,8309.71) | 30646.76(27798.21,33694.66) | 3.04 | 146.54(134.66,160.18) | 201.03(185.04,218.84) | 1.12(0.99,1.26) |
| Kiribati | male | 113.18(103.97,123.37) | 356.66(328.15,390.12) | 2.15 | 428.39(400.03,459.23) | 729.61(682.49,780.47) | 1.98(1.71,2.25) |
| Kuwait | male | 2797.38(2486.42,3115.39) | 14567.79(12929.00,16473.76) | 4.21 | 356.43(322.29,390.80) | 531.85(481.91,585.50) | 1.14(0.92,1.37) |
| Kyrgyzstan | male | 1663.48(1496.84,1830.00) | 4336.21(3842.52,4845.16) | 1.61 | 100.47(91.06,110.39) | 149.02(133.13,165.53) | 1.31(1.26,1.37) |
| Lao People's Democratic Republic | male | 2601.03(2359.17,2869.40) | 9294.30(8373.40,10243.83) | 2.57 | 209.14(191.02,231.28) | 328.52(296.12,362.42) | 1.56(1.54,1.57) |
| Latvia | male | 1605.47(1443.04,1789.05) | 2190.04(1957.06,2424.65) | 0.36 | 116.02(104.78,128.74) | 189.53(170.09,208.80) | 1.87(1.67,2.06) |
| Lebanon | male | 3175.01(2874.30,3536.13) | 10317.83(9215.48,11456.93) | 2.25 | 243.23(220.89,270.30) | 423.30(377.46,472.86) | 1.99(1.92,2.07) |
| Lesotho | male | 1049.91(951.09,1160.53) | 2170.62(1976.14,2386.14) | 1.07 | 206.48(189.11,226.08) | 298.07(275.41,324.60) | 1.59(1.47,1.71) |
| Liberia | male | 1261.46(1129.42,1402.94) | 4047.84(3621.98,4541.13) | 2.21 | 194.89(174.53,215.89) | 277.31(247.32,310.44) | 1.39(1.28,1.49) |
| Libya | male | 3548.06(3211.12,3944.22) | 17498.09(15391.35,19917.44) | 3.93 | 252.23(227.66,281.24) | 465.07(416.29,520.15) | 2.19(2.06,2.33) |
| Lithuania | male | 2179.61(1960.20,2435.41) | 2695.12(2414.16,3013.10) | 0.24 | 115.84(104.29,129.03) | 159.68(143.88,177.14) | 1.15(0.95,1.35) |
| Luxembourg | male | 245.03(219.82,271.71) | 1649.55(1463.13,1832.10) | 5.73 | 105.73(95.46,116.82) | 373.99(334.03,414.98) | 4.82(4.68,4.95) |
| North Macedonia | male | 2329.70(2096.28,2579.82) | 6038.13(5430.94,6683.96) | 1.59 | 229.01(206.48,252.80) | 398.21(360.37,440.01) | 1.96(1.77,2.15) |
| Madagascar | male | 4771.05(4331.48,5249.96) | 14615.02(13109.94,16072.79) | 2.06 | 149.38(136.02,163.30) | 185.41(167.66,203.82) | 0.78(0.75,0.82) |
| Malawi | male | 4496.26(4063.82,4953.49) | 12722.51(11518.16,14055.63) | 1.83 | 192.33(175.76,211.99) | 261.75(238.55,287.80) | 1.16(0.98,1.34) |
| Malaysia | male | 14179.11(13100.17,15318.03) | 52451.07(46956.24,57917.49) | 2.70 | 233.94(217.20,253.31) | 328.95(294.70,362.58) | 1.24(1.08,1.39) |
| Maldives | male | 124.06(111.77,137.56) | 710.09(634.67,790.42) | 4.72 | 196.71(177.16,217.39) | 285.71(255.29,316.48) | 1.24(1.13,1.35) |
| Mali | male | 3169.31(2833.35,3492.75) | 10528.10(9426.07,11541.69) | 2.32 | 134.37(120.61,147.99) | 187.47(167.98,206.76) | 1.16(1.12,1.20) |
| Malta | male | 460.51(413.86,506.95) | 1005.41(890.56,1119.21) | 1.18 | 232.93(209.20,256.02) | 309.06(276.23,341.27) | 1.04(0.92,1.15) |
| Marshall Islands | male | 76.39(68.19,84.66) | 224.97(204.77,248.49) | 1.95 | 522.08(475.95,567.76) | 792.98(729.93,866.00) | 1.43(1.32,1.55) |
| Mauritania | male | 656.78(601.34,719.96) | 1716.78(1553.05,1896.99) | 1.61 | 120.19(110.33,132.12) | 141.46(127.92,156.06) | 0.12(-0.08,0.32) |
| Mauritius | male | 1402.42(1287.73,1525.32) | 5175.29(4671.99,5684.38) | 2.69 | 310.84(284.42,341.21) | 610.47(559.03,663.82) | 2.94(2.61,3.27) |
| Mexico | male | 111888.35(103787.22,120262.52) | 308073.89(283605.09,330639.29) | 1.75 | 417.28(386.24,448.52) | 495.59(458.08,531.24) | 0.67(0.43,0.92) |
| Republic of Moldova | male | 3185.61(2873.11,3555.38) | 4645.98(4193.11,5150.04) | 0.46 | 150.48(135.94,167.50) | 203.99(184.91,225.07) | 1.07(0.85,1.29) |
| Mongolia | male | 490.65(434.84,552.82) | 1795.65(1592.29,2040.66) | 2.66 | 69.48(62.17,77.81) | 110.90(99.21,124.57) | 2.02(1.83,2.20) |
| Montenegro | male | 745.02(669.02,823.85) | 1433.06(1298.23,1585.59) | 0.92 | 238.75(215.79,264.28) | 352.85(320.11,387.39) | 1.25(1.18,1.33) |
| Morocco | male | 15556.00(13942.20,17271.58) | 64500.54(57907.15,71997.45) | 3.15 | 180.06(162.21,200.96) | 344.58(310.27,384.20) | 2.36(2.32,2.39) |
| Mozambique | male | 5937.92(5433.82,6517.17) | 18789.46(17030.50,20594.51) | 2.16 | 174.80(160.88,190.52) | 257.37(235.03,279.25) | 1.52(1.44,1.60) |
| Myanmar | male | 29028.14(26444.37,31825.74) | 76637.04(69969.50,83789.69) | 1.64 | 214.42(196.13,234.00) | 320.90(292.63,351.16) | 1.45(1.32,1.58) |
| Namibia | male | 862.32(790.18,948.45) | 2051.17(1858.52,2250.23) | 1.38 | 223.34(205.55,245.16) | 267.19(244.05,291.04) | 0.71(0.61,0.81) |
| Nepal | male | 10307.14(9264.49,11414.04) | 34702.76(31240.30,38485.12) | 2.37 | 153.97(139.67,170.18) | 274.20(246.75,303.96) | 1.86(1.73,2.00) |
| Netherlands | male | 13737.36(12335.45,15031.35) | 24949.07(22062.81,28048.56) | 0.82 | 159.48(143.01,174.58) | 202.33(179.50,224.89) | 0.68(0.64,0.73) |
| New Zealand | male | 2166.17(1940.39,2400.69) | 5616.98(5095.28,6167.69) | 1.59 | 120.79(108.66,133.64) | 188.13(172.24,205.51) | 1.20(0.95,1.45) |
| Nicaragua | male | 2934.49(2652.84,3259.23) | 10784.15(9800.93,11706.97) | 2.67 | 294.66(267.27,326.33) | 394.99(357.73,430.54) | 1.14(1.04,1.24) |
| Niger | male | 1590.37(1438.26,1751.47) | 7494.27(6714.10,8339.59) | 3.71 | 92.23(84.37,100.88) | 148.87(133.03,164.93) | 1.56(1.35,1.77) |
| Nigeria | male | 37678.75(34113.24,41362.80) | 88741.60(80735.45,97482.13) | 1.36 | 140.75(129.01,153.51) | 170.70(155.68,186.35) | 0.52(0.45,0.60) |
| Democratic People's Republic of Korea | male | 12031.26(10869.66,13436.41) | 31159.90(27698.46,34701.80) | 1.59 | 136.58(124.61,150.66) | 202.32(181.34,225.15) | 1.34(1.32,1.37) |
| Northern Mariana Islands | male | 65.00(58.15,72.60) | 138.03(123.90,153.63) | 1.12 | 311.29(285.19,339.95) | 487.81(447.59,531.67) | 1.65(1.51,1.79) |
| Norway | male | 4973.57(4514.11,5465.20) | 9518.80(8611.52,10566.43) | 0.91 | 199.19(180.54,220.03) | 262.69(238.35,289.76) | 0.50(0.36,0.64) |
| Oman | male | 1710.59(1528.50,1906.53) | 10403.74(9192.21,11684.04) | 5.08 | 244.21(221.18,267.82) | 433.41(393.48,474.56) | 1.93(1.82,2.03) |
| Pakistan | male | 75449.86(67771.75,83379.25) | 253471.02(228041.38,281646.99) | 2.36 | 191.67(172.68,211.74) | 297.48(267.38,330.35) | 1.74(1.62,1.85) |
| Palestine | male | 1333.17(1214.71,1466.15) | 8581.29(7774.66,9327.39) | 5.44 | 267.64(244.05,296.44) | 486.72(443.15,525.54) | 2.11(2.03,2.20) |
| Panama | male | 2379.35(2142.98,2629.92) | 8519.59(7647.20,9322.62) | 2.58 | 269.34(241.69,299.32) | 403.18(361.82,441.17) | 1.60(1.48,1.73) |
| Papua New Guinea | male | 5820.80(5333.46,6337.46) | 22155.64(20348.10,24129.29) | 2.81 | 382.87(354.89,411.74) | 515.39(478.07,554.83) | 1.05(1.00,1.10) |
| Paraguay | male | 2163.25(1966.94,2373.83) | 9156.39(8328.76,10093.53) | 3.23 | 166.74(152.16,184.08) | 291.77(265.00,319.94) | 1.60(1.39,1.81) |
| Peru | male | 8670.49(7872.57,9594.56) | 31859.57(28801.62,35396.35) | 2.67 | 125.75(113.69,139.33) | 195.80(176.62,218.01) | 1.56(1.45,1.66) |
| Philippines | male | 37657.22(34561.72,40997.76) | 107421.68(99030.74,116191.64) | 1.85 | 200.73(185.43,217.87) | 231.21(213.36,250.14) | 0.33(0.18,0.48) |
| Poland | male | 41978.19(38573.81,46185.59) | 78219.54(71283.81,85905.58) | 0.86 | 209.41(191.99,229.22) | 312.18(285.13,340.21) | 1.52(1.26,1.78) |
| Portugal | male | 12919.21(11327.05,14354.49) | 25644.15(22978.90,28352.34) | 0.98 | 222.42(194.71,245.86) | 342.27(304.07,379.16) | 1.66(1.40,1.91) |
| Puerto Rico | male | 6023.15(5481.56,6617.80) | 11060.98(10044.14,12069.37) | 0.84 | 356.03(324.14,393.52) | 489.07(447.07,536.56) | 1.12(0.94,1.29) |
| Qatar | male | 979.53(854.32,1115.71) | 18620.15(16854.85,20765.00) | 18.01 | 497.63(456.97,541.98) | 816.78(771.41,872.95) | 1.69(1.40,1.99) |
| Romania | male | 18683.14(16547.80,21308.24) | 28071.21(24814.60,31511.26) | 0.50 | 146.14(129.67,163.81) | 218.75(193.27,245.87) | 1.33(1.25,1.40) |
| Russian Federation | male | 76453.36(69141.81,84413.30) | 124651.30(113538.46,137851.51) | 0.63 | 100.81(92.10,110.91) | 142.23(130.43,156.60) | 1.13(0.99,1.27) |
| Rwanda | male | 3100.82(2795.77,3385.42) | 7205.66(6502.83,7973.90) | 1.32 | 183.82(168.49,200.82) | 201.07(182.87,221.09) | 0.15(0.04,0.27) |
| Saint Lucia | male | 184.10(170.77,198.93) | 546.92(500.56,596.65) | 1.97 | 408.54(378.43,443.34) | 509.26(469.73,551.43) | 0.84(0.76,0.91) |
| Saint Vincent and the Grenadines | male | 136.17(126.52,147.55) | 342.88(314.26,376.54) | 1.52 | 372.59(346.30,404.28) | 497.71(457.89,541.94) | 0.92(0.88,0.96) |
| Samoa | male | 207.49(191.01,225.97) | 473.20(431.23,519.12) | 1.28 | 353.82(326.17,383.46) | 505.52(463.40,551.60) | 1.16(1.04,1.28) |
| Sao Tome and Principe | male | 57.15(50.66,64.03) | 162.71(145.14,181.03) | 1.85 | 166.12(148.34,185.60) | 239.83(214.32,267.46) | 1.33(1.22,1.45) |
| Saudi Arabia | male | 17241.47(15684.90,18937.94) | 105423.82(93329.00,119635.79) | 5.11 | 294.06(270.18,320.40) | 475.63(431.03,526.32) | 1.69(1.65,1.72) |
| Senegal | male | 4348.18(3897.95,4828.45) | 13600.59(12247.75,15028.30) | 2.13 | 222.23(199.81,246.95) | 296.41(267.44,328.60) | 1.29(1.07,1.51) |
| Serbia | male | 12822.94(11509.12,14209.44) | 21091.47(18767.71,23324.63) | 0.64 | 230.03(207.67,252.45) | 365.54(323.23,402.92) | 1.54(1.45,1.63) |
| Seychelles | male | 69.74(62.73,76.64) | 324.98(292.33,357.28) | 3.66 | 247.65(223.66,273.60) | 512.37(467.43,559.95) | 2.58(2.42,2.73) |
| Sierra Leone | male | 1038.17(954.02,1124.27) | 3154.93(2865.48,3486.80) | 2.04 | 95.85(88.12,103.96) | 136.33(124.46,151.16) | 1.27(1.18,1.35) |
| Singapore | male | 4329.90(3967.75,4679.83) | 12233.53(11025.79,13605.37) | 1.83 | 281.52(256.67,304.68) | 297.48(270.95,327.51) | -0.01(-0.14,0.12) |
| Slovakia | male | 5049.78(4525.86,5642.23) | 9189.74(8168.74,10245.61) | 0.82 | 187.20(166.93,208.63) | 250.26(224.35,276.76) | 0.84(0.78,0.91) |
| Slovenia | male | 2222.00(1973.89,2483.05) | 3926.80(3497.00,4352.71) | 0.77 | 206.45(182.92,229.37) | 259.44(233.84,285.65) | 0.46(0.27,0.65) |
| Solomon Islands | male | 348.00(317.32,380.41) | 1453.42(1331.64,1581.68) | 3.18 | 310.26(285.62,336.07) | 565.15(525.21,608.92) | 2.33(2.21,2.46) |
| Somalia | male | 2915.44(2629.19,3220.36) | 10818.12(9762.97,12109.36) | 2.71 | 174.81(159.32,190.90) | 219.87(201.07,240.29) | 0.80(0.76,0.84) |
| South Africa | male | 23107.92(21187.01,25100.53) | 74001.05(68964.73,79328.79) | 2.20 | 213.72(198.70,230.92) | 317.56(297.04,339.55) | 1.43(1.31,1.55) |
| Republic of Korea | male | 41494.50(38349.41,45007.24) | 109909.45(100012.33,121389.48) | 1.65 | 206.26(190.88,223.18) | 272.09(250.05,298.36) | 0.79(0.69,0.89) |
| South Sudan | male | 2658.51(2416.80,2929.55) | 5430.18(4897.87,6012.35) | 1.04 | 161.18(147.39,176.34) | 203.54(185.37,223.46) | 0.81(0.76,0.86) |
| Spain | male | 55318.53(50424.37,59937.35) | 103700.78(90190.74,116658.56) | 0.87 | 240.37(220.97,259.41) | 316.11(278.16,353.12) | 0.42(0.11,0.72) |
| Sri Lanka | male | 17310.96(15736.51,18838.06) | 64570.93(59631.71,70280.27) | 2.73 | 268.14(243.55,293.23) | 524.07(485.66,567.65) | 2.46(2.34,2.59) |
| Sudan | male | 11483.60(10284.08,12801.83) | 51910.45(46361.09,58063.05) | 3.52 | 186.44(166.46,208.09) | 363.98(325.53,407.60) | 2.32(2.28,2.35) |
| Suriname | male | 477.52(433.18,526.54) | 1584.20(1455.25,1726.03) | 2.32 | 315.36(286.47,346.82) | 508.46(470.59,551.37) | 1.86(1.78,1.94) |
| Eswatini | male | 427.54(389.85,469.87) | 1252.78(1149.80,1363.57) | 1.93 | 263.16(243.36,285.07) | 385.40(356.95,416.77) | 1.53(1.30,1.75) |
| Sweden | male | 8791.90(7879.09,9822.76) | 16807.75(14833.06,18931.24) | 0.91 | 161.27(144.35,180.94) | 243.02(216.77,271.83) | 1.57(1.44,1.70) |
| Switzerland | male | 7462.19(6716.38,8379.45) | 15463.82(13565.82,17514.84) | 1.07 | 178.62(160.41,200.22) | 245.68(218.22,276.53) | 1.00(0.86,1.14) |
| Syrian Arab Republic | male | 8161.19(7395.22,9016.78) | 25141.57(22214.20,27925.15) | 2.08 | 218.26(198.21,240.98) | 341.16(305.91,374.40) | 1.44(1.41,1.48) |
| Taiwan (Province of China) | male | 19092.40(17590.78,20622.65) | 44541.00(41353.57,47719.31) | 1.33 | 192.92(178.38,209.04) | 265.81(249.72,282.37) | 0.98(0.84,1.12) |
| Tajikistan | male | 2130.06(1937.48,2356.26) | 10747.70(9683.68,11879.64) | 4.05 | 119.66(109.43,131.56) | 265.63(240.90,293.26) | 2.93(2.83,3.04) |
| United Republic of Tanzania | male | 7728.98(7121.40,8459.32) | 28486.76(25702.51,31397.53) | 2.69 | 123.84(114.48,134.58) | 183.44(166.87,201.33) | 1.66(1.50,1.82) |
| Thailand | male | 39491.26(35868.38,43418.53) | 121895.80(110046.93,136139.57) | 2.09 | 182.62(165.45,201.10) | 262.08(238.12,290.34) | 0.93(0.74,1.11) |
| Bahamas | male | 268.85(243.90,293.67) | 827.73(744.97,920.15) | 2.08 | 292.98(267.79,317.81) | 397.74(360.78,437.43) | 0.97(0.93,1.01) |
| Gambia | male | 336.23(303.45,373.93) | 1279.05(1151.95,1410.93) | 2.80 | 141.05(128.23,156.20) | 210.45(189.74,232.59) | 1.41(1.36,1.46) |
| Timor-Leste | male | 326.82(293.30,362.59) | 1375.61(1237.71,1519.92) | 3.21 | 153.68(138.02,169.90) | 285.22(254.74,316.43) | 1.97(1.83,2.11) |
| Togo | male | 921.37(829.35,1014.55) | 3834.91(3450.35,4247.92) | 3.16 | 123.02(112.08,134.77) | 175.56(159.88,192.88) | 1.23(1.13,1.33) |
| Tonga | male | 106.46(97.76,116.32) | 217.54(201.03,235.74) | 1.04 | 321.15(296.19,349.66) | 510.27(471.48,551.31) | 1.69(1.57,1.81) |
| Trinidad and Tobago | male | 2288.34(2080.72,2477.56) | 5160.46(4771.02,5615.79) | 1.26 | 485.05(442.38,523.59) | 588.75(545.66,640.37) | 0.53(0.48,0.57) |
| Tunisia | male | 7819.82(7074.56,8648.26) | 30475.49(27183.88,33951.91) | 2.90 | 254.51(230.19,282.11) | 458.43(411.83,508.70) | 2.35(2.08,2.63) |
| Turkey | male | 42313.26(38612.75,46429.39) | 130318.98(116381.66,144414.13) | 2.08 | 192.66(175.93,211.27) | 277.91(249.26,307.86) | 2.55(2.06,3.04) |
| Turkmenistan | male | 1474.70(1303.66,1638.49) | 5451.48(4830.54,6064.24) | 2.70 | 119.35(107.25,132.08) | 214.26(191.18,237.01) | 1.99(1.91,2.07) |
| Uganda | male | 7665.00(6948.54,8482.60) | 25176.09(22592.62,27893.50) | 2.28 | 190.12(172.89,209.41) | 260.73(236.09,288.45) | 1.09(0.97,1.21) |
| Ukraine | male | 31714.58(27999.10,36089.26) | 40384.51(36290.57,45662.12) | 0.27 | 115.77(103.23,130.43) | 155.17(140.21,173.89) | 0.99(0.81,1.17) |
| United Arab Emirates | male | 2914.80(2549.31,3335.85) | 50273.53(43423.60,58303.76) | 16.25 | 383.84(352.54,421.14) | 583.36(537.03,638.60) | 1.46(1.26,1.66) |
| United Kingdom | male | 70230.51(63522.89,77681.00) | 162187.27(147815.53,179096.92) | 1.31 | 206.17(186.26,227.92) | 398.86(363.84,438.77) | 2.74(2.59,2.89) |
| United States of America | male | 344696.23(315764.94,374424.45) | 872376.70(809239.05,943683.60) | 1.53 | 260.83(238.34,282.57) | 410.42(381.87,441.21) | 2.69(2.25,3.14) |
| Uruguay | male | 1221.35(1126.58,1319.23) | 4360.92(3877.47,4809.12) | 2.57 | 72.75(67.12,78.35) | 220.13(196.20,242.16) | 3.90(3.31,4.50) |
| Uzbekistan | male | 8180.16(7270.70,9003.73) | 43761.14(39822.80,48058.89) | 4.35 | 114.54(103.39,125.35) | 280.16(256.38,306.61) | 3.51(3.33,3.69) |
| Vanuatu | male | 156.78(141.13,173.96) | 591.71(540.92,646.15) | 2.77 | 301.16(272.04,330.27) | 482.63(441.76,525.52) | 1.69(1.66,1.73) |
| Venezuela (Bolivarian Republic of) | male | 16861.48(15243.45,18612.47) | 56602.29(51658.46,62294.39) | 2.36 | 279.15(250.83,311.53) | 375.29(343.88,412.90) | 1.18(1.07,1.30) |
| Viet nam | male | 31455.83(28297.62,34466.10) | 133102.95(120924.42,146836.17) | 3.23 | 155.17(139.83,170.25) | 276.24(250.91,302.04) | 2.10(2.01,2.19) |
| Virginia | male | 8406.88(7523.64,9452.11) | 22949.05(20868.84,25098.24) | 1.73 | 250.35(224.38,280.62) | 405.65(370.63,441.74) | 2.76(2.34,3.20) |
| Yemen | male | 5085.88(4508.91,5679.58) | 23725.43(21096.76,26588.48) | 3.66 | 145.99(130.85,163.37) | 236.65(210.36,266.69) | 1.73(1.67,1.79) |
| Zambia | male | 3448.58(3142.07,3798.23) | 11514.32(10386.84,12755.24) | 2.34 | 182.04(167.17,199.37) | 238.62(218.74,260.60) | 0.94(0.85,1.02) |
| Zimbabwe | male | 4951.10(4430.20,5456.01) | 11823.39(10577.01,13220.01) | 1.39 | 193.85(174.65,212.88) | 277.40(249.90,310.24) | 1.37(1.30,1.43) |
| Monaco | male | 26.48(23.44,29.76) | 65.17(57.50,74.13) | 1.46 | 119.69(105.91,133.79) | 226.89(201.77,254.87) | 2.29(2.26,2.31) |
| San Marino | male | 20.67(18.42,23.24) | 54.89(48.74,61.86) | 1.66 | 144.81(129.25,163.24) | 247.41(221.16,278.96) | 1.89(1.86,1.92) |
| Saint Kitts and Nevis | male | 53.55(49.10,58.22) | 171.97(155.98,190.68) | 2.21 | 336.64(309.49,363.14) | 455.21(417.04,497.15) | 0.98(0.95,1.02) |
| Cook Islands | male | 29.50(27.20,31.92) | 60.07(56.19,64.33) | 1.04 | 373.39(346.36,401.52) | 597.20(560.07,638.66) | 1.58(1.42,1.74) |
| Nauru | male | 11.63(10.58,12.74) | 23.12(20.99,25.45) | 0.99 | 344.06(318.76,371.36) | 549.02(509.55,591.54) | 1.60(1.56,1.65) |
| Niue | male | 4.49(4.08,4.90) | 6.59(6.06,7.16) | 0.47 | 452.33(410.87,495.03) | 728.69(673.45,792.59) | 1.69(1.57,1.80) |
| Palau | male | 25.36(23.28,27.62) | 77.62(70.75,84.87) | 2.06 | 373.23(344.83,406.21) | 619.24(574.23,671.72) | 1.80(1.66,1.94) |
| Tokelau | male | 1.83(1.66,1.99) | 3.31(3.03,3.63) | 0.81 | 290.57(263.93,317.49) | 486.69(445.82,535.75) | 1.89(1.83,1.94) |
| Tuvalu | male | 11.37(10.30,12.42) | 30.53(28.08,33.48) | 1.68 | 315.51(287.73,343.39) | 516.37(477.06,563.65) | 1.81(1.74,1.88) |
| Afghanistan | female | 10292.88(9185.31,11452.97) | 53456.74(47591.16,60797.76) | 4.19 | 245.10(219.54,271.85) | 451.70(404.09,502.25) | 2.24(2.19,2.28) |
| Albania | female | 1245.71(1103.41,1389.20) | 2767.85(2454.06,3127.31) | 1.22 | 99.74(89.02,111.11) | 148.44(131.84,166.05) | 1.29(1.23,1.35) |
| Algeria | female | 17146.28(15288.96,18944.68) | 82118.84(73977.78,91388.27) | 3.79 | 215.64(193.48,237.97) | 400.66(362.81,442.74) | 2.29(2.23,2.35) |
| American Samoa | female | 88.29(80.37,97.20) | 220.25(202.90,238.36) | 1.49 | 503.47(460.83,550.10) | 805.05(743.82,870.02) | 1.61(1.45,1.76) |
| Andorra | female | 31.55(27.91,35.48) | 113.83(99.96,129.42) | 2.61 | 110.90(98.34,124.73) | 190.99(169.53,216.06) | 1.87(1.81,1.93) |
| Angola | female | 4837.75(4339.72,5383.37) | 19221.51(17250.95,21477.82) | 2.97 | 169.25(153.80,186.67) | 202.89(183.22,223.03) | 0.72(0.69,0.76) |
| Antigua and Barbuda | female | 86.16(80.38,92.84) | 238.20(215.65,265.10) | 1.76 | 309.83(286.27,337.66) | 420.30(383.70,462.46) | 0.97(0.90,1.04) |
| Argentina | female | 29821.26(27236.83,32584.31) | 64032.23(57635.13,70573.11) | 1.15 | 173.79(159.05,189.85) | 246.73(221.48,272.97) | 0.97(0.87,1.07) |
| Armenia | female | 2895.25(2642.86,3184.05) | 5590.61(5013.47,6273.72) | 0.93 | 169.98(156.23,185.09) | 274.20(246.98,301.76) | 1.65(1.40,1.90) |
| Australia | female | 11534.34(10324.35,12678.00) | 29651.78(26508.87,32815.95) | 1.57 | 112.12(100.31,123.42) | 171.15(154.24,189.32) | 1.31(0.96,1.65) |
| Austria | female | 6618.74(6035.33,7344.70) | 13445.03(11864.43,15200.04) | 1.03 | 118.03(107.35,130.51) | 195.90(173.08,220.50) | 1.77(1.72,1.83) |
| Azerbaijan | female | 4425.12(3958.72,4875.53) | 16490.41(14845.87,18373.95) | 2.73 | 134.19(120.91,147.52) | 263.37(239.87,291.27) | 2.56(2.38,2.73) |
| Bahrain | female | 480.34(434.64,526.95) | 4574.80(4257.23,4914.16) | 8.52 | 410.29(378.81,444.26) | 727.56(691.77,763.31) | 2.05(1.84,2.25) |
| Bangladesh | female | 48202.89(43841.97,52752.40) | 170457.91(157084.47,184570.38) | 2.54 | 156.81(143.98,169.87) | 226.06(209.06,244.64) | 1.25(1.10,1.40) |
| Barbados | female | 482.99(459.72,507.37) | 900.32(831.43,976.60) | 0.86 | 338.82(316.70,361.61) | 407.87(376.40,440.87) | 0.49(0.44,0.55) |
| Belarus | female | 7501.74(6708.84,8459.51) | 8402.71(7431.88,9438.27) | 0.12 | 111.02(99.53,124.13) | 121.12(106.60,135.29) | 0.23(0.06,0.39) |
| Belgium | female | 10012.18(9024.74,11071.53) | 17091.97(15037.51,19428.44) | 0.71 | 143.08(128.44,158.05) | 207.37(184.34,233.36) | 1.11(1.06,1.17) |
| Belize | female | 133.34(123.28,143.77) | 666.93(604.18,723.99) | 4.00 | 243.28(225.01,264.41) | 368.93(336.19,399.97) | 1.45(1.28,1.62) |
| Benin | female | 1873.44(1690.05,2055.06) | 8222.80(7478.19,9081.13) | 3.39 | 149.93(136.22,164.88) | 237.78(216.11,263.55) | 1.60(1.52,1.67) |
| Bermuda | female | 61.50(56.39,67.20) | 118.14(105.11,133.48) | 0.92 | 175.37(160.64,192.04) | 226.88(202.55,253.39) | 0.67(0.60,0.75) |
| Bhutan | female | 280.96(253.29,310.58) | 807.85(738.87,886.80) | 1.88 | 156.10(141.48,171.30) | 242.64(221.97,266.88) | 1.58(1.52,1.63) |
| Bolivia (Plurinational State of) | female | 4023.94(3628.81,4441.39) | 13356.09(12143.24,14668.72) | 2.32 | 195.82(176.99,217.06) | 257.74(234.92,283.40) | 0.95(0.88,1.03) |
| Bosnia and Herzegovina | female | 4746.11(4245.10,5282.01) | 9254.06(8347.82,10343.77) | 0.95 | 184.88(166.81,203.66) | 346.47(314.67,384.49) | 2.56(2.40,2.72) |
| Botswana | female | 826.55(754.94,908.57) | 3377.06(3094.43,3707.95) | 3.09 | 215.92(197.87,237.28) | 343.37(316.89,374.59) | 1.92(1.82,2.02) |
| Brazil | female | 137802.28(126616.56,148494.02) | 330782.31(302396.85,361547.51) | 1.40 | 239.70(221.23,259.55) | 255.84(234.06,278.86) | 0.36(0.24,0.48) |
| Brunei Darussalam | female | 359.59(333.54,391.12) | 1204.79(1107.38,1330.69) | 2.35 | 500.24(466.88,539.04) | 531.37(489.58,582.52) | 0.07(-0.07,0.21) |
| Bulgaria | female | 10470.99(9270.44,11669.37) | 12428.90(11083.75,14129.10) | 0.19 | 173.88(155.03,192.41) | 232.01(208.69,260.72) | 0.97(0.84,1.11) |
| Burkina Faso | female | 4578.12(4127.13,5065.75) | 14533.23(13057.07,16034.42) | 2.17 | 164.78(148.88,181.55) | 214.93(194.33,238.69) | 0.98(0.94,1.01) |
| Burundi | female | 2309.52(2092.39,2549.01) | 5156.27(4645.58,5715.97) | 1.23 | 141.74(129.26,155.82) | 156.07(142.61,171.73) | 0.28(0.25,0.31) |
| Cambodia | female | 4864.13(4459.51,5303.33) | 22803.08(20727.60,25198.99) | 3.69 | 151.49(139.72,164.02) | 290.33(263.90,320.83) | 2.25(2.01,2.48) |
| Cameroon | female | 3911.17(3567.10,4301.43) | 18535.66(16896.75,20463.71) | 3.74 | 141.29(129.63,154.39) | 225.77(206.83,248.90) | 1.56(1.19,1.94) |
| Canada | female | 16592.44(15276.41,17947.35) | 44196.48(38880.48,50514.03) | 1.66 | 100.91(92.63,109.62) | 159.83(141.32,181.02) | 1.19(1.07,1.31) |
| Cabo Verde | female | 199.24(178.83,219.94) | 746.29(669.16,828.97) | 2.75 | 152.15(136.35,169.28) | 292.65(262.56,324.50) | 2.40(2.17,2.63) |
| Central African Republic | female | 1476.27(1328.98,1635.68) | 4193.36(3787.98,4627.93) | 1.84 | 169.85(154.18,187.70) | 229.63(207.98,252.63) | 1.15(1.09,1.21) |
| Chad | female | 2515.14(2272.83,2766.86) | 8610.10(7757.61,9523.34) | 2.42 | 148.88(134.51,164.17) | 230.72(209.61,254.25) | 1.58(1.43,1.74) |
| Chile | female | 11518.90(10312.17,12847.84) | 36839.55(32830.11,41421.93) | 2.20 | 195.02(174.39,218.32) | 305.60(273.91,342.43) | 1.75(1.52,1.98) |
| China | female | 920681.35(837393.92,1016090.63) | 1813574.44(1660401.34,1982946.91) | 0.97 | 177.63(161.55,196.58) | 189.19(173.75,205.79) | 0.37(0.15,0.59) |
| Colombia | female | 32477.66(28847.86,36001.69) | 82896.50(74721.04,92600.54) | 1.55 | 285.63(253.06,318.82) | 298.54(269.09,332.00) | -0.20(-0.36,-0.05) |
| Comoros | female | 180.79(165.33,195.64) | 433.78(395.57,474.90) | 1.40 | 133.65(122.74,144.93) | 146.39(133.72,160.87) | 0.30(0.26,0.34) |
| Congo | female | 1409.77(1274.53,1554.46) | 4890.79(4391.92,5428.80) | 2.47 | 180.30(164.62,198.08) | 245.45(224.24,268.88) | 1.15(1.08,1.22) |
| Costa Rica | female | 2580.29(2351.48,2827.60) | 8408.16(7524.74,9419.45) | 2.26 | 246.06(222.93,272.90) | 303.04(272.57,338.26) | 0.39(0.26,0.52) |
| C么te d'Ivoire | female | 4102.28(3677.77,4573.64) | 18106.38(16425.52,19868.74) | 3.41 | 155.33(141.38,170.89) | 250.58(230.00,274.07) | 1.74(1.59,1.90) |
| Croatia | female | 6570.39(5873.75,7391.91) | 8775.62(7766.01,9950.80) | 0.34 | 190.29(171.30,210.88) | 260.92(232.91,294.80) | 0.97(0.90,1.03) |
| Cuba | female | 13656.19(12559.61,14871.74) | 23208.35(20748.43,25942.23) | 0.70 | 251.41(230.84,274.62) | 286.49(259.01,319.31) | 0.40(0.22,0.58) |
| Cyprus | female | 1408.23(1290.39,1544.63) | 2849.37(2585.86,3149.43) | 1.02 | 310.97(283.80,338.48) | 311.92(282.76,344.11) | -0.54(-0.75,-0.33) |
| Czechia | female | 15189.21(13509.92,16870.81) | 30090.03(26435.36,33448.27) | 0.98 | 225.48(201.46,248.74) | 380.01(336.59,420.02) | 1.73(1.50,1.97) |
| Democratic Republic of the Congo | female | 17945.34(16131.14,20067.33) | 57635.78(51465.52,64152.93) | 2.21 | 158.26(145.20,175.08) | 207.11(186.34,229.56) | 0.94(0.92,0.97) |
| Denmark | female | 3322.07(2981.16,3645.91) | 7633.25(6749.01,8519.14) | 1.30 | 95.27(85.02,105.29) | 182.16(161.59,201.89) | 2.12(2.03,2.21) |
| Djibouti | female | 144.22(129.16,161.56) | 689.66(617.94,769.38) | 3.78 | 140.21(128.27,153.77) | 169.33(153.91,187.37) | 0.66(0.62,0.70) |
| Dominica | female | 114.01(106.78,122.00) | 172.53(158.50,188.48) | 0.51 | 320.49(297.09,347.29) | 434.34(398.79,473.20) | 0.89(0.76,1.02) |
| Dominican Republic | female | 3368.97(3100.45,3676.02) | 11597.70(10664.59,12750.72) | 2.44 | 135.10(124.84,147.62) | 221.79(204.09,243.69) | 1.96(1.80,2.12) |
| Ecuador | female | 6165.23(5699.02,6728.07) | 25954.31(23825.28,28340.99) | 3.21 | 191.46(175.97,209.23) | 310.40(284.89,338.70) | 1.85(1.63,2.08) |
| Egypt | female | 30567.18(27889.85,33376.66) | 125298.92(114039.25,138187.76) | 3.10 | 167.29(154.25,182.04) | 311.79(285.25,340.80) | 2.32(2.21,2.43) |
| El Salvador | female | 3498.22(3189.15,3826.38) | 11346.95(10242.22,12438.69) | 2.24 | 187.09(169.77,206.06) | 335.53(302.29,367.97) | 1.79(1.66,1.91) |
| Equatorial Guinea | female | 233.52(209.95,260.32) | 1002.81(898.61,1111.04) | 3.29 | 165.71(150.24,182.76) | 240.04(217.05,264.57) | 1.49(1.40,1.57) |
| Eritrea | female | 1160.64(1045.79,1281.39) | 3871.95(3512.79,4280.77) | 2.34 | 144.58(132.59,159.10) | 175.98(161.36,192.33) | 0.72(0.69,0.75) |
| Estonia | female | 1224.60(1094.07,1360.82) | 1529.27(1349.97,1719.30) | 0.25 | 116.62(104.38,129.43) | 164.69(147.49,182.86) | 1.33(1.06,1.60) |
| Ethiopia | female | 21345.49(19389.80,23531.15) | 40350.48(36295.20,44463.83) | 0.89 | 151.73(139.14,165.77) | 136.40(125.03,149.22) | -0.63(-0.76,-0.51) |
| Micronesia (Federated States of) | female | 95.61(88.59,103.46) | 271.17(249.80,296.48) | 1.84 | 289.67(270.14,312.10) | 568.21(525.37,617.18) | 2.43(2.06,2.81) |
| Fiji | female | 1398.50(1287.17,1529.21) | 3499.26(3287.25,3713.05) | 1.50 | 504.77(471.47,539.95) | 760.75(720.09,806.13) | 1.32(1.09,1.56) |
| Finland | female | 6711.29(6077.89,7500.57) | 11252.47(9965.66,12612.70) | 0.68 | 192.13(173.97,212.50) | 281.22(251.54,311.50) | 1.43(1.31,1.54) |
| France | female | 32159.67(29389.14,35325.31) | 63413.39(55668.85,70529.31) | 0.97 | 83.55(76.29,91.84) | 128.97(114.64,143.06) | 1.67(1.53,1.81) |
| Gabon | female | 612.32(554.84,676.76) | 1809.16(1642.19,2009.53) | 1.95 | 179.17(163.80,197.07) | 251.24(228.71,277.55) | 1.27(1.10,1.44) |
| Georgia | female | 4595.67(4101.42,5124.06) | 6955.38(6231.60,7735.34) | 0.51 | 137.50(123.72,152.20) | 281.19(253.07,307.39) | 1.74(0.50,3.00) |
| Germany | female | 145646.38(131177.93,163783.79) | 211185.49(184911.05,237500.95) | 0.45 | 242.00(217.45,273.41) | 323.54(286.01,358.36) | 0.74(0.49,1.00) |
| Ghana | female | 7217.84(6512.73,8003.69) | 28247.11(25427.77,31592.74) | 2.91 | 173.27(157.98,192.98) | 242.37(219.72,269.50) | 1.68(1.38,1.99) |
| Greece | female | 9877.69(8795.65,10969.68) | 15384.50(13302.98,17342.03) | 0.56 | 136.80(122.72,150.72) | 199.05(172.97,222.32) | 1.27(1.16,1.38) |
| Greenland | female | 13.65(12.18,15.29) | 50.52(44.08,57.48) | 2.70 | 66.39(60.20,73.35) | 146.44(128.95,164.36) | 2.56(2.44,2.67) |
| Grenada | female | 119.52(112.34,127.70) | 265.26(241.69,290.51) | 1.22 | 335.46(312.72,362.82) | 451.47(413.69,491.89) | 1.06(1.02,1.09) |
| Guam | female | 144.69(132.11,158.91) | 296.03(268.21,329.99) | 1.05 | 272.83(252.31,297.45) | 337.02(306.17,373.32) | 0.69(0.50,0.87) |
| Guatemala | female | 5624.72(5120.44,6189.89) | 33075.45(30411.07,35934.60) | 4.88 | 233.35(212.50,257.46) | 438.52(404.27,475.25) | 2.15(1.97,2.33) |
| Guinea | female | 2597.25(2370.61,2839.75) | 7708.74(7019.59,8434.42) | 1.97 | 138.38(126.73,151.32) | 219.51(200.56,238.78) | 1.60(1.55,1.66) |
| Guinea-Bissau | female | 441.70(403.80,482.89) | 1358.83(1223.89,1498.82) | 2.08 | 161.19(148.15,176.83) | 237.64(214.84,262.49) | 1.38(1.30,1.45) |
| Guyana | female | 1071.72(986.26,1157.76) | 2087.85(1917.06,2248.70) | 0.95 | 406.32(377.35,436.30) | 532.56(492.63,572.16) | 0.85(0.72,0.98) |
| Haiti | female | 7500.72(6858.16,8186.18) | 25065.96(22621.99,27577.66) | 2.34 | 327.73(301.56,357.64) | 472.67(431.51,519.58) | 1.23(1.21,1.25) |
| Honduras | female | 3399.47(3058.85,3755.59) | 14807.14(13353.51,16458.36) | 3.36 | 244.42(219.72,269.93) | 351.17(316.11,390.30) | 1.04(0.95,1.13) |
| Hungary | female | 13287.97(11647.57,14895.03) | 19364.21(17355.26,21550.30) | 0.46 | 183.84(163.40,204.94) | 253.50(229.14,279.96) | 1.17(1.01,1.33) |
| Iceland | female | 147.01(132.75,163.91) | 426.51(377.66,481.92) | 1.90 | 109.80(98.67,123.22) | 195.04(174.07,219.49) | 1.89(1.85,1.92) |
| India | female | 543518.30(488084.88,604014.80) | 1911472.93(1729078.34,2109564.77) | 2.52 | 181.34(164.16,200.61) | 286.94(260.49,316.66) | 1.38(1.32,1.45) |
| Indonesia | female | 101030.31(92761.79,110314.63) | 307156.10(281210.00,336203.69) | 2.04 | 149.38(137.37,162.93) | 228.95(210.78,249.34) | 1.51(1.45,1.56) |
| Iran (Islamic Republic of) | female | 28876.03(25972.46,31974.33) | 150870.88(137026.82,166557.99) | 4.22 | 166.15(150.04,183.60) | 338.09(307.89,370.83) | 2.70(2.57,2.82) |
| Iraq | female | 15440.55(14105.07,16941.50) | 73237.47(66226.24,79846.67) | 3.74 | 292.20(266.47,318.66) | 439.64(399.87,480.44) | 1.40(1.33,1.48) |
| Ireland | female | 1505.16(1352.56,1677.07) | 6254.25(5542.11,7104.95) | 3.16 | 76.32(68.58,85.52) | 192.70(171.25,219.13) | 3.33(3.10,3.55) |
| Israel | female | 4178.98(3794.85,4600.08) | 13249.89(11966.33,14417.50) | 2.17 | 173.24(156.68,191.09) | 255.51(230.50,277.80) | 1.39(0.99,1.79) |
| Italy | female | 85685.86(78008.91,94085.68) | 129154.66(115894.79,143135.26) | 0.51 | 207.65(188.76,227.91) | 261.11(232.87,290.79) | 0.94(0.52,1.35) |
| Jamaica | female | 2826.74(2660.58,2997.00) | 6875.87(6284.81,7429.31) | 1.43 | 305.46(285.87,327.06) | 451.66(413.31,487.70) | 1.29(1.12,1.46) |
| Japan | female | 105170.22(95449.20,115820.65) | 129962.17(116879.32,144987.33) | 0.24 | 123.37(111.90,135.49) | 132.49(119.85,147.95) | -0.35(-0.59,-0.10) |
| Jordan | female | 2771.28(2548.45,3008.89) | 15308.42(13829.98,16959.68) | 4.52 | 305.47(283.07,328.65) | 355.16(322.18,390.42) | 0.18(-0.10,0.46) |
| Kazakhstan | female | 14894.10(13400.32,16457.16) | 34148.18(30623.14,37995.33) | 1.29 | 186.09(167.67,205.52) | 322.56(289.53,358.44) | 2.15(2.03,2.26) |
| Kenya | female | 6436.44(5805.67,7128.49) | 21243.00(19007.35,23519.18) | 2.30 | 114.08(104.64,124.74) | 124.64(113.06,137.52) | 0.24(0.19,0.30) |
| Kiribati | female | 108.50(100.02,117.16) | 284.02(258.83,310.06) | 1.62 | 360.78(335.79,388.03) | 510.29(468.94,553.33) | 1.16(1.10,1.22) |
| Kuwait | female | 1456.04(1290.33,1608.76) | 9549.17(8401.13,10845.49) | 5.56 | 328.22(297.05,358.17) | 449.77(402.63,498.82) | 0.83(0.60,1.05) |
| Kyrgyzstan | female | 1950.72(1731.62,2185.03) | 4873.34(4331.87,5397.03) | 1.50 | 104.75(93.41,116.43) | 153.92(137.57,169.18) | 1.29(1.24,1.34) |
| Lao People's Democratic Republic | female | 3016.51(2720.85,3299.28) | 10227.73(9268.56,11272.74) | 2.39 | 221.80(201.66,243.39) | 352.54(321.41,386.24) | 1.58(1.55,1.60) |
| Latvia | female | 1986.12(1782.37,2194.58) | 2451.58(2195.10,2736.68) | 0.23 | 111.38(100.26,122.98) | 172.81(156.22,190.91) | 1.80(1.64,1.96) |
| Lebanon | female | 2916.85(2654.58,3216.95) | 9918.75(8969.15,10949.74) | 2.40 | 218.70(199.26,240.52) | 349.96(316.13,387.20) | 1.61(1.57,1.64) |
| Lesotho | female | 1169.37(1074.56,1277.65) | 2632.19(2409.18,2880.35) | 1.25 | 194.63(179.53,213.34) | 304.82(280.44,332.17) | 1.87(1.76,1.98) |
| Liberia | female | 1077.10(975.66,1186.91) | 3866.62(3455.39,4319.06) | 2.59 | 181.64(164.82,200.19) | 269.43(241.73,299.91) | 1.65(1.52,1.78) |
| Libya | female | 2651.59(2385.16,2944.72) | 15512.06(13764.94,17593.01) | 4.85 | 235.00(210.38,260.30) | 443.94(398.40,494.06) | 2.27(2.20,2.35) |
| Lithuania | female | 2358.19(2116.52,2660.60) | 2591.73(2306.15,2920.17) | 0.10 | 101.00(90.93,113.21) | 124.81(112.39,138.73) | 0.92(0.76,1.09) |
| Luxembourg | female | 261.55(240.09,286.53) | 1283.95(1140.56,1449.62) | 3.91 | 98.36(89.38,107.91) | 302.02(268.91,339.35) | 4.11(3.91,4.30) |
| North Macedonia | female | 2381.10(2116.17,2646.94) | 5644.75(5028.47,6301.00) | 1.37 | 222.74(199.31,247.10) | 368.44(330.13,409.84) | 1.87(1.73,2.01) |
| Madagascar | female | 4253.04(3849.44,4673.23) | 13140.81(11833.81,14625.88) | 2.09 | 126.33(115.76,137.85) | 153.45(139.03,169.28) | 0.68(0.65,0.70) |
| Malawi | female | 4282.62(3866.67,4733.14) | 9912.85(8935.85,11034.80) | 1.31 | 160.34(146.08,176.76) | 177.45(161.57,194.47) | 0.35(0.25,0.44) |
| Malaysia | female | 15173.60(14039.12,16279.83) | 51727.30(46171.11,57292.58) | 2.41 | 248.24(230.86,266.90) | 339.13(303.43,375.38) | 1.16(1.06,1.26) |
| Maldives | female | 108.21(97.88,119.58) | 443.97(395.94,493.62) | 3.10 | 204.11(186.07,225.55) | 254.68(228.66,281.67) | 0.62(0.46,0.77) |
| Mali | female | 3811.35(3467.52,4211.30) | 13067.25(11834.44,14435.15) | 2.43 | 152.08(138.70,167.22) | 231.15(210.77,255.22) | 1.49(1.46,1.53) |
| Malta | female | 521.47(471.24,581.73) | 1056.73(923.64,1202.51) | 1.03 | 233.42(210.41,261.36) | 318.58(282.24,356.04) | 1.01(0.98,1.05) |
| Marshall Islands | female | 66.23(60.00,72.77) | 216.03(197.29,235.98) | 2.26 | 483.18(442.47,527.64) | 796.35(731.82,859.25) | 1.74(1.63,1.85) |
| Mauritania | female | 787.55(720.65,861.19) | 2446.28(2225.90,2682.54) | 2.11 | 131.93(121.09,144.96) | 193.52(176.58,212.20) | 1.08(0.97,1.20) |
| Mauritius | female | 1392.80(1292.97,1501.11) | 5181.13(4678.52,5609.23) | 2.72 | 299.35(276.88,323.01) | 572.85(522.22,614.23) | 2.65(2.40,2.89) |
| Mexico | female | 128733.55(119741.46,137648.84) | 312119.81(288761.98,335058.26) | 1.42 | 458.97(425.64,494.52) | 460.93(427.32,495.57) | -0.10(-0.20,0.00) |
| Republic of Moldova | female | 3890.44(3484.17,4350.94) | 4984.05(4442.17,5576.85) | 0.28 | 153.77(137.75,170.75) | 191.20(171.96,212.78) | 0.92(0.77,1.08) |
| Mongolia | female | 503.07(446.54,563.04) | 1730.28(1536.18,1955.49) | 2.44 | 68.34(60.69,76.75) | 99.37(88.87,110.90) | 1.59(1.49,1.69) |
| Montenegro | female | 718.01(642.22,804.27) | 1409.84(1256.52,1572.62) | 0.96 | 206.90(185.62,230.80) | 317.25(283.65,352.03) | 1.43(1.38,1.48) |
| Morocco | female | 15766.22(14134.48,17522.57) | 64804.50(57743.35,71901.36) | 3.11 | 177.24(158.21,198.05) | 345.36(308.90,380.83) | 2.47(2.41,2.53) |
| Mozambique | female | 5419.07(4897.81,5972.55) | 14956.33(13584.18,16547.89) | 1.76 | 136.74(125.31,150.00) | 171.24(157.98,188.00) | 0.81(0.76,0.86) |
| Myanmar | female | 39839.16(36655.83,43224.65) | 104085.96(96708.48,112878.68) | 1.61 | 265.93(244.86,288.00) | 359.50(333.81,389.58) | 1.03(0.89,1.16) |
| Namibia | female | 1045.38(953.25,1137.47) | 2514.32(2280.60,2755.38) | 1.41 | 237.54(217.82,259.38) | 266.83(243.32,292.46) | 0.29(0.17,0.40) |
| Nepal | female | 8493.50(7614.90,9445.11) | 33244.68(29730.91,36864.55) | 2.91 | 138.51(125.72,154.27) | 232.89(208.74,259.33) | 1.69(1.61,1.77) |
| Netherlands | female | 13833.07(12847.63,15076.24) | 20878.69(18638.06,23345.46) | 0.51 | 143.81(131.64,157.78) | 161.57(144.57,179.22) | 0.25(0.17,0.33) |
| New Zealand | female | 2175.72(1961.15,2405.36) | 5200.37(4720.59,5687.61) | 1.39 | 111.82(100.45,124.28) | 160.75(146.25,174.89) | 0.79(0.41,1.17) |
| Nicaragua | female | 2877.99(2643.55,3132.01) | 10901.68(9801.39,11965.44) | 2.79 | 267.38(245.14,291.87) | 362.27(325.03,396.99) | 0.95(0.82,1.08) |
| Niger | female | 1587.47(1443.74,1744.47) | 9049.70(8141.29,9978.20) | 4.70 | 97.07(89.71,106.06) | 168.58(152.69,185.29) | 1.91(1.77,2.06) |
| Nigeria | female | 32798.77(30102.81,35778.38) | 98615.80(88575.41,109131.50) | 2.01 | 136.93(126.06,150.21) | 156.65(143.00,171.89) | 0.02(-0.19,0.23) |
| Democratic People's Republic of Korea | female | 14530.67(13071.17,16320.01) | 32381.15(28996.80,36318.84) | 1.23 | 132.57(119.38,148.31) | 195.59(175.38,218.12) | 1.33(1.29,1.37) |
| Northern Mariana Islands | female | 49.31(44.17,55.37) | 121.72(109.09,135.72) | 1.47 | 295.36(272.94,321.94) | 448.18(412.08,489.60) | 1.50(1.40,1.61) |
| Norway | female | 4737.93(4271.76,5240.19) | 8444.17(7574.11,9399.50) | 0.78 | 168.44(152.69,186.84) | 229.86(206.83,256.04) | 0.48(0.28,0.68) |
| Oman | female | 996.17(905.27,1095.53) | 4517.45(4071.24,5035.90) | 3.53 | 238.65(218.26,261.26) | 370.08(336.56,409.80) | 1.41(1.33,1.49) |
| Pakistan | female | 60976.73(55591.01,67252.17) | 241467.48(217999.57,268438.83) | 2.96 | 178.36(163.97,195.98) | 309.18(282.15,340.41) | 2.26(2.11,2.41) |
| Palestine | female | 1435.57(1311.86,1571.24) | 6936.72(6286.06,7559.59) | 3.83 | 250.46(229.58,273.73) | 415.86(380.11,452.48) | 1.98(1.85,2.11) |
| Panama | female | 2079.22(1891.43,2279.68) | 7567.43(6852.75,8446.51) | 2.64 | 244.94(222.50,271.09) | 357.62(324.26,399.26) | 1.10(1.00,1.19) |
| Papua New Guinea | female | 3573.42(3240.28,3939.53) | 16465.52(14954.14,18263.50) | 3.61 | 245.68(223.86,269.29) | 407.18(372.58,447.70) | 1.83(1.77,1.89) |
| Paraguay | female | 2514.57(2280.57,2752.30) | 8603.26(7824.50,9412.13) | 2.42 | 189.10(170.90,207.67) | 274.85(250.71,300.76) | 1.34(1.15,1.53) |
| Peru | female | 9124.28(8255.76,9994.32) | 32872.71(29592.44,36288.88) | 2.60 | 127.65(115.57,140.43) | 191.51(171.69,211.44) | 1.32(1.24,1.40) |
| Philippines | female | 39821.39(36561.43,43447.95) | 127604.29(117777.34,138302.82) | 2.20 | 210.55(193.38,230.09) | 265.45(244.56,288.30) | 0.59(0.40,0.78) |
| Poland | female | 45588.32(41667.46,49984.23) | 70251.41(63587.33,77739.59) | 0.54 | 201.08(183.32,219.17) | 247.73(224.46,272.22) | 0.94(0.68,1.19) |
| Portugal | female | 14877.48(13202.65,16840.30) | 28175.19(25209.81,31875.18) | 0.89 | 217.57(192.97,242.69) | 324.35(288.57,365.68) | 1.37(1.17,1.56) |
| Puerto Rico | female | 6554.32(6027.67,7105.87) | 11278.94(10245.87,12388.49) | 0.72 | 343.62(314.83,373.64) | 437.36(396.01,475.31) | 0.78(0.61,0.96) |
| Qatar | female | 339.90(302.13,378.54) | 5299.37(4640.73,5981.69) | 14.59 | 475.71(435.23,517.66) | 821.66(770.07,874.31) | 1.87(1.51,2.22) |
| Romania | female | 17549.83(15702.28,19783.23) | 24685.11(21841.31,27588.66) | 0.41 | 123.37(110.67,137.77) | 174.75(154.97,195.33) | 1.16(1.09,1.24) |
| Russian Federation | female | 100057.56(91563.12,109942.12) | 155964.50(142494.02,171494.40) | 0.56 | 101.30(92.54,111.28) | 139.77(127.67,152.53) | 1.26(1.16,1.36) |
| Rwanda | female | 2902.51(2627.21,3199.50) | 6791.90(6119.47,7499.45) | 1.34 | 139.50(126.97,152.83) | 150.95(137.49,166.66) | 0.22(0.17,0.26) |
| Saint Lucia | female | 255.60(238.96,273.69) | 645.44(591.89,710.51) | 1.53 | 500.87(466.64,541.85) | 584.28(537.11,636.20) | 0.36(0.30,0.43) |
| Saint Vincent and the Grenadines | female | 168.45(156.65,179.85) | 332.80(306.32,362.45) | 0.98 | 414.23(384.49,444.30) | 515.30(477.60,557.24) | 0.61(0.54,0.67) |
| Samoa | female | 171.40(157.80,186.75) | 461.99(423.95,503.99) | 1.70 | 310.55(286.68,338.18) | 525.34(481.34,573.12) | 1.83(1.66,2.00) |
| Sao Tome and Principe | female | 58.96(52.81,65.20) | 161.85(143.59,181.57) | 1.74 | 158.93(142.72,176.15) | 226.86(202.19,252.88) | 1.31(1.16,1.45) |
| Saudi Arabia | female | 9882.71(8984.34,10886.66) | 63776.67(57105.31,72452.10) | 5.45 | 263.99(241.85,289.58) | 441.81(399.45,491.13) | 1.83(1.70,1.95) |
| Senegal | female | 4095.49(3721.54,4536.26) | 14721.72(13212.60,16260.82) | 2.59 | 204.41(184.58,226.60) | 297.59(268.03,329.30) | 1.63(1.42,1.84) |
| Serbia | female | 13209.37(11822.85,14715.68) | 19785.67(17494.48,21991.77) | 0.50 | 214.39(193.31,237.58) | 306.36(271.45,338.28) | 1.28(1.21,1.34) |
| Seychelles | female | 67.53(60.59,73.93) | 290.05(262.73,319.59) | 3.30 | 217.30(194.19,240.40) | 494.76(450.32,540.27) | 2.92(2.74,3.11) |
| Sierra Leone | female | 997.54(914.20,1085.03) | 3986.12(3627.43,4360.16) | 3.00 | 94.81(87.57,103.17) | 173.26(158.61,189.66) | 2.37(2.26,2.49) |
| Singapore | female | 3912.03(3614.15,4195.83) | 8120.49(7237.34,9153.69) | 1.08 | 253.86(235.01,273.55) | 216.45(193.77,242.70) | -0.48(-0.75,-0.22) |
| Slovakia | female | 4971.80(4450.12,5538.75) | 8433.06(7493.80,9389.04) | 0.70 | 160.28(144.16,177.82) | 208.86(186.67,229.77) | 0.82(0.75,0.88) |
| Slovenia | female | 2463.16(2229.73,2723.73) | 3174.98(2848.37,3547.44) | 0.29 | 186.40(169.85,205.12) | 204.41(184.22,226.58) | -0.01(-0.14,0.13) |
| Solomon Islands | female | 270.23(246.50,296.68) | 1146.85(1040.62,1249.90) | 3.24 | 247.55(227.98,269.01) | 420.66(385.02,453.97) | 1.95(1.88,2.02) |
| Somalia | female | 2725.67(2467.15,3007.56) | 9643.85(8700.98,10779.05) | 2.54 | 145.80(133.64,160.36) | 173.93(158.66,192.25) | 0.61(0.58,0.64) |
| South Africa | female | 31515.86(29127.97,34126.51) | 96173.03(89711.12,103565.10) | 2.05 | 230.19(213.05,249.91) | 342.80(321.07,368.72) | 1.44(1.23,1.65) |
| Republic of Korea | female | 34573.23(31548.94,37805.47) | 85369.82(75739.75,94833.79) | 1.47 | 167.57(153.25,183.28) | 210.56(189.58,231.28) | 0.55(0.37,0.73) |
| South Sudan | female | 1905.99(1730.27,2082.69) | 4370.60(3933.15,4841.53) | 1.29 | 139.80(127.54,152.61) | 160.72(145.94,177.36) | 0.45(0.42,0.47) |
| Spain | female | 60212.18(54400.26,66210.80) | 92215.61(82386.26,102937.23) | 0.53 | 228.56(206.71,249.29) | 261.33(235.21,291.51) | 0.61(0.37,0.85) |
| Sri Lanka | female | 16104.06(14659.75,17647.48) | 71630.00(65113.89,78147.70) | 3.45 | 249.28(225.95,274.51) | 507.59(463.28,551.02) | 2.60(2.49,2.70) |
| Sudan | female | 11222.33(10100.10,12521.58) | 48675.12(43594.19,54463.46) | 3.34 | 186.38(167.72,206.98) | 355.25(319.17,396.54) | 2.26(2.20,2.31) |
| Suriname | female | 470.12(428.54,515.39) | 1557.52(1418.89,1720.48) | 2.31 | 296.47(270.97,324.35) | 475.93(436.68,522.47) | 1.83(1.74,1.92) |
| Eswatini | female | 516.61(473.74,563.68) | 1477.81(1365.83,1591.74) | 1.86 | 252.43(233.26,274.85) | 358.50(332.91,387.95) | 1.41(1.22,1.60) |
| Sweden | female | 8305.75(7395.44,9292.53) | 14280.72(12630.07,15957.83) | 0.72 | 135.05(120.75,150.91) | 199.05(177.00,221.65) | 1.51(1.36,1.66) |
| Switzerland | female | 7819.47(7053.94,8663.12) | 13357.21(11781.48,15047.08) | 0.71 | 165.38(148.72,183.95) | 207.20(183.35,233.86) | 0.84(0.72,0.96) |
| Syrian Arab Republic | female | 8089.34(7315.60,8937.99) | 26500.12(23536.03,29422.71) | 2.28 | 231.40(208.47,255.33) | 351.13(315.62,386.00) | 1.27(1.17,1.37) |
| Taiwan (Province of China) | female | 19880.23(18547.88,21226.38) | 47130.49(43735.57,50841.50) | 1.37 | 228.46(213.12,243.74) | 251.96(236.58,268.76) | 0.22(0.11,0.33) |
| Tajikistan | female | 2262.17(2037.53,2479.23) | 11122.74(10087.68,12294.17) | 3.92 | 121.77(110.05,133.52) | 270.75(246.41,299.49) | 3.13(3.04,3.23) |
| United Republic of Tanzania | female | 7459.31(6795.34,8150.54) | 26508.83(24100.58,29354.40) | 2.55 | 108.16(100.10,117.76) | 152.54(139.21,167.63) | 1.54(1.44,1.65) |
| Thailand | female | 42045.85(38451.82,45806.79) | 130391.16(116977.69,146486.19) | 2.10 | 180.88(165.06,197.39) | 250.83(226.60,279.42) | 0.66(0.49,0.82) |
| Bahamas | female | 299.93(274.84,326.36) | 845.79(763.93,928.93) | 1.82 | 284.24(260.50,310.11) | 367.83(333.43,402.30) | 0.77(0.73,0.82) |
| Gambia | female | 296.63(265.94,327.79) | 1385.59(1261.76,1527.11) | 3.67 | 136.68(124.29,152.07) | 218.10(199.06,240.21) | 1.70(1.64,1.75) |
| Timor-Leste | female | 302.95(273.77,335.25) | 1277.37(1154.20,1398.57) | 3.22 | 150.85(137.77,165.19) | 266.86(241.37,292.84) | 1.79(1.65,1.92) |
| Togo | female | 969.92(877.10,1070.09) | 4283.70(3895.96,4716.55) | 3.42 | 114.43(104.96,125.34) | 161.97(147.49,177.30) | 1.17(1.12,1.22) |
| Tonga | female | 129.82(119.87,140.73) | 224.94(207.88,242.61) | 0.73 | 368.48(342.65,397.86) | 495.15(459.57,532.90) | 1.02(1.00,1.04) |
| Trinidad and Tobago | female | 2361.45(2199.17,2531.43) | 4542.32(4162.33,4968.57) | 0.92 | 486.70(453.03,522.56) | 519.77(479.63,568.36) | 0.06(0.01,0.10) |
| Tunisia | female | 5927.06(5317.70,6591.91) | 24546.26(21952.63,27380.99) | 3.14 | 197.34(176.58,219.71) | 360.14(322.92,400.67) | 2.06(2.04,2.08) |
| Turkey | female | 46543.46(43177.97,50412.40) | 127990.24(115657.51,141465.68) | 1.75 | 211.57(197.44,228.77) | 269.10(243.42,296.39) | 2.01(1.51,2.53) |
| Turkmenistan | female | 1704.73(1524.04,1915.08) | 5577.63(5030.54,6161.60) | 2.27 | 125.64(112.46,140.15) | 219.89(199.07,242.02) | 2.11(2.03,2.19) |
| Uganda | female | 6904.05(6198.41,7595.85) | 22115.20(19821.07,24722.63) | 2.20 | 157.17(143.14,174.58) | 190.91(171.35,213.00) | 0.65(0.57,0.73) |
| Ukraine | female | 38319.43(34257.36,42983.48) | 43505.59(38797.22,48408.54) | 0.14 | 110.13(97.54,123.31) | 134.83(121.24,149.31) | 0.73(0.57,0.88) |
| United Arab Emirates | female | 1093.28(969.71,1223.27) | 13586.74(12061.94,15461.77) | 11.43 | 419.13(380.53,456.47) | 601.88(556.14,653.68) | 1.31(0.97,1.65) |
| United Kingdom | female | 66144.56(60153.16,73137.86) | 144229.30(130564.44,158347.56) | 1.18 | 170.68(155.28,189.16) | 322.80(293.49,355.14) | 2.39(2.23,2.54) |
| United States of America | female | 312802.13(287376.43,339271.73) | 693833.47(640535.54,754306.73) | 1.22 | 222.29(203.40,240.75) | 313.76(290.24,337.81) | 2.44(1.94,2.95) |
| Uruguay | female | 1527.42(1423.07,1648.66) | 3643.56(3225.90,4083.09) | 1.39 | 78.62(72.82,84.99) | 160.48(143.00,179.37) | 2.48(2.17,2.79) |
| Uzbekistan | female | 7570.87(6797.63,8422.49) | 42108.22(38340.21,46306.87) | 4.56 | 98.05(88.67,107.84) | 251.81(229.91,276.18) | 3.76(3.55,3.96) |
| Vanuatu | female | 126.49(114.41,139.88) | 549.47(500.32,605.45) | 3.34 | 259.81(236.05,285.25) | 464.97(424.55,509.41) | 2.09(2.07,2.11) |
| Venezuela (Bolivarian Republic of) | female | 17224.55(15589.23,18884.92) | 54785.47(49994.19,60606.80) | 2.18 | 275.01(248.63,301.18) | 345.11(315.48,381.61) | 0.84(0.75,0.93) |
| Viet nam | female | 43728.96(39921.66,48002.43) | 147324.39(135096.32,161710.65) | 2.37 | 172.92(156.94,190.00) | 267.17(245.39,292.65) | 1.53(1.43,1.62) |
| Virginia | female | 7753.32(6881.37,8645.56) | 19221.93(17374.43,21200.94) | 1.48 | 219.87(193.81,244.86) | 323.88(295.53,353.83) | 2.62(2.13,3.11) |
| Yemen | female | 4958.99(4434.92,5526.48) | 24808.26(22213.10,27685.52) | 4.00 | 145.02(129.16,160.51) | 246.74(221.49,274.51) | 1.95(1.81,2.10) |
| Zambia | female | 3154.86(2843.78,3458.10) | 8999.23(8117.92,9980.22) | 1.85 | 158.60(145.41,173.21) | 171.73(157.63,188.95) | 0.20(0.17,0.23) |
| Zimbabwe | female | 5739.02(5196.64,6340.22) | 17630.68(16012.96,19376.31) | 2.07 | 217.54(197.94,239.83) | 337.25(307.89,370.09) | 1.63(1.56,1.71) |
| Monaco | female | 27.56(24.19,31.26) | 58.83(51.67,66.85) | 1.13 | 104.28(92.44,117.44) | 192.36(171.83,215.33) | 2.13(2.08,2.17) |
| San Marino | female | 19.45(17.27,21.87) | 51.75(45.94,58.64) | 1.66 | 126.73(113.25,143.43) | 217.15(192.10,244.69) | 1.86(1.83,1.89) |
| Saint Kitts and Nevis | female | 68.03(63.27,73.33) | 149.53(133.96,164.79) | 1.20 | 373.06(346.33,403.57) | 402.53(366.07,440.16) | 0.04(-0.03,0.11) |
| Cook Islands | female | 29.48(27.36,31.89) | 62.80(58.40,67.71) | 1.13 | 410.74(382.97,441.80) | 586.66(545.63,632.86) | 1.23(1.12,1.33) |
| Nauru | female | 9.52(8.67,10.45) | 21.18(19.33,23.28) | 1.23 | 294.44(272.27,315.01) | 490.18(453.45,530.14) | 1.76(1.71,1.81) |
| Niue | female | 4.15(3.83,4.51) | 6.29(5.85,6.79) | 0.52 | 399.18(367.89,434.13) | 680.65(629.97,736.71) | 1.87(1.75,1.99) |
| Palau | female | 22.62(20.85,24.74) | 63.34(58.02,69.23) | 1.80 | 352.53(326.43,382.21) | 590.47(548.44,640.11) | 1.84(1.67,2.02) |
| Tokelau | female | 2.29(2.12,2.46) | 3.47(3.22,3.79) | 0.52 | 327.66(302.72,353.38) | 523.88(484.79,572.96) | 1.60(1.48,1.72) |
| Tuvalu | female | 13.96(12.89,15.20) | 30.18(27.70,32.74) | 1.16 | 309.44(287.02,335.34) | 541.60(495.43,590.53) | 2.05(1.94,2.17) |

**Supplementary Table 4.** The deaths and age-standardized death rate of type 2 diabetes in 1990 and 2019, and its temporal trends from 1990 to 2019.

| **Nation** | **Sex** | **Death Cases No. (95% UI)** | | **Change in absolute number (%)** | **ASDR per 100,000 No.(95% UI)** | | **1990-2019 EAPC No. (95%CI)** |
| --- | --- | --- | --- | --- | --- | --- | --- |
|  |  | **1990** | **2019** |  | **1990** | **2019** |  |
| Afghanistan | both | 1855.06(1206.30,2762.06) | 4331.04(2506.36,6392.21) | 1.33 | 27.82(18.48,40.50) | 39.88(23.39,58.00) | 1.63(1.48,1.78) |
| Albania | both | 76.61(68.56,84.61) | 149.37(111.34,198.65) | 0.95 | 4.16(3.72,4.61) | 3.44(2.58,4.55) | -1.17(-1.66,-0.67) |
| Algeria | both | 1531.06(1098.94,2116.94) | 4995.30(3848.83,6447.47) | 2.26 | 17.01(12.64,23.51) | 18.18(13.91,23.39) | 0.72(0.43,1.00) |
| American Samoa | both | 15.90(14.04,18.42) | 43.26(37.32,50.29) | 1.72 | 80.53(71.34,94.26) | 98.09(84.71,113.59) | 0.60(0.29,0.91) |
| Andorra | both | 3.21(2.39,4.40) | 8.48(6.54,10.64) | 1.64 | 7.25(5.52,9.68) | 5.55(4.27,7.00) | -0.95(-1.03,-0.86) |
| Angola | both | 1371.54(1061.81,1719.63) | 3691.29(2970.09,4610.37) | 1.69 | 41.03(32.79,50.02) | 41.23(34.25,50.36) | -0.03(-0.07,0.02) |
| Antigua and Barbuda | both | 31.43(28.54,34.04) | 53.82(46.76,61.81) | 0.71 | 56.91(51.81,61.52) | 58.22(50.55,66.57) | -0.32(-0.50,-0.14) |
| Argentina | both | 6573.57(6223.57,6868.94) | 10218.40(9426.08,10970.75) | 0.55 | 21.11(19.91,22.07) | 18.56(17.18,19.91) | -0.99(-1.27,-0.70) |
| Armenia | both | 451.04(425.27,479.02) | 1182.04(987.06,1378.54) | 1.62 | 17.62(16.63,18.77) | 28.58(23.86,33.21) | 0.72(-0.01,1.45) |
| Australia | both | 2098.11(1953.77,2203.47) | 4122.26(3617.78,4512.08) | 0.96 | 11.04(10.22,11.62) | 8.89(7.87,9.68) | -1.06(-1.34,-0.77) |
| Austria | both | 1521.69(1414.60,1600.99) | 2283.57(2012.24,2485.92) | 0.50 | 12.16(11.27,12.76) | 10.98(9.80,11.88) | 0.05(-0.42,0.52) |
| Azerbaijan | both | 530.54(486.01,586.78) | 1953.36(1599.29,2362.43) | 2.68 | 10.74(9.80,11.92) | 22.95(18.86,27.51) | 2.10(1.59,2.61) |
| Bahrain | both | 95.58(80.58,112.87) | 700.10(554.23,878.38) | 6.32 | 77.76(66.23,91.01) | 126.95(102.53,154.58) | 2.04(1.52,2.57) |
| Bangladesh | both | 10362.75(8824.54,12113.26) | 30639.22(24632.66,37232.48) | 1.96 | 26.97(22.86,31.05) | 29.03(23.31,35.16) | 0.01(-0.62,0.64) |
| Barbados | both | 210.56(194.34,223.80) | 299.91(250.90,348.35) | 0.42 | 68.36(63.17,72.64) | 60.72(50.94,70.56) | -0.86(-1.07,-0.66) |
| Belarus | both | 541.59(509.21,568.89) | 357.49(288.78,451.96) | -0.34 | 4.21(3.95,4.43) | 2.21(1.78,2.78) | -3.74(-4.27,-3.22) |
| Belgium | both | 1736.26(1593.15,1841.10) | 1662.04(1447.32,1816.73) | -0.04 | 11.00(10.08,11.68) | 6.08(5.38,6.61) | -2.30(-2.47,-2.12) |
| Belize | both | 38.83(35.20,42.23) | 145.71(126.88,166.38) | 2.75 | 43.07(39.00,46.84) | 56.26(48.90,63.95) | 1.00(0.39,1.61) |
| Benin | both | 473.64(402.68,557.82) | 1416.12(1112.54,1816.62) | 1.99 | 26.32(22.35,30.79) | 34.41(27.29,43.43) | 0.96(0.70,1.21) |
| Bermuda | both | 18.42(16.87,19.97) | 23.30(19.48,28.23) | 0.26 | 31.31(28.66,33.93) | 17.00(14.23,20.61) | -2.36(-2.46,-2.25) |
| Bhutan | both | 36.26(26.11,47.84) | 146.88(112.11,189.04) | 3.05 | 18.57(13.75,24.23) | 30.38(23.35,38.74) | 1.82(1.76,1.89) |
| Bolivia (Plurinational State of) | both | 1163.11(932.86,1455.83) | 3668.20(2856.82,4659.24) | 2.15 | 39.18(31.51,48.42) | 45.13(35.49,56.77) | 0.39(0.32,0.46) |
| Bosnia and Herzegovina | both | 501.23(461.84,546.85) | 2209.32(1680.02,2725.47) | 3.41 | 14.25(13.12,15.56) | 37.00(28.27,45.56) | 4.36(3.66,5.06) |
| Botswana | both | 296.51(231.89,384.74) | 1036.00(767.75,1343.95) | 2.49 | 59.52(47.08,76.64) | 90.79(69.71,116.86) | 1.21(0.77,1.66) |
| Brazil | both | 25555.76(24247.70,26641.61) | 62882.63(57008.54,66255.46) | 1.46 | 32.07(30.04,33.57) | 27.62(24.96,29.18) | -0.49(-0.54,-0.44) |
| Brunei Darussalam | both | 84.17(72.71,99.58) | 162.56(144.07,184.12) | 0.93 | 109.86(95.08,128.37) | 72.67(64.74,81.87) | -0.94(-1.23,-0.65) |
| Bulgaria | both | 1886.90(1747.01,2009.51) | 2101.99(1698.15,2578.93) | 0.11 | 15.14(14.02,16.07) | 13.79(11.14,16.87) | -1.14(-1.53,-0.74) |
| Burkina Faso | both | 1228.40(1011.39,1482.06) | 2683.38(2190.36,3242.80) | 1.18 | 34.08(28.19,40.34) | 34.83(28.81,41.53) | -0.02(-0.12,0.07) |
| Burundi | both | 945.64(734.66,1251.14) | 1328.67(1031.57,1699.53) | 0.41 | 44.68(35.27,58.63) | 35.70(28.38,44.46) | -1.04(-1.15,-0.93) |
| Cambodia | both | 1285.28(997.89,1579.66) | 3351.67(2596.84,4088.25) | 1.61 | 31.36(25.04,37.89) | 31.77(24.81,38.54) | -0.06(-0.14,0.02) |
| Cameroon | both | 1323.70(1028.03,1661.56) | 4870.67(3741.11,6406.92) | 2.68 | 35.40(27.59,44.65) | 49.18(38.32,62.66) | 1.18(0.85,1.50) |
| Canada | both | 3874.93(3545.99,4112.05) | 6857.45(5961.80,7503.74) | 0.77 | 12.10(11.01,12.85) | 9.13(8.03,9.94) | -1.88(-2.53,-1.23) |
| Cabo Verde | both | 19.96(16.99,27.63) | 128.37(107.73,149.65) | 5.43 | 8.45(7.23,11.64) | 31.29(26.31,36.45) | 3.66(2.90,4.44) |
| Central African Republic | both | 509.17(412.84,617.36) | 924.98(693.43,1220.53) | 0.82 | 48.40(39.50,57.76) | 48.85(38.10,62.56) | 0.13(-0.02,0.28) |
| Chad | both | 601.12(472.40,736.70) | 1578.87(1229.87,2019.56) | 1.63 | 23.59(18.71,28.80) | 33.07(26.07,41.50) | 1.14(0.82,1.47) |
| Chile | both | 1440.94(1365.69,1502.72) | 3724.59(3346.17,4030.54) | 1.58 | 15.43(14.58,16.10) | 15.52(13.92,16.78) | -0.10(-0.29,0.10) |
| China | both | 64084.08(56364.69,73218.73) | 168388.12(143232.70,194030.08) | 1.63 | 8.63(7.66,9.79) | 9.17(7.87,10.51) | 0.34(0.08,0.60) |
| Colombia | both | 3193.73(3013.67,3335.33) | 6891.79(5445.90,8680.81) | 1.16 | 19.82(18.43,20.80) | 12.80(10.12,16.19) | -2.45(-2.91,-1.99) |
| Comoros | both | 73.35(47.10,92.13) | 152.45(125.51,189.47) | 1.08 | 37.40(26.05,46.36) | 35.14(29.41,43.40) | -0.36(-0.46,-0.26) |
| Congo | both | 532.07(437.04,635.68) | 1093.95(868.34,1376.14) | 1.06 | 57.15(46.97,68.47) | 51.40(42.02,62.50) | -0.50(-0.60,-0.41) |
| Costa Rica | both | 254.83(236.21,272.40) | 563.35(441.15,710.35) | 1.21 | 15.17(13.96,16.22) | 11.05(8.65,13.91) | -1.93(-2.44,-1.42) |
| C么te d'Ivoire | both | 1016.60(831.01,1207.20) | 3241.92(2526.77,4062.44) | 2.19 | 32.11(26.93,37.46) | 38.44(30.77,46.96) | 0.39(0.03,0.75) |
| Croatia | both | 717.50(666.22,771.14) | 1084.04(867.77,1319.45) | 0.51 | 11.88(11.03,12.72) | 11.29(9.06,13.76) | -0.67(-0.94,-0.40) |
| Cuba | both | 2142.51(2013.50,2246.45) | 1967.52(1600.66,2382.76) | -0.08 | 21.22(19.89,22.22) | 10.04(8.18,12.19) | -2.88(-3.64,-2.12) |
| Cyprus | both | 425.83(330.49,499.07) | 478.86(398.16,554.73) | 0.12 | 63.98(49.74,74.60) | 26.88(21.94,30.97) | -3.43(-3.62,-3.24) |
| Czechia | both | 1670.05(1545.72,1755.45) | 3229.36(2629.82,3864.25) | 0.93 | 12.09(11.17,12.71) | 14.37(11.72,17.22) | 2.50(1.57,3.44) |
| Democratic Republic of the Congo | both | 5276.47(4352.59,6396.23) | 10385.96(8251.38,12973.06) | 0.97 | 41.51(35.06,48.90) | 34.76(28.22,42.73) | -0.70(-0.74,-0.66) |
| Denmark | both | 771.85(723.10,822.30) | 1351.27(1207.35,1473.67) | 0.75 | 9.26(8.70,9.90) | 10.83(9.76,11.78) | 0.64(0.08,1.21) |
| Djibouti | both | 36.09(27.25,48.04) | 191.99(148.33,258.03) | 4.32 | 33.44(26.08,43.18) | 41.93(33.91,53.52) | 0.83(0.75,0.90) |
| Dominica | both | 48.85(43.54,54.31) | 55.32(45.85,65.96) | 0.13 | 65.82(58.98,72.96) | 60.68(50.35,72.44) | -0.51(-0.63,-0.38) |
| Dominican Republic | both | 736.46(652.70,837.67) | 2756.85(2111.90,3593.02) | 2.74 | 21.17(18.68,23.99) | 30.48(23.47,39.26) | 1.75(1.56,1.93) |
| Ecuador | both | 1082.44(1017.96,1173.83) | 4830.30(3860.85,6087.27) | 3.46 | 21.80(20.39,23.93) | 34.47(27.79,43.18) | 1.57(1.29,1.86) |
| Egypt | both | 6009.57(5568.81,6528.96) | 19232.81(14457.09,25267.10) | 2.20 | 23.30(21.45,25.35) | 33.75(26.03,43.78) | 1.63(1.50,1.76) |
| El Salvador | both | 496.73(456.10,561.22) | 2182.24(1666.34,2794.28) | 3.39 | 17.21(15.73,19.60) | 36.31(27.73,46.66) | 2.55(2.23,2.87) |
| Equatorial Guinea | both | 77.62(57.86,98.18) | 210.40(151.11,290.01) | 1.71 | 43.39(32.85,54.12) | 54.33(40.28,72.21) | 1.02(0.88,1.15) |
| Eritrea | both | 319.88(225.77,445.59) | 957.72(720.38,1277.16) | 1.99 | 37.29(26.50,52.38) | 44.67(34.34,58.33) | 0.52(0.34,0.71) |
| Estonia | both | 67.30(61.47,75.99) | 123.12(98.10,153.19) | 0.83 | 3.32(3.03,3.75) | 4.47(3.52,5.60) | 0.89(-0.02,1.81) |
| Ethiopia | both | 9796.88(7970.79,12668.56) | 11852.92(10174.23,13796.72) | 0.21 | 55.12(45.21,68.95) | 34.46(29.56,39.86) | -1.85(-1.98,-1.72) |
| Micronesia (Federated States of) | both | 40.59(31.80,50.89) | 107.11(77.18,150.65) | 1.64 | 94.47(75.05,117.11) | 169.13(126.79,226.11) | 1.91(1.58,2.25) |
| Fiji | both | 508.23(413.22,651.51) | 1698.42(1358.98,2085.70) | 2.34 | 158.10(129.62,203.35) | 257.38(210.35,309.25) | 1.26(0.82,1.71) |
| Finland | both | 459.57(421.77,502.03) | 446.42(390.49,491.27) | -0.03 | 6.44(5.87,7.04) | 3.34(2.95,3.65) | -2.62(-2.84,-2.39) |
| France | both | 6841.42(6217.79,7390.04) | 12220.06(10361.50,13536.54) | 0.79 | 7.76(7.07,8.35) | 7.13(6.25,7.79) | -0.34(-0.93,0.25) |
| Gabon | both | 309.66(236.05,414.29) | 587.78(457.84,731.17) | 0.90 | 60.81(46.63,81.00) | 65.24(51.29,79.54) | 0.20(0.01,0.40) |
| Georgia | both | 595.09(543.26,654.23) | 1254.28(1056.19,1452.42) | 1.11 | 9.69(8.87,10.64) | 20.46(17.20,23.78) | 2.55(0.80,4.34) |
| Germany | both | 20468.50(18929.96,21677.01) | 21707.14(19167.65,23698.79) | 0.06 | 15.37(14.17,16.27) | 9.65(8.63,10.46) | -2.06(-2.32,-1.81) |
| Ghana | both | 1500.60(1220.74,1830.28) | 5855.99(4786.62,7219.34) | 2.90 | 28.85(24.08,34.61) | 42.66(35.37,51.07) | 1.62(1.24,1.99) |
| Greece | both | 1133.81(1061.70,1192.44) | 1349.53(1203.99,1464.60) | 0.19 | 7.50(6.97,7.89) | 5.00(4.54,5.40) | -0.72(-1.15,-0.29) |
| Greenland | both | 4.58(3.95,5.30) | 5.65(4.44,6.82) | 0.24 | 15.64(13.53,18.02) | 9.00(7.21,10.78) | -2.45(-2.70,-2.19) |
| Grenada | both | 51.42(46.58,56.30) | 72.54(65.56,79.84) | 0.41 | 67.69(61.36,74.15) | 71.43(64.58,78.12) | 0.22(-0.01,0.46) |
| Guam | both | 23.99(20.61,27.15) | 44.72(37.57,54.17) | 0.86 | 39.83(33.60,45.49) | 23.70(19.89,28.63) | -2.10(-2.46,-1.74) |
| Guatemala | both | 544.37(480.60,605.53) | 6346.78(5059.47,7860.71) | 10.66 | 16.56(14.73,18.28) | 60.11(48.39,74.03) | 3.66(2.94,4.39) |
| Guinea | both | 820.41(666.10,987.34) | 1849.69(1443.04,2288.56) | 1.25 | 27.61(22.33,33.18) | 37.70(29.50,46.11) | 1.19(0.98,1.40) |
| Guinea-Bissau | both | 145.53(111.86,190.59) | 294.73(221.67,377.24) | 1.03 | 40.24(31.62,51.71) | 48.59(37.30,60.97) | 0.63(0.41,0.85) |
| Guyana | both | 284.60(250.99,317.45) | 538.61(424.26,673.31) | 0.89 | 79.81(70.80,88.69) | 92.84(74.49,114.71) | 0.17(-0.31,0.66) |
| Haiti | both | 2358.68(1848.12,2926.68) | 3826.54(2678.31,5441.17) | 0.62 | 78.52(61.60,97.31) | 61.63(43.79,86.98) | -0.70(-0.79,-0.62) |
| Honduras | both | 245.60(200.52,283.46) | 1066.56(863.21,1338.12) | 3.34 | 11.94(9.49,13.75) | 18.70(15.30,22.90) | 1.68(1.49,1.87) |
| Hungary | both | 1709.46(1615.37,1789.15) | 2392.41(1985.11,2876.03) | 0.40 | 11.58(10.93,12.12) | 11.68(9.66,14.06) | 0.32(-0.02,0.65) |
| Iceland | both | 14.33(12.88,15.78) | 24.44(20.64,27.58) | 0.71 | 4.77(4.30,5.23) | 3.92(3.36,4.45) | -1.05(-1.23,-0.87) |
| India | both | 72125.11(61628.66,85897.20) | 258927.15(224419.59,300778.85) | 2.59 | 21.13(18.07,25.06) | 26.19(22.76,30.27) | 0.69(0.46,0.93) |
| Indonesia | both | 29124.94(25555.76,32931.41) | 98679.39(82290.71,113403.78) | 2.39 | 31.02(27.42,34.94) | 48.26(40.52,54.32) | 1.82(1.70,1.95) |
| Iran (Islamic Republic of) | both | 2413.53(2110.13,2761.37) | 14190.85(12014.43,15320.14) | 4.88 | 12.46(10.69,14.44) | 21.98(18.39,23.79) | 2.31(2.05,2.57) |
| Iraq | both | 3584.06(2884.02,4284.13) | 9037.68(7191.06,10967.51) | 1.52 | 49.22(39.45,58.61) | 45.78(37.07,54.43) | -0.39(-0.47,-0.31) |
| Ireland | both | 403.47(379.02,425.68) | 479.64(417.85,527.36) | 0.19 | 9.91(9.28,10.45) | 6.10(5.33,6.71) | -1.93(-2.10,-1.76) |
| Israel | both | 796.48(743.60,838.69) | 2593.31(2272.11,2811.74) | 2.26 | 17.09(15.82,18.00) | 20.62(18.22,22.30) | -0.53(-1.55,0.50) |
| Italy | both | 17472.08(16491.60,18095.27) | 21118.97(18048.11,22727.78) | 0.21 | 19.03(17.80,19.75) | 11.92(10.39,12.71) | -1.66(-1.73,-1.59) |
| Jamaica | both | 1104.87(1028.82,1168.54) | 2341.83(1925.43,2835.13) | 1.12 | 60.97(56.89,64.44) | 75.51(61.79,91.90) | 0.46(0.14,0.77) |
| Japan | both | 9867.89(9201.90,10238.69) | 8539.45(6915.22,9450.55) | -0.13 | 6.06(5.61,6.31) | 1.96(1.68,2.13) | -3.84(-4.30,-3.37) |
| Jordan | both | 666.38(561.90,782.56) | 2000.48(1694.01,2395.69) | 2.00 | 63.93(53.77,74.93) | 40.22(34.10,47.54) | -1.98(-2.40,-1.55) |
| Kazakhstan | both | 824.49(774.95,875.32) | 2437.15(2121.83,2786.92) | 1.96 | 6.75(6.37,7.18) | 15.44(13.46,17.62) | 1.58(0.77,2.40) |
| Kenya | both | 1883.48(1668.51,2166.07) | 5737.98(4775.80,6900.97) | 2.05 | 27.26(24.24,31.16) | 31.89(26.88,37.36) | 0.59(0.43,0.75) |
| Kiribati | both | 53.49(43.46,65.07) | 133.78(101.20,169.83) | 1.50 | 146.43(120.11,175.84) | 203.99(158.28,252.68) | 0.78(0.37,1.20) |
| Kuwait | both | 129.50(117.03,144.51) | 354.39(294.64,423.47) | 1.74 | 28.75(25.44,32.25) | 18.47(15.26,22.20) | -1.79(-2.49,-1.09) |
| Kyrgyzstan | both | 182.91(169.82,196.45) | 294.10(256.24,336.27) | 0.61 | 6.07(5.64,6.53) | 6.56(5.71,7.48) | -0.83(-1.29,-0.37) |
| Lao People's Democratic Republic | both | 728.54(542.60,974.21) | 1439.26(1121.78,1826.45) | 0.98 | 37.95(28.87,48.98) | 37.54(29.42,47.25) | -0.24(-0.31,-0.16) |
| Latvia | both | 163.77(153.49,176.14) | 322.37(269.69,385.56) | 0.97 | 4.61(4.31,4.97) | 7.69(6.44,9.20) | 1.83(1.35,2.31) |
| Lebanon | both | 399.92(345.20,481.00) | 820.83(587.80,1051.69) | 1.05 | 20.39(17.53,24.43) | 15.92(11.38,20.38) | -0.63(-0.87,-0.40) |
| Lesotho | both | 449.11(368.84,541.01) | 1235.61(902.85,1611.77) | 1.75 | 50.25(41.33,60.02) | 110.91(82.55,143.05) | 3.55(3.16,3.94) |
| Liberia | both | 286.79(238.22,339.02) | 587.37(441.15,764.82) | 1.05 | 29.31(24.80,34.47) | 34.56(26.37,44.39) | 0.62(0.46,0.78) |
| Libya | both | 238.05(174.48,299.51) | 834.20(604.58,1106.34) | 2.50 | 14.22(10.45,17.97) | 18.33(13.35,24.07) | 1.36(1.12,1.60) |
| Lithuania | both | 158.15(147.60,171.96) | 217.55(176.70,264.46) | 0.38 | 3.52(3.29,3.83) | 3.71(3.02,4.52) | -0.06(-0.31,0.19) |
| Luxembourg | both | 54.75(50.20,59.09) | 64.33(54.21,74.64) | 0.17 | 10.17(9.26,10.99) | 5.79(4.94,6.70) | -2.03(-2.13,-1.92) |
| North Macedonia | both | 329.78(293.06,364.04) | 1001.17(798.95,1229.71) | 2.04 | 19.37(17.18,21.34) | 33.57(26.96,40.86) | 2.25(1.77,2.73) |
| Madagascar | both | 1349.74(1111.11,1609.33) | 2600.68(1974.68,3357.32) | 0.93 | 30.21(24.93,36.00) | 29.97(23.18,38.39) | -0.11(-0.24,0.02) |
| Malawi | both | 1287.22(1092.31,1497.21) | 2285.26(1891.48,2735.33) | 0.78 | 40.11(34.10,46.43) | 37.17(31.34,43.67) | -0.41(-0.63,-0.19) |
| Malaysia | both | 2238.41(2015.37,2458.27) | 3232.92(2548.90,4249.34) | 0.44 | 26.57(23.76,29.17) | 13.28(10.54,17.49) | -3.32(-3.88,-2.75) |
| Maldives | both | 24.18(19.31,31.93) | 48.41(40.14,57.88) | 1.00 | 32.41(26.48,40.40) | 18.96(15.58,22.57) | -2.34(-2.71,-1.97) |
| Mali | both | 1008.21(827.56,1214.43) | 2527.69(2024.03,3107.66) | 1.51 | 28.57(23.39,34.48) | 34.60(27.71,41.57) | 0.61(0.48,0.75) |
| Malta | both | 93.67(86.19,100.81) | 119.20(101.98,135.47) | 0.27 | 23.12(21.21,24.89) | 11.80(10.15,13.36) | -2.20(-2.43,-1.98) |
| Marshall Islands | both | 12.22(10.26,14.53) | 34.60(26.04,46.00) | 1.83 | 82.83(68.84,99.39) | 112.90(86.87,145.94) | 1.01(0.78,1.23) |
| Mauritania | both | 301.18(249.66,362.68) | 596.19(458.13,762.18) | 0.98 | 33.77(28.39,40.19) | 33.07(25.81,41.58) | -0.12(-0.23,0.00) |
| Mauritius | both | 317.94(298.44,336.85) | 1819.15(1500.84,2202.09) | 4.72 | 44.67(42.03,47.40) | 107.25(88.81,129.36) | 5.09(4.07,6.11) |
| Mexico | both | 25669.83(24585.16,26411.96) | 72036.04(62171.37,81851.05) | 1.81 | 63.89(60.73,65.91) | 63.95(55.36,72.65) | -0.21(-0.39,-0.02) |
| Republic of Moldova | both | 289.69(275.39,305.36) | 269.76(232.50,309.63) | -0.07 | 6.57(6.24,6.92) | 4.61(3.97,5.30) | -2.10(-2.50,-1.70) |
| Mongolia | both | 44.55(36.03,55.59) | 113.36(85.95,150.90) | 1.54 | 4.41(3.61,5.37) | 5.04(3.91,6.45) | 0.15(-0.15,0.46) |
| Montenegro | both | 68.73(59.49,77.74) | 134.10(111.93,160.93) | 0.95 | 11.66(10.07,13.13) | 13.73(11.51,16.38) | 0.72(0.54,0.89) |
| Morocco | both | 1561.82(1253.84,2195.73) | 6195.54(4687.10,7778.60) | 2.97 | 13.09(10.39,18.56) | 22.36(17.15,28.34) | 2.17(2.00,2.33) |
| Mozambique | both | 1823.27(1510.37,2160.60) | 4245.12(3351.54,5360.56) | 1.33 | 36.59(30.51,42.87) | 45.46(36.69,56.67) | 1.08(0.93,1.22) |
| Myanmar | both | 11490.47(8680.05,14814.56) | 20862.11(17962.12,24264.62) | 0.82 | 53.11(41.29,66.64) | 50.07(43.65,57.70) | -0.31(-0.35,-0.26) |
| Namibia | both | 375.81(313.24,437.97) | 767.21(605.29,967.40) | 1.04 | 56.81(47.81,66.26) | 61.70(48.98,77.66) | -0.06(-0.48,0.36) |
| Nepal | both | 802.08(638.98,1004.66) | 3245.46(2563.31,3952.16) | 3.05 | 11.09(8.83,13.74) | 18.66(14.66,22.57) | 2.00(1.72,2.29) |
| Netherlands | both | 3356.79(3062.35,3568.31) | 3191.91(2833.07,3495.06) | -0.05 | 16.56(15.08,17.64) | 8.51(7.61,9.29) | -2.76(-3.17,-2.34) |
| New Zealand | both | 398.35(371.08,426.62) | 655.69(588.59,707.81) | 0.65 | 10.27(9.57,10.98) | 7.92(7.15,8.51) | -1.82(-2.32,-1.31) |
| Nicaragua | both | 408.98(375.40,449.33) | 1867.56(1563.60,2202.16) | 3.57 | 28.46(25.97,31.58) | 46.85(39.65,54.36) | 1.32(0.92,1.73) |
| Niger | both | 570.58(445.28,704.28) | 1926.08(1486.32,2414.67) | 2.38 | 24.91(19.58,30.58) | 30.68(24.12,37.53) | 0.74(0.57,0.91) |
| Nigeria | both | 11124.56(9093.55,13521.37) | 22796.73(18033.30,28412.18) | 1.05 | 29.76(24.64,35.59) | 32.82(26.57,39.98) | 0.39(0.25,0.52) |
| Democratic People's Republic of Korea | both | 1930.48(1515.37,2447.73) | 3854.87(3142.96,4724.87) | 1.00 | 13.37(10.58,16.90) | 12.38(10.13,15.17) | -0.23(-0.36,-0.10) |
| Northern Mariana Islands | both | 7.25(6.06,8.85) | 26.57(22.22,31.03) | 2.67 | 50.41(43.10,60.40) | 58.47(50.02,67.70) | 0.81(0.32,1.30) |
| Norway | both | 493.23(447.49,519.50) | 622.43(544.77,669.27) | 0.26 | 6.75(6.15,7.10) | 5.65(5.01,6.05) | -0.72(-1.09,-0.34) |
| Oman | both | 233.12(178.75,300.71) | 641.93(546.52,742.62) | 1.75 | 44.74(34.25,57.20) | 58.33(50.42,66.51) | 1.31(1.12,1.50) |
| Pakistan | both | 11829.30(7884.87,14518.35) | 44961.12(35202.56,55336.78) | 2.80 | 23.19(15.38,28.42) | 49.55(38.74,60.79) | 2.75(2.47,3.03) |
| Palestine | both | 412.51(327.18,511.16) | 1257.32(1081.19,1443.47) | 2.05 | 53.89(42.96,66.43) | 68.84(59.52,78.69) | 0.82(0.39,1.25) |
| Panama | both | 260.70(237.69,279.50) | 1179.92(927.39,1471.77) | 3.53 | 18.40(16.73,19.73) | 28.24(22.11,35.29) | 1.15(0.85,1.45) |
| Papua New Guinea | both | 1309.55(1020.34,1659.10) | 4498.76(3464.62,5725.78) | 2.44 | 77.11(60.98,97.47) | 104.02(82.47,130.12) | 0.95(0.77,1.12) |
| Paraguay | both | 471.65(413.58,529.17) | 2390.18(1853.32,3009.44) | 4.07 | 22.82(19.88,25.64) | 45.36(35.24,57.31) | 2.52(2.04,3.00) |
| Peru | both | 1493.75(1295.14,1729.29) | 4584.98(3448.91,6033.16) | 2.07 | 13.25(11.49,15.35) | 14.38(10.80,18.93) | 0.45(0.15,0.74) |
| Philippines | both | 8528.58(7703.86,9403.25) | 26038.72(21645.03,31288.59) | 2.05 | 35.53(31.90,39.08) | 36.95(31.06,43.75) | 0.10(-0.08,0.28) |
| Poland | both | 5317.17(5059.63,5582.02) | 7607.63(6425.77,8901.05) | 0.43 | 12.09(11.48,12.69) | 10.41(8.80,12.20) | -0.22(-0.57,0.13) |
| Portugal | both | 2777.60(2635.45,2911.28) | 4194.20(3616.99,4599.41) | 0.51 | 19.92(18.70,20.87) | 14.49(12.73,15.76) | -1.69(-2.04,-1.34) |
| Puerto Rico | both | 1570.38(1454.82,1652.09) | 3176.96(2484.71,3971.45) | 1.02 | 44.39(40.87,46.72) | 40.73(31.98,51.16) | -0.73(-1.04,-0.42) |
| Qatar | both | 62.07(51.96,73.89) | 335.52(253.21,437.97) | 4.41 | 111.16(94.36,130.89) | 122.07(98.85,151.52) | 0.75(0.22,1.29) |
| Romania | both | 1809.55(1723.46,1909.87) | 2302.53(1896.62,2712.32) | 0.27 | 6.51(6.18,6.86) | 5.95(4.87,7.02) | -0.33(-0.61,-0.04) |
| Russian Federation | both | 7484.52(7066.31,7755.26) | 17838.35(15440.27,20296.41) | 1.38 | 4.24(4.01,4.40) | 7.45(6.45,8.47) | 1.60(0.81,2.40) |
| Rwanda | both | 1262.12(986.92,1608.77) | 1763.24(1345.47,2302.58) | 0.40 | 49.40(38.94,62.61) | 36.51(28.00,47.32) | -1.77(-2.06,-1.47) |
| Saint Lucia | both | 69.20(64.12,73.72) | 125.68(107.29,147.37) | 0.82 | 83.86(77.50,89.43) | 60.88(51.74,71.25) | -2.04(-2.40,-1.67) |
| Saint Vincent and the Grenadines | both | 59.34(54.02,64.08) | 103.45(91.28,118.26) | 0.74 | 85.81(78.33,92.62) | 80.43(71.06,91.55) | -0.68(-0.93,-0.42) |
| Samoa | both | 57.99(46.14,72.60) | 121.29(98.01,153.39) | 1.09 | 71.66(58.02,90.09) | 89.18(72.69,111.28) | 0.65(0.39,0.91) |
| Sao Tome and Principe | both | 6.43(5.27,7.56) | 13.08(10.69,15.50) | 1.03 | 11.24(9.44,13.00) | 14.79(12.18,17.33) | 0.90(0.80,0.99) |
| Saudi Arabia | both | 1267.80(949.62,1683.49) | 2796.60(2211.10,3439.15) | 1.21 | 25.17(19.16,33.07) | 19.51(16.12,23.69) | -1.61(-1.97,-1.26) |
| Senegal | both | 792.63(635.44,951.16) | 2430.03(1877.38,3046.88) | 2.07 | 28.54(23.22,34.15) | 37.86(29.88,46.79) | 1.03(0.83,1.24) |
| Serbia | both | 1942.54(1601.82,2200.63) | 3514.24(2870.86,4271.31) | 0.81 | 18.81(15.58,21.31) | 21.40(17.62,25.90) | 0.66(0.44,0.89) |
| Seychelles | both | 7.89(6.97,8.90) | 24.22(21.18,27.52) | 2.07 | 14.10(12.47,15.88) | 24.38(21.16,27.70) | 1.83(1.64,2.03) |
| Sierra Leone | both | 413.37(334.51,499.38) | 1005.59(774.53,1290.77) | 1.43 | 23.94(19.58,28.78) | 32.67(25.64,41.12) | 1.27(1.02,1.52) |
| Singapore | both | 362.36(342.31,380.20) | 175.00(153.34,192.63) | -0.52 | 18.22(17.01,19.17) | 2.39(2.08,2.64) | -7.24(-8.40,-6.07) |
| Slovakia | both | 756.82(694.34,826.41) | 766.81(602.98,955.46) | 0.01 | 12.63(11.58,13.73) | 8.20(6.47,10.20) | -1.39(-1.53,-1.24) |
| Slovenia | both | 246.54(194.68,307.70) | 345.61(270.11,434.78) | 0.40 | 10.47(8.27,13.00) | 7.06(5.56,8.93) | -3.57(-4.51,-2.63) |
| Solomon Islands | both | 107.56(78.04,151.38) | 417.28(336.49,513.12) | 2.88 | 76.66(56.50,108.40) | 133.60(110.61,162.80) | 1.89(1.68,2.10) |
| Somalia | both | 924.56(692.85,1215.01) | 2307.57(1743.41,2990.35) | 1.50 | 43.08(33.27,55.33) | 42.70(33.33,54.08) | 0.15(0.09,0.21) |
| South Africa | both | 8085.51(6925.42,9115.11) | 26872.43(24567.83,29032.10) | 2.32 | 42.10(35.80,47.60) | 67.67(61.96,73.06) | 2.01(1.48,2.54) |
| Republic of Korea | both | 4660.91(4404.49,5759.56) | 12612.35(10966.47,14249.55) | 1.71 | 17.00(15.87,23.74) | 14.70(12.70,16.64) | -1.36(-2.27,-0.45) |
| South Sudan | both | 723.73(564.27,917.88) | 1044.72(764.96,1372.81) | 0.44 | 35.03(27.66,44.25) | 33.69(25.40,43.15) | -0.10(-0.13,-0.08) |
| Spain | both | 9406.76(8591.33,9962.58) | 10011.22(8469.30,11101.94) | 0.06 | 17.19(15.58,18.23) | 7.99(6.86,8.75) | -2.89(-3.06,-2.73) |
| Sri Lanka | both | 2247.70(2013.10,2509.94) | 12739.55(9763.37,16396.36) | 4.67 | 25.06(22.34,27.83) | 57.05(42.95,73.14) | 3.66(3.11,4.21) |
| Sudan | both | 957.17(693.00,1333.50) | 2548.76(1723.13,3551.57) | 1.66 | 11.51(8.44,16.41) | 15.65(10.59,21.73) | 1.36(1.17,1.56) |
| Suriname | both | 89.59(81.49,97.09) | 257.86(216.06,306.88) | 1.88 | 36.05(32.80,39.09) | 44.48(37.36,52.79) | 0.70(0.39,1.01) |
| Eswatini | both | 210.02(164.78,256.30) | 668.67(481.93,896.59) | 2.18 | 83.27(65.56,100.86) | 134.58(97.47,177.28) | 2.07(1.42,2.72) |
| Sweden | both | 1355.24(1223.21,1457.38) | 1982.25(1738.36,2153.70) | 0.46 | 8.42(7.64,9.02) | 7.93(7.03,8.57) | -0.25(-0.47,-0.04) |
| Switzerland | both | 1508.88(1373.86,1603.81) | 1369.90(1156.95,1510.46) | -0.09 | 13.42(12.18,14.27) | 6.42(5.51,7.04) | -3.25(-3.57,-2.94) |
| Syrian Arab Republic | both | 872.69(683.44,1088.76) | 1598.62(1216.29,2139.01) | 0.83 | 18.67(14.40,23.23) | 15.89(12.31,20.76) | -1.27(-1.62,-0.92) |
| Taiwan (Province of China) | both | 4482.26(4248.80,4669.15) | 10630.69(8440.07,13386.45) | 1.37 | 31.40(29.54,32.81) | 26.54(21.06,33.54) | -1.72(-2.34,-1.10) |
| Tajikistan | both | 327.02(298.58,362.25) | 1478.46(1182.45,1845.68) | 3.52 | 11.63(10.64,12.83) | 31.28(25.16,38.15) | 3.89(3.52,4.27) |
| United Republic of Tanzania | both | 3217.36(2735.47,3740.36) | 7621.40(6127.39,9169.60) | 1.37 | 35.15(29.94,40.43) | 36.55(29.88,43.82) | 0.15(0.12,0.19) |
| Thailand | both | 8086.19(6936.51,9388.49) | 18337.97(13631.88,23776.23) | 1.27 | 24.90(21.30,29.01) | 18.24(13.61,23.58) | -1.86(-2.25,-1.48) |
| Bahamas | both | 69.21(63.18,75.55) | 136.90(114.46,165.68) | 0.98 | 48.47(44.02,52.88) | 38.03(31.76,45.64) | -1.31(-1.52,-1.10) |
| Gambia | both | 69.92(53.36,88.20) | 307.30(230.81,388.77) | 3.40 | 24.21(19.13,30.12) | 36.95(28.00,46.18) | 1.32(1.09,1.55) |
| Timor-Leste | both | 52.84(40.53,68.52) | 175.99(134.78,222.93) | 2.33 | 21.87(17.08,27.95) | 24.41(18.85,30.58) | 0.38(0.15,0.60) |
| Togo | both | 266.10(217.38,322.02) | 962.69(728.01,1248.42) | 2.62 | 25.78(21.26,30.99) | 32.84(25.44,41.92) | 0.78(0.55,1.02) |
| Tonga | both | 44.32(37.06,52.11) | 80.75(64.03,99.93) | 0.82 | 86.25(72.23,100.87) | 104.61(83.04,129.12) | 0.83(0.62,1.04) |
| Trinidad and Tobago | both | 974.20(930.08,1021.38) | 1789.86(1373.24,2286.39) | 0.84 | 121.59(115.74,127.41) | 97.78(75.34,124.14) | -1.24(-1.47,-1.01) |
| Tunisia | both | 459.45(370.64,615.64) | 1722.47(1236.94,2321.96) | 2.75 | 10.90(8.73,15.01) | 14.84(10.71,19.94) | 1.26(1.16,1.37) |
| Turkey | both | 12931.09(11085.64,14894.00) | 18831.44(15145.22,22948.73) | 0.46 | 40.35(34.56,47.01) | 22.73(18.27,27.61) | -1.65(-1.94,-1.37) |
| Turkmenistan | both | 187.22(173.02,198.92) | 660.04(521.92,834.42) | 2.53 | 9.72(9.02,10.29) | 16.38(13.11,20.61) | 1.08(0.68,1.48) |
| Uganda | both | 2071.72(1597.44,2712.72) | 4631.94(3564.54,5896.92) | 1.24 | 37.74(29.54,48.97) | 39.84(31.14,50.32) | -0.12(-0.32,0.09) |
| Ukraine | both | 2613.93(2488.07,2731.22) | 2426.64(2073.34,2829.91) | -0.07 | 3.71(3.52,3.88) | 3.25(2.77,3.78) | -1.63(-2.02,-1.23) |
| United Arab Emirates | both | 175.05(136.66,216.60) | 1231.99(869.39,1662.50) | 6.04 | 75.28(58.30,92.75) | 55.19(41.41,70.71) | -1.02(-1.75,-0.28) |
| United Kingdom | both | 7397.16(6914.03,7654.14) | 6069.50(5423.68,6433.95) | -0.18 | 7.90(7.35,8.19) | 4.29(3.88,4.52) | -2.42(-2.61,-2.22) |
| United States of America | both | 43919.16(40925.86,45553.32) | 73408.81(67731.01,76758.58) | 0.67 | 13.40(12.52,13.88) | 12.67(11.80,13.23) | -0.85(-1.32,-0.37) |
| Uruguay | both | 663.04(622.62,697.08) | 866.41(779.69,939.38) | 0.31 | 16.77(15.71,17.65) | 14.63(13.36,15.78) | -0.40(-0.53,-0.26) |
| Uzbekistan | both | 974.38(894.46,1055.69) | 7190.63(5988.68,8488.36) | 6.38 | 8.60(7.91,9.29) | 35.30(29.96,41.18) | 4.73(4.04,5.43) |
| Vanuatu | both | 25.75(18.59,37.13) | 122.58(91.53,165.02) | 3.76 | 43.76(32.14,62.09) | 77.53(58.32,103.12) | 2.11(1.98,2.23) |
| Venezuela (Bolivarian Republic of) | both | 2952.90(2791.56,3097.47) | 10584.92(8191.82,13562.49) | 2.58 | 32.72(30.74,34.39) | 37.83(29.39,48.28) | 0.27(0.10,0.44) |
| Viet nam | both | 11327.25(9050.49,13676.77) | 27732.87(22236.06,33468.78) | 1.45 | 30.90(24.59,37.24) | 34.50(27.85,41.49) | 0.55(0.35,0.75) |
| Virginia | both | 845.72(782.79,902.78) | 1807.95(1501.84,2120.81) | 1.14 | 12.06(11.15,12.88) | 12.53(10.40,14.70) | -0.58(-1.09,-0.06) |
| Yemen | both | 484.90(332.52,719.62) | 1620.08(1137.50,2302.38) | 2.34 | 11.66(8.14,17.85) | 14.18(10.20,19.83) | 0.93(0.78,1.08) |
| Zambia | both | 1018.57(856.63,1193.00) | 2360.52(1906.99,2906.41) | 1.32 | 42.23(35.76,49.29) | 42.64(35.37,51.42) | -0.26(-0.53,0.01) |
| Zimbabwe | both | 1208.93(1058.49,1373.18) | 3341.41(2603.91,4126.62) | 1.76 | 34.96(30.42,39.78) | 55.64(43.86,68.72) | 2.19(1.82,2.56) |
| Monaco | both | 2.44(1.94,2.98) | 3.51(2.87,4.14) | 0.44 | 3.03(2.43,3.71) | 3.10(2.51,3.68) | 0.24(0.07,0.41) |
| San Marino | both | 2.31(1.94,2.75) | 4.52(3.10,6.12) | 0.95 | 7.05(5.91,8.33) | 5.79(3.93,7.99) | -0.48(-0.59,-0.37) |
| Saint Kitts and Nevis | both | 25.52(23.31,27.98) | 31.22(26.68,36.30) | 0.22 | 69.56(63.79,75.48) | 53.86(46.83,62.33) | -0.80(-0.97,-0.63) |
| Cook Islands | both | 14.14(12.30,16.51) | 26.77(22.54,31.62) | 0.89 | 121.38(105.58,141.63) | 110.42(92.78,130.43) | -0.51(-0.70,-0.33) |
| Nauru | both | 3.76(2.89,5.16) | 5.90(4.42,8.76) | 0.57 | 112.79(90.04,144.75) | 162.80(126.94,227.02) | 1.05(0.77,1.34) |
| Niue | both | 2.00(1.64,2.50) | 2.62(2.05,3.48) | 0.31 | 88.31(72.09,110.56) | 122.00(95.17,162.49) | 0.95(0.68,1.23) |
| Palau | both | 7.06(5.69,8.94) | 22.84(17.43,28.94) | 2.23 | 79.06(63.99,98.36) | 119.21(92.22,148.24) | 1.37(1.12,1.63) |
| Tokelau | both | 1.00(0.80,1.24) | 1.23(0.95,1.59) | 0.23 | 75.58(60.64,92.84) | 96.45(74.96,123.15) | 0.80(0.57,1.03) |
| Tuvalu | both | 5.63(4.54,6.96) | 11.00(8.33,14.84) | 0.95 | 88.49(72.62,107.63) | 115.83(88.37,155.15) | 0.83(0.60,1.06) |
| Afghanistan | male | 630.18(437.45,909.43) | 1063.03(730.42,1525.18) | 0.69 | 18.31(13.10,26.01) | 21.09(14.91,29.41) | 0.78(0.66,0.91) |
| Albania | male | 36.21(30.42,41.64) | 72.95(51.75,99.69) | 1.01 | 4.40(3.77,5.04) | 3.64(2.61,4.91) | -1.29(-1.85,-0.72) |
| Algeria | male | 654.05(481.19,901.65) | 2090.93(1529.74,2710.34) | 2.20 | 14.44(10.60,19.66) | 14.60(10.85,18.64) | 0.35(0.05,0.66) |
| American Samoa | male | 8.98(7.63,10.67) | 21.63(18.72,24.71) | 1.41 | 89.37(76.13,105.83) | 102.51(89.35,115.61) | 0.44(0.00,0.88) |
| Andorra | male | 2.15(1.56,3.00) | 5.15(4.06,6.43) | 1.40 | 9.72(7.29,12.98) | 7.46(5.87,9.28) | -0.91(-0.96,-0.87) |
| Angola | male | 912.06(668.54,1183.17) | 2246.09(1779.39,2818.74) | 1.46 | 56.00(41.11,70.22) | 57.27(47.30,70.02) | 0.07(-0.03,0.16) |
| Antigua and Barbuda | male | 13.57(12.14,14.90) | 23.16(19.26,27.26) | 0.71 | 61.22(54.95,67.08) | 54.82(46.17,64.25) | -0.54(-0.88,-0.19) |
| Argentina | male | 3179.06(2992.16,3355.24) | 5282.73(4804.10,5778.95) | 0.66 | 23.74(22.38,25.06) | 22.98(20.94,25.12) | -0.72(-1.06,-0.38) |
| Armenia | male | 160.81(149.69,172.77) | 471.45(391.27,558.00) | 1.93 | 15.02(14.00,16.15) | 27.25(22.50,32.03) | 1.17(0.54,1.79) |
| Australia | male | 1013.59(951.65,1073.94) | 2215.96(1971.74,2434.21) | 1.19 | 13.13(12.30,13.88) | 11.08(9.90,12.16) | -1.02(-1.36,-0.69) |
| Austria | male | 529.96(498.97,559.20) | 1104.40(998.65,1202.30) | 1.08 | 12.27(11.53,12.93) | 13.56(12.30,14.72) | 0.76(0.23,1.30) |
| Azerbaijan | male | 224.91(200.46,250.58) | 876.64(676.13,1137.44) | 2.90 | 11.17(10.02,12.36) | 22.85(17.75,29.11) | 1.97(1.60,2.35) |
| Bahrain | male | 53.42(43.15,66.32) | 407.49(316.67,527.19) | 6.63 | 82.23(67.21,101.12) | 124.53(99.58,156.50) | 1.80(1.24,2.36) |
| Bangladesh | male | 6065.63(5043.02,7107.44) | 14615.20(11370.36,18339.47) | 1.41 | 28.65(23.65,33.72) | 26.02(20.32,32.38) | -0.75(-1.50,0.01) |
| Barbados | male | 71.72(64.82,78.17) | 121.56(101.05,143.99) | 0.69 | 57.61(52.00,62.58) | 56.30(46.85,66.78) | -1.05(-1.42,-0.67) |
| Belarus | male | 181.47(168.67,193.35) | 144.70(114.15,184.60) | -0.20 | 4.04(3.76,4.30) | 2.69(2.15,3.39) | -2.62(-3.00,-2.22) |
| Belgium | male | 539.90(505.18,574.24) | 721.16(647.58,791.00) | 0.34 | 9.41(8.77,9.99) | 6.82(6.15,7.47) | -1.37(-1.58,-1.15) |
| Belize | male | 13.80(12.21,15.59) | 68.43(58.45,79.62) | 3.96 | 31.13(27.57,35.07) | 51.51(43.84,59.95) | 1.43(0.70,2.16) |
| Benin | male | 251.48(202.63,306.37) | 646.30(461.63,879.63) | 1.57 | 29.09(23.63,35.04) | 34.58(25.22,45.52) | 0.54(0.23,0.84) |
| Bermuda | male | 8.21(7.36,9.12) | 12.31(10.32,14.56) | 0.50 | 33.35(30.04,37.08) | 21.73(18.30,25.59) | -1.34(-1.47,-1.21) |
| Bhutan | male | 15.91(9.79,22.78) | 72.99(50.59,98.50) | 3.59 | 18.37(11.40,26.23) | 29.86(20.79,40.02) | 1.97(1.84,2.11) |
| Bolivia (Plurinational State of) | male | 405.93(310.74,549.05) | 1421.43(1039.05,1913.38) | 2.50 | 29.28(22.47,38.89) | 36.70(26.88,48.89) | 0.72(0.62,0.82) |
| Bosnia and Herzegovina | male | 167.78(153.91,183.08) | 910.05(721.65,1124.66) | 4.42 | 11.06(10.14,12.09) | 36.21(29.00,44.56) | 5.42(4.61,6.23) |
| Botswana | male | 131.33(97.33,172.36) | 460.35(329.69,603.04) | 2.51 | 59.86(44.91,76.62) | 95.75(70.27,123.58) | 1.03(0.38,1.69) |
| Brazil | male | 10379.73(9844.97,10872.28) | 28023.27(25954.75,29886.37) | 1.70 | 27.47(25.78,28.85) | 28.10(25.74,30.01) | 0.18(0.11,0.25) |
| Brunei Darussalam | male | 45.17(36.96,54.95) | 89.67(75.63,107.14) | 0.98 | 133.85(110.60,162.69) | 98.01(84.92,113.63) | -0.29(-0.70,0.11) |
| Bulgaria | male | 831.11(760.52,898.02) | 933.12(742.02,1165.51) | 0.12 | 14.70(13.50,15.84) | 15.07(12.03,18.71) | -0.71(-1.11,-0.31) |
| Burkina Faso | male | 721.19(577.24,876.79) | 1533.12(1227.29,1886.74) | 1.13 | 41.77(34.29,50.27) | 44.70(36.44,53.95) | 0.13(0.03,0.23) |
| Burundi | male | 514.37(381.08,723.26) | 778.02(596.98,1019.75) | 0.51 | 53.87(40.04,74.78) | 40.98(32.09,53.57) | -1.18(-1.27,-1.09) |
| Cambodia | male | 548.37(406.69,708.62) | 1411.94(1069.74,1785.71) | 1.57 | 31.28(23.80,39.48) | 32.31(24.94,40.41) | 0.10(0.01,0.20) |
| Cameroon | male | 700.61(496.67,962.94) | 2411.12(1773.14,3226.07) | 2.44 | 38.80(28.08,53.23) | 50.90(38.40,66.32) | 0.83(0.46,1.20) |
| Canada | male | 1767.25(1648.24,1885.22) | 3613.67(3239.74,3961.28) | 1.04 | 13.73(12.80,14.61) | 11.36(10.19,12.48) | -1.59(-2.28,-0.90) |
| Cabo Verde | male | 8.38(6.95,10.04) | 48.98(42.37,56.99) | 4.85 | 8.63(7.15,10.22) | 29.55(25.57,34.34) | 2.77(1.95,3.60) |
| Central African Republic | male | 340.95(270.64,423.89) | 569.19(425.31,760.95) | 0.67 | 70.52(56.25,85.32) | 66.67(51.83,84.27) | -0.14(-0.24,-0.04) |
| Chad | male | 292.28(224.71,393.43) | 765.48(583.32,1024.90) | 1.62 | 23.75(18.16,32.06) | 29.67(22.94,39.17) | 0.58(0.19,0.97) |
| Chile | male | 678.38(640.46,719.69) | 1768.67(1594.70,1937.16) | 1.61 | 16.22(15.28,17.17) | 17.16(15.40,18.79) | 0.15(-0.10,0.40) |
| China | male | 26648.89(22298.37,30997.51) | 83173.52(67047.21,101487.29) | 2.12 | 8.15(6.98,9.34) | 10.45(8.61,12.51) | 1.07(0.87,1.27) |
| Colombia | male | 1249.26(1178.54,1316.54) | 2920.14(2281.96,3738.79) | 1.34 | 15.69(14.68,16.56) | 12.22(9.53,15.65) | -1.87(-2.40,-1.33) |
| Comoros | male | 38.16(24.05,49.57) | 72.60(56.94,92.64) | 0.90 | 41.15(28.38,52.35) | 38.12(30.41,48.01) | -0.44(-0.58,-0.30) |
| Congo | male | 325.65(265.95,389.31) | 581.52(442.78,739.23) | 0.79 | 79.57(65.41,94.68) | 58.77(45.94,72.90) | -1.37(-1.58,-1.16) |
| Costa Rica | male | 98.54(90.88,105.97) | 264.57(204.94,338.12) | 1.68 | 12.08(11.10,13.05) | 11.51(8.92,14.69) | -0.96(-1.48,-0.43) |
| C么te d'Ivoire | male | 631.49(507.08,772.02) | 1686.80(1290.66,2168.61) | 1.67 | 36.83(30.04,44.64) | 38.50(30.60,47.51) | -0.37(-0.81,0.06) |
| Croatia | male | 272.97(249.20,299.39) | 470.05(372.35,583.76) | 0.72 | 11.74(10.76,12.84) | 12.78(10.20,15.79) | -0.08(-0.36,0.21) |
| Cuba | male | 750.28(703.89,792.49) | 814.03(658.98,988.77) | 0.08 | 15.05(14.11,15.87) | 9.09(7.34,11.00) | -2.01(-2.88,-1.13) |
| Cyprus | male | 163.28(129.72,201.08) | 233.71(200.47,272.54) | 0.43 | 50.58(40.58,61.69) | 28.10(24.07,32.72) | -2.53(-2.98,-2.08) |
| Czechia | male | 694.72(632.21,744.58) | 1508.58(1209.26,1819.25) | 1.17 | 13.10(11.98,14.01) | 16.96(13.71,20.28) | 2.88(1.94,3.84) |
| Democratic Republic of the Congo | male | 3485.08(2805.04,4268.72) | 6389.20(4934.69,8208.47) | 0.83 | 57.47(46.98,69.39) | 50.32(40.21,62.13) | -0.59(-0.66,-0.53) |
| Denmark | male | 376.95(353.11,408.47) | 743.44(664.35,820.21) | 0.97 | 11.23(10.54,12.15) | 14.05(12.53,15.50) | 0.87(0.25,1.49) |
| Djibouti | male | 20.38(14.47,29.58) | 119.46(87.48,166.81) | 4.86 | 38.14(27.89,53.57) | 49.03(37.63,65.21) | 0.88(0.78,0.98) |
| Dominica | male | 15.99(13.85,18.28) | 22.91(18.11,28.25) | 0.43 | 54.84(47.83,62.23) | 54.32(43.26,66.85) | -0.43(-0.59,-0.27) |
| Dominican Republic | male | 328.50(283.30,379.66) | 1415.25(997.07,1969.53) | 3.31 | 18.55(16.01,21.40) | 32.08(22.79,44.42) | 2.37(2.16,2.58) |
| Ecuador | male | 440.73(410.79,472.72) | 2221.88(1727.78,2899.77) | 4.04 | 17.67(16.36,19.02) | 33.13(26.08,42.69) | 2.08(1.79,2.37) |
| Egypt | male | 2608.68(2277.39,2908.99) | 9507.68(6892.74,12722.65) | 2.64 | 19.76(17.14,22.04) | 29.15(21.41,38.75) | 1.53(1.40,1.66) |
| El Salvador | male | 206.55(188.03,227.93) | 877.11(658.64,1127.24) | 3.25 | 15.36(13.93,17.00) | 34.81(26.02,45.01) | 2.71(2.30,3.13) |
| Equatorial Guinea | male | 50.91(36.09,65.38) | 115.39(78.40,157.38) | 1.27 | 65.27(45.73,81.64) | 72.96(52.15,95.55) | 0.44(0.17,0.70) |
| Eritrea | male | 166.99(102.31,254.75) | 450.55(304.26,634.13) | 1.70 | 45.99(28.39,67.00) | 49.56(34.12,68.12) | 0.05(-0.16,0.26) |
| Estonia | male | 25.06(22.44,28.37) | 48.18(37.10,60.36) | 0.92 | 3.62(3.26,4.05) | 5.00(3.86,6.27) | 1.24(0.46,2.03) |
| Ethiopia | male | 6117.40(4685.69,8515.62) | 7202.74(5756.86,8961.69) | 0.18 | 66.54(51.27,89.17) | 40.89(32.84,50.57) | -1.81(-2.01,-1.61) |
| Micronesia (Federated States of) | male | 19.34(14.88,25.83) | 50.87(30.64,78.71) | 1.63 | 90.57(71.85,116.23) | 164.82(106.47,245.69) | 1.95(1.60,2.29) |
| Fiji | male | 259.17(209.77,333.49) | 820.18(658.76,1020.60) | 2.16 | 166.68(137.05,213.96) | 280.13(231.67,338.27) | 1.32(0.87,1.77) |
| Finland | male | 150.20(139.06,168.68) | 225.14(199.85,248.63) | 0.50 | 5.96(5.51,6.63) | 4.18(3.71,4.60) | -1.47(-1.60,-1.35) |
| France | male | 2677.90(2507.87,2866.55) | 5824.83(5196.29,6444.67) | 1.18 | 8.56(7.96,9.21) | 9.10(8.17,10.01) | 0.23(-0.40,0.86) |
| Gabon | male | 207.46(148.87,294.60) | 373.26(285.94,468.45) | 0.80 | 97.12(70.58,137.89) | 94.38(73.53,115.67) | -0.18(-0.23,-0.14) |
| Georgia | male | 266.23(236.18,298.99) | 586.70(491.55,690.25) | 1.20 | 11.36(10.17,12.66) | 24.43(20.52,28.68) | 2.89(1.55,4.25) |
| Germany | male | 6430.11(6037.86,6797.32) | 9738.09(8822.31,10667.58) | 0.51 | 14.35(13.45,15.19) | 11.01(10.04,12.02) | -1.41(-1.66,-1.16) |
| Ghana | male | 648.75(517.98,800.97) | 3364.02(2645.98,4228.14) | 4.19 | 27.04(22.11,32.45) | 56.47(45.18,70.02) | 3.24(2.69,3.80) |
| Greece | male | 436.80(410.17,462.97) | 645.48(578.89,706.60) | 0.48 | 6.54(6.11,6.92) | 5.61(5.08,6.14) | 0.27(-0.16,0.69) |
| Greenland | male | 2.35(1.93,2.87) | 3.90(3.04,4.77) | 0.66 | 15.84(13.07,19.23) | 11.83(9.40,14.23) | -0.90(-1.03,-0.78) |
| Grenada | male | 18.75(16.63,20.93) | 32.08(28.46,36.25) | 0.71 | 61.25(54.41,68.07) | 68.79(61.56,76.48) | 0.35(-0.30,1.01) |
| Guam | male | 9.20(7.75,10.89) | 20.49(16.82,24.67) | 1.23 | 28.87(24.42,34.00) | 23.01(18.90,27.41) | -0.70(-1.01,-0.40) |
| Guatemala | male | 243.88(205.49,286.77) | 2570.43(1985.47,3219.69) | 9.54 | 14.51(12.32,16.90) | 54.81(43.07,67.89) | 3.83(3.07,4.59) |
| Guinea | male | 356.99(273.44,461.87) | 799.08(617.80,1007.83) | 1.24 | 24.26(18.57,31.48) | 32.61(25.44,40.77) | 1.09(0.80,1.38) |
| Guinea-Bissau | male | 83.60(62.42,113.94) | 128.84(98.37,165.06) | 0.54 | 47.10(35.53,62.55) | 46.76(36.84,58.68) | -0.15(-0.37,0.08) |
| Guyana | male | 120.10(102.26,137.82) | 239.09(184.34,302.73) | 0.99 | 69.52(59.84,79.32) | 87.73(69.23,109.30) | 0.53(0.08,0.98) |
| Haiti | male | 660.63(508.18,901.32) | 1046.70(737.30,1518.05) | 0.58 | 46.46(36.06,63.00) | 36.08(25.61,52.08) | -0.69(-0.93,-0.45) |
| Honduras | male | 120.24(92.77,147.43) | 492.41(386.87,618.96) | 3.10 | 12.05(9.16,14.72) | 18.43(14.53,23.15) | 1.71(1.43,1.98) |
| Hungary | male | 606.83(566.71,642.23) | 1054.66(858.03,1271.07) | 0.74 | 10.30(9.62,10.86) | 13.78(11.25,16.63) | 1.38(0.96,1.81) |
| Iceland | male | 7.10(6.33,7.97) | 16.37(14.16,18.69) | 1.30 | 5.56(4.97,6.24) | 6.10(5.28,6.97) | 0.06(-0.12,0.25) |
| India | male | 39255.37(31707.10,49454.75) | 132827.91(108478.49,160464.21) | 2.38 | 22.42(18.06,28.29) | 28.38(23.29,33.93) | 0.84(0.63,1.06) |
| Indonesia | male | 13640.27(11528.54,15652.22) | 48236.45(38396.24,59349.50) | 2.54 | 31.28(26.52,35.88) | 49.74(40.39,59.38) | 1.87(1.76,1.97) |
| Iran (Islamic Republic of) | male | 1113.78(944.92,1327.65) | 6524.28(5882.44,7190.75) | 4.86 | 10.93(9.27,13.11) | 19.84(17.77,21.94) | 2.52(2.21,2.83) |
| Iraq | male | 1611.37(1259.62,2008.70) | 4604.98(3564.16,5554.69) | 1.86 | 45.87(35.77,57.07) | 47.92(38.44,56.39) | -0.02(-0.16,0.12) |
| Ireland | male | 192.96(178.71,206.03) | 261.05(227.77,291.49) | 0.35 | 11.24(10.43,11.97) | 7.78(6.79,8.67) | -1.67(-1.92,-1.41) |
| Israel | male | 354.55(329.97,378.48) | 1217.64(1086.51,1332.16) | 2.43 | 16.62(15.46,17.70) | 22.76(20.36,24.87) | -0.15(-1.20,0.92) |
| Italy | male | 6312.47(6051.10,6519.82) | 9331.73(8355.49,9965.34) | 0.48 | 17.32(16.55,17.92) | 13.75(12.42,14.65) | -0.77(-0.83,-0.71) |
| Jamaica | male | 394.71(361.90,425.43) | 941.04(754.46,1151.26) | 1.38 | 47.97(43.95,51.72) | 67.13(53.74,82.09) | 1.15(0.68,1.64) |
| Japan | male | 4638.90(4434.11,4789.91) | 4175.54(3704.13,4527.03) | -0.10 | 6.88(6.51,7.12) | 2.55(2.28,2.75) | -3.39(-3.77,-3.01) |
| Jordan | male | 253.18(201.39,317.26) | 1054.48(820.89,1333.31) | 3.16 | 46.26(36.74,57.63) | 38.65(30.57,48.41) | -0.67(-0.82,-0.52) |
| Kazakhstan | male | 249.77(231.13,269.52) | 879.19(739.88,1022.92) | 2.52 | 5.59(5.20,6.01) | 14.31(12.04,16.69) | 2.09(1.31,2.87) |
| Kenya | male | 1084.15(893.07,1320.82) | 3482.64(2755.69,4318.51) | 2.21 | 32.34(26.84,39.34) | 42.89(34.76,51.93) | 0.97(0.67,1.28) |
| Kiribati | male | 23.37(18.12,29.81) | 67.95(45.88,92.24) | 1.91 | 140.68(110.95,175.10) | 237.78(161.20,311.82) | 1.38(0.78,1.98) |
| Kuwait | male | 66.12(58.62,76.91) | 225.28(176.67,283.61) | 2.41 | 24.49(21.28,28.95) | 19.56(15.34,24.82) | -1.33(-2.18,-0.48) |
| Kyrgyzstan | male | 73.11(66.77,79.85) | 128.88(109.49,150.71) | 0.76 | 6.06(5.54,6.62) | 6.47(5.55,7.52) | -0.81(-1.26,-0.35) |
| Lao People's Democratic Republic | male | 306.23(216.33,414.93) | 608.20(438.17,849.49) | 0.99 | 34.01(24.46,44.83) | 32.79(24.40,44.49) | -0.26(-0.31,-0.21) |
| Latvia | male | 53.16(48.65,58.76) | 109.13(86.15,134.41) | 1.05 | 4.28(3.91,4.71) | 7.81(6.19,9.58) | 2.16(1.64,2.69) |
| Lebanon | male | 221.62(184.06,271.32) | 492.80(305.26,689.25) | 1.22 | 23.33(19.39,28.36) | 21.23(13.25,29.63) | 0.17(-0.22,0.55) |
| Lesotho | male | 203.93(159.88,255.07) | 514.36(370.06,690.36) | 1.52 | 55.63(44.29,68.58) | 114.48(83.09,149.19) | 2.77(2.50,3.04) |
| Liberia | male | 149.80(120.03,185.98) | 259.00(178.98,365.64) | 0.73 | 28.63(23.19,34.93) | 30.02(21.59,41.91) | 0.13(-0.04,0.30) |
| Libya | male | 111.82(78.09,147.41) | 390.40(248.50,571.37) | 2.49 | 12.92(9.14,17.00) | 16.97(10.79,24.72) | 1.26(1.07,1.46) |
| Lithuania | male | 62.61(57.29,68.57) | 91.76(74.75,112.06) | 0.47 | 3.70(3.39,4.04) | 4.46(3.63,5.43) | 0.62(0.31,0.94) |
| Luxembourg | male | 20.19(18.24,22.30) | 29.15(24.70,34.15) | 0.44 | 10.13(9.19,11.14) | 6.55(5.56,7.64) | -1.57(-1.76,-1.38) |
| North Macedonia | male | 130.77(114.44,147.06) | 427.54(331.26,539.23) | 2.27 | 15.69(13.70,17.64) | 30.82(24.42,38.16) | 2.51(1.93,3.10) |
| Madagascar | male | 735.78(567.08,920.58) | 1315.64(954.28,1754.81) | 0.79 | 32.88(25.49,41.23) | 32.16(23.92,42.00) | -0.09(-0.22,0.04) |
| Malawi | male | 676.05(557.60,798.72) | 1335.94(1091.98,1629.44) | 0.98 | 45.80(37.93,54.03) | 51.87(43.26,61.49) | 0.32(-0.05,0.68) |
| Malaysia | male | 953.26(835.03,1069.77) | 1542.23(1204.66,1959.10) | 0.62 | 22.88(19.86,25.79) | 12.46(9.79,15.61) | -3.33(-4.00,-2.65) |
| Maldives | male | 11.74(9.30,14.81) | 28.40(23.13,34.32) | 1.42 | 27.59(22.29,33.84) | 20.47(16.65,24.66) | -1.40(-1.74,-1.06) |
| Mali | male | 427.93(336.17,536.22) | 989.58(753.90,1293.61) | 1.31 | 24.99(20.00,31.08) | 26.40(20.38,33.92) | 0.17(0.10,0.23) |
| Malta | male | 35.28(31.95,39.02) | 57.45(50.34,65.32) | 0.63 | 21.06(19.07,23.15) | 13.78(12.07,15.62) | -1.22(-1.46,-0.98) |
| Marshall Islands | male | 5.47(4.39,6.89) | 13.50(9.79,18.13) | 1.47 | 76.76(61.90,95.64) | 85.60(65.10,110.46) | 0.47(0.30,0.65) |
| Mauritania | male | 131.54(105.94,160.03) | 233.20(161.71,315.09) | 0.77 | 32.04(26.10,38.48) | 25.83(18.22,34.17) | -0.92(-1.00,-0.84) |
| Mauritius | male | 152.31(140.56,164.41) | 925.57(748.07,1124.55) | 5.08 | 46.39(43.13,49.70) | 120.85(98.35,145.82) | 5.52(4.46,6.59) |
| Mexico | male | 11148.41(10696.23,11496.14) | 36231.85(29810.25,43807.90) | 2.25 | 56.39(53.93,58.28) | 68.72(56.65,82.85) | 0.50(0.31,0.69) |
| Republic of Moldova | male | 110.81(103.42,119.02) | 114.60(97.17,132.28) | 0.03 | 6.32(5.91,6.78) | 4.97(4.22,5.70) | -1.61(-1.99,-1.24) |
| Mongolia | male | 24.21(18.99,30.36) | 70.31(52.05,95.82) | 1.90 | 5.40(4.38,6.61) | 6.92(5.38,8.85) | 0.53(0.22,0.83) |
| Montenegro | male | 28.60(24.14,33.27) | 60.65(48.73,74.48) | 1.12 | 11.42(9.70,13.17) | 14.36(11.68,17.46) | 0.95(0.78,1.12) |
| Morocco | male | 648.64(503.26,818.56) | 2607.31(1899.31,3267.46) | 3.02 | 11.28(8.77,14.13) | 19.43(14.32,24.06) | 1.89(1.51,2.27) |
| Mozambique | male | 1021.70(824.61,1238.47) | 2634.31(2085.99,3297.42) | 1.58 | 43.91(35.80,52.71) | 65.45(53.45,79.72) | 1.84(1.67,2.01) |
| Myanmar | male | 5361.87(3915.30,7143.01) | 10601.36(8941.13,12705.75) | 0.98 | 53.99(40.44,69.60) | 58.81(50.65,69.21) | 0.30(0.25,0.35) |
| Namibia | male | 167.22(127.19,212.14) | 357.05(280.82,463.33) | 1.14 | 56.74(44.25,71.66) | 70.26(55.68,90.31) | 0.46(0.06,0.86) |
| Nepal | male | 458.99(342.77,597.53) | 1650.09(1200.78,2129.00) | 2.60 | 10.77(8.07,13.78) | 17.22(12.66,22.03) | 1.82(1.50,2.15) |
| Netherlands | male | 1217.75(1144.18,1294.79) | 1437.98(1294.25,1579.75) | 0.18 | 15.95(14.91,16.95) | 9.41(8.49,10.33) | -2.25(-2.68,-1.81) |
| New Zealand | male | 197.75(182.18,214.58) | 355.25(323.31,387.02) | 0.80 | 11.95(10.97,12.91) | 9.67(8.80,10.50) | -1.77(-2.34,-1.20) |
| Nicaragua | male | 203.07(183.01,226.41) | 863.37(682.64,1052.19) | 3.25 | 31.87(28.60,35.59) | 48.25(38.52,58.11) | 1.16(0.94,1.37) |
| Niger | male | 299.31(218.13,400.44) | 801.33(589.08,1081.97) | 1.68 | 25.26(18.60,33.45) | 26.32(19.86,34.71) | 0.22(0.10,0.35) |
| Nigeria | male | 6117.47(4506.78,7951.25) | 12233.98(8866.76,17021.32) | 1.00 | 33.99(25.93,43.00) | 36.82(27.70,49.45) | 0.33(0.19,0.47) |
| Democratic People's Republic of Korea | male | 668.48(490.37,873.81) | 1437.43(1136.15,1783.35) | 1.15 | 12.51(9.38,16.17) | 12.13(9.86,14.61) | -0.07(-0.17,0.03) |
| Northern Mariana Islands | male | 3.84(3.15,4.72) | 14.83(12.49,17.34) | 2.86 | 44.23(37.55,53.17) | 63.92(55.23,73.20) | 1.60(1.00,2.20) |
| Norway | male | 222.49(205.37,234.06) | 300.49(273.11,325.01) | 0.35 | 7.88(7.24,8.29) | 6.72(6.11,7.28) | -0.70(-1.12,-0.27) |
| Oman | male | 109.73(81.40,142.97) | 357.93(276.55,438.59) | 2.26 | 41.85(32.21,53.18) | 64.24(51.47,76.74) | 2.02(1.79,2.24) |
| Pakistan | male | 6732.36(4194.23,8928.17) | 20331.40(14992.09,27086.18) | 2.02 | 23.98(15.02,31.97) | 44.06(32.96,57.72) | 2.17(1.90,2.43) |
| Palestine | male | 175.01(132.23,227.14) | 598.87(509.99,698.07) | 2.42 | 50.97(38.72,65.48) | 71.50(60.98,83.00) | 1.10(0.72,1.48) |
| Panama | male | 106.08(96.30,116.65) | 542.86(419.56,697.57) | 4.12 | 14.86(13.45,16.32) | 27.53(21.30,35.33) | 1.72(1.40,2.04) |
| Papua New Guinea | male | 917.17(689.78,1188.70) | 2992.65(2258.41,3817.07) | 2.26 | 107.91(82.29,138.65) | 134.29(104.27,167.79) | 0.63(0.45,0.81) |
| Paraguay | male | 180.29(155.97,207.00) | 1076.65(822.63,1382.26) | 4.97 | 18.81(16.22,21.51) | 43.51(33.57,55.57) | 3.11(2.66,3.57) |
| Peru | male | 726.96(618.04,843.25) | 2201.75(1594.95,2955.11) | 2.03 | 13.07(11.14,15.21) | 14.45(10.47,19.42) | 0.78(0.49,1.08) |
| Philippines | male | 4622.15(4045.75,5265.92) | 12948.79(9928.77,16660.64) | 1.80 | 36.17(31.83,40.84) | 38.41(29.83,48.67) | 0.03(-0.19,0.24) |
| Poland | male | 1966.86(1832.33,2082.29) | 3432.35(2742.90,4272.16) | 0.75 | 11.09(10.40,11.73) | 12.17(9.77,15.06) | 0.74(0.39,1.09) |
| Portugal | male | 1110.68(1052.55,1171.23) | 1767.93(1597.81,1937.57) | 0.59 | 19.57(18.53,20.54) | 15.92(14.44,17.32) | -1.25(-1.61,-0.88) |
| Puerto Rico | male | 698.61(651.58,741.31) | 1516.26(1174.54,1914.70) | 1.17 | 42.90(39.91,45.51) | 46.71(35.96,59.16) | -0.08(-0.47,0.30) |
| Qatar | male | 35.81(28.22,45.74) | 226.54(162.74,301.54) | 5.33 | 112.67(91.11,137.90) | 107.38(83.77,136.35) | 0.12(-0.39,0.63) |
| Romania | male | 805.41(757.35,859.47) | 1083.92(882.72,1283.21) | 0.35 | 6.61(6.19,7.02) | 6.85(5.59,8.10) | 0.12(-0.20,0.43) |
| Russian Federation | male | 1934.28(1816.58,2045.98) | 5088.27(4213.18,6093.57) | 1.63 | 3.57(3.37,3.75) | 6.12(5.09,7.31) | 1.65(0.93,2.37) |
| Rwanda | male | 668.51(515.21,920.32) | 887.04(642.79,1205.73) | 0.33 | 59.43(46.51,81.39) | 45.89(33.78,61.80) | -1.74(-2.07,-1.41) |
| Saint Lucia | male | 24.93(22.52,27.43) | 57.09(48.15,68.16) | 1.29 | 72.30(65.63,79.06) | 61.18(51.66,72.16) | -1.23(-1.54,-0.93) |
| Saint Vincent and the Grenadines | male | 18.38(16.24,20.63) | 48.18(41.91,55.82) | 1.62 | 62.81(56.12,70.10) | 74.34(65.10,85.33) | 0.02(-0.25,0.28) |
| Samoa | male | 31.32(24.31,40.07) | 56.19(46.47,67.79) | 0.79 | 81.29(64.03,102.39) | 85.84(71.66,100.99) | 0.01(-0.19,0.21) |
| Sao Tome and Principe | male | 3.66(2.82,4.61) | 7.92(6.17,9.58) | 1.16 | 14.42(11.53,17.71) | 18.86(15.00,22.69) | 1.00(0.92,1.08) |
| Saudi Arabia | male | 688.37(496.73,964.43) | 1767.37(1378.83,2168.70) | 1.57 | 24.66(18.11,33.88) | 20.78(16.82,24.74) | -1.29(-1.63,-0.95) |
| Senegal | male | 428.03(328.20,540.69) | 1056.66(774.32,1349.98) | 1.47 | 30.71(24.03,38.36) | 34.54(26.25,43.62) | 0.36(0.16,0.56) |
| Serbia | male | 810.76(663.51,957.22) | 1543.37(1222.96,1936.36) | 0.90 | 16.18(13.10,19.03) | 21.51(17.29,26.74) | 1.27(1.02,1.53) |
| Seychelles | male | 3.16(2.70,3.75) | 10.65(8.98,12.69) | 2.37 | 13.80(11.80,16.29) | 23.13(19.65,27.20) | 1.51(1.23,1.80) |
| Sierra Leone | male | 230.26(180.81,286.26) | 403.81(299.55,535.69) | 0.75 | 25.85(20.57,31.86) | 26.68(20.22,34.88) | 0.12(-0.10,0.33) |
| Singapore | male | 140.41(131.88,149.34) | 86.35(77.06,96.47) | -0.39 | 15.81(14.82,16.85) | 2.64(2.33,2.96) | -6.41(-7.48,-5.33) |
| Slovakia | male | 321.42(287.93,361.68) | 335.05(250.19,429.24) | 0.04 | 12.97(11.67,14.47) | 8.92(6.71,11.29) | -1.10(-1.23,-0.97) |
| Slovenia | male | 84.93(65.73,107.45) | 166.08(130.65,210.65) | 0.96 | 9.71(7.59,12.18) | 9.00(7.09,11.40) | -2.39(-3.35,-1.41) |
| Solomon Islands | male | 55.81(39.59,85.95) | 202.64(158.70,252.55) | 2.63 | 73.24(52.39,113.41) | 128.09(103.11,156.16) | 1.93(1.69,2.16) |
| Somalia | male | 513.01(372.53,691.99) | 1150.94(833.61,1523.37) | 1.24 | 51.19(38.08,66.33) | 51.02(38.58,65.31) | 0.08(0.01,0.15) |
| South Africa | male | 3205.64(2757.37,3668.81) | 11041.58(10031.07,12043.70) | 2.44 | 40.39(34.62,45.98) | 69.28(63.30,75.23) | 2.12(1.59,2.65) |
| Republic of Korea | male | 2483.13(2346.86,2660.70) | 6267.55(5535.68,7062.37) | 1.52 | 21.53(20.20,23.43) | 17.96(15.92,20.17) | -1.33(-2.16,-0.50) |
| South Sudan | male | 433.08(319.93,580.66) | 629.63(446.61,857.06) | 0.45 | 39.10(29.26,51.59) | 38.42(28.08,51.19) | -0.06(-0.10,-0.02) |
| Spain | male | 3233.48(3037.72,3430.37) | 4022.10(3597.63,4415.36) | 0.24 | 15.00(14.06,15.94) | 8.73(7.84,9.56) | -2.03(-2.21,-1.84) |
| Sri Lanka | male | 1290.26(1130.57,1460.63) | 5863.71(4413.74,7641.64) | 3.54 | 28.64(25.12,32.21) | 61.60(47.66,79.16) | 3.76(3.13,4.39) |
| Sudan | male | 437.14(328.86,617.86) | 1313.58(794.15,2009.72) | 2.00 | 10.14(7.60,14.13) | 14.89(9.08,22.53) | 1.56(1.31,1.80) |
| Suriname | male | 39.85(35.31,44.64) | 126.68(103.09,153.64) | 2.18 | 34.30(30.47,38.34) | 48.05(38.81,57.99) | 1.05(0.72,1.38) |
| Eswatini | male | 97.91(73.34,126.39) | 333.16(224.85,428.14) | 2.40 | 92.45(70.04,117.99) | 170.78(116.85,214.47) | 2.32(1.71,2.93) |
| Sweden | male | 616.43(568.14,664.14) | 1018.83(913.33,1113.06) | 0.65 | 9.76(8.96,10.47) | 9.76(8.78,10.61) | -0.11(-0.37,0.15) |
| Switzerland | male | 567.35(529.63,608.42) | 632.65(557.07,701.91) | 0.12 | 13.70(12.75,14.70) | 7.58(6.71,8.40) | -2.63(-2.96,-2.31) |
| Syrian Arab Republic | male | 370.12(286.37,470.84) | 748.21(548.48,997.35) | 1.02 | 14.81(11.38,18.85) | 13.68(10.17,18.05) | -0.90(-1.21,-0.58) |
| Taiwan (Province of China) | male | 1975.41(1864.34,2079.56) | 5281.97(4170.60,6792.14) | 1.67 | 26.16(24.68,27.50) | 28.90(22.81,37.18) | -0.69(-1.30,-0.08) |
| Tajikistan | male | 157.92(140.54,178.93) | 752.19(590.61,989.09) | 3.76 | 12.47(11.15,14.08) | 32.89(26.26,41.49) | 3.74(3.32,4.16) |
| United Republic of Tanzania | male | 1712.31(1422.25,2079.81) | 4097.49(3044.63,5106.56) | 1.39 | 39.37(32.60,47.53) | 42.67(31.97,52.60) | 0.24(0.10,0.37) |
| Thailand | male | 3312.17(2550.00,4275.72) | 7869.45(5644.93,10389.84) | 1.38 | 21.74(16.81,28.02) | 17.32(12.50,22.67) | -1.57(-2.10,-1.04) |
| Bahamas | male | 26.69(23.78,29.93) | 62.17(50.02,76.69) | 1.33 | 43.53(38.98,48.67) | 38.63(31.30,47.20) | -0.89(-1.09,-0.69) |
| Gambia | male | 38.79(29.09,52.30) | 139.10(104.73,183.33) | 2.59 | 26.30(20.28,34.58) | 35.54(26.97,46.18) | 0.79(0.55,1.03) |
| Timor-Leste | male | 19.43(13.42,25.97) | 71.32(48.61,95.17) | 2.67 | 15.94(11.36,20.82) | 20.06(13.97,26.25) | 0.86(0.60,1.11) |
| Togo | male | 129.08(102.16,162.47) | 424.51(318.54,561.96) | 2.29 | 26.62(21.53,33.24) | 34.15(25.76,44.03) | 0.74(0.37,1.11) |
| Tonga | male | 15.31(12.35,19.19) | 36.16(25.95,47.21) | 1.36 | 63.74(51.68,78.89) | 105.22(76.28,136.34) | 1.89(1.42,2.36) |
| Trinidad and Tobago | male | 449.92(424.89,478.46) | 921.19(690.18,1210.55) | 1.05 | 119.68(112.93,126.84) | 107.63(81.24,140.38) | -0.89(-1.16,-0.63) |
| Tunisia | male | 221.26(169.83,278.52) | 862.81(592.18,1236.65) | 2.90 | 10.27(8.01,12.83) | 15.65(10.90,22.08) | 1.65(1.53,1.77) |
| Turkey | male | 5088.79(4034.30,6426.08) | 7949.46(6301.42,9936.38) | 0.56 | 32.99(26.10,41.64) | 20.77(16.46,25.88) | -1.23(-1.45,-1.01) |
| Turkmenistan | male | 84.95(77.10,92.04) | 307.54(240.31,398.25) | 2.62 | 10.36(9.46,11.20) | 17.05(13.42,21.66) | 0.92(0.49,1.35) |
| Uganda | male | 1277.67(886.99,1847.13) | 2677.13(1880.33,3605.15) | 1.10 | 48.16(34.25,69.09) | 54.67(39.23,72.95) | 0.08(-0.16,0.33) |
| Ukraine | male | 918.41(866.06,974.38) | 1036.35(830.74,1281.04) | 0.13 | 3.83(3.61,4.04) | 3.80(3.07,4.64) | -1.15(-1.55,-0.75) |
| United Arab Emirates | male | 105.66(79.35,139.93) | 986.31(659.94,1379.57) | 8.33 | 66.90(50.61,86.36) | 57.07(40.37,75.28) | -0.81(-1.21,-0.41) |
| United Kingdom | male | 3224.97(3079.08,3323.98) | 2905.26(2666.35,3066.00) | -0.10 | 9.32(8.82,9.63) | 4.95(4.54,5.22) | -2.60(-2.81,-2.40) |
| United States of America | male | 18567.64(17590.51,19301.85) | 38827.32(36553.44,40540.82) | 1.09 | 14.13(13.38,14.71) | 15.40(14.49,16.10) | -0.33(-0.81,0.16) |
| Uruguay | male | 287.22(269.64,305.83) | 402.56(367.65,441.44) | 0.40 | 17.37(16.32,18.44) | 17.51(16.01,19.19) | 0.15(0.02,0.28) |
| Uzbekistan | male | 466.11(431.14,509.00) | 3447.41(2824.34,4127.67) | 6.40 | 9.54(8.78,10.32) | 37.26(31.24,43.68) | 4.51(3.85,5.16) |
| Vanuatu | male | 13.23(9.50,21.46) | 56.78(43.07,78.21) | 3.29 | 40.50(29.34,66.59) | 68.20(52.21,92.32) | 2.00(1.88,2.11) |
| Venezuela (Bolivarian Republic of) | male | 1333.32(1254.24,1404.12) | 5416.71(4076.24,7021.38) | 3.06 | 30.86(28.83,32.67) | 41.90(31.82,53.76) | 0.86(0.61,1.10) |
| Viet nam | male | 3368.02(2529.28,4271.51) | 10995.51(8873.21,13464.92) | 2.26 | 22.94(17.26,28.67) | 33.21(27.25,39.97) | 1.69(1.52,1.87) |
| Virginia | male | 349.92(320.62,378.79) | 948.92(735.26,1196.32) | 1.71 | 12.56(11.52,13.53) | 15.03(11.71,18.89) | -0.07(-0.60,0.46) |
| Yemen | male | 196.20(135.20,288.33) | 650.07(448.63,919.86) | 2.31 | 10.26(7.45,14.51) | 11.69(8.26,16.23) | 0.68(0.53,0.83) |
| Zambia | male | 586.10(478.88,703.19) | 1432.27(1143.80,1769.56) | 1.44 | 46.70(38.09,56.54) | 55.23(45.35,66.50) | 0.29(-0.09,0.66) |
| Zimbabwe | male | 480.65(405.58,564.58) | 1213.49(925.96,1489.44) | 1.52 | 27.35(23.32,31.61) | 45.94(34.89,56.22) | 1.50(0.98,2.02) |
| Monaco | male | 1.20(0.97,1.47) | 2.05(1.69,2.45) | 0.71 | 3.96(3.21,4.78) | 4.31(3.52,5.16) | 0.45(0.21,0.70) |
| San Marino | male | 1.05(0.86,1.28) | 2.25(1.51,3.03) | 1.13 | 7.77(6.42,9.44) | 6.91(4.62,9.37) | -0.33(-0.38,-0.29) |
| Saint Kitts and Nevis | male | 9.40(8.27,10.56) | 16.39(13.84,19.11) | 0.74 | 60.38(53.87,67.05) | 58.48(50.81,66.69) | -0.49(-0.66,-0.32) |
| Cook Islands | male | 7.09(6.08,8.44) | 13.35(11.38,15.47) | 0.88 | 116.67(100.60,138.57) | 113.23(96.83,130.55) | -0.43(-0.71,-0.14) |
| Nauru | male | 1.88(1.36,2.77) | 2.78(1.86,4.51) | 0.48 | 103.31(78.02,141.88) | 157.64(112.93,236.65) | 1.19(0.86,1.52) |
| Niue | male | 0.77(0.62,0.99) | 1.20(0.87,1.55) | 0.57 | 85.05(68.60,109.36) | 127.23(92.32,164.03) | 1.25(0.91,1.60) |
| Palau | male | 3.30(2.63,4.26) | 11.80(8.57,15.51) | 2.57 | 74.25(60.06,93.84) | 119.25(88.77,152.99) | 1.55(1.25,1.85) |
| Tokelau | male | 0.28(0.23,0.33) | 0.41(0.31,0.52) | 0.48 | 46.34(38.35,55.88) | 65.74(49.52,82.92) | 1.21(0.95,1.48) |
| Tuvalu | male | 2.01(1.62,2.61) | 4.33(3.10,6.04) | 1.15 | 74.43(61.87,93.41) | 97.78(71.24,133.66) | 0.84(0.66,1.03) |
| Afghanistan | female | 1224.89(690.70,1943.17) | 3268.01(1661.38,5108.21) | 1.67 | 38.16(22.16,60.59) | 57.10(28.93,88.76) | 1.79(1.62,1.96) |
| Albania | female | 40.40(35.29,46.66) | 76.42(56.19,104.65) | 0.89 | 3.95(3.43,4.59) | 3.24(2.38,4.42) | -1.10(-1.55,-0.65) |
| Algeria | female | 877.01(576.67,1321.29) | 2904.37(2158.74,4089.14) | 2.31 | 20.23(13.34,31.07) | 22.91(16.90,33.43) | 1.11(0.78,1.45) |
| American Samoa | female | 6.92(5.82,8.42) | 21.62(17.74,26.67) | 2.12 | 71.69(59.87,87.92) | 93.87(77.25,115.13) | 0.80(0.61,0.99) |
| Andorra | female | 1.06(0.75,1.53) | 3.33(2.38,4.44) | 2.14 | 5.00(3.57,6.98) | 3.86(2.76,5.17) | -0.87(-1.15,-0.59) |
| Angola | female | 459.48(319.93,630.72) | 1445.20(1123.93,1902.09) | 2.15 | 27.59(19.19,37.69) | 29.61(23.58,38.57) | 0.15(0.05,0.24) |
| Antigua and Barbuda | female | 17.86(15.73,20.06) | 30.66(26.50,35.11) | 0.72 | 53.33(47.08,59.67) | 61.06(52.89,69.87) | -0.08(-0.30,0.13) |
| Argentina | female | 3394.51(3128.35,3609.23) | 4935.67(4445.47,5436.98) | 0.45 | 18.98(17.51,20.14) | 15.17(13.71,16.66) | -1.24(-1.48,-1.01) |
| Armenia | female | 290.24(269.32,313.21) | 710.59(588.47,835.89) | 1.45 | 19.36(17.99,20.88) | 29.23(24.11,34.37) | 0.42(-0.37,1.22) |
| Australia | female | 1084.52(973.63,1161.23) | 1906.30(1564.39,2153.26) | 0.76 | 9.61(8.65,10.26) | 7.00(5.88,7.86) | -1.28(-1.55,-1.02) |
| Austria | female | 991.73(900.03,1063.00) | 1179.17(973.53,1334.49) | 0.19 | 11.74(10.67,12.55) | 8.83(7.50,9.90) | -0.67(-1.09,-0.25) |
| Azerbaijan | female | 305.64(272.31,354.67) | 1076.73(832.64,1380.86) | 2.52 | 10.48(9.32,12.21) | 22.95(17.72,29.29) | 2.16(1.54,2.78) |
| Bahrain | female | 42.17(34.53,53.63) | 292.61(227.12,355.84) | 5.94 | 73.37(60.74,93.82) | 129.32(100.85,155.58) | 2.31(1.80,2.82) |
| Bangladesh | female | 4297.12(3261.90,5617.80) | 16024.02(12048.73,19998.45) | 2.73 | 24.76(18.70,31.35) | 32.29(23.35,40.18) | 0.92(0.37,1.48) |
| Barbados | female | 138.84(126.36,149.77) | 178.34(147.53,206.90) | 0.28 | 75.14(68.84,80.88) | 63.63(52.58,73.93) | -0.71(-0.86,-0.56) |
| Belarus | female | 360.12(333.12,386.04) | 212.79(168.86,268.44) | -0.41 | 4.29(3.98,4.60) | 1.99(1.57,2.52) | -4.33(-4.94,-3.72) |
| Belgium | female | 1196.36(1074.21,1291.16) | 940.88(780.49,1065.09) | -0.21 | 11.69(10.53,12.58) | 5.33(4.56,5.98) | -2.98(-3.14,-2.81) |
| Belize | female | 25.03(22.26,27.80) | 77.28(67.31,87.80) | 2.09 | 54.23(48.20,60.25) | 61.24(53.43,69.79) | 0.76(0.20,1.33) |
| Benin | female | 222.16(179.99,270.27) | 769.82(590.12,995.57) | 2.47 | 23.84(19.21,29.13) | 34.24(26.53,43.69) | 1.38(1.17,1.60) |
| Bermuda | female | 10.21(9.04,11.38) | 10.99(8.68,13.74) | 0.08 | 29.41(26.03,32.69) | 13.22(10.53,16.67) | -3.34(-3.56,-3.12) |
| Bhutan | female | 20.35(14.11,28.07) | 73.89(55.17,100.81) | 2.63 | 19.12(13.41,26.37) | 30.96(23.22,41.98) | 1.64(1.58,1.71) |
| Bolivia (Plurinational State of) | female | 757.18(563.58,960.30) | 2246.77(1697.88,2856.01) | 1.97 | 47.46(35.03,59.74) | 52.42(39.77,66.33) | 0.22(0.12,0.32) |
| Bosnia and Herzegovina | female | 333.45(299.69,377.87) | 1299.27(872.25,1614.95) | 2.90 | 16.27(14.58,18.32) | 36.93(25.02,45.94) | 3.76(3.11,4.42) |
| Botswana | female | 165.18(122.27,228.38) | 575.65(398.96,804.00) | 2.48 | 58.78(43.85,80.96) | 86.94(61.42,119.32) | 1.43(1.14,1.72) |
| Brazil | female | 15176.03(14162.12,15967.75) | 34859.36(31019.63,37443.18) | 1.30 | 35.60(32.90,37.67) | 26.97(23.98,28.98) | -0.98(-1.03,-0.94) |
| Brunei Darussalam | female | 39.00(31.35,46.32) | 72.90(63.31,83.14) | 0.87 | 96.24(77.45,113.03) | 60.28(53.10,68.13) | -1.31(-1.66,-0.96) |
| Bulgaria | female | 1055.79(946.53,1146.86) | 1168.87(940.51,1457.74) | 0.11 | 15.40(13.78,16.63) | 12.68(10.21,15.78) | -1.50(-1.90,-1.10) |
| Burkina Faso | female | 507.21(384.98,661.29) | 1150.25(916.95,1440.40) | 1.27 | 27.16(20.88,34.64) | 27.24(21.83,33.65) | -0.08(-0.19,0.02) |
| Burundi | female | 431.27(313.86,586.05) | 550.65(419.00,699.17) | 0.28 | 37.35(27.28,49.83) | 30.64(23.52,38.75) | -0.98(-1.12,-0.84) |
| Cambodia | female | 736.90(535.22,959.91) | 1939.73(1456.07,2440.45) | 1.63 | 31.51(23.71,40.32) | 31.23(23.67,39.17) | -0.21(-0.29,-0.12) |
| Cameroon | female | 623.09(481.41,789.97) | 2459.55(1800.88,3302.73) | 2.95 | 32.39(24.71,40.99) | 47.51(35.39,62.18) | 1.53(1.25,1.82) |
| Canada | female | 2107.68(1868.55,2269.52) | 3243.77(2692.74,3669.13) | 0.54 | 10.92(9.68,11.76) | 7.28(6.21,8.18) | -2.31(-2.93,-1.68) |
| Cabo Verde | female | 11.58(9.42,17.79) | 79.38(61.08,99.93) | 5.86 | 8.38(6.83,12.82) | 31.75(24.56,39.53) | 3.99(3.18,4.81) |
| Central African Republic | female | 168.22(122.40,224.45) | 355.79(249.49,506.81) | 1.12 | 31.12(22.24,41.11) | 35.90(25.45,49.86) | 0.65(0.41,0.89) |
| Chad | female | 308.84(220.97,413.13) | 813.39(579.92,1090.62) | 1.63 | 23.45(16.80,31.30) | 37.03(26.67,48.96) | 1.71(1.45,1.97) |
| Chile | female | 762.56(708.45,805.97) | 1955.91(1707.19,2159.40) | 1.56 | 14.76(13.72,15.58) | 14.13(12.39,15.59) | -0.34(-0.50,-0.19) |
| China | female | 37435.20(31607.20,45067.03) | 85214.59(68086.00,103515.70) | 1.28 | 9.33(7.89,11.14) | 8.49(6.80,10.28) | -0.23(-0.55,0.08) |
| Colombia | female | 1944.47(1814.19,2052.07) | 3971.65(3114.25,4949.02) | 1.04 | 23.53(21.75,24.92) | 13.23(10.36,16.50) | -2.90(-3.32,-2.47) |
| Comoros | female | 35.18(23.25,45.57) | 79.85(64.66,101.00) | 1.27 | 34.22(23.59,44.00) | 32.91(26.69,41.28) | -0.25(-0.35,-0.15) |
| Congo | female | 206.42(153.59,263.94) | 512.43(387.79,667.15) | 1.48 | 40.81(30.26,52.50) | 45.49(35.16,57.96) | 0.43(0.25,0.62) |
| Costa Rica | female | 156.29(141.61,168.30) | 298.77(233.02,375.76) | 0.91 | 18.02(16.31,19.45) | 10.58(8.24,13.34) | -2.71(-3.21,-2.21) |
| C么te d'Ivoire | female | 385.12(305.16,475.94) | 1555.13(1163.53,1972.46) | 3.04 | 27.00(21.72,33.04) | 38.16(29.38,47.69) | 1.41(1.09,1.73) |
| Croatia | female | 444.53(401.94,487.71) | 614.00(485.80,757.65) | 0.38 | 11.72(10.61,12.82) | 9.99(7.91,12.23) | -1.13(-1.42,-0.85) |
| Cuba | female | 1392.23(1297.66,1473.45) | 1153.49(936.88,1393.87) | -0.17 | 27.08(25.29,28.66) | 10.78(8.70,13.06) | -3.49(-4.19,-2.79) |
| Cyprus | female | 262.55(160.27,317.18) | 245.16(167.95,289.42) | -0.07 | 77.14(48.53,92.08) | 24.98(17.50,29.45) | -4.24(-4.40,-4.09) |
| Czechia | female | 975.33(880.13,1049.80) | 1720.77(1378.70,2051.83) | 0.76 | 11.27(10.13,12.09) | 12.26(9.87,14.67) | 2.11(1.20,3.03) |
| Democratic Republic of the Congo | female | 1791.39(1406.73,2266.46) | 3996.76(3041.26,5212.44) | 1.23 | 26.99(21.57,33.45) | 24.21(18.71,31.28) | -0.38(-0.44,-0.32) |
| Denmark | female | 394.90(360.35,428.19) | 607.83(522.06,692.35) | 0.54 | 7.71(7.07,8.44) | 8.15(7.08,9.23) | 0.26(-0.23,0.76) |
| Djibouti | female | 15.71(11.48,20.71) | 72.53(54.18,97.93) | 3.62 | 29.56(22.08,38.38) | 34.78(26.95,45.06) | 0.64(0.58,0.70) |
| Dominica | female | 32.86(28.61,37.21) | 32.41(26.76,39.14) | -0.01 | 72.52(63.11,81.97) | 65.40(53.74,78.98) | -0.45(-0.60,-0.30) |
| Dominican Republic | female | 407.96(355.20,466.17) | 1341.60(1046.89,1749.01) | 2.29 | 23.65(20.55,27.13) | 28.63(22.33,37.19) | 1.14(0.93,1.35) |
| Ecuador | female | 641.71(594.25,710.97) | 2608.42(2055.17,3241.05) | 3.06 | 26.12(24.05,29.46) | 35.41(27.85,43.80) | 1.10(0.81,1.40) |
| Egypt | female | 3400.89(3052.42,3823.94) | 9725.13(7120.48,13699.10) | 1.86 | 26.84(24.06,30.54) | 42.01(31.52,59.63) | 2.07(1.90,2.24) |
| El Salvador | female | 290.18(260.48,337.48) | 1305.13(956.14,1682.16) | 3.50 | 18.79(16.82,21.80) | 37.37(27.25,48.49) | 2.40(2.12,2.69) |
| Equatorial Guinea | female | 26.71(17.06,38.23) | 95.01(63.57,138.01) | 2.56 | 27.49(17.48,39.75) | 42.13(29.61,59.54) | 1.87(1.62,2.12) |
| Eritrea | female | 152.88(84.87,236.46) | 507.17(362.12,693.96) | 2.32 | 31.71(17.98,49.55) | 41.28(29.87,56.61) | 0.92(0.75,1.10) |
| Estonia | female | 42.24(37.08,49.26) | 74.94(58.82,94.58) | 0.77 | 3.12(2.76,3.65) | 4.05(3.14,5.15) | 0.57(-0.44,1.58) |
| Ethiopia | female | 3679.48(2634.02,4981.94) | 4650.19(3863.39,5445.34) | 0.26 | 43.81(31.15,58.10) | 27.79(23.20,31.95) | -1.93(-2.07,-1.79) |
| Micronesia (Federated States of) | female | 21.25(15.84,28.31) | 56.24(40.89,76.53) | 1.65 | 97.87(73.27,131.07) | 170.88(125.67,226.31) | 1.84(1.52,2.16) |
| Fiji | female | 249.06(194.44,353.73) | 878.25(700.20,1080.36) | 2.53 | 152.01(118.92,219.54) | 245.35(199.13,300.26) | 1.30(0.83,1.76) |
| Finland | female | 309.37(276.97,343.46) | 221.28(180.17,252.02) | -0.28 | 6.40(5.72,7.15) | 2.61(2.19,2.96) | -3.62(-3.98,-3.26) |
| France | female | 4163.53(3638.65,4609.55) | 6395.24(5039.36,7359.08) | 0.54 | 7.14(6.34,7.84) | 5.62(4.62,6.37) | -0.96(-1.52,-0.41) |
| Gabon | female | 102.20(77.67,135.02) | 214.53(158.27,287.21) | 1.10 | 36.37(27.57,48.02) | 43.90(32.63,58.00) | 0.65(0.19,1.11) |
| Georgia | female | 328.86(295.00,370.64) | 667.58(554.42,788.03) | 1.03 | 8.65(7.78,9.71) | 17.56(14.53,20.72) | 2.24(0.18,4.34) |
| Germany | female | 14038.39(12697.59,15068.22) | 11969.05(10047.33,13523.36) | -0.15 | 15.51(14.06,16.61) | 8.30(7.09,9.29) | -2.64(-2.93,-2.35) |
| Ghana | female | 851.85(662.39,1089.91) | 2491.97(1993.10,3149.14) | 1.93 | 30.55(24.01,38.75) | 32.63(26.39,40.58) | 0.04(-0.21,0.28) |
| Greece | female | 697.01(638.21,746.42) | 704.05(611.21,789.43) | 0.01 | 8.18(7.47,8.75) | 4.44(3.92,4.90) | -1.57(-2.01,-1.14) |
| Greenland | female | 2.23(1.75,2.68) | 1.75(1.37,2.20) | -0.21 | 15.12(11.86,18.04) | 5.99(4.72,7.49) | -4.37(-4.92,-3.82) |
| Grenada | female | 32.67(28.99,36.40) | 40.46(35.67,44.92) | 0.24 | 71.94(63.61,80.10) | 71.34(62.60,79.22) | 0.05(-0.12,0.23) |
| Guam | female | 14.79(11.86,17.28) | 24.23(19.78,30.13) | 0.64 | 49.31(39.88,58.04) | 24.36(19.95,30.46) | -3.05(-3.57,-2.52) |
| Guatemala | female | 300.50(258.01,343.45) | 3776.35(3026.83,4658.03) | 11.57 | 18.89(16.48,21.27) | 64.64(52.06,79.01) | 3.44(2.77,4.12) |
| Guinea | female | 463.42(347.01,601.44) | 1050.61(776.85,1342.08) | 1.27 | 31.02(23.10,40.02) | 42.74(31.62,54.29) | 1.25(1.11,1.39) |
| Guinea-Bissau | female | 61.93(42.84,85.00) | 165.89(118.76,220.79) | 1.68 | 33.80(23.44,45.40) | 49.48(35.78,65.53) | 1.37(1.12,1.62) |
| Guyana | female | 164.50(141.77,189.39) | 299.53(237.93,375.12) | 0.82 | 88.66(76.78,101.53) | 96.59(77.20,119.17) | -0.09(-0.61,0.43) |
| Haiti | female | 1698.05(1205.12,2194.16) | 2779.84(1830.36,4117.07) | 0.64 | 106.53(75.08,135.64) | 84.36(56.79,123.94) | -0.68(-0.76,-0.61) |
| Honduras | female | 125.36(101.61,151.17) | 574.15(434.58,762.42) | 3.58 | 11.86(9.57,14.38) | 18.93(14.51,24.75) | 1.66(1.33,2.00) |
| Hungary | female | 1102.63(1017.31,1170.70) | 1337.75(1103.19,1623.20) | 0.21 | 12.22(11.27,12.96) | 10.02(8.28,12.17) | -0.48(-0.76,-0.20) |
| Iceland | female | 7.23(6.19,8.24) | 8.07(6.33,9.54) | 0.12 | 4.12(3.56,4.65) | 2.13(1.72,2.50) | -2.79(-3.06,-2.52) |
| India | female | 32869.74(25657.87,44673.57) | 126099.24(102747.02,152591.41) | 2.84 | 19.79(15.34,27.09) | 24.27(19.64,29.32) | 0.58(0.32,0.84) |
| Indonesia | female | 15484.67(12915.72,18609.21) | 50442.94(39720.81,61997.43) | 2.26 | 31.02(25.86,37.61) | 46.99(37.68,56.82) | 1.75(1.60,1.90) |
| Iran (Islamic Republic of) | female | 1299.75(1044.84,1568.31) | 7666.57(5320.68,8526.79) | 4.90 | 13.84(10.86,16.86) | 24.23(17.22,27.04) | 2.21(1.99,2.42) |
| Iraq | female | 1972.69(1532.46,2429.73) | 4432.71(3491.08,5598.25) | 1.25 | 52.22(40.44,64.76) | 43.56(34.55,54.21) | -0.73(-0.81,-0.66) |
| Ireland | female | 210.51(193.00,227.87) | 218.59(183.16,250.83) | 0.04 | 8.87(8.09,9.57) | 4.81(4.08,5.48) | -2.21(-2.36,-2.06) |
| Israel | female | 441.93(405.81,474.48) | 1375.67(1144.23,1540.09) | 2.11 | 17.46(15.91,18.66) | 18.60(15.78,20.72) | -0.93(-1.92,0.08) |
| Italy | female | 11159.61(10314.08,11694.10) | 11787.23(9578.37,12989.86) | 0.06 | 19.74(18.21,20.73) | 10.34(8.64,11.29) | -2.35(-2.47,-2.22) |
| Jamaica | female | 710.16(656.30,756.93) | 1400.79(1148.83,1687.88) | 0.97 | 71.84(66.52,76.37) | 82.54(67.84,100.18) | -0.01(-0.32,0.31) |
| Japan | female | 5228.99(4736.74,5526.47) | 4363.90(3231.80,5060.11) | -0.17 | 5.39(4.86,5.71) | 1.43(1.13,1.61) | -4.55(-5.10,-3.98) |
| Jordan | female | 413.20(322.35,502.68) | 946.00(753.79,1165.35) | 1.29 | 81.36(64.14,99.16) | 42.28(34.16,51.74) | -2.85(-3.56,-2.14) |
| Kazakhstan | female | 574.71(531.39,620.39) | 1557.96(1328.69,1805.47) | 1.71 | 7.40(6.86,7.99) | 15.94(13.66,18.39) | 1.30(0.47,2.14) |
| Kenya | female | 799.33(670.38,937.43) | 2255.35(1710.22,2867.83) | 1.82 | 22.57(18.97,26.38) | 23.58(18.32,29.60) | 0.32(0.21,0.43) |
| Kiribati | female | 30.12(23.40,37.06) | 65.83(49.25,83.30) | 1.19 | 150.44(117.54,186.55) | 180.06(135.64,223.79) | 0.32(0.06,0.58) |
| Kuwait | female | 63.39(54.78,71.45) | 129.11(101.45,160.72) | 1.04 | 33.68(28.96,38.02) | 16.71(13.00,20.85) | -2.31(-2.89,-1.72) |
| Kyrgyzstan | female | 109.79(100.38,119.99) | 165.23(141.18,191.24) | 0.50 | 6.01(5.52,6.60) | 6.58(5.64,7.61) | -0.82(-1.28,-0.36) |
| Lao People's Democratic Republic | female | 422.31(288.13,599.31) | 831.06(573.90,1073.05) | 0.97 | 41.45(28.61,57.38) | 41.56(29.10,53.67) | -0.23(-0.33,-0.14) |
| Latvia | female | 110.61(101.96,120.98) | 213.24(164.51,268.35) | 0.93 | 4.74(4.37,5.19) | 7.34(5.67,9.33) | 1.52(1.06,1.98) |
| Lebanon | female | 178.30(147.45,216.13) | 328.04(244.21,455.10) | 0.84 | 17.65(14.50,21.52) | 11.59(8.66,16.07) | -1.58(-1.71,-1.46) |
| Lesotho | female | 245.18(184.57,308.59) | 721.26(477.81,990.55) | 1.94 | 46.36(35.11,58.04) | 106.70(71.52,144.58) | 4.12(3.56,4.69) |
| Liberia | female | 136.99(109.00,165.34) | 328.38(242.67,428.22) | 1.40 | 30.19(24.32,36.35) | 39.12(29.02,50.78) | 1.03(0.86,1.20) |
| Libya | female | 126.22(93.63,177.85) | 443.80(318.45,595.64) | 2.52 | 15.65(11.50,22.34) | 19.72(14.17,26.31) | 1.42(1.11,1.73) |
| Lithuania | female | 95.54(86.85,106.97) | 125.79(101.39,153.33) | 0.32 | 3.37(3.06,3.78) | 3.20(2.57,3.92) | -0.59(-0.81,-0.36) |
| Luxembourg | female | 34.56(30.62,38.21) | 35.18(28.47,42.02) | 0.02 | 9.97(8.84,10.97) | 5.14(4.24,6.14) | -2.34(-2.49,-2.19) |
| North Macedonia | female | 199.01(173.34,226.74) | 573.63(442.71,714.85) | 1.88 | 22.51(19.51,25.70) | 35.43(27.45,43.76) | 2.01(1.60,2.43) |
| Madagascar | female | 613.97(489.83,742.47) | 1285.04(956.33,1678.25) | 1.09 | 27.54(21.99,33.20) | 28.17(21.25,36.80) | -0.08(-0.21,0.06) |
| Malawi | female | 611.18(483.47,751.20) | 949.33(757.63,1169.43) | 0.55 | 35.34(28.02,43.23) | 27.29(21.94,33.69) | -1.04(-1.15,-0.94) |
| Malaysia | female | 1285.15(1098.79,1478.81) | 1690.69(1299.80,2569.25) | 0.32 | 29.93(25.49,34.44) | 14.09(10.88,21.51) | -3.27(-3.75,-2.79) |
| Maldives | female | 12.44(8.71,17.64) | 20.01(15.96,24.41) | 0.61 | 39.57(27.90,52.92) | 17.14(13.58,21.01) | -3.54(-3.97,-3.12) |
| Mali | female | 580.27(440.63,725.82) | 1538.11(1119.63,1927.88) | 1.65 | 31.99(24.19,39.91) | 43.36(31.09,53.84) | 0.99(0.78,1.20) |
| Malta | female | 58.39(52.84,63.90) | 61.76(50.57,72.44) | 0.06 | 24.45(22.04,26.77) | 10.18(8.44,11.91) | -3.04(-3.30,-2.77) |
| Marshall Islands | female | 6.75(5.30,8.50) | 21.10(15.05,28.49) | 2.13 | 88.41(69.23,112.38) | 142.64(104.54,186.48) | 1.50(1.17,1.84) |
| Mauritania | female | 169.64(132.60,215.33) | 362.98(263.19,464.30) | 1.14 | 35.33(27.47,44.40) | 40.58(29.83,51.69) | 0.53(0.33,0.73) |
| Mauritius | female | 165.63(152.26,180.31) | 893.58(727.40,1078.84) | 4.40 | 42.65(39.23,46.26) | 95.02(77.36,115.05) | 4.66(3.68,5.64) |
| Mexico | female | 14521.42(13817.16,15050.59) | 35804.19(30031.12,43214.83) | 1.47 | 71.10(67.16,74.06) | 59.61(50.13,71.64) | -0.83(-1.01,-0.64) |
| Republic of Moldova | female | 178.88(167.40,191.65) | 155.16(131.86,181.99) | -0.13 | 6.76(6.31,7.24) | 4.38(3.71,5.13) | -2.43(-2.86,-2.00) |
| Mongolia | female | 20.35(15.72,28.96) | 43.06(32.20,57.88) | 1.12 | 3.67(2.85,5.10) | 3.69(2.83,4.91) | -0.26(-0.60,0.09) |
| Montenegro | female | 40.13(33.11,47.64) | 73.45(60.20,91.42) | 0.83 | 11.76(9.69,13.94) | 13.03(10.74,16.07) | 0.52(0.29,0.74) |
| Morocco | female | 913.18(678.34,1472.81) | 3588.23(2707.23,5032.41) | 2.93 | 14.85(11.03,24.80) | 25.22(19.30,35.99) | 2.38(2.11,2.64) |
| Mozambique | female | 801.57(615.89,1022.61) | 1610.80(1191.38,2207.03) | 1.01 | 30.56(23.73,38.17) | 31.57(23.74,43.08) | 0.26(0.05,0.46) |
| Myanmar | female | 6128.60(4427.13,8166.58) | 10260.75(8714.41,11983.50) | 0.67 | 52.53(38.53,68.67) | 43.49(36.80,50.50) | -0.84(-0.93,-0.76) |
| Namibia | female | 208.59(161.09,262.35) | 410.16(302.39,553.60) | 0.97 | 56.79(43.91,70.94) | 55.49(40.97,74.34) | -0.48(-0.91,-0.05) |
| Nepal | female | 343.09(245.42,477.27) | 1595.37(1235.58,2018.15) | 3.65 | 10.75(7.51,14.34) | 19.48(14.50,24.63) | 2.22(1.97,2.48) |
| Netherlands | female | 2139.03(1898.77,2321.65) | 1753.93(1478.44,1990.01) | -0.18 | 16.55(14.73,17.92) | 7.56(6.45,8.52) | -3.19(-3.60,-2.79) |
| New Zealand | female | 200.60(182.52,219.01) | 300.44(257.67,334.31) | 0.50 | 9.07(8.23,9.86) | 6.41(5.55,7.11) | -2.01(-2.46,-1.55) |
| Nicaragua | female | 205.91(182.61,231.55) | 1004.19(854.39,1175.25) | 3.88 | 26.11(23.01,29.43) | 45.44(38.85,52.54) | 1.45(0.81,2.08) |
| Niger | female | 271.27(194.74,362.82) | 1124.75(829.26,1440.25) | 3.15 | 24.58(17.51,32.21) | 34.79(25.75,43.65) | 1.15(0.92,1.37) |
| Nigeria | female | 5007.08(3845.47,6372.10) | 10562.75(7709.33,13971.20) | 1.11 | 26.11(20.18,32.85) | 29.17(21.85,37.54) | 0.44(0.32,0.55) |
| Democratic People's Republic of Korea | female | 1262.00(942.44,1702.90) | 2417.44(1876.95,3164.63) | 0.92 | 13.82(10.42,18.71) | 12.60(9.77,16.45) | -0.27(-0.41,-0.13) |
| Northern Mariana Islands | female | 3.41(2.65,4.40) | 11.74(9.60,14.83) | 2.45 | 58.05(45.17,73.82) | 52.96(43.51,65.91) | -0.07(-0.44,0.30) |
| Norway | female | 270.73(235.10,291.11) | 321.94(268.49,355.80) | 0.19 | 5.81(5.10,6.24) | 4.68(3.98,5.12) | -0.82(-1.17,-0.47) |
| Oman | female | 123.39(89.15,164.95) | 284.01(235.59,337.22) | 1.30 | 48.45(35.13,64.34) | 54.10(44.46,66.00) | 0.58(0.40,0.76) |
| Pakistan | female | 5096.94(3551.46,6613.94) | 24629.72(15891.42,32398.81) | 3.83 | 22.20(15.18,28.85) | 55.23(35.89,71.14) | 3.32(3.00,3.65) |
| Palestine | female | 237.50(186.06,297.50) | 658.45(553.39,764.46) | 1.77 | 56.55(44.45,70.91) | 67.01(56.64,77.80) | 0.59(0.12,1.06) |
| Panama | female | 154.62(138.48,169.28) | 637.06(496.43,785.65) | 3.12 | 21.80(19.45,23.86) | 28.68(22.38,35.62) | 0.67(0.37,0.97) |
| Papua New Guinea | female | 392.38(285.02,571.58) | 1506.11(1131.89,2045.41) | 2.84 | 46.66(33.61,68.90) | 72.45(54.94,96.38) | 1.49(1.30,1.68) |
| Paraguay | female | 291.36(248.61,336.00) | 1313.53(989.66,1677.89) | 3.51 | 26.22(22.33,30.20) | 46.42(35.00,58.91) | 2.09(1.58,2.60) |
| Peru | female | 766.78(658.48,920.95) | 2383.23(1779.83,3122.26) | 2.11 | 13.40(11.48,16.09) | 14.26(10.64,18.73) | 0.15(-0.16,0.47) |
| Philippines | female | 3906.43(3450.36,4560.59) | 13089.93(10202.89,16612.57) | 2.35 | 34.89(30.38,40.39) | 35.24(27.73,44.50) | 0.16(-0.04,0.36) |
| Poland | female | 3350.30(3126.05,3586.18) | 4175.28(3294.34,5100.71) | 0.25 | 12.51(11.66,13.39) | 8.83(6.96,10.84) | -1.01(-1.36,-0.65) |
| Portugal | female | 1666.92(1550.00,1772.57) | 2426.26(1984.43,2732.47) | 0.46 | 19.98(18.54,21.23) | 13.27(11.19,14.78) | -2.05(-2.40,-1.71) |
| Puerto Rico | female | 871.78(786.73,932.76) | 1660.70(1294.76,2075.54) | 0.90 | 45.19(40.71,48.36) | 35.61(27.99,44.88) | -1.33(-1.59,-1.06) |
| Qatar | female | 26.26(20.84,32.29) | 108.98(84.40,139.96) | 3.15 | 111.86(88.74,138.66) | 173.19(134.57,213.10) | 2.39(1.64,3.15) |
| Romania | female | 1004.15(936.32,1077.97) | 1218.62(992.83,1460.84) | 0.21 | 6.40(5.97,6.87) | 5.20(4.24,6.26) | -0.75(-1.00,-0.50) |
| Russian Federation | female | 5550.24(5177.07,5779.90) | 12750.09(10665.23,14915.18) | 1.30 | 4.60(4.28,4.79) | 8.05(6.74,9.44) | 1.51(0.71,2.32) |
| Rwanda | female | 593.61(426.17,801.66) | 876.20(655.27,1188.02) | 0.48 | 41.89(29.81,56.58) | 30.73(23.23,41.62) | -1.62(-1.87,-1.37) |
| Saint Lucia | female | 44.26(40.43,48.01) | 68.59(56.84,80.81) | 0.55 | 92.17(84.11,99.87) | 59.77(49.56,70.51) | -2.61(-3.04,-2.18) |
| Saint Vincent and the Grenadines | female | 40.96(37.04,44.59) | 55.27(47.90,63.48) | 0.35 | 102.27(92.49,111.03) | 86.09(74.53,98.78) | -0.98(-1.27,-0.69) |
| Samoa | female | 26.67(20.34,38.32) | 65.10(49.52,85.66) | 1.44 | 63.29(48.45,91.50) | 92.33(71.47,122.18) | 1.26(0.93,1.60) |
| Sao Tome and Principe | female | 2.77(2.23,3.28) | 5.16(4.05,6.66) | 0.86 | 9.00(7.32,10.64) | 11.22(8.85,14.53) | 0.61(0.46,0.75) |
| Saudi Arabia | female | 579.43(429.05,756.10) | 1029.23(793.57,1369.04) | 0.78 | 26.15(18.98,34.02) | 17.62(13.83,23.19) | -2.13(-2.51,-1.75) |
| Senegal | female | 364.60(286.32,471.25) | 1373.37(1025.40,1762.68) | 2.77 | 26.49(20.72,34.09) | 40.80(30.79,52.14) | 1.66(1.42,1.89) |
| Serbia | female | 1131.77(901.08,1322.08) | 1970.88(1575.92,2429.90) | 0.74 | 20.53(16.33,23.90) | 20.86(16.88,25.51) | 0.19(-0.04,0.42) |
| Seychelles | female | 4.73(4.03,5.56) | 13.57(11.28,16.04) | 1.87 | 14.10(12.02,16.55) | 24.69(20.46,29.04) | 2.03(1.87,2.20) |
| Sierra Leone | female | 183.11(138.14,232.77) | 601.78(443.33,794.82) | 2.29 | 22.00(16.68,27.77) | 38.26(27.87,50.54) | 2.26(1.94,2.58) |
| Singapore | female | 221.95(205.05,236.91) | 88.65(73.63,100.65) | -0.60 | 20.19(18.52,21.62) | 2.18(1.81,2.47) | -7.92(-9.14,-6.69) |
| Slovakia | female | 435.41(383.76,489.33) | 431.76(334.52,542.82) | -0.01 | 12.15(10.78,13.61) | 7.48(5.81,9.42) | -1.66(-1.83,-1.49) |
| Slovenia | female | 161.61(125.27,200.98) | 179.54(133.06,229.21) | 0.11 | 10.64(8.30,13.22) | 5.61(4.20,7.25) | -4.48(-5.41,-3.54) |
| Solomon Islands | female | 51.74(36.14,91.88) | 214.64(170.42,271.70) | 3.15 | 81.04(56.01,144.83) | 139.13(113.04,172.75) | 1.81(1.60,2.01) |
| Somalia | female | 411.55(267.14,583.98) | 1156.63(806.63,1616.58) | 1.81 | 36.35(24.18,50.15) | 37.31(26.43,51.25) | 0.39(0.29,0.50) |
| South Africa | female | 4879.86(4088.55,5650.38) | 15830.85(14095.75,17479.57) | 2.24 | 43.14(35.93,49.90) | 66.04(58.83,73.05) | 1.93(1.39,2.46) |
| Republic of Korea | female | 2177.78(1983.02,3251.81) | 6344.80(4993.31,7477.17) | 1.91 | 13.90(12.53,22.49) | 12.10(9.62,14.16) | -1.50(-2.49,-0.50) |
| South Sudan | female | 290.66(212.40,394.08) | 415.09(292.32,556.47) | 0.43 | 30.65(22.62,40.91) | 28.47(20.66,37.37) | -0.18(-0.24,-0.13) |
| Spain | female | 6173.28(5488.93,6653.93) | 5989.12(4714.62,6912.28) | -0.03 | 18.30(16.19,19.71) | 7.26(5.91,8.23) | -3.52(-3.70,-3.35) |
| Sri Lanka | female | 957.44(805.97,1181.11) | 6875.84(4730.67,8993.94) | 6.18 | 21.52(18.10,25.86) | 53.46(36.42,70.59) | 3.64(3.14,4.14) |
| Sudan | female | 520.03(343.91,825.32) | 1235.18(811.25,1831.49) | 1.38 | 13.01(8.69,21.10) | 16.59(11.00,24.44) | 1.21(1.03,1.39) |
| Suriname | female | 49.74(44.13,55.64) | 131.19(108.86,158.58) | 1.64 | 37.61(33.28,42.23) | 41.24(34.21,49.95) | 0.34(0.03,0.64) |
| Eswatini | female | 112.11(81.30,141.49) | 335.51(207.06,482.44) | 1.99 | 76.62(55.44,96.50) | 111.73(70.21,158.18) | 1.90(1.21,2.59) |
| Sweden | female | 738.81(640.33,820.70) | 963.42(802.09,1085.89) | 0.30 | 7.36(6.45,8.08) | 6.34(5.44,7.11) | -0.51(-0.71,-0.31) |
| Switzerland | female | 941.53(831.37,1021.24) | 737.25(587.00,845.29) | -0.22 | 12.88(11.44,13.90) | 5.41(4.43,6.14) | -3.79(-4.12,-3.46) |
| Syrian Arab Republic | female | 502.57(375.17,640.84) | 850.41(645.38,1135.91) | 0.69 | 23.01(17.11,29.40) | 19.66(15.19,26.36) | -1.28(-1.66,-0.91) |
| Taiwan (Province of China) | female | 2506.84(2320.66,2668.68) | 5348.72(4187.29,6661.20) | 1.13 | 37.11(34.23,39.61) | 24.22(18.91,30.24) | -2.70(-3.34,-2.06) |
| Tajikistan | female | 169.10(149.86,192.12) | 726.27(476.33,912.21) | 3.29 | 10.85(9.59,12.34) | 29.79(19.57,36.72) | 4.10(3.71,4.49) |
| United Republic of Tanzania | female | 1505.05(1213.15,1807.60) | 3523.91(2813.10,4336.45) | 1.34 | 31.75(25.74,37.76) | 31.72(25.67,38.93) | 0.10(-0.04,0.24) |
| Thailand | female | 4774.03(3906.22,5749.31) | 10468.52(8020.47,13507.34) | 1.19 | 27.34(22.31,33.10) | 18.80(14.37,24.28) | -2.07(-2.38,-1.75) |
| Bahamas | female | 42.52(37.70,47.70) | 74.73(62.57,90.64) | 0.76 | 51.63(45.76,57.97) | 37.35(31.20,45.42) | -1.56(-1.78,-1.34) |
| Gambia | female | 31.13(22.93,40.18) | 168.20(120.19,219.05) | 4.40 | 22.03(16.57,28.19) | 37.89(27.59,48.82) | 1.83(1.59,2.06) |
| Timor-Leste | female | 33.42(23.77,46.49) | 104.67(77.33,136.08) | 2.13 | 27.91(20.44,37.62) | 28.67(21.36,37.17) | 0.02(-0.18,0.22) |
| Togo | female | 137.02(107.62,171.21) | 538.18(389.90,710.93) | 2.93 | 25.07(19.57,30.99) | 31.69(23.32,41.45) | 0.84(0.72,0.97) |
| Tonga | female | 29.01(23.61,34.41) | 44.59(35.37,56.21) | 0.54 | 107.00(87.16,126.93) | 104.54(83.29,131.92) | 0.05(-0.04,0.14) |
| Trinidad and Tobago | female | 524.28(494.45,555.46) | 868.68(676.20,1093.30) | 0.66 | 123.08(115.82,130.57) | 88.92(69.00,112.22) | -1.58(-1.79,-1.37) |
| Tunisia | female | 238.19(182.29,405.60) | 859.66(614.65,1218.29) | 2.61 | 11.60(8.88,20.14) | 14.13(10.13,20.21) | 0.90(0.79,1.01) |
| Turkey | female | 7842.29(6011.44,9297.82) | 10881.97(8620.37,13505.97) | 0.39 | 46.19(35.35,55.20) | 24.05(19.05,29.90) | -1.97(-2.35,-1.59) |
| Turkmenistan | female | 102.27(92.42,110.93) | 352.50(276.76,444.03) | 2.45 | 9.17(8.31,9.93) | 15.85(12.61,19.98) | 1.24(0.85,1.64) |
| Uganda | female | 794.05(579.54,1071.45) | 1954.81(1526.07,2551.39) | 1.46 | 28.49(20.83,38.19) | 29.84(23.42,38.36) | -0.04(-0.23,0.15) |
| Ukraine | female | 1695.53(1569.65,1798.09) | 1390.29(1098.15,1722.98) | -0.18 | 3.66(3.39,3.89) | 2.90(2.28,3.60) | -1.98(-2.36,-1.60) |
| United Arab Emirates | female | 69.39(50.00,91.07) | 245.68(181.95,328.83) | 2.54 | 85.68(58.34,114.38) | 51.24(37.98,67.48) | -1.23(-2.62,0.17) |
| United Kingdom | female | 4172.20(3790.40,4366.32) | 3164.24(2742.99,3423.46) | -0.24 | 7.06(6.42,7.38) | 3.73(3.29,4.01) | -2.42(-2.63,-2.21) |
| United States of America | female | 25351.51(23069.60,26626.94) | 34581.49(30910.37,36803.32) | 0.36 | 12.87(11.85,13.46) | 10.38(9.44,11.01) | -1.45(-1.93,-0.96) |
| Uruguay | female | 375.82(345.02,402.15) | 463.85(401.70,515.98) | 0.23 | 16.12(14.82,17.26) | 12.52(11.07,13.76) | -0.82(-0.98,-0.66) |
| Uzbekistan | female | 508.27(452.38,559.98) | 3743.22(3087.15,4481.99) | 6.36 | 7.79(6.95,8.55) | 33.64(28.19,39.68) | 4.97(4.25,5.70) |
| Vanuatu | female | 12.52(8.10,21.92) | 65.81(45.51,91.43) | 4.25 | 47.99(31.47,83.86) | 87.91(60.96,119.82) | 2.15(2.01,2.28) |
| Venezuela (Bolivarian Republic of) | female | 1619.59(1503.81,1715.95) | 5168.21(4051.53,6715.32) | 2.19 | 34.04(31.37,36.12) | 34.08(26.67,44.10) | -0.28(-0.59,0.04) |
| Viet nam | female | 7959.23(6030.59,9894.53) | 16737.36(12589.80,20634.55) | 1.10 | 35.53(26.94,44.06) | 34.52(25.79,42.78) | -0.06(-0.30,0.18) |
| Virginia | female | 495.80(447.26,542.74) | 859.02(681.87,1059.89) | 0.73 | 11.67(10.60,12.72) | 10.41(8.18,12.92) | -1.16(-1.68,-0.63) |
| Yemen | female | 288.69(181.27,491.44) | 970.01(619.22,1454.14) | 2.36 | 12.82(8.20,21.61) | 16.54(10.79,24.42) | 1.16(1.01,1.31) |
| Zambia | female | 432.47(342.89,531.86) | 928.26(729.93,1197.25) | 1.15 | 37.46(29.77,46.22) | 32.10(25.54,41.21) | -0.78(-0.89,-0.67) |
| Zimbabwe | female | 728.28(610.67,865.35) | 2127.92(1570.61,2768.13) | 1.92 | 40.03(33.67,47.56) | 60.95(45.15,78.26) | 2.67(2.09,3.25) |
| Monaco | female | 1.24(0.91,1.62) | 1.46(1.07,1.83) | 0.18 | 2.38(1.74,3.10) | 2.11(1.56,2.67) | -0.23(-0.34,-0.13) |
| San Marino | female | 1.26(0.99,1.56) | 2.28(1.50,3.27) | 0.81 | 6.43(5.08,8.01) | 4.87(3.20,7.19) | -0.64(-0.82,-0.46) |
| Saint Kitts and Nevis | female | 16.12(14.28,18.06) | 14.83(12.47,17.58) | -0.08 | 76.13(67.98,84.80) | 48.80(41.19,57.80) | -1.01(-1.22,-0.80) |
| Cook Islands | female | 7.05(5.81,8.77) | 13.41(10.87,16.46) | 0.90 | 125.63(103.41,157.09) | 107.04(86.78,131.63) | -0.59(-0.69,-0.49) |
| Nauru | female | 1.88(1.44,2.46) | 3.12(2.12,4.68) | 0.66 | 122.78(96.84,154.93) | 168.39(118.94,241.86) | 0.98(0.74,1.22) |
| Niue | female | 1.23(0.95,1.57) | 1.42(0.98,1.98) | 0.16 | 89.73(68.98,114.91) | 117.26(80.96,163.19) | 0.74(0.51,0.97) |
| Palau | female | 3.76(2.79,4.90) | 11.04(7.77,14.24) | 1.93 | 83.15(61.41,106.64) | 117.46(82.23,147.99) | 1.18(0.97,1.40) |
| Tokelau | female | 0.72(0.55,0.93) | 0.82(0.57,1.10) | 0.13 | 99.73(75.72,128.23) | 127.29(89.77,169.00) | 0.76(0.56,0.96) |
| Tuvalu | female | 3.62(2.82,4.62) | 6.67(4.61,9.01) | 0.84 | 98.39(77.06,124.26) | 130.80(90.57,176.90) | 0.89(0.64,1.15) |

**Supplementary Table 5.** The DALYs and age-standardized DALY rate of type 2 diabetes in 1990 and 2019, and its temporal trends from 1990 to 2019.

| **Nation** | **Sex** | **DALY Cases No. (95% UI)** | | **Change in absolute number (%)** | **Age-standardized DALY rate per 100,000 No.(95% UI)** | | **1990-2019 EAPC No. (95%CI)** |
| --- | --- | --- | --- | --- | --- | --- | --- |
|  |  | **1990** | **2019** |  | **1990** | **2019** |  |
| Afghanistan | both | 73967.40(52412.08,102292.66) | 222032.13(160451.07,295439.40) | 2.00 | 997.07(713.44,1373.48) | 1567.90(1136.29,2073.00) | 1.83(1.74,1.93) |
| Albania | both | 5621.01(4259.80,7327.02) | 14435.15(10357.18,19510.14) | 1.57 | 262.47(200.38,344.13) | 348.75(249.28,469.82) | 0.74(0.60,0.88) |
| Algeria | both | 79090.35(58771.45,102411.52) | 329574.41(249708.43,430894.76) | 3.17 | 641.35(485.37,828.73) | 951.02(727.94,1234.63) | 1.61(1.45,1.78) |
| American Samoa | both | 677.09(569.69,802.09) | 1867.96(1541.41,2246.78) | 1.76 | 2704.43(2302.73,3174.36) | 3725.45(3080.28,4463.18) | 1.02(0.75,1.29) |
| Andorra | both | 157.47(120.07,203.69) | 532.00(392.92,706.47) | 2.38 | 285.71(219.81,367.04) | 387.41(283.86,514.19) | 1.02(0.95,1.08) |
| Angola | both | 53707.71(42485.70,65863.87) | 159151.93(128093.85,195515.95) | 1.96 | 1261.06(1014.69,1533.61) | 1306.91(1068.72,1593.27) | 0.13(0.07,0.18) |
| Antigua and Barbuda | both | 893.60(792.89,1014.30) | 1953.50(1613.13,2327.12) | 1.19 | 1715.76(1519.03,1946.89) | 1905.94(1573.74,2245.33) | 0.06(-0.06,0.17) |
| Argentina | both | 207836.36(182197.39,239081.80) | 395929.49(326727.90,479158.84) | 0.91 | 640.37(561.85,737.27) | 746.30(614.04,908.90) | 0.09(-0.10,0.27) |
| Armenia | both | 18733.49(16085.19,21990.69) | 44262.13(36382.30,53197.91) | 1.36 | 665.08(571.64,779.61) | 1064.57(874.06,1278.05) | 1.00(0.49,1.52) |
| Australia | both | 70348.64(59187.93,83500.20) | 169763.78(129792.73,216150.69) | 1.41 | 363.09(305.52,430.29) | 424.76(321.71,543.92) | 0.31(0.14,0.47) |
| Austria | both | 44435.82(37715.53,52755.57) | 82714.35(65386.11,104086.28) | 0.86 | 378.80(319.40,453.07) | 486.96(378.24,623.07) | 1.03(0.73,1.33) |
| Azerbaijan | both | 26943.05(22219.50,32388.80) | 99252.51(79600.52,121570.54) | 2.68 | 503.39(416.63,603.08) | 971.34(781.15,1180.46) | 1.94(1.60,2.27) |
| Bahrain | both | 3654.37(3058.41,4311.35) | 31385.13(25063.59,39005.52) | 7.59 | 2067.56(1751.71,2415.78) | 3232.51(2622.36,3929.33) | 1.63(1.27,1.99) |
| Bangladesh | both | 374387.58(310284.00,445312.68) | 1095929.08(886460.80,1350362.80) | 1.93 | 763.07(642.33,893.14) | 845.12(688.09,1036.22) | 0.30(-0.05,0.65) |
| Barbados | both | 5338.91(4745.49,5968.36) | 8864.99(7329.82,10542.48) | 0.66 | 1876.03(1659.17,2111.32) | 1860.31(1540.38,2213.44) | -0.40(-0.54,-0.27) |
| Belarus | both | 38291.25(29521.70,48521.51) | 41993.51(29620.20,57037.92) | 0.10 | 298.94(229.87,379.04) | 278.74(194.90,378.08) | -0.82(-1.06,-0.58) |
| Belgium | both | 60043.28(48165.49,74142.44) | 93817.86(69033.99,123339.46) | 0.56 | 398.58(318.01,496.41) | 451.98(327.19,600.27) | 0.23(0.16,0.30) |
| Belize | both | 1195.10(1049.56,1370.31) | 5441.96(4636.84,6381.05) | 3.55 | 1253.46(1100.77,1424.86) | 1848.42(1584.04,2154.92) | 1.29(0.82,1.75) |
| Benin | both | 15402.80(12957.42,17965.23) | 54012.57(43137.33,67308.93) | 2.51 | 761.66(641.65,884.40) | 1066.02(852.93,1312.41) | 1.17(0.96,1.37) |
| Bermuda | both | 595.18(510.47,686.95) | 967.15(766.70,1204.43) | 0.62 | 948.20(814.69,1092.02) | 795.16(626.74,989.99) | -0.84(-0.97,-0.71) |
| Bhutan | both | 1635.35(1252.95,2038.45) | 5527.52(4379.63,6948.82) | 2.38 | 622.88(484.13,767.65) | 968.29(768.86,1201.62) | 1.62(1.56,1.67) |
| Bolivia (Plurinational State of) | both | 37881.74(31456.42,46644.34) | 119117.54(94785.14,147128.94) | 2.14 | 1143.59(954.12,1403.81) | 1329.88(1059.82,1638.89) | 0.42(0.36,0.47) |
| Bosnia and Herzegovina | both | 24729.32(20005.23,30222.80) | 76453.42(60311.49,95076.85) | 2.09 | 602.25(489.43,728.24) | 1293.93(1026.16,1598.44) | 3.29(2.89,3.69) |
| Botswana | both | 9010.29(7143.51,11424.92) | 32917.26(25035.94,42044.93) | 2.65 | 1549.09(1243.24,1950.17) | 2377.17(1843.02,3018.34) | 1.29(0.88,1.69) |
| Brazil | both | 1016119.89(889834.22,1159971.05) | 2414756.81(2046263.26,2836619.02) | 1.38 | 1090.59(959.53,1243.90) | 1012.05(859.16,1187.55) | -0.19(-0.25,-0.13) |
| Brunei Darussalam | both | 3035.29(2592.56,3607.32) | 7548.87(6239.65,9113.68) | 1.49 | 2987.99(2579.27,3512.69) | 2343.21(1977.99,2775.80) | -0.52(-0.73,-0.31) |
| Bulgaria | both | 80050.07(66415.44,95412.82) | 98744.56(76662.42,124008.17) | 0.23 | 638.40(527.55,764.81) | 729.60(564.13,924.46) | 0.05(-0.16,0.26) |
| Burkina Faso | both | 40621.22(33552.06,48357.94) | 102745.52(84231.86,125159.34) | 1.53 | 925.92(776.28,1088.27) | 1067.18(883.65,1286.26) | 0.40(0.31,0.49) |
| Burundi | both | 29993.57(23615.03,39134.69) | 49967.81(39707.78,61713.89) | 0.67 | 1230.31(976.24,1593.52) | 1035.58(830.77,1261.30) | -0.88(-0.99,-0.77) |
| Cambodia | both | 45541.26(36246.30,55318.26) | 142498.31(113436.00,177725.53) | 2.13 | 929.78(752.60,1117.71) | 1158.86(928.72,1436.52) | 0.64(0.50,0.78) |
| Cameroon | both | 41693.61(33600.58,51060.55) | 166249.45(129974.08,212737.40) | 2.99 | 920.28(746.30,1124.67) | 1341.00(1069.59,1686.05) | 1.31(0.98,1.64) |
| Canada | both | 105856.81(92058.18,122238.68) | 264599.29(207302.71,330044.13) | 1.50 | 326.15(283.96,376.39) | 399.67(310.63,503.38) | 0.02(-0.28,0.32) |
| Cabo Verde | both | 954.22(751.77,1195.24) | 4464.21(3656.46,5392.64) | 3.68 | 416.66(327.34,520.12) | 1042.22(857.42,1260.52) | 2.83(2.38,3.28) |
| Central African Republic | both | 19111.56(15936.84,23341.78) | 40123.77(31251.02,51382.14) | 1.10 | 1485.24(1244.39,1772.98) | 1628.13(1287.29,2050.82) | 0.40(0.27,0.52) |
| Chad | both | 20094.75(16287.16,24234.52) | 60320.31(47191.56,74486.05) | 2.00 | 705.45(571.89,844.53) | 1029.27(813.57,1266.33) | 1.29(0.99,1.58) |
| Chile | both | 57766.82(48392.86,68803.73) | 170569.88(132942.18,213283.00) | 1.95 | 573.86(482.66,683.07) | 711.64(555.77,890.77) | 0.71(0.57,0.85) |
| China | both | 3914032.98(3129814.53,4846143.20) | 9603362.57(7588720.87,11904722.76) | 1.45 | 435.81(351.04,536.33) | 476.52(376.36,589.46) | 0.42(0.19,0.65) |
| Colombia | both | 173516.88(139966.20,210509.73) | 465298.67(351442.88,596714.10) | 1.68 | 934.99(758.44,1139.52) | 881.63(666.30,1132.55) | -0.76(-1.00,-0.52) |
| Comoros | both | 2170.82(1423.11,2703.77) | 4716.70(3864.63,5775.28) | 1.17 | 968.03(661.58,1196.71) | 958.80(793.92,1164.61) | -0.19(-0.30,-0.07) |
| Congo | both | 18759.23(15645.64,22128.86) | 43654.66(34901.66,54371.06) | 1.33 | 1650.39(1389.19,1943.70) | 1554.89(1267.41,1899.57) | -0.33(-0.42,-0.24) |
| Costa Rica | both | 14407.08(11548.60,17814.28) | 45854.30(34104.95,60168.69) | 2.18 | 797.18(642.06,984.57) | 884.49(658.04,1159.42) | 0.02(-0.19,0.24) |
| C么te d'Ivoire | both | 37092.13(31004.12,44113.81) | 126030.35(100322.34,155404.21) | 2.40 | 880.82(747.13,1033.58) | 1136.21(924.73,1372.41) | 0.64(0.32,0.96) |
| Croatia | both | 36085.24(28752.37,45057.27) | 57459.42(43377.09,74486.64) | 0.59 | 566.88(451.94,705.85) | 698.24(522.38,909.46) | 0.42(0.31,0.53) |
| Cuba | both | 101903.17(83215.15,123539.02) | 159686.26(118660.76,209676.40) | 0.57 | 986.64(804.40,1196.72) | 885.52(655.69,1164.86) | -0.60(-0.97,-0.22) |
| Cyprus | both | 10093.88(8507.42,11891.24) | 15272.99(12285.48,18868.45) | 0.51 | 1282.68(1081.34,1499.30) | 794.82(639.92,979.27) | -2.18(-2.37,-1.99) |
| Czechia | both | 90552.59(70430.10,114256.33) | 200117.72(149259.20,259901.12) | 1.21 | 666.83(516.54,842.42) | 1016.48(754.02,1325.47) | 1.89(1.56,2.22) |
| Democratic Republic of the Congo | both | 195539.61(161132.11,235804.40) | 461009.87(368884.68,571785.47) | 1.36 | 1185.23(989.20,1406.12) | 1185.93(959.64,1445.99) | -0.04(-0.08,0.00) |
| Denmark | both | 23129.60(20063.22,26773.67) | 44297.21(36523.34,53849.01) | 0.92 | 303.80(261.23,353.66) | 417.49(338.85,512.55) | 1.04(0.75,1.34) |
| Djibouti | both | 1374.33(1073.45,1760.93) | 7231.73(5533.15,9284.09) | 4.26 | 909.31(720.99,1147.88) | 1152.98(915.15,1441.32) | 0.84(0.76,0.93) |
| Dominica | both | 1318.13(1152.42,1506.18) | 1912.09(1560.48,2323.31) | 0.45 | 1899.30(1645.80,2173.34) | 2164.47(1766.98,2632.85) | 0.27(0.17,0.36) |
| Dominican Republic | both | 28404.03(24169.93,32944.30) | 107868.07(85147.72,133559.81) | 2.80 | 700.53(594.52,811.95) | 1119.08(885.02,1385.13) | 1.92(1.77,2.06) |
| Ecuador | both | 40983.12(35493.75,47595.84) | 177086.35(143588.55,216421.19) | 3.32 | 745.95(646.34,865.70) | 1162.50(945.17,1418.15) | 1.45(1.18,1.71) |
| Egypt | both | 219146.36(193502.16,248721.31) | 835076.15(661795.12,1022770.12) | 2.81 | 718.24(637.23,811.60) | 1224.68(973.01,1492.76) | 2.16(2.05,2.28) |
| El Salvador | both | 23271.30(19322.59,27613.92) | 88054.50(69429.14,108503.05) | 2.78 | 755.90(627.64,892.76) | 1501.09(1182.06,1851.70) | 2.37(2.14,2.60) |
| Equatorial Guinea | both | 2844.89(2231.87,3514.24) | 7944.96(5936.01,10419.09) | 1.79 | 1341.19(1062.01,1644.05) | 1542.28(1176.92,1986.46) | 0.67(0.52,0.83) |
| Eritrea | both | 12255.66(9390.43,16509.43) | 36688.21(28447.41,47124.75) | 1.99 | 1096.24(839.92,1450.56) | 1267.83(1001.29,1610.58) | 0.43(0.30,0.57) |
| Estonia | both | 5827.25(4342.92,7555.02) | 9681.81(7068.12,12796.71) | 0.66 | 291.41(217.31,379.71) | 421.84(305.48,562.88) | 1.27(0.85,1.69) |
| Ethiopia | both | 330789.96(272887.48,418617.23) | 381716.03(324570.12,441099.82) | 0.15 | 1531.92(1276.62,1908.91) | 903.25(777.51,1033.07) | -2.16(-2.29,-2.02) |
| Micronesia (Federated States of) | both | 1366.34(1085.40,1694.60) | 3823.70(2802.01,5283.42) | 1.80 | 2718.24(2192.34,3344.85) | 4896.31(3682.18,6620.37) | 1.97(1.62,2.32) |
| Fiji | both | 17200.41(14182.24,21044.23) | 54197.12(44219.13,65144.97) | 2.15 | 4357.64(3605.72,5323.40) | 6884.30(5667.75,8214.82) | 1.25(0.86,1.63) |
| Finland | both | 25966.52(19977.87,33117.16) | 50288.11(35951.04,67596.60) | 0.94 | 376.06(288.46,478.65) | 489.43(343.80,664.06) | 0.96(0.85,1.07) |
| France | both | 184657.90(156665.56,216536.38) | 356113.98(289357.58,435072.25) | 0.93 | 225.56(190.72,266.32) | 278.20(220.64,345.74) | 0.64(0.37,0.91) |
| Gabon | both | 9878.30(7766.40,12567.08) | 20672.69(16597.46,25313.50) | 1.09 | 1713.47(1350.08,2169.36) | 1889.11(1534.59,2301.87) | 0.33(0.16,0.50) |
| Georgia | both | 30091.38(24380.03,36480.65) | 57554.56(45882.05,71083.74) | 0.91 | 479.39(387.70,583.29) | 1012.31(801.58,1256.14) | 2.26(1.43,3.11) |
| Germany | both | 695998.57(574869.28,847430.19) | 1032452.88(786681.48,1323075.41) | 0.48 | 558.52(457.13,685.65) | 602.22(453.18,783.66) | -0.19(-0.49,0.11) |
| Ghana | both | 53997.86(43977.36,65159.61) | 215388.17(176365.91,262522.97) | 2.99 | 834.94(689.93,989.03) | 1287.81(1065.04,1573.46) | 1.88(1.51,2.26) |
| Greece | both | 51430.52(40125.51,64853.35) | 93510.60(68148.12,123137.31) | 0.82 | 342.07(265.27,429.19) | 456.55(329.62,610.28) | 1.07(0.98,1.15) |
| Greenland | both | 158.66(135.77,186.06) | 332.36(259.39,419.18) | 1.09 | 440.81(380.57,512.55) | 466.14(364.76,585.31) | -0.15(-0.31,0.01) |
| Grenada | both | 1410.39(1241.53,1594.64) | 2617.09(2229.24,3075.03) | 0.86 | 2036.87(1787.99,2306.29) | 2313.36(1978.13,2701.29) | 0.46(0.36,0.56) |
| Guam | both | 993.51(835.37,1174.13) | 2273.84(1813.62,2836.60) | 1.29 | 1213.52(1032.59,1424.18) | 1195.48(951.92,1488.79) | -0.17(-0.42,0.08) |
| Guatemala | both | 30000.88(24468.74,36556.13) | 253586.16(204734.48,306355.89) | 7.45 | 759.76(618.50,921.42) | 2172.32(1750.22,2620.24) | 3.35(2.86,3.84) |
| Guinea | both | 25189.52(20918.37,29987.04) | 62152.10(49946.66,76559.46) | 1.47 | 754.23(623.79,895.04) | 1096.31(883.45,1351.87) | 1.37(1.21,1.53) |
| Guinea-Bissau | both | 4872.21(3808.61,6208.98) | 10875.75(8625.79,13594.86) | 1.23 | 1140.95(899.96,1430.70) | 1405.90(1124.88,1749.12) | 0.71(0.52,0.90) |
| Guyana | both | 10339.35(8956.74,11909.04) | 20601.38(16536.83,24961.26) | 0.99 | 2519.42(2199.67,2877.95) | 3099.36(2501.35,3745.98) | 0.43(0.03,0.84) |
| Haiti | both | 83469.74(66525.81,102521.27) | 164463.51(123331.32,213890.11) | 0.97 | 2367.99(1897.53,2870.57) | 2178.40(1654.28,2860.62) | -0.18(-0.27,-0.08) |
| Honduras | both | 18260.76(14073.31,22893.53) | 76662.55(58566.02,97766.57) | 3.20 | 801.85(615.65,1006.52) | 1174.14(898.84,1492.31) | 1.34(1.27,1.40) |
| Hungary | both | 84040.44(67165.83,104246.72) | 133852.80(102924.69,171001.10) | 0.59 | 579.72(461.16,718.42) | 744.81(568.63,961.99) | 1.00(0.75,1.25) |
| Iceland | both | 672.10(521.20,869.59) | 1947.73(1416.82,2623.13) | 1.90 | 240.03(185.40,311.19) | 377.78(273.05,509.76) | 1.48(1.43,1.53) |
| India | both | 3386362.80(2802934.01,4043664.14) | 12012998.02(9793463.52,14477028.56) | 2.55 | 721.20(602.55,854.69) | 1027.95(843.26,1231.05) | 1.02(0.93,1.11) |
| Indonesia | both | 1085181.36(941531.09,1234982.99) | 3556368.87(3005868.87,4057138.05) | 2.28 | 982.51(856.86,1110.38) | 1494.03(1272.63,1692.41) | 1.64(1.52,1.75) |
| Iran (Islamic Republic of) | both | 138646.05(111692.20,170124.67) | 716456.93(578136.04,882592.04) | 4.17 | 516.51(420.71,628.24) | 958.11(776.60,1170.16) | 2.49(2.36,2.63) |
| Iraq | both | 128267.10(107542.23,151908.85) | 400205.75(319444.74,489469.19) | 2.12 | 1544.01(1292.48,1824.71) | 1624.99(1314.76,1979.27) | 0.03(-0.03,0.10) |
| Ireland | both | 11237.71(9677.90,13067.46) | 28039.44(20613.35,36991.38) | 1.50 | 275.49(236.13,322.85) | 388.72(284.43,515.54) | 1.05(0.90,1.20) |
| Israel | both | 24712.76(20982.88,29071.94) | 76579.95(62796.49,92953.90) | 2.10 | 514.04(436.49,603.96) | 674.77(549.59,825.62) | 0.12(-0.54,0.79) |
| Italy | both | 514775.21(443900.59,598154.37) | 774156.40(616354.22,953118.07) | 0.50 | 583.36(499.42,680.72) | 586.64(455.50,742.84) | 0.16(-0.05,0.38) |
| Jamaica | both | 30281.94(27308.88,33655.93) | 67418.17(55514.97,80860.77) | 1.23 | 1720.96(1554.18,1914.07) | 2260.99(1858.35,2714.27) | 0.66(0.39,0.94) |
| Japan | both | 552718.89(440112.13,688889.01) | 868352.92(635820.93,1156921.56) | 0.57 | 325.89(259.75,405.19) | 313.75(226.25,421.05) | -0.40(-0.62,-0.17) |
| Jordan | both | 21550.75(18283.14,25267.78) | 86617.30(70835.17,107106.86) | 3.02 | 1624.67(1380.10,1899.10) | 1292.88(1069.38,1569.32) | -1.13(-1.47,-0.80) |
| Kazakhstan | both | 62003.91(47868.08,79229.55) | 153586.50(118906.17,194946.55) | 1.48 | 470.29(363.19,595.97) | 859.63(670.02,1081.65) | 1.73(1.43,2.04) |
| Kenya | both | 61068.09(52939.44,70673.80) | 211954.87(177063.57,252994.24) | 2.47 | 729.05(638.36,835.47) | 912.44(771.69,1070.69) | 0.83(0.65,1.01) |
| Kiribati | both | 1874.55(1545.73,2230.75) | 4929.72(3853.82,6147.53) | 1.63 | 4432.88(3665.62,5242.00) | 6161.41(4876.73,7611.34) | 0.82(0.43,1.20) |
| Kuwait | both | 7481.43(5929.47,9358.78) | 35526.37(25656.82,47276.16) | 3.75 | 1072.42(877.31,1310.96) | 1136.27(841.60,1480.01) | -0.09(-0.52,0.35) |
| Kyrgyzstan | both | 10764.05(8664.62,13200.93) | 22591.35(17350.83,28804.43) | 1.10 | 342.35(275.84,420.17) | 446.63(345.04,565.84) | 0.31(0.05,0.56) |
| Lao People's Democratic Republic | both | 27730.55(21417.43,35963.27) | 63214.15(49752.79,78640.06) | 1.28 | 1234.33(966.94,1574.04) | 1363.53(1089.62,1690.22) | 0.19(0.14,0.25) |
| Latvia | both | 10938.12(8457.75,13950.69) | 17205.86(13286.77,21928.36) | 0.57 | 314.66(242.26,400.36) | 491.74(375.76,637.05) | 1.57(1.32,1.82) |
| Lebanon | both | 18443.01(14744.92,22808.30) | 51733.81(38475.56,67616.33) | 1.81 | 801.76(649.51,990.43) | 991.24(737.79,1295.03) | 0.89(0.75,1.03) |
| Lesotho | both | 13055.26(10878.00,15527.95) | 36425.62(27251.14,47003.09) | 1.79 | 1316.25(1101.30,1553.55) | 2817.55(2120.95,3606.79) | 3.42(3.06,3.79) |
| Liberia | both | 9783.60(8069.43,11611.36) | 24336.02(18738.95,30650.88) | 1.49 | 878.79(732.48,1038.87) | 1128.67(883.10,1412.59) | 0.98(0.84,1.13) |
| Libya | both | 13403.13(10316.77,16938.49) | 62338.36(46425.22,81547.32) | 3.65 | 686.76(531.84,866.67) | 1138.79(859.69,1475.17) | 1.96(1.85,2.07) |
| Lithuania | both | 12127.12(9230.05,15617.96) | 16971.57(12595.94,22121.07) | 0.40 | 273.58(208.24,352.13) | 339.73(252.03,447.52) | 0.75(0.58,0.92) |
| Luxembourg | both | 1611.33(1362.54,1909.34) | 5344.80(3860.63,7112.83) | 2.32 | 299.48(251.92,356.77) | 556.16(399.42,741.56) | 2.30(2.11,2.49) |
| North Macedonia | both | 14294.68(11582.56,17310.19) | 40464.18(31978.78,50490.33) | 1.83 | 758.94(620.99,918.03) | 1255.92(994.84,1561.52) | 1.89(1.60,2.19) |
| Madagascar | both | 45989.40(38863.14,54563.33) | 106531.40(84964.20,130977.83) | 1.32 | 853.59(719.60,1006.33) | 906.75(729.46,1105.72) | 0.16(0.08,0.25) |
| Malawi | both | 43785.71(37027.87,50483.13) | 86325.52(71221.25,104612.34) | 0.97 | 1100.94(941.13,1262.24) | 1131.50(941.51,1358.12) | -0.03(-0.26,0.19) |
| Malaysia | both | 92598.66(78764.53,108114.39) | 227262.32(173031.99,292445.13) | 1.45 | 956.70(818.62,1112.19) | 819.82(625.60,1047.98) | -0.83(-1.04,-0.62) |
| Maldives | both | 925.87(753.83,1180.58) | 2558.94(2010.30,3231.55) | 1.76 | 985.03(815.04,1219.54) | 785.32(625.79,989.09) | -1.12(-1.47,-0.77) |
| Mali | both | 33870.10(28012.02,40546.58) | 90847.56(72555.89,110457.67) | 1.68 | 803.16(666.70,952.54) | 1024.91(833.76,1229.47) | 0.78(0.70,0.87) |
| Malta | both | 2951.94(2491.72,3513.17) | 5412.10(4195.39,6921.88) | 0.83 | 693.36(584.83,824.37) | 629.41(483.94,805.99) | -0.27(-0.38,-0.16) |
| Marshall Islands | both | 507.69(421.28,613.65) | 1685.29(1315.05,2146.21) | 2.32 | 2804.10(2342.64,3381.60) | 4191.30(3300.67,5261.92) | 1.32(1.10,1.53) |
| Mauritania | both | 8752.87(7296.61,10340.58) | 17934.58(13929.18,22120.33) | 1.05 | 865.60(722.15,1016.36) | 861.48(671.50,1057.80) | -0.09(-0.17,-0.02) |
| Mauritius | both | 12138.85(10764.51,13854.87) | 57568.58(47491.11,68081.56) | 3.74 | 1563.47(1389.86,1781.83) | 3239.57(2678.42,3824.69) | 4.04(3.29,4.80) |
| Mexico | both | 978244.31(867744.91,1102003.68) | 2716392.98(2316223.86,3202412.33) | 1.78 | 2167.67(1927.48,2438.99) | 2261.13(1935.88,2664.10) | 0.01(-0.15,0.18) |
| Republic of Moldova | both | 20129.80(15881.92,25349.18) | 26839.99(19518.52,35423.09) | 0.33 | 443.24(349.90,557.87) | 478.93(346.40,633.74) | 0.02(-0.18,0.21) |
| Mongolia | both | 2589.04(2008.05,3222.82) | 8680.60(6547.49,11322.08) | 2.35 | 227.60(175.98,282.20) | 316.00(240.33,409.41) | 1.19(1.04,1.34) |
| Montenegro | both | 3789.00(2928.17,4737.89) | 8190.96(6209.76,10482.83) | 1.16 | 606.22(471.25,755.21) | 848.80(642.28,1090.15) | 1.14(1.03,1.25) |
| Morocco | both | 77214.69(61490.62,97914.64) | 325868.96(252352.05,408279.52) | 3.22 | 546.75(436.72,695.74) | 1008.63(786.89,1261.78) | 2.34(2.24,2.43) |
| Mozambique | both | 60888.44(51245.29,71836.66) | 155908.64(125707.50,193183.65) | 1.56 | 986.47(836.14,1157.77) | 1322.15(1081.52,1611.12) | 1.36(1.21,1.51) |
| Myanmar | both | 398330.15(305981.45,506958.75) | 756043.78(627538.30,914435.37) | 0.90 | 1599.72(1249.34,1993.54) | 1587.12(1329.67,1890.32) | -0.14(-0.18,-0.10) |
| Namibia | both | 10967.03(9187.23,12795.88) | 22813.97(17878.50,28160.00) | 1.08 | 1497.49(1255.93,1736.88) | 1634.86(1293.78,2008.76) | 0.00(-0.37,0.38) |
| Nepal | both | 46430.05(35820.58,59091.38) | 175155.26(134743.70,224249.76) | 2.77 | 465.54(364.91,579.16) | 776.39(608.71,980.86) | 1.75(1.60,1.90) |
| Netherlands | both | 97553.32(82690.66,114160.04) | 127870.08(98494.56,162893.61) | 0.31 | 494.54(418.96,579.31) | 401.05(304.68,516.58) | -1.03(-1.20,-0.86) |
| New Zealand | both | 13715.78(11882.11,15875.77) | 28741.54(23108.14,35182.85) | 1.10 | 358.54(310.12,414.73) | 392.17(313.31,483.12) | -0.51(-0.83,-0.20) |
| Nicaragua | both | 18338.41(15423.23,21868.28) | 77017.27(62456.82,94492.85) | 3.20 | 1122.15(948.31,1336.49) | 1664.31(1366.60,2032.36) | 1.14(0.96,1.32) |
| Niger | both | 17926.00(14258.57,21665.63) | 67483.51(53527.34,83908.46) | 2.76 | 623.90(499.73,749.62) | 845.69(674.56,1040.25) | 0.99(0.89,1.10) |
| Nigeria | both | 340098.41(280338.90,406893.97) | 736863.13(585173.92,899651.68) | 1.17 | 774.96(646.31,920.84) | 851.23(691.20,1029.08) | 0.26(0.17,0.34) |
| Democratic People's Republic of Korea | both | 88336.49(68878.90,109669.41) | 198409.51(155155.72,252244.53) | 1.25 | 519.42(411.67,641.84) | 610.96(478.88,772.87) | 0.55(0.49,0.60) |
| Northern Mariana Islands | both | 347.50(283.79,418.70) | 1171.00(972.16,1420.88) | 2.37 | 1583.16(1323.41,1873.38) | 2076.26(1736.74,2488.53) | 1.12(0.75,1.48) |
| Norway | both | 23289.50(18382.34,29088.74) | 38572.36(29094.05,49843.78) | 0.66 | 370.43(289.98,466.88) | 438.45(327.29,572.08) | 0.01(-0.16,0.17) |
| Oman | both | 8538.57(6731.66,10395.90) | 29392.21(23840.04,36086.57) | 2.44 | 1248.34(991.95,1505.85) | 1618.46(1363.58,1912.06) | 1.05(0.86,1.24) |
| Pakistan | both | 475470.35(369268.70,574435.79) | 1829817.08(1480682.99,2215525.93) | 2.85 | 803.56(621.77,961.15) | 1554.53(1270.08,1863.08) | 2.43(2.22,2.64) |
| Palestine | both | 12032.79(9684.63,14775.24) | 44332.44(37713.37,52722.75) | 2.68 | 1398.92(1131.05,1700.57) | 1907.93(1642.58,2233.13) | 1.06(0.78,1.35) |
| Panama | both | 12245.84(9909.55,15036.35) | 52452.84(41187.78,65283.29) | 3.28 | 803.06(654.14,984.51) | 1262.87(991.51,1573.39) | 1.44(1.30,1.57) |
| Papua New Guinea | both | 49419.81(39470.32,60632.31) | 182916.33(145541.62,224594.83) | 2.70 | 2331.38(1874.94,2849.20) | 3260.52(2613.04,3989.29) | 1.11(0.95,1.26) |
| Paraguay | both | 16915.71(14261.14,19912.96) | 77760.37(62462.68,94519.77) | 3.60 | 749.18(632.17,876.13) | 1385.35(1113.08,1681.78) | 2.13(1.79,2.46) |
| Peru | both | 58878.56(48811.70,70162.53) | 197365.87(155840.68,245411.73) | 2.35 | 480.41(397.08,571.18) | 612.76(484.28,761.29) | 0.88(0.69,1.07) |
| Philippines | both | 330699.55(291676.45,372697.62) | 977807.53(833784.70,1148973.84) | 1.96 | 1012.88(890.63,1144.98) | 1195.51(1024.22,1403.12) | 0.48(0.38,0.58) |
| Poland | both | 277933.37(227942.34,336395.33) | 463606.70(353174.51,588122.30) | 0.67 | 634.83(520.16,768.21) | 708.22(535.50,902.47) | 0.62(0.26,0.98) |
| Portugal | both | 91610.65(78379.46,108054.40) | 153283.93(120019.84,192196.31) | 0.67 | 654.82(558.36,774.15) | 684.53(524.62,868.43) | -0.15(-0.35,0.05) |
| Puerto Rico | both | 53536.72(45529.37,62898.42) | 109968.93(88272.31,135650.38) | 1.05 | 1481.51(1259.98,1735.80) | 1706.33(1363.31,2117.16) | 0.19(-0.08,0.46) |
| Qatar | both | 2580.31(2096.23,3128.18) | 28694.37(21143.16,37664.51) | 10.12 | 2528.14(2147.48,2948.90) | 2975.32(2401.18,3673.13) | 0.80(0.31,1.29) |
| Romania | both | 109066.38(86391.52,136071.11) | 165624.99(125856.95,214103.96) | 0.52 | 387.94(306.49,483.92) | 482.75(364.61,624.98) | 0.72(0.59,0.84) |
| Russian Federation | both | 485698.72(387255.95,599335.93) | 893782.15(711033.29,1105597.68) | 0.84 | 270.36(215.11,333.19) | 387.91(307.05,480.30) | 1.10(0.88,1.32) |
| Rwanda | both | 40489.65(33011.33,49824.81) | 60961.99(47792.67,76894.74) | 0.51 | 1330.29(1086.48,1632.30) | 991.38(795.33,1253.85) | -1.76(-2.07,-1.45) |
| Saint Lucia | both | 2242.92(1955.26,2550.70) | 4896.19(3997.74,5927.92) | 1.18 | 2538.15(2214.91,2878.57) | 2269.51(1861.75,2742.07) | -0.90(-1.10,-0.70) |
| Saint Vincent and the Grenadines | both | 1734.69(1538.22,1957.87) | 3530.75(3007.02,4139.52) | 1.04 | 2407.72(2135.27,2720.18) | 2610.44(2224.38,3059.60) | -0.15(-0.34,0.04) |
| Samoa | both | 1946.75(1572.01,2403.56) | 4419.00(3606.13,5440.82) | 1.27 | 2138.74(1738.43,2629.40) | 2887.06(2369.92,3536.54) | 0.94(0.71,1.17) |
| Sao Tome and Principe | both | 311.19(247.71,387.20) | 747.73(586.37,944.40) | 1.40 | 483.37(386.44,600.69) | 687.80(541.76,859.15) | 1.22(1.09,1.34) |
| Saudi Arabia | both | 58772.14(46154.36,72872.52) | 245548.39(187706.25,316976.44) | 3.18 | 902.32(710.14,1117.00) | 1064.47(829.68,1348.61) | 0.19(0.02,0.35) |
| Senegal | both | 29799.20(24359.18,35697.77) | 94064.43(73521.74,115895.57) | 2.16 | 906.91(748.04,1081.03) | 1229.28(975.74,1504.98) | 1.23(1.03,1.43) |
| Serbia | both | 86012.67(69935.90,104070.55) | 149270.13(118065.93,185389.17) | 0.74 | 755.95(617.36,911.01) | 962.84(749.56,1206.37) | 0.82(0.70,0.94) |
| Seychelles | both | 372.37(296.32,459.58) | 1507.90(1159.42,1916.04) | 3.05 | 657.33(525.08,813.66) | 1345.60(1036.15,1705.60) | 2.44(2.27,2.62) |
| Sierra Leone | both | 11341.83(9193.41,13614.57) | 32394.46(25369.81,40626.98) | 1.86 | 592.31(481.09,708.17) | 874.60(694.12,1090.40) | 1.56(1.34,1.77) |
| Singapore | both | 18713.22(15231.61,22911.71) | 38066.00(26372.62,52495.83) | 1.03 | 796.19(649.23,967.34) | 473.44(327.77,650.53) | -1.85(-2.12,-1.57) |
| Slovakia | both | 32847.26(26623.04,39939.22) | 50209.27(37669.81,65507.44) | 0.53 | 549.28(444.33,667.37) | 561.02(419.48,734.71) | 0.05(-0.02,0.12) |
| Slovenia | both | 12102.65(9365.43,15401.89) | 20889.36(15061.23,27254.34) | 0.73 | 499.35(385.64,633.94) | 524.59(370.80,690.09) | -0.84(-1.24,-0.44) |
| Solomon Islands | both | 4159.26(3155.79,5680.75) | 16919.79(13814.61,20779.61) | 3.07 | 2513.15(1927.60,3388.83) | 4391.70(3647.60,5319.26) | 1.94(1.76,2.12) |
| Somalia | both | 32971.78(25454.15,42617.95) | 89777.80(70163.35,113912.93) | 1.72 | 1198.95(946.92,1518.68) | 1251.87(996.59,1563.13) | 0.26(0.21,0.30) |
| South Africa | both | 264253.47(232289.07,296709.69) | 826465.95(732937.02,928952.79) | 2.13 | 1227.95(1074.71,1378.80) | 1843.95(1642.67,2065.49) | 1.71(1.28,2.15) |
| Republic of Korea | both | 207424.66(176501.08,243950.87) | 525344.44(410939.74,652314.33) | 1.53 | 642.72(548.81,768.98) | 596.57(466.52,740.62) | -0.97(-1.49,-0.44) |
| South Sudan | both | 22713.32(17959.57,28441.33) | 38240.37(29498.88,47994.90) | 0.68 | 935.24(747.88,1160.89) | 963.60(748.31,1185.68) | 0.10(0.08,0.13) |
| Spain | both | 331059.56(268528.58,406520.24) | 492078.56(362602.16,651361.54) | 0.49 | 606.01(490.72,743.50) | 553.13(398.04,741.35) | -0.68(-0.87,-0.50) |
| Sri Lanka | both | 95731.50(80176.43,113755.59) | 475543.62(376233.36,588438.32) | 3.97 | 884.94(744.84,1045.57) | 1881.24(1499.55,2323.20) | 3.21(2.81,3.61) |
| Sudan | both | 49524.16(37354.74,64065.08) | 172228.12(126964.48,221856.23) | 2.48 | 510.28(386.47,656.18) | 855.62(643.55,1090.18) | 2.00(1.91,2.10) |
| Suriname | both | 3710.55(3137.82,4383.13) | 12187.94(9907.91,14832.78) | 2.28 | 1369.33(1159.62,1616.71) | 1977.11(1611.75,2404.32) | 1.34(1.17,1.51) |
| Eswatini | both | 6150.97(4956.71,7379.04) | 19714.76(14760.42,25596.25) | 2.21 | 2088.06(1683.78,2485.38) | 3347.02(2545.61,4277.36) | 2.04(1.39,2.68) |
| Sweden | both | 46277.42(38159.24,55790.33) | 77183.80(60938.86,96268.49) | 0.67 | 328.54(266.85,397.89) | 408.01(313.64,518.15) | 0.82(0.71,0.94) |
| Switzerland | both | 44499.95(36953.94,54125.85) | 65706.06(49296.11,86005.43) | 0.48 | 431.55(355.38,528.88) | 405.89(300.49,546.32) | -0.52(-0.63,-0.41) |
| Syrian Arab Republic | both | 41770.98(33396.89,51291.03) | 108820.44(81226.11,142142.67) | 1.61 | 740.13(594.84,903.01) | 853.15(646.49,1103.18) | 0.07(-0.12,0.26) |
| Taiwan (Province of China) | both | 157114.23(136841.52,181288.17) | 362748.96(289655.93,450997.92) | 1.31 | 966.54(848.43,1107.60) | 925.69(737.44,1155.05) | -1.00(-1.44,-0.57) |
| Tajikistan | both | 15739.78(13317.44,18590.74) | 70906.42(57027.50,87317.56) | 3.50 | 526.56(446.39,617.62) | 1223.26(994.80,1494.62) | 3.16(2.90,3.43) |
| United Republic of Tanzania | both | 95502.08(81886.97,110116.25) | 253613.53(207346.83,308277.55) | 1.66 | 865.24(746.25,987.38) | 1000.71(833.20,1203.15) | 0.66(0.60,0.72) |
| Thailand | both | 309661.94(261794.55,364249.36) | 833465.35(651858.75,1033448.43) | 1.69 | 825.63(705.03,966.80) | 818.51(640.02,1017.86) | -0.81(-1.14,-0.49) |
| Bahamas | both | 2502.43(2168.37,2891.36) | 6273.14(5021.76,7787.76) | 1.51 | 1551.39(1350.87,1780.77) | 1551.13(1250.25,1918.21) | -0.32(-0.46,-0.18) |
| Gambia | both | 2424.23(1906.30,2978.15) | 10348.38(8144.25,12706.20) | 3.27 | 676.05(536.77,824.00) | 1057.65(832.72,1297.56) | 1.44(1.25,1.63) |
| Timor-Leste | both | 2231.68(1761.70,2799.02) | 7856.25(6013.25,9911.58) | 2.52 | 699.37(562.95,861.44) | 947.13(733.78,1188.38) | 0.93(0.71,1.15) |
| Togo | both | 8710.93(7225.12,10410.87) | 33883.10(26579.12,42550.23) | 2.89 | 680.34(567.86,808.06) | 902.56(719.75,1131.30) | 0.90(0.70,1.11) |
| Tonga | both | 1407.96(1194.82,1639.17) | 2492.59(2041.27,3016.91) | 0.77 | 2433.64(2073.38,2830.94) | 3112.61(2552.56,3768.92) | 0.96(0.80,1.12) |
| Trinidad and Tobago | both | 30801.79(27911.98,34012.70) | 58064.11(46007.71,71993.89) | 0.89 | 3619.02(3286.89,3987.20) | 3100.06(2462.60,3828.05) | -1.03(-1.22,-0.83) |
| Tunisia | both | 29009.07(22159.65,37305.65) | 120897.15(90700.91,157871.31) | 3.17 | 563.98(434.18,720.31) | 943.84(713.81,1233.84) | 1.93(1.82,2.04) |
| Turkey | both | 413748.38(351292.00,484045.87) | 770690.29(619599.37,956089.41) | 0.86 | 1141.59(976.14,1333.62) | 869.94(702.29,1073.51) | -0.25(-0.64,0.15) |
| Turkmenistan | both | 9873.20(8241.62,11816.52) | 36953.76(29245.50,46847.13) | 2.74 | 472.63(395.12,565.79) | 842.21(669.42,1059.29) | 1.66(1.42,1.90) |
| Uganda | both | 68357.94(54787.55,86913.38) | 176392.90(141577.14,220214.32) | 1.58 | 1034.67(831.13,1311.09) | 1187.02(968.26,1456.13) | 0.20(-0.01,0.40) |
| Ukraine | both | 205622.88(160560.82,259795.65) | 247515.30(187734.62,319579.76) | 0.20 | 296.92(231.69,374.35) | 351.05(266.75,451.67) | 0.16(-0.03,0.35) |
| United Arab Emirates | both | 7697.23(6225.22,9331.91) | 87940.06(66705.16,113102.97) | 10.42 | 1884.22(1558.66,2246.72) | 1865.19(1467.97,2332.36) | 0.01(-0.47,0.49) |
| United Kingdom | both | 317628.72(256414.33,392496.00) | 629185.96(457034.90,830861.51) | 0.98 | 370.21(295.31,461.16) | 589.17(419.71,787.81) | 1.90(1.77,2.02) |
| United States of America | both | 1887310.36(1578350.54,2254701.85) | 4127419.33(3314502.77,5112093.19) | 1.19 | 618.75(515.56,740.85) | 789.98(628.64,981.47) | 1.14(0.98,1.30) |
| Uruguay | both | 15738.60(14450.36,17242.74) | 27391.14(22887.27,32699.23) | 0.74 | 401.32(368.18,439.21) | 529.05(440.23,637.28) | 0.96(0.63,1.29) |
| Uzbekistan | both | 49352.28(41290.06,58442.26) | 315461.27(262690.72,378519.43) | 5.39 | 407.06(341.84,480.18) | 1301.71(1093.47,1551.54) | 4.02(3.55,4.49) |
| Vanuatu | both | 1056.25(807.83,1425.03) | 4926.61(3816.65,6294.02) | 3.66 | 1465.96(1131.08,1965.65) | 2641.72(2061.84,3351.17) | 2.10(2.01,2.19) |
| Venezuela (Bolivarian Republic of) | both | 119566.06(102902.29,138942.55) | 431171.26(339934.05,530900.53) | 2.61 | 1180.99(1021.76,1372.08) | 1456.73(1152.11,1792.32) | 0.61(0.46,0.77) |
| Viet nam | both | 339046.49(274584.83,411607.33) | 967700.03(774604.20,1176849.91) | 1.85 | 844.02(685.07,1017.97) | 1058.51(849.13,1279.71) | 0.95(0.76,1.14) |
| Virginia | both | 40137.96(33024.07,48778.68) | 107418.80(84346.52,133651.10) | 1.68 | 576.41(473.96,701.78) | 793.70(621.07,989.07) | 1.45(1.26,1.63) |
| Yemen | both | 24098.71(18085.54,32144.74) | 96675.98(72067.66,127175.32) | 3.01 | 467.30(354.49,626.37) | 676.50(512.32,878.75) | 1.40(1.32,1.49) |
| Zambia | both | 34110.27(29008.69,39591.46) | 87474.98(71863.27,107033.50) | 1.56 | 1138.89(970.47,1319.41) | 1217.59(1011.96,1467.60) | -0.07(-0.33,0.18) |
| Zimbabwe | both | 43347.47(37289.93,49975.21) | 125040.51(100340.48,152084.19) | 1.88 | 1036.35(894.01,1191.01) | 1720.61(1382.09,2083.39) | 2.22(1.93,2.51) |
| Monaco | both | 130.92(97.94,170.19) | 286.98(203.89,387.45) | 1.19 | 205.13(151.54,269.22) | 350.06(247.12,472.45) | 1.90(1.88,1.92) |
| San Marino | both | 93.78(73.06,119.75) | 243.90(180.67,321.26) | 1.60 | 288.47(224.02,367.46) | 415.16(302.95,550.86) | 1.33(1.27,1.40) |
| Saint Kitts and Nevis | both | 744.53(654.21,846.23) | 1289.70(1049.29,1539.94) | 0.73 | 2067.95(1826.45,2358.93) | 1886.09(1557.50,2241.09) | -0.45(-0.58,-0.32) |
| Cook Islands | both | 420.88(360.89,492.26) | 809.82(686.62,953.95) | 0.92 | 3221.87(2782.06,3737.09) | 3341.81(2821.65,3960.03) | -0.01(-0.13,0.12) |
| Nauru | both | 139.89(109.90,191.19) | 243.90(188.43,355.05) | 0.74 | 3130.78(2510.26,4072.68) | 4673.67(3709.51,6470.25) | 1.19(0.91,1.47) |
| Niue | both | 56.79(47.10,69.47) | 84.75(67.77,103.84) | 0.49 | 2665.76(2205.28,3264.06) | 3959.98(3166.77,4853.46) | 1.23(1.00,1.46) |
| Palau | both | 245.92(199.66,298.97) | 867.68(691.14,1070.50) | 2.53 | 2367.73(1937.01,2862.02) | 3760.83(3008.48,4602.55) | 1.58(1.35,1.81) |
| Tokelau | both | 29.69(23.91,36.30) | 40.66(32.31,50.64) | 0.37 | 2250.69(1812.78,2744.19) | 3024.69(2420.85,3766.55) | 0.97(0.79,1.15) |
| Tuvalu | both | 187.15(154.64,230.36) | 374.51(293.04,492.32) | 1.00 | 2597.90(2159.12,3169.19) | 3551.11(2794.10,4647.18) | 1.03(0.85,1.21) |
| Afghanistan | male | 27258.13(20417.37,36764.18) | 71491.82(53409.24,94871.22) | 1.62 | 722.51(547.46,971.76) | 1053.36(805.06,1376.27) | 1.48(1.42,1.54) |
| Albania | male | 2915.18(2190.18,3823.62) | 7435.20(5228.42,10033.91) | 1.55 | 277.35(210.87,363.38) | 376.98(266.76,511.18) | 0.73(0.58,0.88) |
| Algeria | male | 35662.71(26575.93,46117.25) | 152655.29(112592.42,199620.65) | 3.28 | 586.68(438.47,753.82) | 866.04(648.73,1123.72) | 1.54(1.35,1.73) |
| American Samoa | male | 386.53(322.52,462.95) | 952.28(780.63,1139.60) | 1.46 | 2990.85(2523.32,3539.24) | 3891.99(3201.69,4645.91) | 0.85(0.50,1.20) |
| Andorra | male | 98.75(74.60,127.99) | 309.56(229.11,409.47) | 2.13 | 347.65(265.29,444.97) | 446.90(332.30,588.73) | 0.85(0.82,0.88) |
| Angola | male | 34655.69(26659.35,43304.66) | 95287.45(76038.43,117703.75) | 1.75 | 1639.34(1274.40,2016.31) | 1732.10(1428.13,2105.19) | 0.21(0.10,0.32) |
| Antigua and Barbuda | male | 412.08(360.88,470.80) | 913.49(747.25,1105.92) | 1.22 | 1819.13(1592.45,2077.54) | 1879.03(1546.35,2273.69) | -0.07(-0.26,0.12) |
| Argentina | male | 101202.18(88933.65,114867.23) | 204413.60(171167.10,246705.96) | 1.02 | 697.23(614.63,791.27) | 863.37(722.10,1041.96) | 0.22(-0.01,0.46) |
| Armenia | male | 7239.36(6150.05,8635.86) | 18765.80(15347.83,22523.12) | 1.59 | 580.86(497.81,685.13) | 1039.87(853.06,1244.73) | 1.46(1.02,1.90) |
| Australia | male | 36131.07(30481.34,43110.75) | 93857.60(72716.48,119820.50) | 1.60 | 419.25(354.64,499.12) | 500.06(384.90,640.44) | 0.33(0.15,0.50) |
| Austria | male | 18162.71(15378.76,21726.34) | 42931.47(33810.62,54209.54) | 1.36 | 402.12(340.02,479.95) | 566.62(443.72,721.98) | 1.41(1.03,1.79) |
| Azerbaijan | male | 11624.26(9516.82,14177.57) | 45063.05(35183.60,56734.27) | 2.88 | 500.26(416.07,600.92) | 952.04(751.78,1183.58) | 1.84(1.59,2.09) |
| Bahrain | male | 2129.82(1765.29,2565.18) | 20075.34(15846.01,25164.94) | 8.43 | 2175.55(1807.53,2616.00) | 3246.60(2649.60,3978.11) | 1.47(1.10,1.84) |
| Bangladesh | male | 206081.22(171700.88,242027.94) | 529494.71(420977.20,660839.56) | 1.57 | 790.27(666.29,925.15) | 793.45(634.32,986.15) | -0.12(-0.55,0.31) |
| Barbados | male | 1978.64(1734.37,2254.26) | 3884.57(3216.83,4666.85) | 0.96 | 1649.02(1443.07,1882.90) | 1796.00(1485.19,2159.65) | -0.38(-0.62,-0.13) |
| Belarus | male | 14485.80(11144.31,18680.00) | 17688.66(12290.87,24287.75) | 0.22 | 290.49(223.21,373.31) | 294.95(206.44,401.96) | -0.52(-0.74,-0.30) |
| Belgium | male | 24696.25(19440.50,31438.33) | 46872.69(34388.12,62306.74) | 0.90 | 391.72(309.11,494.91) | 496.69(362.52,667.34) | 0.72(0.62,0.82) |
| Belize | male | 462.93(400.79,542.48) | 2550.19(2171.58,2986.14) | 4.51 | 965.57(837.66,1124.30) | 1705.21(1458.18,1996.03) | 1.65(1.12,2.19) |
| Benin | male | 8027.04(6655.35,9608.81) | 25542.30(19486.52,32562.66) | 2.18 | 829.50(691.69,990.02) | 1075.41(823.17,1366.19) | 0.84(0.58,1.09) |
| Bermuda | male | 293.24(250.67,342.82) | 522.12(414.64,643.74) | 0.78 | 1058.50(906.08,1238.06) | 943.19(749.47,1160.58) | -0.38(-0.49,-0.27) |
| Bhutan | male | 768.84(568.11,996.14) | 2845.81(2185.17,3677.70) | 2.70 | 598.66(443.04,768.83) | 967.06(739.43,1237.31) | 1.88(1.78,1.99) |
| Bolivia (Plurinational State of) | male | 14612.66(11663.24,18650.59) | 51641.31(39841.22,65393.88) | 2.53 | 933.99(749.90,1183.99) | 1197.59(929.44,1507.37) | 0.79(0.72,0.85) |
| Bosnia and Herzegovina | male | 10449.59(8123.22,13118.71) | 36114.06(28574.83,44985.61) | 2.46 | 562.11(444.07,699.86) | 1375.62(1089.39,1712.20) | 3.83(3.38,4.28) |
| Botswana | male | 4169.53(3212.44,5360.53) | 15199.59(11213.96,19364.83) | 2.65 | 1587.78(1238.85,2012.20) | 2496.27(1876.99,3141.03) | 1.02(0.38,1.66) |
| Brazil | male | 463149.89(400395.39,533254.26) | 1169946.59(993213.72,1372072.58) | 1.53 | 1038.57(898.95,1194.52) | 1074.34(914.61,1260.22) | 0.26(0.17,0.34) |
| Brunei Darussalam | male | 1705.61(1417.09,2054.49) | 4406.03(3580.36,5357.70) | 1.58 | 3450.96(2887.44,4142.13) | 2874.90(2415.94,3409.49) | -0.15(-0.40,0.11) |
| Bulgaria | male | 37707.06(31000.77,45915.11) | 47256.65(36369.52,59919.36) | 0.25 | 650.24(533.11,790.14) | 802.07(615.85,1015.11) | 0.34(0.14,0.54) |
| Burkina Faso | male | 22393.95(18402.29,26968.65) | 55083.03(44308.69,67115.33) | 1.46 | 1079.24(898.64,1293.16) | 1269.36(1036.56,1527.51) | 0.45(0.34,0.55) |
| Burundi | male | 16125.05(12366.05,21891.36) | 29216.95(22942.20,36689.93) | 0.81 | 1464.01(1120.89,1979.39) | 1170.14(927.09,1467.81) | -1.05(-1.15,-0.94) |
| Cambodia | male | 19913.71(15053.76,25077.49) | 64692.52(50793.29,81684.69) | 2.25 | 935.71(730.31,1155.53) | 1222.65(966.18,1528.43) | 0.92(0.78,1.06) |
| Cameroon | male | 22025.12(16162.59,28798.15) | 84715.32(64519.02,108236.83) | 2.85 | 1000.07(749.58,1298.29) | 1405.47(1088.64,1772.48) | 1.06(0.68,1.44) |
| Canada | male | 52922.17(45892.03,61861.49) | 146787.79(116068.26,182750.39) | 1.77 | 374.11(325.53,434.60) | 475.70(375.14,593.12) | 0.19(-0.13,0.51) |
| Cabo Verde | male | 409.90(320.65,508.60) | 1958.79(1579.88,2378.60) | 3.78 | 428.31(337.35,531.49) | 1035.36(842.30,1250.89) | 2.47(2.04,2.89) |
| Central African Republic | male | 12440.24(10163.30,15279.80) | 24541.78(18983.42,31507.85) | 0.97 | 2080.87(1716.74,2503.60) | 2141.10(1709.94,2695.50) | 0.14(0.05,0.23) |
| Chad | male | 9805.00(7888.51,12206.52) | 29763.81(23303.72,37775.67) | 2.04 | 714.02(572.76,885.68) | 953.11(749.21,1196.12) | 0.85(0.51,1.20) |
| Chile | male | 27241.31(22984.85,32465.96) | 83129.43(65011.77,104199.67) | 2.05 | 593.26(502.25,702.91) | 762.84(598.66,954.43) | 0.82(0.68,0.97) |
| China | male | 1889054.71(1477927.37,2395592.83) | 5071241.68(3958579.32,6304326.62) | 1.68 | 423.21(334.11,529.57) | 522.61(409.67,646.19) | 0.83(0.63,1.02) |
| Colombia | male | 80798.66(63738.88,100722.93) | 226424.27(167447.32,294248.03) | 1.80 | 886.28(699.68,1104.71) | 935.50(693.59,1215.51) | -0.33(-0.57,-0.08) |
| Comoros | male | 1137.15(724.29,1456.20) | 2361.76(1862.32,2943.75) | 1.08 | 1053.82(702.74,1332.80) | 1046.68(839.85,1286.75) | -0.20(-0.34,-0.05) |
| Congo | male | 11350.58(9383.53,13346.23) | 23684.44(18618.65,29482.77) | 1.09 | 2234.93(1872.85,2608.18) | 1751.79(1393.77,2153.22) | -1.13(-1.33,-0.93) |
| Costa Rica | male | 6914.27(5372.89,8731.33) | 24190.18(17819.02,31848.55) | 2.50 | 783.78(609.79,989.64) | 1003.48(742.63,1323.36) | 0.63(0.44,0.82) |
| C么te d'Ivoire | male | 22559.82(18498.38,27028.08) | 65981.79(52029.83,82087.06) | 1.92 | 994.72(825.34,1184.92) | 1133.61(921.29,1387.93) | -0.06(-0.45,0.33) |
| Croatia | male | 16148.56(12565.74,20508.92) | 28498.42(21345.93,37008.22) | 0.76 | 608.25(478.44,766.57) | 800.03(596.75,1046.50) | 0.70(0.57,0.83) |
| Cuba | male | 47388.01(37021.42,59218.89) | 84622.83(61451.13,112446.68) | 0.79 | 929.23(725.61,1162.28) | 983.19(714.28,1303.07) | -0.01(-0.34,0.32) |
| Cyprus | male | 4498.34(3696.77,5401.28) | 8107.70(6509.45,10077.14) | 0.80 | 1200.39(994.47,1438.90) | 886.44(709.88,1097.20) | -1.62(-1.90,-1.33) |
| Czechia | male | 42607.14(32633.46,54107.08) | 104628.50(77555.12,136632.11) | 1.46 | 750.74(576.15,952.42) | 1190.53(879.55,1556.63) | 2.05(1.73,2.37) |
| Democratic Republic of the Congo | male | 122291.20(100232.78,147051.56) | 278878.84(221349.87,346570.59) | 1.28 | 1594.61(1324.60,1898.52) | 1583.13(1267.55,1953.45) | -0.12(-0.19,-0.05) |
| Denmark | male | 12062.15(10509.18,13926.80) | 24524.06(20505.60,29438.58) | 1.03 | 359.94(313.33,416.38) | 493.11(408.88,597.64) | 1.11(0.74,1.49) |
| Djibouti | male | 786.20(588.49,1065.15) | 4492.13(3355.58,5963.00) | 4.71 | 1010.24(772.52,1357.87) | 1321.09(1020.96,1709.26) | 0.92(0.82,1.03) |
| Dominica | male | 504.45(432.56,585.66) | 979.95(784.37,1206.09) | 0.94 | 1749.60(1494.39,2041.55) | 2233.58(1795.43,2748.11) | 0.60(0.49,0.72) |
| Dominican Republic | male | 13286.58(11285.10,15426.74) | 57291.44(43023.33,74319.30) | 3.31 | 662.18(560.46,768.81) | 1207.91(911.65,1554.20) | 2.41(2.25,2.57) |
| Ecuador | male | 18242.06(15642.50,21292.83) | 84739.15(68075.55,105723.75) | 3.65 | 665.68(570.47,778.08) | 1152.85(928.74,1430.00) | 1.75(1.49,2.02) |
| Egypt | male | 96522.64(83487.47,110999.41) | 428393.21(330029.62,527528.47) | 3.44 | 618.60(537.49,706.18) | 1146.52(882.87,1410.27) | 2.38(2.26,2.51) |
| El Salvador | male | 11005.10(8986.44,13270.33) | 39696.31(30888.59,49030.71) | 2.61 | 762.39(622.86,918.58) | 1591.73(1238.52,1969.39) | 2.61(2.28,2.93) |
| Equatorial Guinea | male | 1807.65(1357.36,2275.37) | 4385.77(3179.20,5745.79) | 1.43 | 1919.80(1460.28,2379.53) | 1995.98(1503.16,2581.29) | 0.16(-0.13,0.45) |
| Eritrea | male | 6514.38(4433.43,9294.83) | 18546.21(13544.95,25056.96) | 1.85 | 1325.44(898.64,1868.84) | 1428.29(1059.07,1876.25) | 0.08(-0.11,0.27) |
| Estonia | male | 2389.73(1779.84,3099.91) | 4280.54(3134.66,5677.80) | 0.79 | 309.64(231.08,400.03) | 461.48(337.41,610.35) | 1.42(1.00,1.83) |
| Ethiopia | male | 199226.50(156554.94,264895.32) | 222092.80(183174.34,267469.54) | 0.11 | 1795.57(1425.42,2380.71) | 1035.06(853.76,1240.46) | -2.15(-2.33,-1.96) |
| Micronesia (Federated States of) | male | 680.50(531.46,894.34) | 1913.49(1254.31,2862.77) | 1.81 | 2664.97(2112.15,3449.91) | 4933.05(3323.72,7191.67) | 2.04(1.68,2.41) |
| Fiji | male | 8769.29(7195.45,10997.80) | 26849.47(21709.05,32704.81) | 2.06 | 4499.78(3718.09,5608.25) | 7216.35(5926.86,8650.28) | 1.26(0.89,1.62) |
| Finland | male | 10974.60(8445.31,14106.11) | 24566.66(17636.68,32945.82) | 1.24 | 387.68(299.58,495.14) | 521.88(371.55,695.81) | 1.08(0.97,1.19) |
| France | male | 87666.92(74039.41,104571.56) | 186033.08(152089.16,227429.81) | 1.12 | 260.96(220.61,310.70) | 332.30(269.79,409.73) | 0.71(0.38,1.03) |
| Gabon | male | 6662.59(4995.67,8915.53) | 13380.46(10533.33,16494.18) | 1.01 | 2583.60(1948.07,3455.28) | 2602.81(2103.75,3136.60) | -0.03(-0.08,0.01) |
| Georgia | male | 13423.43(11007.43,16364.54) | 27747.87(22229.41,34164.99) | 1.07 | 517.85(424.52,630.84) | 1160.68(928.59,1430.05) | 2.58(1.69,3.48) |
| Germany | male | 276606.36(224006.54,339823.51) | 525218.97(404396.72,672305.74) | 0.90 | 568.63(462.05,695.64) | 675.89(515.04,868.76) | 0.11(-0.14,0.36) |
| Ghana | male | 23427.04(19182.74,28108.96) | 118887.79(95777.02,145964.54) | 4.07 | 768.92(636.05,910.09) | 1608.19(1310.80,1960.63) | 3.25(2.70,3.80) |
| Greece | male | 24057.99(18456.77,31089.65) | 49439.37(35868.87,65823.78) | 1.06 | 349.74(268.93,451.68) | 527.31(380.62,701.61) | 1.53(1.44,1.61) |
| Greenland | male | 96.25(79.01,117.30) | 230.55(181.27,291.31) | 1.40 | 505.09(422.29,606.45) | 598.10(470.97,756.13) | 0.64(0.52,0.77) |
| Grenada | male | 557.02(482.32,637.46) | 1307.18(1101.25,1542.85) | 1.35 | 1880.07(1628.73,2149.32) | 2361.47(2006.77,2770.85) | 0.78(0.48,1.09) |
| Guam | male | 440.81(364.89,531.87) | 1142.47(891.80,1426.86) | 1.59 | 1005.74(841.82,1208.29) | 1207.59(945.69,1503.73) | 0.70(0.54,0.86) |
| Guatemala | male | 14299.02(11370.61,17657.44) | 109007.94(87709.22,132468.01) | 6.62 | 725.08(579.65,895.00) | 2059.90(1658.53,2497.59) | 3.39(2.83,3.96) |
| Guinea | male | 11192.40(8931.10,13916.62) | 28083.37(22548.52,35207.96) | 1.51 | 676.50(542.26,833.42) | 992.63(802.21,1235.22) | 1.36(1.12,1.60) |
| Guinea-Bissau | male | 2695.80(2087.24,3514.47) | 4869.55(3906.81,6057.25) | 0.81 | 1314.38(1018.12,1695.22) | 1380.70(1122.92,1693.98) | 0.07(-0.12,0.26) |
| Guyana | male | 4517.37(3860.32,5250.03) | 9587.31(7683.53,11781.75) | 1.12 | 2261.75(1943.80,2625.54) | 3022.53(2429.41,3678.33) | 0.78(0.40,1.16) |
| Haiti | male | 26018.72(20907.48,33151.21) | 53253.45(40157.30,69453.95) | 1.05 | 1552.61(1253.62,1967.75) | 1513.93(1157.77,1977.22) | 0.06(-0.15,0.26) |
| Honduras | male | 9570.50(7300.45,12262.27) | 37577.91(28514.67,48620.70) | 2.93 | 862.70(659.31,1106.81) | 1229.02(931.60,1577.42) | 1.34(1.26,1.42) |
| Hungary | male | 36227.89(28377.84,45881.56) | 66422.55(50734.35,85129.67) | 0.83 | 593.92(466.59,754.52) | 885.64(674.90,1141.71) | 1.50(1.16,1.85) |
| Iceland | male | 347.86(267.88,443.86) | 1116.18(827.93,1505.53) | 2.21 | 268.93(207.02,342.46) | 446.02(329.00,600.62) | 1.74(1.69,1.79) |
| India | male | 1873367.16(1525233.93,2282871.53) | 6413602.23(5056820.84,7784706.07) | 2.42 | 767.08(635.48,925.65) | 1115.00(887.66,1344.26) | 1.10(1.01,1.19) |
| Indonesia | male | 515238.07(439483.53,591840.78) | 1785329.26(1439270.11,2148605.25) | 2.47 | 981.12(843.88,1124.08) | 1546.96(1258.64,1843.06) | 1.75(1.65,1.85) |
| Iran (Islamic Republic of) | male | 70747.09(55886.20,87857.42) | 343767.77(276157.62,422869.80) | 3.86 | 498.12(398.87,614.42) | 913.79(740.01,1115.30) | 2.52(2.35,2.69) |
| Iraq | male | 58716.65(47630.55,70999.15) | 199861.67(159893.83,243996.34) | 2.40 | 1454.09(1177.35,1767.01) | 1652.18(1339.43,1995.44) | 0.29(0.23,0.35) |
| Ireland | male | 5729.36(4963.21,6672.47) | 15540.22(11393.41,20577.68) | 1.71 | 311.68(269.49,363.36) | 453.76(332.82,602.61) | 1.07(0.99,1.16) |
| Israel | male | 11246.00(9561.03,13203.28) | 38403.42(31888.61,46430.43) | 2.41 | 512.91(434.43,601.80) | 741.85(613.76,900.28) | 0.43(-0.27,1.14) |
| Italy | male | 219059.30(187000.91,255674.97) | 389376.41(309800.93,481756.56) | 0.78 | 582.41(495.48,683.36) | 670.09(525.72,837.21) | 0.72(0.42,1.02) |
| Jamaica | male | 11480.98(10122.29,13042.33) | 29823.48(24001.91,36157.81) | 1.60 | 1398.00(1232.86,1589.88) | 2084.82(1682.69,2527.36) | 1.26(0.89,1.63) |
| Japan | male | 308989.28(243651.96,386666.80) | 490230.37(359387.60,646367.97) | 0.59 | 405.03(320.49,505.35) | 390.83(283.67,520.37) | -0.30(-0.49,-0.10) |
| Jordan | male | 9400.74(7630.24,11366.98) | 49226.77(38765.35,61874.61) | 4.24 | 1332.43(1099.03,1603.34) | 1349.17(1081.72,1674.91) | -0.07(-0.21,0.08) |
| Kazakhstan | male | 20480.48(15688.81,26289.14) | 57167.70(44535.19,73479.50) | 1.79 | 376.39(290.59,476.85) | 751.55(588.68,949.49) | 1.96(1.64,2.28) |
| Kenya | male | 34353.09(28994.34,41290.72) | 129644.01(105063.42,156880.74) | 2.77 | 843.50(715.76,1003.65) | 1185.13(971.26,1415.95) | 1.20(0.88,1.52) |
| Kiribati | male | 855.29(679.56,1070.09) | 2612.46(1861.65,3440.42) | 2.05 | 4343.17(3477.05,5363.88) | 7223.14(5165.68,9376.05) | 1.37(0.83,1.92) |
| Kuwait | male | 4559.49(3533.19,5817.08) | 21983.05(16004.24,29725.70) | 3.82 | 1025.76(820.82,1284.76) | 1215.68(917.37,1595.55) | 0.18(-0.29,0.65) |
| Kyrgyzstan | male | 4539.73(3708.94,5562.74) | 10082.80(7687.70,12902.87) | 1.22 | 337.68(274.86,412.18) | 437.37(334.33,557.83) | 0.28(0.02,0.54) |
| Lao People's Democratic Republic | male | 12206.80(9374.83,15966.68) | 28822.72(22276.54,37444.21) | 1.36 | 1139.80(883.99,1466.43) | 1266.48(992.01,1615.83) | 0.27(0.22,0.31) |
| Latvia | male | 4196.54(3193.32,5347.66) | 7063.78(5402.72,9117.23) | 0.68 | 311.49(237.53,397.10) | 520.89(397.45,670.75) | 1.80(1.52,2.08) |
| Lebanon | male | 9746.50(7807.35,12268.41) | 27199.13(19803.52,35829.54) | 1.79 | 864.60(699.79,1074.25) | 1149.75(838.66,1510.46) | 1.29(1.07,1.51) |
| Lesotho | male | 6300.89(5106.17,7682.82) | 16357.37(12386.48,21359.50) | 1.60 | 1451.20(1185.98,1760.82) | 2990.06(2267.08,3820.82) | 2.88(2.60,3.16) |
| Liberia | male | 5164.40(4203.87,6263.97) | 11548.53(8568.21,15130.39) | 1.24 | 869.78(709.09,1051.01) | 1044.64(783.62,1349.79) | 0.69(0.53,0.84) |
| Libya | male | 7021.35(5307.35,9041.13) | 31494.73(22520.66,42190.40) | 3.49 | 670.21(509.24,855.89) | 1123.99(810.65,1491.76) | 1.94(1.82,2.06) |
| Lithuania | male | 5271.40(3993.12,6843.03) | 7805.95(5826.42,10346.31) | 0.48 | 293.47(221.85,379.38) | 393.59(295.57,523.43) | 1.01(0.81,1.21) |
| Luxembourg | male | 702.26(586.48,846.97) | 2856.13(2034.18,3843.30) | 3.07 | 314.35(263.96,378.00) | 626.46(447.53,845.52) | 2.64(2.52,2.76) |
| North Macedonia | male | 6517.69(5181.75,8050.41) | 19496.24(15107.96,24202.16) | 1.99 | 711.81(570.45,880.29) | 1262.47(986.69,1558.99) | 2.04(1.69,2.38) |
| Madagascar | male | 24235.11(19632.37,29487.78) | 54473.80(42414.19,68395.91) | 1.25 | 905.30(735.33,1102.58) | 964.61(761.23,1203.24) | 0.23(0.13,0.32) |
| Malawi | male | 22900.50(19252.56,26867.41) | 51174.40(41672.24,62218.11) | 1.23 | 1236.29(1045.45,1434.24) | 1502.99(1244.46,1806.67) | 0.56(0.20,0.93) |
| Malaysia | male | 42570.41(35895.90,50393.96) | 113294.79(86070.07,146122.87) | 1.66 | 884.74(744.97,1045.08) | 807.95(614.85,1035.53) | -0.70(-0.97,-0.43) |
| Maldives | male | 456.76(369.33,559.88) | 1527.20(1198.91,1950.01) | 2.34 | 861.15(706.08,1041.92) | 831.12(659.77,1045.57) | -0.40(-0.70,-0.10) |
| Mali | male | 14511.31(11671.23,17843.44) | 37152.56(29390.55,46596.29) | 1.56 | 703.70(569.95,857.54) | 822.85(655.30,1015.55) | 0.51(0.47,0.56) |
| Malta | male | 1264.61(1042.22,1532.06) | 2632.31(2058.09,3305.09) | 1.08 | 672.88(559.67,811.35) | 658.06(512.12,832.45) | 0.09(-0.05,0.24) |
| Marshall Islands | male | 244.43(197.57,301.36) | 743.41(578.95,946.29) | 2.04 | 2689.22(2181.76,3278.49) | 3579.54(2833.73,4505.12) | 1.01(0.87,1.15) |
| Mauritania | male | 3895.79(3194.04,4614.00) | 7007.83(5191.76,9065.84) | 0.80 | 813.25(671.52,959.15) | 676.85(505.00,863.56) | -0.83(-0.93,-0.73) |
| Mauritius | male | 6124.01(5387.78,7004.26) | 30529.82(24973.63,36411.21) | 3.99 | 1670.48(1479.36,1902.50) | 3663.33(3007.96,4348.70) | 4.45(3.62,5.28) |
| Mexico | male | 448659.31(397474.41,506287.49) | 1399852.41(1163985.44,1669777.34) | 2.12 | 2036.27(1804.30,2295.34) | 2475.32(2066.40,2947.09) | 0.61(0.41,0.81) |
| Republic of Moldova | male | 8195.31(6412.76,10355.64) | 11779.21(8497.85,15673.54) | 0.44 | 428.55(334.90,537.40) | 495.31(356.55,657.03) | 0.22(0.01,0.43) |
| Mongolia | male | 1314.13(1020.54,1629.81) | 4742.08(3584.48,6133.32) | 2.61 | 247.22(194.47,303.50) | 370.03(282.29,468.70) | 1.48(1.28,1.68) |
| Montenegro | male | 1783.23(1370.89,2269.97) | 3979.00(2997.85,5135.91) | 1.23 | 634.97(491.05,805.57) | 904.84(680.97,1173.55) | 1.20(1.10,1.31) |
| Morocco | male | 35113.31(27354.52,44025.46) | 149153.22(111315.76,190483.09) | 3.25 | 505.61(393.25,628.48) | 936.05(701.84,1175.73) | 2.23(2.11,2.35) |
| Mozambique | male | 33768.21(27920.24,40158.12) | 96756.22(78076.07,119146.00) | 1.87 | 1160.06(964.35,1370.72) | 1829.35(1506.02,2193.54) | 2.06(1.88,2.23) |
| Myanmar | male | 182271.49(136493.42,236700.68) | 375857.86(312097.36,459621.35) | 1.06 | 1562.19(1190.55,1987.31) | 1773.50(1488.33,2143.66) | 0.42(0.37,0.47) |
| Namibia | male | 4969.43(3928.98,6083.32) | 10925.54(8810.51,13721.66) | 1.20 | 1487.05(1181.80,1833.20) | 1835.89(1488.67,2294.21) | 0.50(0.12,0.89) |
| Nepal | male | 27270.35(21092.24,34707.79) | 96785.85(73774.61,124409.11) | 2.55 | 516.25(402.74,652.23) | 877.93(674.84,1117.50) | 1.83(1.67,1.99) |
| Netherlands | male | 44043.64(37033.22,52790.10) | 68692.19(53527.17,88249.96) | 0.56 | 526.44(444.59,627.73) | 464.00(357.85,594.07) | -0.74(-0.94,-0.55) |
| New Zealand | male | 6817.25(5885.95,7862.03) | 15366.48(12428.20,18654.15) | 1.25 | 391.71(338.58,451.28) | 446.59(361.16,547.74) | -0.43(-0.75,-0.11) |
| Nicaragua | male | 9027.99(7529.27,10790.01) | 36962.15(29513.30,45679.59) | 3.09 | 1191.87(1002.97,1425.81) | 1753.38(1405.66,2139.21) | 1.18(1.05,1.31) |
| Niger | male | 9392.82(7076.42,12301.21) | 28846.10(22332.87,36689.95) | 2.07 | 628.44(479.22,814.90) | 747.71(585.00,938.28) | 0.60(0.52,0.68) |
| Nigeria | male | 194016.54(148140.71,245763.72) | 380891.60(289530.42,499513.95) | 0.96 | 854.18(670.16,1070.63) | 931.35(720.66,1205.66) | 0.28(0.17,0.39) |
| Democratic People's Republic of Korea | male | 36349.43(27632.72,46487.42) | 88065.66(68597.75,112299.57) | 1.42 | 511.08(396.64,649.00) | 620.12(486.82,777.08) | 0.65(0.61,0.68) |
| Northern Mariana Islands | male | 197.09(157.96,240.88) | 650.13(536.62,781.19) | 2.30 | 1468.59(1215.49,1757.49) | 2236.40(1876.49,2658.21) | 1.66(1.19,2.13) |
| Norway | male | 11710.57(9293.32,14568.25) | 20437.85(15460.54,26281.40) | 0.75 | 426.33(335.96,533.06) | 490.45(367.98,635.44) | -0.02(-0.23,0.19) |
| Oman | male | 4444.94(3476.64,5504.39) | 17886.23(14012.44,22362.04) | 3.02 | 1176.89(929.17,1453.25) | 1713.32(1403.51,2051.30) | 1.54(1.33,1.75) |
| Pakistan | male | 270630.35(200851.09,333807.37) | 889942.20(702910.72,1121785.36) | 2.29 | 841.78(626.36,1034.84) | 1462.24(1157.05,1818.92) | 1.99(1.79,2.19) |
| Palestine | male | 5365.60(4199.98,6742.58) | 23153.77(19381.98,27505.93) | 3.32 | 1375.92(1078.30,1729.58) | 2031.40(1725.90,2395.25) | 1.26(1.00,1.51) |
| Panama | male | 5945.02(4666.62,7441.01) | 26715.80(21057.03,33557.59) | 3.49 | 769.43(607.02,962.56) | 1315.36(1036.24,1651.98) | 1.83(1.68,1.97) |
| Papua New Guinea | male | 33446.28(26107.62,41584.11) | 116693.81(91595.56,143635.48) | 2.49 | 3080.12(2422.62,3841.12) | 3993.86(3144.76,4884.16) | 0.80(0.65,0.96) |
| Paraguay | male | 7128.62(5910.09,8502.82) | 38528.15(30570.17,47111.88) | 4.40 | 655.00(544.00,778.20) | 1411.09(1121.25,1720.98) | 2.61(2.28,2.94) |
| Peru | male | 29302.43(24059.24,35030.55) | 97719.86(76033.31,122997.52) | 2.33 | 485.32(398.27,579.04) | 627.71(488.71,789.47) | 1.09(0.88,1.29) |
| Philippines | male | 187643.22(163498.40,212784.44) | 496580.79(401214.14,618973.14) | 1.65 | 1110.67(968.11,1263.92) | 1253.86(1018.72,1554.21) | 0.21(0.06,0.36) |
| Poland | male | 119067.07(96739.54,144951.31) | 234135.12(177495.41,297841.39) | 0.97 | 634.67(516.25,771.39) | 823.71(622.66,1044.47) | 1.16(0.82,1.50) |
| Portugal | male | 40436.30(34054.89,48274.27) | 71202.99(56123.49,89401.68) | 0.76 | 669.36(562.49,799.84) | 737.87(573.86,929.46) | 0.10(-0.13,0.33) |
| Puerto Rico | male | 25516.29(21655.98,30100.43) | 54843.22(43666.96,67773.31) | 1.15 | 1527.33(1297.54,1801.25) | 1913.93(1517.54,2377.64) | 0.51(0.20,0.82) |
| Qatar | male | 1694.67(1342.45,2118.49) | 21496.10(15697.17,28546.64) | 11.68 | 2545.47(2071.49,3057.78) | 2769.75(2202.23,3468.86) | 0.42(0.02,0.82) |
| Romania | male | 52547.48(41169.46,66825.41) | 82272.96(61944.31,107645.71) | 0.57 | 412.02(322.36,523.06) | 545.37(409.27,712.24) | 0.93(0.81,1.05) |
| Russian Federation | male | 166734.69(128913.66,209291.44) | 330478.63(260442.16,415782.11) | 0.98 | 251.48(196.17,313.31) | 368.82(291.13,464.91) | 1.16(0.99,1.34) |
| Rwanda | male | 21215.63(16938.40,27533.92) | 31471.71(23906.39,40918.89) | 0.48 | 1571.92(1251.88,2040.26) | 1209.82(937.19,1566.13) | -1.81(-2.17,-1.44) |
| Saint Lucia | male | 854.18(739.48,980.79) | 2287.89(1853.60,2765.98) | 1.68 | 2190.74(1906.18,2512.18) | 2216.75(1800.71,2665.89) | -0.23(-0.36,-0.11) |
| Saint Vincent and the Grenadines | male | 621.56(538.71,716.17) | 1765.48(1494.67,2095.81) | 1.84 | 1929.23(1675.17,2223.08) | 2527.30(2146.31,2985.81) | 0.49(0.30,0.68) |
| Samoa | male | 1067.18(855.11,1343.35) | 2138.30(1758.95,2570.61) | 1.00 | 2371.24(1923.21,2958.95) | 2787.95(2323.22,3319.71) | 0.37(0.19,0.56) |
| Sao Tome and Principe | male | 163.09(127.12,205.17) | 405.57(318.32,505.00) | 1.49 | 538.50(424.35,675.58) | 773.88(613.57,951.32) | 1.29(1.19,1.39) |
| Saudi Arabia | male | 34147.85(26193.63,43522.66) | 153011.91(115643.52,199550.92) | 3.48 | 898.95(691.03,1144.79) | 1110.36(853.98,1406.95) | 0.38(0.22,0.53) |
| Senegal | male | 15850.20(12672.83,19215.66) | 43050.24(33410.56,54149.33) | 1.72 | 962.28(776.14,1160.47) | 1169.63(914.75,1451.07) | 0.79(0.61,0.98) |
| Serbia | male | 40530.36(32139.97,49571.68) | 74051.96(57791.94,93445.79) | 0.83 | 746.36(591.21,910.79) | 1054.89(823.41,1332.23) | 1.18(1.04,1.33) |
| Seychelles | male | 173.67(137.82,218.56) | 758.90(575.03,972.85) | 3.37 | 694.76(553.97,872.62) | 1372.27(1050.30,1745.15) | 2.21(2.03,2.39) |
| Sierra Leone | male | 6282.83(5046.05,7751.61) | 13531.41(10380.28,17506.29) | 1.15 | 638.98(515.77,779.10) | 731.68(572.01,942.94) | 0.51(0.34,0.67) |
| Singapore | male | 8866.66(6940.41,11041.38) | 22677.30(15625.33,31803.17) | 1.56 | 792.87(623.73,981.29) | 559.46(386.49,780.95) | -1.38(-1.65,-1.12) |
| Slovakia | male | 15381.08(12260.71,18908.07) | 24406.65(18103.50,31979.86) | 0.59 | 596.34(478.57,733.75) | 621.99(462.09,813.05) | 0.13(0.06,0.20) |
| Slovenia | male | 5092.56(3907.94,6557.80) | 10896.51(7932.70,14230.81) | 1.14 | 518.29(397.68,662.14) | 607.58(438.53,797.49) | -0.49(-0.95,-0.03) |
| Solomon Islands | male | 2163.22(1610.02,3062.47) | 8482.24(6907.24,10450.80) | 2.92 | 2431.77(1839.00,3464.48) | 4356.66(3581.83,5308.55) | 2.06(1.85,2.27) |
| Somalia | male | 18055.86(13651.81,23654.93) | 46301.03(35434.78,59298.48) | 1.56 | 1394.18(1077.82,1798.62) | 1474.98(1132.60,1854.89) | 0.24(0.17,0.31) |
| South Africa | male | 108727.81(94064.78,122696.05) | 358960.88(319969.69,402878.74) | 2.30 | 1171.67(1006.14,1324.87) | 1877.29(1684.94,2093.26) | 1.84(1.39,2.28) |
| Republic of Korea | male | 113304.20(98205.25,131234.52) | 286285.56(226442.03,355293.14) | 1.53 | 776.21(675.84,896.09) | 700.39(554.75,865.40) | -0.99(-1.48,-0.49) |
| South Sudan | male | 13763.25(10803.05,17686.81) | 22383.75(16911.83,28643.74) | 0.63 | 1024.97(804.49,1304.98) | 1084.52(826.77,1367.91) | 0.17(0.13,0.21) |
| Spain | male | 139831.19(112368.69,174513.14) | 244751.06(179595.79,326889.83) | 0.75 | 599.27(481.74,745.38) | 620.80(450.41,832.01) | -0.51(-0.81,-0.21) |
| Sri Lanka | male | 53051.41(44686.02,62974.81) | 227100.86(179397.08,281134.55) | 3.28 | 975.46(821.90,1152.98) | 2014.59(1604.48,2486.50) | 3.30(2.83,3.77) |
| Sudan | male | 24625.85(18484.00,31828.34) | 91644.55(66039.42,121542.50) | 2.72 | 488.62(372.21,627.52) | 857.85(619.19,1124.79) | 2.12(2.02,2.22) |
| Suriname | male | 1773.20(1488.78,2098.87) | 6135.12(4972.08,7521.19) | 2.46 | 1355.43(1144.73,1602.30) | 2109.48(1715.71,2590.72) | 1.58(1.39,1.77) |
| Eswatini | male | 2986.70(2261.83,3783.43) | 10346.29(7367.11,13282.96) | 2.46 | 2296.85(1763.82,2867.63) | 4238.74(3011.44,5284.97) | 2.37(1.72,3.02) |
| Sweden | male | 23387.32(19143.45,28456.82) | 42480.43(33364.92,52664.95) | 0.82 | 377.41(307.45,460.16) | 477.32(368.18,600.69) | 0.86(0.73,0.98) |
| Switzerland | male | 19992.63(16464.81,24427.03) | 34267.36(25637.70,45507.33) | 0.71 | 466.00(383.61,569.59) | 460.18(341.88,614.65) | -0.40(-0.57,-0.23) |
| Syrian Arab Republic | male | 19566.53(15409.52,24364.39) | 53600.72(40041.36,69758.19) | 1.74 | 663.92(525.38,825.18) | 820.15(615.48,1057.33) | 0.38(0.21,0.55) |
| Taiwan (Province of China) | male | 75909.72(65480.53,88503.16) | 191107.62(152091.15,236930.94) | 1.52 | 867.19(754.42,1005.72) | 1039.01(826.38,1291.68) | -0.18(-0.63,0.28) |
| Tajikistan | male | 7537.15(6352.24,8808.59) | 35368.59(27917.08,44041.08) | 3.69 | 544.58(460.16,635.58) | 1251.07(1000.50,1527.09) | 3.01(2.71,3.32) |
| United Republic of Tanzania | male | 51227.87(42923.78,60600.30) | 137632.49(109476.04,170481.39) | 1.69 | 960.20(812.35,1128.35) | 1140.61(911.62,1396.64) | 0.67(0.54,0.81) |
| Thailand | male | 139889.80(113367.28,172317.48) | 396440.82(304467.53,501673.69) | 1.83 | 780.80(636.81,969.43) | 841.73(649.94,1063.19) | -0.50(-0.90,-0.09) |
| Bahamas | male | 1070.91(918.65,1254.63) | 3029.82(2397.85,3784.63) | 1.83 | 1476.89(1271.18,1720.76) | 1619.58(1293.86,2011.77) | 0.01(-0.12,0.13) |
| Gambia | male | 1370.74(1062.07,1760.03) | 4918.03(3844.08,6167.68) | 2.59 | 724.45(571.87,913.45) | 1036.11(812.95,1287.81) | 1.06(0.86,1.26) |
| Timor-Leste | male | 956.76(726.21,1215.46) | 3665.91(2690.94,4682.51) | 2.83 | 579.87(450.11,727.90) | 887.77(658.80,1131.90) | 1.41(1.17,1.64) |
| Togo | male | 4259.40(3492.69,5172.99) | 15825.26(12312.02,20377.75) | 2.72 | 707.49(585.37,856.83) | 956.23(757.77,1213.00) | 0.92(0.60,1.25) |
| Tonga | male | 513.25(421.23,625.79) | 1179.35(917.31,1485.07) | 1.30 | 1852.10(1536.11,2244.01) | 3129.51(2436.60,3916.64) | 1.97(1.59,2.34) |
| Trinidad and Tobago | male | 14684.51(13330.32,16429.84) | 30817.37(24123.02,38485.67) | 1.10 | 3589.03(3263.72,4001.49) | 3391.38(2659.85,4226.44) | -0.69(-0.91,-0.48) |
| Tunisia | male | 15722.51(11739.91,20333.24) | 65145.74(48070.73,85081.75) | 3.14 | 593.76(446.58,761.73) | 1040.95(771.29,1355.65) | 2.19(2.01,2.38) |
| Turkey | male | 179121.55(146628.46,220333.20) | 363437.81(284840.45,453302.81) | 1.03 | 1011.05(831.00,1239.86) | 860.19(678.27,1066.49) | 0.14(-0.20,0.47) |
| Turkmenistan | male | 4453.49(3725.09,5339.06) | 17165.69(13552.52,21533.93) | 2.85 | 480.77(404.39,573.77) | 843.05(666.39,1058.62) | 1.48(1.21,1.75) |
| Uganda | male | 41123.37(31204.86,56111.53) | 101173.31(78327.98,131561.80) | 1.46 | 1279.07(966.70,1732.44) | 1540.80(1204.87,1971.89) | 0.30(0.06,0.55) |
| Ukraine | male | 79825.55(62135.37,101920.27) | 107679.44(81517.59,138726.73) | 0.35 | 298.96(232.79,381.50) | 382.07(289.57,491.63) | 0.41(0.23,0.59) |
| United Arab Emirates | male | 5081.33(3987.26,6415.17) | 69551.33(51958.58,89709.26) | 12.69 | 1765.17(1406.61,2187.74) | 1909.75(1484.31,2404.00) | 0.12(-0.14,0.39) |
| United Kingdom | male | 156377.01(125921.33,193271.18) | 341601.58(247536.91,455679.43) | 1.18 | 425.93(342.34,526.62) | 683.39(488.53,918.99) | 1.92(1.78,2.05) |
| United States of America | male | 903042.64(750044.04,1083212.28) | 2261986.78(1812209.17,2797894.34) | 1.50 | 676.87(561.70,813.01) | 928.08(742.48,1147.98) | 1.40(1.22,1.58) |
| Uruguay | male | 7183.47(6573.13,7859.38) | 14081.58(11759.52,17040.64) | 0.96 | 418.08(382.95,457.16) | 638.13(531.00,769.22) | 1.46(1.12,1.80) |
| Uzbekistan | male | 24449.67(20376.50,29075.73) | 154258.26(128366.20,187785.40) | 5.31 | 450.07(375.36,529.50) | 1378.99(1162.19,1663.15) | 3.81(3.37,4.25) |
| Vanuatu | male | 562.96(424.61,800.85) | 2422.32(1894.37,3162.82) | 3.30 | 1426.53(1079.69,2040.58) | 2479.23(1948.42,3196.62) | 2.03(1.95,2.10) |
| Venezuela (Bolivarian Republic of) | male | 57579.07(49305.79,66978.88) | 223020.28(175022.78,274521.29) | 2.87 | 1180.80(1018.72,1369.84) | 1589.27(1248.27,1948.62) | 0.96(0.80,1.11) |
| Viet nam | male | 120030.57(94225.21,147234.22) | 439142.31(350264.58,538410.93) | 2.66 | 703.25(556.41,864.34) | 1087.99(886.99,1322.03) | 1.82(1.66,1.99) |
| Virginia | male | 19086.75(15543.34,23394.41) | 57911.07(44919.65,72214.55) | 2.03 | 623.98(510.48,762.15) | 917.98(710.79,1142.66) | 1.67(1.46,1.87) |
| Yemen | male | 11046.63(8146.12,15179.90) | 43455.53(31683.67,57313.27) | 2.93 | 439.11(330.60,584.07) | 614.95(453.83,807.47) | 1.26(1.16,1.35) |
| Zambia | male | 18745.74(15671.25,22223.69) | 52583.05(42970.40,64329.20) | 1.81 | 1228.15(1028.41,1444.07) | 1524.33(1258.79,1829.13) | 0.44(0.06,0.81) |
| Zimbabwe | male | 19450.32(16358.72,23018.00) | 50028.86(40312.95,61285.07) | 1.57 | 914.55(771.99,1074.05) | 1548.42(1256.28,1873.31) | 1.67(1.24,2.11) |
| Monaco | male | 64.25(48.93,83.63) | 152.83(108.71,204.91) | 1.38 | 235.79(177.89,307.12) | 398.51(279.65,531.42) | 1.90(1.85,1.95) |
| San Marino | male | 47.23(36.33,61.14) | 126.08(93.13,167.31) | 1.67 | 320.01(246.43,412.35) | 459.41(333.52,610.42) | 1.29(1.23,1.35) |
| Saint Kitts and Nevis | male | 294.43(255.45,339.55) | 705.47(572.64,850.32) | 1.40 | 1873.46(1627.18,2165.81) | 2076.38(1715.69,2466.54) | -0.07(-0.23,0.09) |
| Cook Islands | male | 214.05(182.38,253.64) | 418.79(357.37,488.51) | 0.96 | 3098.64(2658.43,3644.18) | 3517.86(2989.19,4117.33) | 0.21(0.01,0.42) |
| Nauru | male | 73.33(54.26,107.16) | 120.39(85.45,183.82) | 0.64 | 3007.16(2280.10,4125.06) | 4738.51(3478.07,6919.72) | 1.32(1.00,1.63) |
| Niue | male | 25.36(20.59,31.59) | 41.93(32.60,51.72) | 0.65 | 2693.99(2191.64,3352.27) | 4174.14(3281.82,5154.71) | 1.39(1.11,1.67) |
| Palau | male | 122.08(99.37,151.54) | 473.05(366.28,599.39) | 2.87 | 2322.35(1915.07,2849.88) | 3902.08(3047.37,4877.23) | 1.73(1.47,1.99) |
| Tokelau | male | 8.90(7.31,10.77) | 15.37(12.21,19.02) | 0.73 | 1482.04(1213.23,1799.75) | 2287.49(1823.55,2828.93) | 1.51(1.32,1.70) |
| Tuvalu | male | 70.57(57.86,89.47) | 161.23(121.13,211.41) | 1.28 | 2245.72(1862.36,2785.16) | 3171.59(2426.32,4117.71) | 1.15(1.00,1.29) |
| Afghanistan | female | 46709.27(30450.26,70318.01) | 150540.31(99017.27,208491.06) | 2.22 | 1280.24(841.26,1907.17) | 2042.23(1331.65,2841.15) | 1.91(1.79,2.02) |
| Albania | female | 2705.83(2041.98,3514.40) | 6999.95(4968.81,9433.71) | 1.59 | 247.72(187.83,323.75) | 322.47(228.13,438.94) | 0.77(0.64,0.91) |
| Algeria | female | 43427.64(32178.50,57656.72) | 176919.12(134468.52,233003.10) | 3.07 | 700.39(520.52,932.90) | 1046.02(799.18,1365.35) | 1.71(1.55,1.87) |
| American Samoa | female | 290.56(239.18,346.53) | 915.68(746.85,1111.65) | 2.15 | 2407.84(1996.27,2849.70) | 3565.24(2916.69,4338.21) | 1.25(1.06,1.44) |
| Andorra | female | 58.73(42.81,77.69) | 222.45(160.49,302.66) | 2.79 | 221.76(162.77,290.70) | 327.13(233.87,445.53) | 1.32(1.19,1.46) |
| Angola | female | 19052.02(14352.43,24851.03) | 63864.49(50755.10,80214.71) | 2.35 | 894.70(677.09,1150.96) | 967.98(781.59,1204.46) | 0.25(0.19,0.30) |
| Antigua and Barbuda | female | 481.52(417.35,557.07) | 1040.01(855.25,1240.74) | 1.16 | 1631.43(1403.29,1892.70) | 1930.58(1599.17,2289.01) | 0.18(0.04,0.32) |
| Argentina | female | 106634.17(91875.45,123840.30) | 191515.89(155037.65,236418.98) | 0.80 | 592.35(510.23,687.08) | 649.79(523.05,803.00) | -0.04(-0.19,0.10) |
| Armenia | female | 11494.13(9833.63,13498.49) | 25496.33(20720.11,30888.85) | 1.22 | 724.59(621.51,851.45) | 1076.32(867.07,1307.09) | 0.70(0.13,1.26) |
| Australia | female | 34217.57(28583.97,40862.09) | 75906.18(57559.59,96906.65) | 1.22 | 318.70(265.21,381.98) | 356.45(267.31,460.76) | 0.21(0.05,0.37) |
| Austria | female | 26273.11(22175.32,31389.81) | 39782.88(31007.78,51043.84) | 0.51 | 354.49(296.09,430.78) | 415.73(316.00,544.79) | 0.60(0.39,0.82) |
| Azerbaijan | female | 15318.80(12591.02,18891.46) | 54189.46(42453.39,67731.37) | 2.54 | 505.70(415.84,620.76) | 984.95(775.88,1232.08) | 2.01(1.60,2.42) |
| Bahrain | female | 1524.55(1270.97,1868.30) | 11309.79(9074.84,13782.39) | 6.42 | 1952.21(1640.07,2383.60) | 3200.82(2597.54,3885.57) | 1.81(1.46,2.16) |
| Bangladesh | female | 168306.36(132264.22,216322.82) | 566434.37(449319.21,699886.16) | 2.37 | 724.90(580.08,895.66) | 896.38(714.23,1093.75) | 0.81(0.52,1.09) |
| Barbados | female | 3360.27(2984.33,3781.30) | 4980.42(4103.47,5937.34) | 0.48 | 2039.44(1797.80,2299.86) | 1909.26(1564.53,2278.00) | -0.40(-0.50,-0.31) |
| Belarus | female | 23805.44(18323.70,30139.84) | 24304.85(17083.95,32878.69) | 0.02 | 302.36(231.59,384.35) | 268.30(186.35,366.59) | -0.99(-1.25,-0.73) |
| Belgium | female | 35347.03(28688.60,43379.83) | 46945.16(34494.89,61842.26) | 0.33 | 395.58(318.03,492.25) | 409.32(291.44,550.74) | -0.20(-0.29,-0.10) |
| Belize | female | 732.17(635.91,834.61) | 2891.78(2448.80,3404.85) | 2.95 | 1542.73(1339.46,1763.40) | 1999.55(1710.69,2349.08) | 1.03(0.58,1.47) |
| Benin | female | 7375.76(6028.61,8875.09) | 28470.27(22517.15,35624.47) | 2.86 | 699.19(573.01,837.61) | 1057.17(837.65,1310.80) | 1.50(1.34,1.66) |
| Bermuda | female | 301.95(256.16,350.23) | 445.02(343.46,563.73) | 0.47 | 857.30(727.57,994.83) | 669.87(512.57,855.85) | -1.34(-1.55,-1.14) |
| Bhutan | female | 866.52(654.89,1103.54) | 2681.70(2121.37,3411.42) | 2.09 | 651.23(493.90,826.53) | 969.80(772.45,1232.58) | 1.34(1.29,1.40) |
| Bolivia (Plurinational State of) | female | 23269.09(18281.69,29124.81) | 67476.23(53210.95,84045.82) | 1.90 | 1327.98(1041.90,1657.67) | 1448.06(1146.23,1798.44) | 0.18(0.09,0.27) |
| Bosnia and Herzegovina | female | 14279.73(11719.90,17267.03) | 40339.36(31163.65,49736.14) | 1.82 | 622.83(515.28,745.73) | 1209.10(934.31,1495.36) | 2.88(2.51,3.25) |
| Botswana | female | 4840.76(3672.43,6338.56) | 17717.67(12965.00,23830.86) | 2.66 | 1511.57(1148.56,1977.52) | 2283.34(1692.17,3067.94) | 1.59(1.36,1.82) |
| Brazil | female | 552970.00(487092.53,630574.07) | 1244810.22(1048593.85,1463667.50) | 1.25 | 1131.92(1001.86,1287.65) | 956.47(806.23,1124.57) | -0.57(-0.62,-0.51) |
| Brunei Darussalam | female | 1329.67(1091.19,1588.91) | 3142.84(2574.91,3840.40) | 1.36 | 2638.92(2164.31,3129.43) | 1948.59(1623.45,2340.67) | -0.85(-1.04,-0.66) |
| Bulgaria | female | 42343.02(34839.33,50710.27) | 51487.92(39467.65,65055.01) | 0.22 | 625.00(513.94,751.16) | 664.37(504.19,839.37) | -0.22(-0.44,0.00) |
| Burkina Faso | female | 18227.27(14331.78,22708.16) | 47662.49(37877.54,58877.99) | 1.61 | 786.36(619.41,964.34) | 902.70(723.77,1107.49) | 0.41(0.33,0.49) |
| Burundi | female | 13868.53(10503.94,18380.13) | 20750.85(16372.38,25933.13) | 0.50 | 1039.59(796.17,1355.09) | 894.33(715.88,1109.44) | -0.83(-0.96,-0.70) |
| Cambodia | female | 25627.54(19513.49,32962.99) | 77805.79(60984.94,97588.95) | 2.04 | 926.51(715.81,1166.37) | 1108.96(877.07,1386.35) | 0.41(0.26,0.56) |
| Cameroon | female | 19668.49(15487.34,24367.38) | 81534.12(61766.38,105638.71) | 3.15 | 845.27(667.39,1038.93) | 1278.15(973.86,1630.63) | 1.58(1.30,1.87) |
| Canada | female | 52934.64(45590.68,61428.93) | 117811.49(91554.38,149175.90) | 1.23 | 287.64(247.57,334.20) | 331.65(254.11,424.12) | -0.27(-0.56,0.03) |
| Cabo Verde | female | 544.33(422.17,718.52) | 2505.41(2007.09,3063.93) | 3.60 | 410.79(319.22,539.07) | 1034.05(827.62,1267.03) | 2.98(2.48,3.47) |
| Central African Republic | female | 6671.32(5213.86,8436.57) | 15581.99(11645.68,20396.10) | 1.34 | 985.29(767.49,1235.57) | 1209.52(911.71,1562.77) | 0.84(0.64,1.04) |
| Chad | female | 10289.75(7991.63,13063.75) | 30556.50(23052.40,38629.09) | 1.97 | 697.00(543.04,879.17) | 1119.40(854.36,1418.78) | 1.75(1.50,2.00) |
| Chile | female | 30525.51(25471.60,37009.98) | 87440.45(67728.33,111791.80) | 1.86 | 556.38(464.80,672.94) | 667.29(515.95,853.28) | 0.61(0.47,0.76) |
| China | female | 2024978.27(1613886.71,2491805.68) | 4532120.89(3558126.68,5675222.99) | 1.24 | 449.52(359.42,552.80) | 435.37(341.79,546.02) | 0.00(-0.27,0.27) |
| Colombia | female | 92718.22(76335.89,112153.48) | 238874.40(181776.75,307712.83) | 1.58 | 979.97(809.43,1183.65) | 835.67(632.80,1075.75) | -1.14(-1.39,-0.90) |
| Comoros | female | 1033.67(678.00,1323.46) | 2354.93(1909.61,2917.43) | 1.28 | 889.63(601.03,1128.56) | 886.23(725.22,1095.23) | -0.14(-0.25,-0.04) |
| Congo | female | 7408.65(5849.64,9229.15) | 19970.21(15442.82,25596.85) | 1.70 | 1192.13(935.87,1483.41) | 1379.69(1081.73,1738.86) | 0.55(0.36,0.73) |
| Costa Rica | female | 7492.81(6135.55,9140.26) | 21664.13(16248.34,28307.72) | 1.89 | 809.57(666.32,985.56) | 780.49(586.21,1019.82) | -0.58(-0.84,-0.32) |
| C么te d'Ivoire | female | 14532.31(11738.38,17598.05) | 60048.56(46043.38,74523.01) | 3.13 | 749.65(611.85,893.36) | 1135.22(892.23,1387.56) | 1.60(1.32,1.88) |
| Croatia | female | 19936.68(15978.95,24975.33) | 28961.00(21912.46,38343.48) | 0.45 | 527.62(420.42,662.97) | 608.14(449.15,811.80) | 0.15(0.03,0.27) |
| Cuba | female | 54515.16(45815.47,64269.36) | 75063.43(56536.16,98152.67) | 0.38 | 1042.08(877.31,1229.94) | 795.82(594.87,1041.93) | -1.17(-1.59,-0.75) |
| Cyprus | female | 5595.54(4127.78,6680.82) | 7165.29(5559.26,8997.17) | 0.28 | 1361.75(1007.95,1611.96) | 703.70(543.99,883.68) | -2.75(-2.89,-2.61) |
| Czechia | female | 47945.45(37413.22,61043.89) | 95489.22(70533.31,125180.96) | 0.99 | 598.86(465.13,766.30) | 859.50(623.02,1129.48) | 1.66(1.31,2.01) |
| Democratic Republic of the Congo | female | 73248.42(58330.99,89832.58) | 182131.02(143309.63,228351.70) | 1.49 | 822.74(662.68,996.24) | 874.32(688.07,1084.17) | 0.22(0.18,0.27) |
| Denmark | female | 11067.45(9490.30,12992.18) | 19773.15(15982.71,24428.22) | 0.79 | 254.25(215.05,300.96) | 347.83(272.35,436.95) | 0.90(0.69,1.11) |
| Djibouti | female | 588.13(448.43,756.06) | 2739.60(2074.05,3520.65) | 3.66 | 813.10(631.49,1022.17) | 962.60(755.16,1211.39) | 0.63(0.57,0.69) |
| Dominica | female | 813.68(704.67,930.55) | 932.14(767.55,1123.44) | 0.15 | 2008.89(1719.77,2312.39) | 2077.85(1705.94,2520.11) | -0.05(-0.17,0.08) |
| Dominican Republic | female | 15117.44(12784.39,17838.96) | 50576.64(40412.59,62274.17) | 2.35 | 737.78(622.75,866.55) | 1028.81(822.82,1264.45) | 1.42(1.27,1.57) |
| Ecuador | female | 22741.06(19812.35,26635.25) | 92347.20(74770.93,112941.07) | 3.06 | 825.39(718.37,964.31) | 1168.56(950.34,1428.00) | 1.16(0.90,1.43) |
| Egypt | female | 122623.72(106580.29,141598.38) | 406682.94(319731.61,519030.99) | 2.32 | 817.27(711.54,942.81) | 1343.94(1057.53,1709.72) | 2.13(1.99,2.26) |
| El Salvador | female | 12266.20(10258.02,14561.00) | 48358.20(37966.06,59865.33) | 2.94 | 750.30(627.53,886.16) | 1433.54(1126.98,1775.92) | 2.18(2.01,2.35) |
| Equatorial Guinea | female | 1037.24(761.95,1370.55) | 3559.18(2546.68,4777.56) | 2.43 | 888.67(653.04,1176.53) | 1222.41(891.43,1637.77) | 1.42(1.25,1.60) |
| Eritrea | female | 5741.28(3785.01,8253.06) | 18142.00(13889.33,23575.00) | 2.16 | 932.25(613.41,1348.79) | 1146.50(876.54,1473.53) | 0.77(0.66,0.89) |
| Estonia | female | 3437.52(2548.71,4494.13) | 5401.27(3917.42,7141.52) | 0.57 | 276.31(205.60,362.62) | 389.83(277.27,520.75) | 1.16(0.72,1.59) |
| Ethiopia | female | 131563.46(99787.59,171397.45) | 159623.23(132930.84,189299.39) | 0.21 | 1252.74(950.21,1603.63) | 764.15(650.75,889.72) | -2.14(-2.30,-1.99) |
| Micronesia (Federated States of) | female | 685.84(531.24,884.23) | 1910.21(1417.23,2539.44) | 1.79 | 2766.03(2153.10,3549.13) | 4825.93(3626.53,6332.83) | 1.88(1.54,2.21) |
| Fiji | female | 8431.11(6730.63,11364.35) | 27347.65(22007.82,32905.90) | 2.24 | 4238.75(3408.24,5759.26) | 6667.04(5420.76,7986.93) | 1.27(0.85,1.68) |
| Finland | female | 14991.92(11577.13,19300.12) | 25721.45(18058.15,34999.51) | 0.72 | 356.29(271.02,460.28) | 457.99(315.27,626.17) | 0.92(0.80,1.03) |
| France | female | 96990.98(82423.18,113343.78) | 170080.91(134363.90,210518.69) | 0.75 | 195.93(164.82,232.10) | 231.74(177.68,293.82) | 0.55(0.34,0.75) |
| Gabon | female | 3215.70(2539.56,4025.31) | 7292.23(5577.90,9365.86) | 1.27 | 1034.03(816.61,1286.01) | 1277.31(984.57,1633.07) | 0.75(0.34,1.17) |
| Georgia | female | 16667.95(13357.77,20521.96) | 29806.69(23549.97,37015.62) | 0.79 | 450.61(358.55,555.47) | 893.29(697.91,1111.86) | 1.94(1.17,2.73) |
| Germany | female | 419392.21(346311.96,511002.80) | 507233.91(383863.24,658566.15) | 0.21 | 537.44(437.54,661.19) | 532.14(392.05,708.01) | -0.48(-0.84,-0.11) |
| Ghana | female | 30570.82(24428.26,37798.15) | 96500.39(78161.39,120988.55) | 2.16 | 896.63(725.17,1101.48) | 1038.46(847.37,1289.69) | 0.57(0.35,0.79) |
| Greece | female | 27372.53(21552.24,34383.23) | 44071.23(31985.81,59387.07) | 0.61 | 332.89(261.24,418.93) | 392.33(280.99,533.11) | 0.58(0.49,0.68) |
| Greenland | female | 62.40(50.48,74.35) | 101.81(78.08,128.86) | 0.63 | 369.30(298.13,437.44) | 314.50(241.98,396.10) | -1.49(-1.87,-1.11) |
| Grenada | female | 853.37(746.63,971.64) | 1309.91(1110.42,1539.25) | 0.53 | 2163.47(1878.55,2472.69) | 2249.57(1910.71,2642.37) | 0.18(0.07,0.29) |
| Guam | female | 552.70(457.92,658.35) | 1131.37(912.39,1406.48) | 1.05 | 1415.09(1174.25,1663.27) | 1183.32(950.25,1467.95) | -0.91(-1.29,-0.54) |
| Guatemala | female | 15701.86(12715.99,19151.04) | 144578.22(116212.62,175243.15) | 8.21 | 796.13(644.53,973.72) | 2268.28(1829.98,2739.94) | 3.29(2.86,3.72) |
| Guinea | female | 13997.12(10850.73,17379.51) | 34068.72(26800.56,42650.02) | 1.43 | 831.89(645.09,1030.78) | 1201.83(944.47,1501.01) | 1.38(1.29,1.48) |
| Guinea-Bissau | female | 2176.40(1619.49,2898.35) | 6006.21(4557.85,7784.77) | 1.76 | 976.68(731.03,1270.47) | 1421.27(1091.20,1820.41) | 1.35(1.13,1.58) |
| Guyana | female | 5821.97(4969.78,6753.43) | 11014.07(8843.22,13408.17) | 0.89 | 2756.32(2353.07,3172.41) | 3163.60(2560.67,3839.50) | 0.15(-0.27,0.58) |
| Haiti | female | 57451.01(43172.62,72574.12) | 111210.06(81473.46,149852.36) | 0.94 | 3129.73(2377.59,3906.29) | 2770.56(2029.74,3698.17) | -0.32(-0.39,-0.26) |
| Honduras | female | 8690.27(6755.86,10870.18) | 39084.65(29744.78,50288.99) | 3.50 | 743.63(579.44,929.57) | 1125.32(864.38,1441.87) | 1.34(1.22,1.46) |
| Hungary | female | 47812.55(38492.37,58533.20) | 67430.25(51121.86,86310.27) | 0.41 | 562.25(449.24,688.23) | 628.40(473.17,805.90) | 0.54(0.36,0.72) |
| Iceland | female | 324.24(248.22,419.72) | 831.55(579.14,1129.42) | 1.56 | 213.94(161.51,280.30) | 314.12(218.12,431.08) | 1.12(1.06,1.19) |
| India | female | 1512995.63(1211392.53,1882635.38) | 5599395.80(4501334.29,6836406.89) | 2.70 | 671.86(541.57,834.59) | 943.76(762.49,1149.53) | 0.96(0.86,1.06) |
| Indonesia | female | 569943.29(478563.01,675164.80) | 1771039.60(1433121.20,2151472.43) | 2.11 | 987.76(834.90,1161.90) | 1448.00(1187.93,1741.73) | 1.52(1.37,1.66) |
| Iran (Islamic Republic of) | female | 67898.97(55242.74,83144.99) | 372689.16(295913.69,461409.38) | 4.49 | 534.00(438.41,648.37) | 1002.62(803.18,1235.04) | 2.47(2.37,2.57) |
| Iraq | female | 69550.45(56545.56,83939.09) | 200344.08(159015.95,247370.57) | 1.88 | 1632.88(1322.43,1978.59) | 1598.57(1285.30,1960.93) | -0.21(-0.31,-0.10) |
| Ireland | female | 5508.36(4700.31,6502.73) | 12499.21(9202.66,16517.14) | 1.27 | 244.18(206.79,289.52) | 330.14(241.91,438.46) | 0.98(0.71,1.25) |
| Israel | female | 13466.76(11450.64,16008.47) | 38176.52(31020.86,46684.25) | 1.83 | 513.84(436.91,610.49) | 612.33(489.84,756.79) | -0.19(-0.83,0.44) |
| Italy | female | 295715.91(256562.49,340864.03) | 384779.99(306033.50,475631.72) | 0.30 | 575.18(492.93,674.00) | 511.27(391.71,651.03) | -0.40(-0.56,-0.24) |
| Jamaica | female | 18800.96(16927.16,20831.75) | 37594.69(31080.00,45143.76) | 1.00 | 2008.95(1807.91,2225.33) | 2424.86(1996.49,2912.08) | 0.24(-0.02,0.51) |
| Japan | female | 243729.61(194838.98,303616.23) | 378122.56(274121.73,507456.29) | 0.55 | 257.29(205.03,321.30) | 241.26(171.78,326.70) | -0.63(-0.93,-0.34) |
| Jordan | female | 12150.01(10081.84,14420.09) | 37390.53(29580.01,45881.28) | 2.08 | 1916.01(1572.95,2281.48) | 1227.99(985.11,1475.60) | -2.07(-2.61,-1.52) |
| Kazakhstan | female | 41523.42(32032.31,52971.98) | 96418.80(74069.34,121480.03) | 1.32 | 529.53(408.60,675.36) | 933.04(717.49,1177.49) | 1.67(1.36,1.97) |
| Kenya | female | 26715.00(22735.23,31605.21) | 82310.86(65859.11,102065.96) | 2.08 | 620.45(530.57,723.69) | 678.00(551.03,832.82) | 0.40(0.33,0.47) |
| Kiribati | female | 1019.25(823.77,1246.83) | 2317.26(1796.47,2903.03) | 1.27 | 4503.36(3638.70,5470.86) | 5329.06(4168.98,6639.94) | 0.32(0.09,0.54) |
| Kuwait | female | 2921.94(2376.90,3591.26) | 13543.32(9568.75,18306.11) | 3.64 | 1136.18(942.73,1382.58) | 1020.52(737.57,1336.31) | -0.49(-0.89,-0.10) |
| Kyrgyzstan | female | 6224.32(4972.19,7661.03) | 12508.55(9625.91,16118.53) | 1.01 | 342.13(272.35,418.74) | 452.66(348.96,578.64) | 0.37(0.12,0.62) |
| Lao People's Democratic Republic | female | 15523.75(11359.61,21446.81) | 34391.42(25854.42,43181.81) | 1.22 | 1321.55(976.21,1792.01) | 1451.79(1104.21,1814.02) | 0.13(0.06,0.21) |
| Latvia | female | 6741.58(5215.75,8615.43) | 10142.08(7606.48,13166.93) | 0.50 | 312.32(240.83,400.03) | 464.39(342.38,606.32) | 1.40(1.16,1.64) |
| Lebanon | female | 8696.51(6869.61,10861.97) | 24534.68(18341.75,32522.93) | 1.82 | 740.76(590.89,919.76) | 860.87(643.72,1142.27) | 0.51(0.44,0.58) |
| Lesotho | female | 6754.37(5336.45,8216.49) | 20068.25(13844.36,26713.71) | 1.97 | 1209.78(961.53,1471.20) | 2665.16(1863.35,3519.23) | 3.89(3.38,4.40) |
| Liberia | female | 4619.20(3691.79,5513.61) | 12787.49(9818.39,16077.58) | 1.77 | 887.98(717.15,1059.18) | 1216.40(938.55,1509.94) | 1.27(1.12,1.43) |
| Libya | female | 6381.78(4912.55,8122.13) | 30843.63(22921.55,39909.52) | 3.83 | 706.74(544.90,899.04) | 1154.01(864.16,1481.41) | 1.97(1.84,2.09) |
| Lithuania | female | 6855.72(5163.50,8937.85) | 9165.62(6739.33,12119.46) | 0.34 | 256.92(193.28,336.13) | 298.40(219.03,397.34) | 0.52(0.36,0.69) |
| Luxembourg | female | 909.07(768.08,1078.40) | 2488.67(1783.04,3345.93) | 1.74 | 284.18(237.49,340.00) | 488.77(345.77,662.80) | 1.90(1.63,2.17) |
| North Macedonia | female | 7776.99(6382.97,9440.56) | 20967.94(16640.03,26085.48) | 1.70 | 799.17(661.45,967.14) | 1237.16(980.29,1541.09) | 1.74(1.48,2.00) |
| Madagascar | female | 21754.29(17939.96,25985.22) | 52057.60(40432.41,64356.40) | 1.39 | 800.33(664.20,945.38) | 855.59(673.14,1054.33) | 0.13(0.04,0.21) |
| Malawi | female | 20885.21(17037.26,25161.29) | 35151.11(28590.58,43291.54) | 0.68 | 983.23(803.73,1172.68) | 842.94(690.18,1030.34) | -0.66(-0.74,-0.58) |
| Malaysia | female | 50028.25(42074.53,58729.59) | 113967.53(85832.07,151030.34) | 1.28 | 1022.51(866.28,1193.72) | 830.70(626.38,1096.27) | -0.93(-1.10,-0.76) |
| Maldives | female | 469.11(343.96,640.28) | 1031.74(801.14,1302.93) | 1.20 | 1158.19(876.85,1519.40) | 730.35(572.90,922.41) | -2.01(-2.42,-1.60) |
| Mali | female | 19358.78(15305.46,23545.30) | 53695.01(41405.67,66303.50) | 1.77 | 899.96(721.60,1089.96) | 1240.08(972.38,1524.96) | 1.03(0.88,1.18) |
| Malta | female | 1687.33(1426.59,2010.82) | 2779.79(2126.53,3613.17) | 0.65 | 706.00(595.94,843.54) | 603.36(453.27,787.49) | -0.59(-0.75,-0.44) |
| Marshall Islands | female | 263.26(214.90,321.23) | 941.89(718.64,1227.13) | 2.58 | 2930.51(2383.62,3561.49) | 4840.33(3715.12,6239.09) | 1.60(1.29,1.90) |
| Mauritania | female | 4857.08(3874.31,5969.93) | 10926.75(8263.67,13552.91) | 1.25 | 914.28(734.79,1119.76) | 1049.02(803.58,1295.59) | 0.48(0.32,0.65) |
| Mauritius | female | 6014.84(5269.39,6916.78) | 27038.77(22138.30,32102.19) | 3.50 | 1458.13(1279.11,1671.14) | 2844.53(2325.90,3369.04) | 3.59(2.92,4.27) |
| Mexico | female | 529585.00(468918.09,598968.19) | 1316540.57(1091423.37,1581573.00) | 1.49 | 2289.25(2033.96,2584.76) | 2069.19(1722.78,2484.29) | -0.54(-0.67,-0.41) |
| Republic of Moldova | female | 11934.49(9421.98,15071.40) | 15060.78(10906.41,19803.43) | 0.26 | 452.51(355.27,570.58) | 465.42(338.05,612.20) | -0.13(-0.32,0.07) |
| Mongolia | female | 1274.91(969.47,1665.59) | 3938.52(2877.15,5259.19) | 2.09 | 210.75(160.67,276.36) | 271.55(198.22,358.45) | 0.89(0.79,0.99) |
| Montenegro | female | 2005.77(1547.83,2518.60) | 4211.96(3195.16,5441.12) | 1.10 | 578.61(446.46,724.85) | 794.72(597.73,1035.65) | 1.08(0.96,1.20) |
| Morocco | female | 42101.37(32972.92,57202.30) | 176715.74(136020.52,226227.79) | 3.20 | 587.09(463.16,799.75) | 1080.38(835.06,1372.98) | 2.44(2.28,2.60) |
| Mozambique | female | 27120.23(21659.58,33758.14) | 59152.41(46516.91,76403.41) | 1.18 | 833.92(673.28,1017.79) | 925.19(736.59,1195.02) | 0.50(0.31,0.69) |
| Myanmar | female | 216058.65(161195.32,288931.78) | 380185.92(312851.16,461038.59) | 0.76 | 1637.20(1241.94,2137.90) | 1437.74(1190.78,1734.35) | -0.65(-0.72,-0.58) |
| Namibia | female | 5997.60(4799.29,7254.98) | 11888.43(8898.61,15258.27) | 0.98 | 1506.28(1208.75,1817.34) | 1483.35(1112.86,1898.90) | -0.41(-0.78,-0.05) |
| Nepal | female | 19159.70(14305.53,25136.71) | 78369.41(59464.61,101256.56) | 3.09 | 405.93(306.17,524.91) | 682.47(527.12,865.05) | 1.76(1.60,1.91) |
| Netherlands | female | 53509.69(45440.29,62410.57) | 59177.89(45774.36,74925.89) | 0.11 | 458.65(387.70,537.84) | 340.28(256.82,436.39) | -1.34(-1.52,-1.17) |
| New Zealand | female | 6898.52(5880.00,8041.55) | 13375.07(10532.04,16633.74) | 0.94 | 333.77(283.62,388.84) | 343.78(268.64,429.26) | -0.66(-0.99,-0.32) |
| Nicaragua | female | 9310.42(7825.80,11096.29) | 40055.12(32842.19,49354.40) | 3.30 | 1066.93(898.11,1270.98) | 1589.13(1312.91,1942.70) | 1.09(0.83,1.36) |
| Niger | female | 8533.18(6391.86,11160.31) | 38637.41(30101.28,48870.33) | 3.53 | 617.95(470.46,789.27) | 939.23(735.17,1163.96) | 1.32(1.16,1.47) |
| Nigeria | female | 146081.87(115154.74,180544.47) | 355971.53(270786.12,452776.01) | 1.44 | 693.25(551.22,854.80) | 778.17(607.05,968.24) | 0.28(0.22,0.34) |
| Democratic People's Republic of Korea | female | 51987.05(40349.69,65101.98) | 110343.86(85288.71,140577.90) | 1.12 | 517.77(403.64,647.19) | 599.37(463.29,764.11) | 0.50(0.43,0.57) |
| Northern Mariana Islands | female | 150.41(121.19,186.65) | 520.87(427.83,645.86) | 2.46 | 1748.48(1421.42,2152.48) | 1904.44(1575.71,2355.83) | 0.47(0.22,0.71) |
| Norway | female | 11578.93(9066.60,14642.66) | 18134.52(13640.48,23680.36) | 0.57 | 319.54(246.52,408.86) | 387.20(285.29,514.83) | -0.02(-0.19,0.16) |
| Oman | female | 4093.63(3123.47,5123.98) | 11505.98(9386.78,13987.11) | 1.81 | 1351.13(1035.20,1685.78) | 1544.00(1284.10,1844.97) | 0.50(0.33,0.67) |
| Pakistan | female | 204840.00(161025.76,251280.78) | 939874.89(697828.75,1175230.98) | 3.59 | 757.42(594.08,923.81) | 1652.05(1221.20,2046.60) | 2.91(2.68,3.14) |
| Palestine | female | 6667.18(5386.58,8180.26) | 21178.67(17709.40,25360.13) | 2.18 | 1422.55(1150.70,1739.63) | 1790.08(1515.61,2122.53) | 0.85(0.53,1.17) |
| Panama | female | 6300.82(5222.88,7652.26) | 25737.05(20020.44,31747.27) | 3.08 | 836.12(695.92,1011.91) | 1210.08(941.39,1493.96) | 1.05(0.90,1.20) |
| Papua New Guinea | female | 15973.53(12439.59,20749.22) | 66222.52(51468.10,85269.54) | 3.15 | 1543.78(1207.46,2015.14) | 2452.37(1933.61,3127.57) | 1.61(1.46,1.76) |
| Paraguay | female | 9787.09(8223.13,11549.02) | 39232.22(30692.20,48003.68) | 3.01 | 835.28(703.68,985.43) | 1353.46(1060.84,1655.27) | 1.73(1.36,2.09) |
| Peru | female | 29576.12(24402.92,35660.87) | 99646.01(78820.02,123977.51) | 2.37 | 475.19(392.08,573.65) | 598.48(473.81,744.15) | 0.70(0.50,0.90) |
| Philippines | female | 143056.33(122404.22,167156.39) | 481226.74(390876.64,579010.98) | 2.36 | 915.39(788.52,1068.64) | 1131.25(927.22,1360.40) | 0.77(0.71,0.84) |
| Poland | female | 158866.30(129977.28,192654.67) | 229471.58(174074.06,293902.32) | 0.44 | 623.79(508.39,756.81) | 603.02(452.99,778.80) | 0.11(-0.27,0.48) |
| Portugal | female | 51174.35(43611.41,60530.31) | 82080.94(63901.37,103707.44) | 0.60 | 638.81(540.15,760.91) | 638.46(481.33,823.13) | -0.36(-0.55,-0.18) |
| Puerto Rico | female | 28020.43(23695.96,33015.85) | 55125.72(43962.38,68431.11) | 0.97 | 1438.06(1215.32,1692.43) | 1530.84(1200.69,1911.84) | -0.12(-0.37,0.12) |
| Qatar | female | 885.64(726.29,1070.78) | 7198.27(5468.89,9402.50) | 7.13 | 2575.20(2107.63,3102.36) | 3628.58(2952.61,4381.58) | 1.69(1.04,2.35) |
| Romania | female | 56518.90(44736.71,70587.58) | 83352.03(62900.94,108767.39) | 0.47 | 365.89(289.05,456.11) | 426.51(318.90,558.10) | 0.49(0.36,0.62) |
| Russian Federation | female | 318964.03(256789.66,391299.29) | 563303.53(446787.26,690339.39) | 0.77 | 279.57(223.97,344.37) | 395.82(308.98,487.61) | 1.05(0.81,1.29) |
| Rwanda | female | 19274.02(14730.11,24908.09) | 29490.29(23143.57,38205.24) | 0.53 | 1142.01(868.44,1475.63) | 840.24(667.84,1083.81) | -1.62(-1.88,-1.36) |
| Saint Lucia | female | 1388.74(1208.69,1583.18) | 2608.31(2085.36,3162.08) | 0.88 | 2822.84(2458.22,3217.56) | 2312.86(1845.64,2815.46) | -1.36(-1.62,-1.10) |
| Saint Vincent and the Grenadines | female | 1113.14(980.09,1254.43) | 1765.27(1493.52,2076.92) | 0.59 | 2792.62(2469.47,3147.40) | 2691.84(2279.54,3163.70) | -0.56(-0.78,-0.35) |
| Samoa | female | 879.57(681.91,1171.95) | 2280.70(1785.46,2894.05) | 1.59 | 1918.82(1490.01,2553.99) | 2987.55(2345.41,3771.69) | 1.51(1.22,1.80) |
| Sao Tome and Principe | female | 148.10(114.63,187.96) | 342.16(262.08,445.86) | 1.31 | 437.71(339.62,551.43) | 609.43(472.49,791.36) | 1.10(0.95,1.26) |
| Saudi Arabia | female | 24624.29(19276.44,30484.56) | 92536.48(70820.20,120556.88) | 2.76 | 914.07(722.85,1127.89) | 994.63(771.07,1275.59) | -0.14(-0.34,0.05) |
| Senegal | female | 13949.01(11112.64,17283.26) | 51014.19(39524.24,63923.44) | 2.66 | 851.24(683.97,1050.94) | 1284.82(1002.55,1595.34) | 1.66(1.44,1.89) |
| Serbia | female | 45482.31(36599.39,55274.24) | 75218.17(59855.91,93407.89) | 0.65 | 753.97(609.26,913.93) | 871.52(687.24,1084.37) | 0.47(0.36,0.58) |
| Seychelles | female | 198.70(158.58,245.97) | 748.99(577.09,951.18) | 2.77 | 624.79(495.83,775.58) | 1310.01(1015.87,1661.33) | 2.64(2.45,2.82) |
| Sierra Leone | female | 5059.00(3938.04,6243.95) | 18863.05(14480.68,24332.66) | 2.73 | 543.22(425.73,671.00) | 1015.43(785.05,1300.03) | 2.51(2.23,2.79) |
| Singapore | female | 9846.55(8132.51,11932.49) | 15388.69(10607.37,21471.18) | 0.56 | 798.39(664.98,962.08) | 385.85(265.76,537.51) | -2.43(-2.74,-2.11) |
| Slovakia | female | 17466.18(14229.06,21386.37) | 25802.62(19440.65,33977.10) | 0.48 | 506.89(409.03,622.57) | 505.22(376.35,666.34) | -0.05(-0.15,0.06) |
| Slovenia | female | 7010.09(5427.37,8936.29) | 9992.85(7258.00,13169.22) | 0.43 | 476.17(366.61,608.49) | 449.41(322.66,596.03) | -1.17(-1.53,-0.80) |
| Solomon Islands | female | 1996.03(1459.36,3316.08) | 8437.55(6785.41,10545.95) | 3.23 | 2609.54(1917.90,4300.72) | 4426.19(3623.40,5468.05) | 1.81(1.63,1.98) |
| Somalia | female | 14915.92(10441.64,20284.16) | 43476.77(32488.85,57066.72) | 1.91 | 1029.17(729.29,1368.12) | 1090.80(818.27,1422.12) | 0.40(0.33,0.46) |
| South Africa | female | 155525.66(135251.93,177116.38) | 467505.07(407555.72,533646.77) | 2.01 | 1270.31(1104.07,1448.85) | 1812.71(1585.28,2064.86) | 1.61(1.19,2.04) |
| Republic of Korea | female | 94120.47(77773.17,117741.65) | 239058.88(184970.09,303035.60) | 1.54 | 532.19(440.61,667.04) | 500.80(384.33,638.46) | -1.00(-1.55,-0.44) |
| South Sudan | female | 8950.07(6794.77,11548.06) | 15856.62(11958.31,20434.97) | 0.77 | 825.69(628.01,1054.11) | 828.25(631.90,1050.51) | 0.05(0.00,0.10) |
| Spain | female | 191228.36(156299.63,233098.42) | 247327.50(183490.11,331375.40) | 0.29 | 601.99(486.92,737.85) | 490.61(347.68,666.24) | -0.81(-0.92,-0.70) |
| Sri Lanka | female | 42680.09(34507.76,52453.75) | 248442.75(192500.44,309470.85) | 4.82 | 794.37(650.84,967.11) | 1769.51(1368.89,2192.59) | 3.18(2.84,3.52) |
| Sudan | female | 24898.32(18381.50,33494.11) | 80583.57(59599.53,104097.40) | 2.24 | 534.22(396.52,718.61) | 852.79(641.34,1101.74) | 1.88(1.76,2.00) |
| Suriname | female | 1937.35(1617.98,2279.99) | 6052.82(4881.02,7405.49) | 2.12 | 1385.51(1161.24,1629.05) | 1857.06(1497.98,2267.34) | 1.09(0.92,1.27) |
| Eswatini | female | 3164.27(2414.05,3876.92) | 9368.46(6270.87,12992.28) | 1.96 | 1923.24(1468.62,2364.14) | 2724.94(1836.43,3750.13) | 1.77(1.12,2.43) |
| Sweden | female | 22890.09(18813.29,27858.28) | 34703.37(27088.19,43921.32) | 0.52 | 285.32(232.13,349.75) | 341.67(259.33,444.20) | 0.72(0.60,0.84) |
| Switzerland | female | 24507.32(20455.51,29684.28) | 31438.70(23271.16,41261.85) | 0.28 | 397.46(325.04,487.98) | 355.04(255.75,477.06) | -0.64(-0.76,-0.53) |
| Syrian Arab Republic | female | 22204.45(17781.86,27454.28) | 55219.71(41558.05,72786.83) | 1.49 | 824.28(662.92,1013.65) | 898.17(684.09,1169.51) | -0.19(-0.40,0.02) |
| Taiwan (Province of China) | female | 81204.50(70819.47,93456.05) | 171641.34(136184.00,215099.38) | 1.11 | 1084.55(953.08,1238.99) | 817.06(644.96,1026.23) | -1.90(-2.31,-1.48) |
| Tajikistan | female | 8202.64(6864.51,9869.06) | 35537.83(27219.15,43791.93) | 3.33 | 508.12(423.86,611.43) | 1196.72(923.22,1480.98) | 3.35(3.10,3.60) |
| United Republic of Tanzania | female | 44274.21(36289.83,52111.44) | 115981.04(93986.12,140982.00) | 1.62 | 781.25(644.84,912.65) | 878.26(720.56,1063.17) | 0.66(0.53,0.79) |
| Thailand | female | 169772.14(142582.93,203030.37) | 437024.53(340261.27,547261.49) | 1.57 | 862.53(723.10,1025.68) | 794.04(618.11,991.54) | -1.08(-1.36,-0.80) |
| Bahamas | female | 1431.52(1239.31,1667.34) | 3243.32(2596.58,4025.03) | 1.27 | 1606.61(1393.73,1867.18) | 1490.70(1200.89,1840.47) | -0.58(-0.73,-0.42) |
| Gambia | female | 1053.49(811.92,1331.07) | 5430.35(4170.42,6840.55) | 4.15 | 620.18(481.80,779.10) | 1075.15(827.36,1350.66) | 1.87(1.67,2.07) |
| Timor-Leste | female | 1274.91(956.96,1742.73) | 4190.34(3233.95,5290.65) | 2.29 | 824.59(627.39,1064.52) | 1005.53(782.72,1257.49) | 0.51(0.30,0.72) |
| Togo | female | 4451.53(3556.42,5378.74) | 18057.84(13725.20,23304.29) | 3.06 | 656.38(525.52,787.97) | 858.36(658.60,1096.24) | 0.91(0.82,1.01) |
| Tonga | female | 894.71(743.22,1058.16) | 1313.24(1070.33,1598.00) | 0.47 | 2987.68(2484.02,3510.21) | 3104.91(2535.18,3779.35) | 0.17(0.11,0.24) |
| Trinidad and Tobago | female | 16117.28(14553.43,17758.57) | 27246.74(21660.08,33589.18) | 0.69 | 3645.84(3298.19,4011.62) | 2823.49(2239.95,3463.83) | -1.37(-1.56,-1.18) |
| Tunisia | female | 13286.56(10275.49,17638.36) | 55751.41(41694.76,73568.62) | 3.20 | 533.00(411.25,703.99) | 851.52(635.85,1121.66) | 1.65(1.61,1.69) |
| Turkey | female | 234626.83(189716.22,277537.15) | 407252.47(326494.67,505328.83) | 0.74 | 1253.44(1016.84,1487.14) | 873.17(700.61,1082.39) | -0.55(-1.00,-0.10) |
| Turkmenistan | female | 5419.72(4464.86,6544.43) | 19788.07(15427.48,24867.74) | 2.65 | 463.06(381.17,558.55) | 841.98(661.61,1056.13) | 1.83(1.60,2.05) |
| Uganda | female | 27234.57(21306.63,34407.84) | 75219.59(59348.19,94186.49) | 1.76 | 806.13(632.52,1021.64) | 917.70(732.76,1143.09) | 0.27(0.10,0.45) |
| Ukraine | female | 125797.33(97690.25,159848.35) | 139835.86(102934.81,182804.51) | 0.11 | 295.26(227.75,375.97) | 327.80(240.97,428.35) | -0.03(-0.23,0.17) |
| United Arab Emirates | female | 2615.90(2054.11,3243.66) | 18388.74(13988.03,24105.07) | 6.03 | 2032.82(1571.38,2527.70) | 1741.29(1374.73,2210.97) | -0.16(-1.07,0.76) |
| United Kingdom | female | 161251.71(130372.11,199033.12) | 287584.38(208178.57,382100.66) | 0.78 | 325.62(259.02,407.96) | 499.18(355.58,667.30) | 1.78(1.66,1.90) |
| United States of America | female | 984267.71(829202.30,1172460.80) | 1865432.56(1481665.80,2322602.02) | 0.90 | 570.54(477.42,681.12) | 668.28(524.55,834.77) | 0.81(0.68,0.95) |
| Uruguay | female | 8555.13(7779.84,9482.95) | 13309.57(11086.28,16071.85) | 0.56 | 385.27(349.73,427.82) | 442.23(364.99,537.45) | 0.51(0.21,0.82) |
| Uzbekistan | female | 24902.60(20612.64,29970.52) | 161203.01(132500.48,194549.82) | 5.47 | 369.65(305.56,444.52) | 1236.94(1027.68,1478.41) | 4.26(3.77,4.75) |
| Vanuatu | female | 493.29(355.88,749.21) | 2504.28(1850.44,3222.71) | 4.08 | 1518.80(1089.98,2343.51) | 2824.55(2107.41,3622.70) | 2.15(2.03,2.27) |
| Venezuela (Bolivarian Republic of) | female | 61986.98(53185.70,72221.00) | 208150.98(161235.47,258511.22) | 2.36 | 1176.00(1018.38,1369.12) | 1335.28(1036.63,1657.16) | 0.29(0.05,0.53) |
| Viet nam | female | 219015.92(169909.97,268221.86) | 528557.72(415720.91,653407.60) | 1.41 | 939.27(729.84,1146.73) | 1025.04(799.07,1265.22) | 0.37(0.16,0.58) |
| Virginia | female | 21051.22(17345.92,25391.13) | 49507.73(38056.70,62528.32) | 1.35 | 536.39(441.87,649.71) | 684.71(523.50,866.81) | 1.19(1.02,1.36) |
| Yemen | female | 13052.09(9460.22,18134.68) | 53220.45(38264.86,71500.95) | 3.08 | 494.80(357.21,695.47) | 736.40(535.37,978.65) | 1.53(1.44,1.61) |
| Zambia | female | 15364.54(12525.99,18814.45) | 34891.93(27851.80,44033.59) | 1.27 | 1040.53(854.74,1256.53) | 940.78(760.42,1174.05) | -0.61(-0.73,-0.50) |
| Zimbabwe | female | 23897.15(20026.68,28300.91) | 75011.64(57234.38,94821.43) | 2.14 | 1133.86(955.53,1344.54) | 1823.57(1404.03,2289.20) | 2.63(2.18,3.08) |
| Monaco | female | 66.67(48.64,87.66) | 134.15(93.66,183.41) | 1.01 | 179.89(129.90,238.59) | 306.11(212.37,422.40) | 1.85(1.79,1.91) |
| San Marino | female | 46.55(35.85,59.32) | 117.81(87.28,156.25) | 1.53 | 259.36(196.54,332.37) | 374.85(269.03,507.10) | 1.38(1.29,1.46) |
| Saint Kitts and Nevis | female | 450.10(389.98,515.58) | 584.23(469.77,708.05) | 0.30 | 2219.52(1925.31,2554.98) | 1694.62(1383.41,2039.11) | -0.80(-0.92,-0.67) |
| Cook Islands | female | 206.83(170.18,250.50) | 391.03(321.30,473.42) | 0.89 | 3353.21(2780.71,4045.41) | 3166.93(2591.95,3840.66) | -0.22(-0.28,-0.16) |
| Nauru | female | 66.56(52.05,87.23) | 123.51(89.02,177.20) | 0.86 | 3260.38(2600.42,4118.21) | 4609.72(3398.72,6374.26) | 1.09(0.84,1.34) |
| Niue | female | 31.43(25.05,39.46) | 42.82(32.73,54.37) | 0.36 | 2628.12(2086.15,3333.80) | 3760.54(2875.49,4776.39) | 1.08(0.90,1.27) |
| Palau | female | 123.84(96.40,157.17) | 394.63(296.91,494.68) | 2.19 | 2406.44(1876.52,3024.28) | 3564.08(2693.90,4451.43) | 1.39(1.20,1.57) |
| Tokelau | female | 20.79(16.11,26.31) | 25.29(19.25,32.45) | 0.22 | 2875.26(2240.01,3647.25) | 3783.19(2890.98,4834.75) | 0.87(0.69,1.05) |
| Tuvalu | female | 116.57(93.50,145.30) | 213.28(158.25,282.52) | 0.83 | 2867.46(2309.74,3550.57) | 3879.73(2891.07,5118.78) | 0.99(0.79,1.19) |

**Supplementary Table 6.** Age distribution of incidence rate for type 2 diabetes in different countries in 2019.

| 2019incidence rate | 15 to 19 | 20 to 24 | 25 to 29 | 30 to 34 | 35 to 39 | 40 to 44 | 45 to 49 | 50 to 54 | 55 to 59 | 60 to 64 | 65 to 69 | 70 to 74 | 75 to 79 | 80 plus | 80-84 | 85-89 | 90-94 | all ages |
| --- | --- | --- | --- | --- | --- | --- | --- | --- | --- | --- | --- | --- | --- | --- | --- | --- | --- | --- |
| Afghanistan | 103.9767278 | 259.5989066 | 323.1635252 | 342.7701942 | 421.7336439 | 679.4186583 | 1083.517612 | 1260.167193 | 1259.18331 | 1093.294752 | 819.1626705 | 578.4589241 | 357.6724681 | 151.4639251 | 237.4089268 | 111.9348874 | 40.86862217 | 252.1862821 |
| Albania | 23.90437984 | 81.0958383 | 130.1359492 | 157.2681007 | 181.2073169 | 233.1589529 | 312.2102537 | 394.8109574 | 478.8229454 | 505.3494854 | 481.1192667 | 388.3970618 | 232.9341304 | 89.12557899 | 121.747778 | 50.95183887 | 12.78004624 | 213.9587723 |
| Algeria | 71.30717727 | 177.4871279 | 234.893626 | 272.2992929 | 349.7198351 | 593.9908635 | 975.9981899 | 1220.91062 | 1342.151064 | 1216.824051 | 925.4977903 | 652.071602 | 384.0474753 | 157.7674473 | 199.257286 | 81.5321134 | 19.2152832 | 381.1722248 |
| American Samoa | 403.3874142 | 856.4122243 | 981.5915849 | 1152.940694 | 1479.603893 | 1834.148162 | 2134.299433 | 1828.223688 | 1261.570392 | 976.7834667 | 882.3699684 | 646.6583476 | 305.3934477 | 80.55630383 | 111.8517376 | 46.92010329 | 8.826551789 | 800.2167847 |
| Andorra | 72.58649999 | 129.074155 | 122.7584459 | 137.0339435 | 175.4911152 | 267.2150496 | 407.0709839 | 550.3334705 | 692.9683795 | 754.958256 | 745.360145 | 581.5091687 | 284.4334253 | 59.80975674 | 24.11455487 | 7.871596794 | 4.419241904 | 320.5680317 |
| Angola | 93.52179784 | 132.619621 | 146.6072036 | 177.3279236 | 226.186457 | 367.2551377 | 593.0849711 | 701.632296 | 713.1474453 | 731.4702878 | 953.4193293 | 707.2710727 | 427.6394909 | 174.5032538 | 289.0847052 | 121.4174636 | 28.43532118 | 405.8069218 |
| Antigua and Barbuda | 134.6156531 | 204.2555399 | 230.6565006 | 277.3979944 | 366.1759296 | 624.3389831 | 1020.188033 | 1266.834822 | 1381.41577 | 1324.740347 | 1156.892047 | 956.2383095 | 742.3371838 | 341.8765076 | 494.8670447 | 198.1160173 | 39.63076701 | 530.6305792 |
| Argentina | 51.47023018 | 104.9941335 | 132.6553951 | 179.2771835 | 249.1631402 | 384.6452807 | 577.4462739 | 768.3613204 | 952.1724286 | 938.420057 | 768.1137144 | 543.81739 | 268.2231781 | 70.72317094 | 252.0297977 | 111.6909481 | 33.52929228 | 289.2043337 |
| Armenia | 61.8948302 | 129.6793504 | 173.8906047 | 219.4561095 | 288.3820937 | 454.6025463 | 704.2700378 | 839.673982 | 877.5035905 | 756.2920451 | 506.0102288 | 327.33387 | 206.9470581 | 83.92468081 | 328.6569192 | 136.8019168 | 34.4305859 | 330.7096876 |
| Australia | 19.41789227 | 50.90111728 | 89.51035788 | 146.2619075 | 137.2343638 | 201.0854662 | 299.7769798 | 396.3201813 | 489.4063474 | 557.6126656 | 599.5249314 | 565.3862172 | 474.7070923 | 244.9920653 | 240.1557264 | 113.1488994 | 40.46746836 | 103.9920438 |
| Austria | 46.31367699 | 91.01737761 | 92.7256848 | 112.0548169 | 150.6359979 | 251.3859427 | 408.8865901 | 612.5035505 | 847.1058048 | 887.7555854 | 772.9418459 | 560.6526408 | 259.6221649 | 54.11904719 | 327.3454078 | 132.4923144 | 27.44300734 | 320.2128429 |
| Azerbaijan | 70.4338794 | 138.8738863 | 178.8978096 | 221.7735668 | 290.0039201 | 462.6043369 | 723.8615839 | 810.230953 | 751.0034199 | 624.1954977 | 439.1052571 | 298.8483544 | 197.955798 | 97.04203919 | 358.3500489 | 139.9651388 | 20.79891373 | 298.5969543 |
| Bahrain | 89.31302762 | 215.3562039 | 270.058687 | 305.1609547 | 401.0278552 | 978.3769591 | 1863.566894 | 3177.005824 | 4152.603919 | 2844.842108 | 1304.979608 | 616.5182018 | 282.7141885 | 79.00987382 | 156.4711016 | 63.28208685 | 11.85189381 | 996.4412897 |
| Bangladesh | 84.45353659 | 258.5681347 | 308.9522773 | 413.0643072 | 257.1260525 | 334.5538209 | 416.7335712 | 518.2270233 | 635.8436787 | 743.3738176 | 840.2072243 | 767.534961 | 562.6427196 | 271.865149 | 215.3296023 | 150.7906629 | 38.41085059 | 266.2266841 |
| Barbados | 134.4268766 | 197.1724593 | 223.2243717 | 266.9077751 | 346.279267 | 563.6346922 | 896.5751798 | 1086.227953 | 1152.457878 | 1181.914312 | 1187.666314 | 1049.889157 | 817.3933509 | 369.1698802 | 407.7144281 | 169.0112269 | 35.49326022 | 557.6402515 |
| Belarus | 14.41825597 | 79.67663458 | 144.0560904 | 160.8442629 | 168.4122838 | 206.4894611 | 273.3837445 | 310.3492562 | 319.4434867 | 303.9096499 | 264.394874 | 218.3156035 | 168.5995342 | 77.39082588 | 112.0262873 | 44.92987768 | 8.790142781 | 169.4937954 |
| Belgium | 70.91173041 | 126.9005742 | 120.9261682 | 136.0813987 | 225.6655869 | 283.5389564 | 344.9668836 | 404.6044759 | 461.4597878 | 464.1670852 | 419.9108929 | 353.2765074 | 266.1725556 | 119.8726733 | 221.4438566 | 90.64721479 | 20.46568933 | 246.7777849 |
| Belize | 115.844694 | 172.9096161 | 202.1228562 | 238.4662982 | 297.3358012 | 515.158302 | 866.4662594 | 976.9531625 | 896.4194374 | 848.7990426 | 825.7483786 | 761.1332799 | 666.3129692 | 320.2364152 | 232.2555157 | 109.5071602 | 38.71436262 | 289.628832 |
| Benin | 58.27520145 | 80.94658617 | 100.1111951 | 125.51924 | 158.1164843 | 252.2893093 | 402.4619096 | 587.3587587 | 798.6093629 | 941.3675527 | 811.6120109 | 500.6346953 | 185.4539824 | 14.16622505 | 24.94900705 | 7.837718865 | 4.730450045 | 427.3822983 |
| Bermuda | 86.41803946 | 127.2770127 | 145.4778132 | 172.6017957 | 218.6147849 | 338.4745885 | 524.9225566 | 645.5642888 | 708.36319 | 760.9444214 | 802.258912 | 774.5448746 | 696.1068537 | 334.3929734 | 347.9466873 | 146.1229577 | 37.8839809 | 402.7469378 |
| Bhutan | 81.36293879 | 123.0762222 | 169.1569814 | 233.7721919 | 310.3277111 | 419.2030501 | 554.890564 | 644.4986232 | 692.4742108 | 639.150054 | 499.6399696 | 445.0105897 | 468.2221245 | 264.0511385 | 365.1571128 | 140.7702604 | 23.08827118 | 231.6063474 |
| Bolivia (Plurinational State of) | 51.25740832 | 79.31117294 | 104.7095976 | 153.5748612 | 224.8634677 | 369.3437217 | 578.76894 | 747.7014534 | 876.6303209 | 922.9391189 | 900.0678971 | 756.4527111 | 518.3728202 | 232.7360323 | 160.7947572 | 63.165877 | 9.201279187 | 224.4439094 |
| Bosnia and Herzegovina | 33.64869466 | 130.7594438 | 219.9185085 | 269.8069779 | 324.5437554 | 506.0455331 | 797.7552594 | 1195.887481 | 1647.627985 | 1481.433376 | 913.9914412 | 524.7861647 | 253.9086169 | 70.64323252 | 92.99120296 | 37.93588852 | 7.230683258 | 571.7667306 |
| Botswana | 66.97283491 | 96.79906694 | 116.5386161 | 150.8406849 | 204.1027846 | 356.0336224 | 598.7372146 | 974.8850147 | 1423.932632 | 1443.723153 | 1167.24314 | 922.2625865 | 705.5784271 | 348.0664677 | 464.1273085 | 175.1725655 | 19.58890989 | 262.3069237 |
| Brazil | 67.31942608 | 368.0667141 | 414.3117983 | 398.9568674 | 438.234886 | 508.1173011 | 608.8251475 | 672.4789888 | 704.0425355 | 819.6472696 | 1004.541054 | 844.0704497 | 414.1350595 | 110.7294659 | 359.5371349 | 141.6853381 | 24.38694679 | 443.7661389 |
| Brunei Darussalam | 111.6101111 | 214.890122 | 326.2248778 | 485.1279475 | 673.2093047 | 936.925787 | 1254.486854 | 2226.086783 | 3296.385161 | 2380.643406 | 947.6741204 | 331.4798658 | 185.6724348 | 76.30948772 | 91.98795885 | 44.46870484 | 12.47139063 | 697.375804 |
| Bulgaria | 34.82451046 | 122.6947871 | 197.6320049 | 235.4804381 | 276.1819688 | 387.602512 | 562.8915763 | 708.3367161 | 824.5065487 | 791.6106073 | 641.3933036 | 465.1859105 | 258.4586445 | 87.19138491 | 121.3850735 | 49.19524176 | 10.13320549 | 370.1949098 |
| Burkina Faso | 53.32899998 | 78.6325557 | 103.3559531 | 130.6580864 | 160.5340783 | 240.9470512 | 368.9211292 | 522.8914105 | 696.9184727 | 828.4995835 | 921.1112398 | 817.7860331 | 579.9935428 | 262.9705687 | 358.9180853 | 138.9173815 | 21.45689276 | 120.298753 |
| Burundi | 58.14063022 | 79.80556839 | 90.29314045 | 108.1331075 | 133.7094319 | 201.4882526 | 310.2305747 | 418.3073313 | 523.8592046 | 611.6721409 | 681.8609647 | 642.8646037 | 521.188986 | 264.8195955 | 354.694555 | 136.6459635 | 22.57046955 | 98.6473828 |
| Cambodia | 61.71262796 | 120.5893525 | 161.6183807 | 210.6849584 | 277.7601081 | 391.1753718 | 545.5011145 | 697.3289703 | 842.7179937 | 1042.330633 | 1267.235533 | 797.3476902 | 659.6506061 | 317.187312 | 283.2664913 | 117.3193519 | 30.31662989 | 443.9755306 |
| Cameroon | 51.39412759 | 70.64153954 | 88.72626223 | 114.6874149 | 147.0542029 | 231.4434845 | 364.4366751 | 541.1167988 | 751.6642108 | 943.249314 | 1111.168893 | 987.8811171 | 656.7136086 | 292.9810893 | 392.4972286 | 152.2851906 | 20.75262132 | 129.4302862 |
| Canada | 21.36899678 | 22.66123262 | 56.92059508 | 124.0829034 | 191.9189972 | 268.2051273 | 351.9909863 | 497.3384365 | 694.2920773 | 739.0185006 | 655.4074473 | 477.0348483 | 212.8798567 | 39.86705289 | 64.38061047 | 23.12991032 | 5.994784833 | 270.8974337 |
| Cabo Verde | 62.86548185 | 92.70127063 | 121.2409928 | 158.4011305 | 203.8813596 | 315.7025402 | 488.9966702 | 713.226197 | 978.3904513 | 1121.486023 | 1133.12319 | 925.0531099 | 573.0327651 | 212.6196835 | 378.0188483 | 144.4020555 | 21.92342249 | 243.6978299 |
| Central African Republic | 107.6184194 | 414.6664182 | 460.1278715 | 447.6651134 | 485.9304257 | 565.931871 | 684.136044 | 753.4354108 | 777.1176256 | 890.7702464 | 1089.388065 | 916.3225138 | 478.9040973 | 135.5592634 | 214.670185 | 112.172543 | 21.17120824 | 479.5257841 |
| Chad | 52.57799502 | 74.56514858 | 94.35271507 | 120.1520484 | 151.9280455 | 240.3193463 | 379.7636649 | 541.0714489 | 720.1712698 | 855.7761864 | 959.7727859 | 848.017593 | 567.2550091 | 247.786671 | 26.55663292 | 9.417394394 | 3.916067874 | 103.7984509 |
| Chile | 46.35400172 | 101.373135 | 128.0824645 | 170.6858741 | 238.0099941 | 412.4293596 | 679.3929596 | 1027.447961 | 1419.167528 | 1294.993387 | 809.4762081 | 458.8402497 | 202.4537468 | 39.75894824 | 59.35952311 | 78.3619884 | 16.39338224 | 394.085845 |
| China | 88.59370454 | 191.2724718 | 210.593481 | 233.3449127 | 301.8351928 | 369.3438756 | 436.8227488 | 446.1050636 | 401.0969661 | 383.4774924 | 389.3265511 | 335.1042695 | 227.2681334 | 97.44731786 | 131.4199402 | 49.09579434 | 10.16601794 | 262.8832542 |
| Colombia | 126.9048046 | 113.3105336 | 146.7806636 | 205.2378579 | 289.0039265 | 433.1833521 | 630.1394929 | 778.5500068 | 879.0794999 | 884.2476258 | 770.5344956 | 552.9530804 | 398.9186488 | 166.6493322 | 255.3159375 | 152.5298991 | 30.74675846 | 358.4874047 |
| Comoros | 53.56052436 | 72.28427401 | 80.28129767 | 96.39016483 | 120.7889982 | 181.9572738 | 277.8176569 | 375.1237519 | 474.8816662 | 567.5618806 | 647.492156 | 634.2522834 | 550.6554798 | 283.5155093 | 390.3515755 | 149.7604518 | 22.26258917 | 132.6837796 |
| Congo | 94.35702792 | 137.156947 | 157.4870262 | 196.7181038 | 257.0239763 | 414.1251094 | 665.6162544 | 800.8508629 | 832.028264 | 840.4374845 | 820.6045173 | 689.9880521 | 473.6737667 | 219.9305538 | 371.0927857 | 142.3019431 | 22.97610019 | 209.9537031 |
| Costa Rica | 138.2754607 | 188.7898965 | 202.2635529 | 260.3429814 | 359.4689394 | 525.7961846 | 746.1230892 | 969.5814411 | 1184.777922 | 1116.953638 | 842.1826852 | 612.0793803 | 416.3818855 | 165.0091297 | 253.7053782 | 110.1964522 | 31.86610774 | 389.0154473 |
| C么te d'Ivoire | 55.84764491 | 78.75027459 | 98.89714596 | 126.0197216 | 160.8605666 | 250.4979484 | 391.0519466 | 575.9059525 | 794.0760237 | 953.7966512 | 1064.944425 | 927.4837526 | 609.6580472 | 263.6029507 | 356.2516259 | 149.7457833 | 33.7761802 | 138.9914766 |
| Croatia | 35.77139466 | 119.6353069 | 196.7164253 | 245.5105887 | 295.1690104 | 416.5869206 | 600.7062359 | 803.4839454 | 1013.964571 | 1031.083361 | 904.7560597 | 676.9627345 | 354.3441593 | 107.3775469 | 192.5858781 | 74.10819093 | 16.23380012 | 439.3064082 |
| Cuba | 135.5933979 | 201.0894413 | 226.7182299 | 263.6298742 | 330.1409138 | 507.8740251 | 777.7068979 | 898.4155381 | 898.2116077 | 893.7022763 | 883.7287178 | 791.2171222 | 642.4103859 | 285.3822281 | 348.0597476 | 146.5653716 | 37.23325569 | 472.9555935 |
| Cyprus | 63.94102913 | 117.5846615 | 112.850226 | 126.8361467 | 162.9498585 | 284.3853227 | 482.224314 | 974.238061 | 1664.220473 | 1712.91933 | 711.2259384 | 560.8456314 | 284.6733551 | 75.17813164 | 116.0732896 | 44.36703429 | 6.163055373 | 298.3621568 |
| Czechia | 49.34581729 | 170.7452107 | 287.8334134 | 360.8163152 | 432.7516133 | 640.0549139 | 957.1265999 | 1343.883388 | 1738.661754 | 1586.605256 | 1128.149054 | 731.5045144 | 353.6533766 | 86.3268924 | 299.4157038 | 125.8829376 | 29.8906117 | 633.503628 |
| Democratic Republic of the Congo | 90.0242095 | 127.0338376 | 142.8726499 | 172.2580575 | 218.7136749 | 361.0743065 | 588.6926076 | 710.9467821 | 745.1983542 | 762.6148398 | 756.5638313 | 639.9074989 | 455.646916 | 213.1629774 | 181.7732272 | 76.8593422 | 15.64799098 | 156.853212 |
| Denmark | 68.2202495 | 118.8629617 | 113.0061895 | 125.9518845 | 159.674271 | 245.9282954 | 380.6553664 | 497.8070674 | 595.9702371 | 653.2405741 | 675.1849335 | 547.2393829 | 288.3282784 | 81.10568822 | 125.502453 | 48.84581313 | 7.600085659 | 273.7539757 |
| Djibouti | 62.98849049 | 85.72978058 | 94.51679112 | 112.6990556 | 140.9166319 | 211.9724721 | 328.1586192 | 451.0246781 | 570.6465518 | 679.3809947 | 769.4268176 | 722.7249298 | 564.3218008 | 288.3456032 | 413.3027585 | 167.4106248 | 35.9939349 | 138.6477729 |
| Dominica | 170.5042893 | 284.9149418 | 322.6835826 | 378.7042068 | 501.4421818 | 815.2904676 | 1265.546116 | 1447.689209 | 1427.657701 | 1309.634252 | 1136.796989 | 912.2848702 | 678.6192358 | 310.8943676 | 322.6581185 | 123.9439724 | 17.91018843 | 587.8151625 |
| Dominican Republic | 90.20909975 | 132.3105203 | 149.8688942 | 180.6289637 | 235.8198813 | 335.4239769 | 475.1775437 | 552.5209775 | 573.7230149 | 580.5164243 | 575.0659663 | 564.2664514 | 551.9169042 | 297.1071879 | 390.6875085 | 159.2084435 | 31.50014028 | 213.9490687 |
| Ecuador | 63.47914909 | 95.17488034 | 121.0848708 | 166.6957902 | 232.4033363 | 375.5939539 | 587.6238284 | 818.7189707 | 1055.124332 | 1157.097729 | 1153.48109 | 960.1957904 | 627.6441656 | 261.9869609 | 24.36856799 | 7.136047408 | 5.47478704 | 283.7313113 |
| Egypt | 61.23315132 | 151.0832459 | 188.3588047 | 204.4356778 | 255.5335743 | 450.7148902 | 771.6454027 | 945.1919437 | 989.9819827 | 891.7602414 | 691.7739801 | 495.7037011 | 301.4738386 | 121.9116479 | 371.4610245 | 145.400468 | 25.89892767 | 254.0705093 |
| El Salvador | 131.0437142 | 188.5343605 | 216.6521746 | 292.3536539 | 410.3603695 | 607.8692772 | 869.1254195 | 1067.483114 | 1206.63839 | 1084.039479 | 769.839005 | 544.6812052 | 390.1617187 | 161.0434011 | 248.129406 | 109.8739196 | 33.80644249 | 350.8934206 |
| Equatorial Guinea | 93.35485608 | 134.8167977 | 152.1149564 | 183.6000365 | 234.6703819 | 395.2983271 | 642.04093 | 792.6780325 | 867.7568565 | 892.722007 | 871.3797157 | 723.6192296 | 494.1198532 | 221.8956713 | 295.7721498 | 121.6830824 | 29.23473884 | 160.9288363 |
| Eritrea | 67.34013805 | 95.6755177 | 108.7972718 | 130.6633514 | 163.2784269 | 241.5164155 | 361.8211299 | 470.6729021 | 573.5341366 | 643.7340267 | 668.2349325 | 608.7553905 | 500.864577 | 272.9716814 | 345.3598703 | 133.7390807 | 22.24975656 | 121.6649 |
| Estonia | 22.98109483 | 108.0338625 | 194.5260526 | 221.0400988 | 235.3144612 | 296.0814117 | 399.603312 | 451.9205703 | 458.8467856 | 429.5739813 | 367.1894954 | 299.4777715 | 227.7568905 | 99.61464345 | 148.1420043 | 58.15300192 | 10.13537633 | 235.654007 |
| Ethiopia | 45.82923311 | 62.56876376 | 72.061376 | 86.79496438 | 106.6986463 | 154.5099143 | 230.4942938 | 316.1823673 | 410.2945462 | 509.9425338 | 613.7343401 | 614.8963464 | 523.2048894 | 275.8520844 | 23.82294337 | 7.734988625 | 4.574942945 | 79.94318391 |
| Micronesia (Federated States of) | 240.636469 | 486.9094743 | 590.194287 | 703.7406657 | 874.4079257 | 1091.044478 | 1333.662183 | 1331.990626 | 1158.924852 | 1133.199708 | 1209.259886 | 954.447173 | 462.2197743 | 137.0138629 | 287.4063449 | 115.9125331 | 28.38740675 | 546.2794114 |
| Fiji | 255.296369 | 481.2853361 | 562.2481765 | 734.5904337 | 1012.109639 | 1463.784575 | 1983.91066 | 2166.290402 | 2129.711988 | 2083.145073 | 1959.155703 | 1284.773491 | 492.1799754 | 97.29710185 | 347.7012217 | 146.0454248 | 38.53458743 | 807.9821107 |
| Finland | 126.6067612 | 216.4580414 | 202.6295347 | 216.0279239 | 268.5760985 | 391.1878086 | 575.4513266 | 724.8120963 | 840.0202626 | 888.4652383 | 883.7533096 | 696.7663396 | 361.04122 | 93.44605591 | 152.2863931 | 53.08668836 | 5.944163987 | 400.9916604 |
| France | 37.71664125 | 73.19877486 | 71.4358623 | 83.61903912 | 109.9101596 | 168.9351987 | 259.1003929 | 361.7539589 | 474.0768247 | 528.9498247 | 534.1469274 | 424.4057745 | 209.9128622 | 47.63299634 | 80.72405174 | 30.57721543 | 3.963956391 | 198.5658582 |
| Gabon | 97.7708223 | 141.6341686 | 161.6660719 | 198.9247952 | 254.694993 | 381.3161596 | 554.9174185 | 715.0398173 | 858.244607 | 889.7052066 | 833.6120387 | 721.2544881 | 569.0106341 | 261.0059454 | 134.5960214 | 51.8279855 | 10.56337553 | 278.6863164 |
| Georgia | 79.20036279 | 168.4744643 | 227.2334644 | 280.9901165 | 360.4600803 | 586.6623252 | 929.322881 | 995.5218005 | 864.7438719 | 684.0279977 | 449.2279671 | 284.6082061 | 183.4910992 | 67.19899288 | 205.5084547 | 84.73186939 | 18.07313611 | 378.9843613 |
| Germany | 108.2716843 | 186.5632193 | 187.398913 | 215.0177295 | 277.9700051 | 434.0056907 | 669.8972304 | 925.2374236 | 1180.743659 | 1235.895965 | 1145.735637 | 855.1326438 | 411.2451562 | 109.3805891 | 23.74173221 | 8.110000351 | 3.954426258 | 508.7556448 |
| Ghana | 60.31164277 | 85.94424887 | 110.4789748 | 142.3179596 | 180.1304448 | 281.0318286 | 440.1686025 | 640.5739977 | 870.3540447 | 1027.670231 | 1116.724675 | 943.0141002 | 598.09412 | 255.0929147 | 291.8599485 | 119.3295005 | 28.30076785 | 175.4069147 |
| Greece | 82.33744341 | 145.8636022 | 130.6161861 | 138.2423735 | 175.3355837 | 301.3083315 | 507.6818504 | 669.8938742 | 792.371067 | 778.1667211 | 652.2867423 | 464.2092371 | 216.4793333 | 46.68809728 | 73.0068336 | 26.41676624 | 3.407886707 | 324.2655688 |
| Greenland | 26.61752186 | 26.28063902 | 64.14095825 | 140.4789328 | 223.3570376 | 327.6022763 | 438.5419331 | 586.6979509 | 771.3544983 | 770.0538381 | 602.4061968 | 409.339488 | 172.4375552 | 35.28481965 | 365.4315224 | 152.9349693 | 37.32317773 | 270.9320224 |
| Grenada | 146.9493545 | 227.7280879 | 267.9011841 | 343.0129311 | 469.4759441 | 748.1987628 | 1144.439053 | 1364.118693 | 1435.725096 | 1419.152186 | 1337.71816 | 1080.020164 | 741.5914666 | 323.3415771 | 196.6119639 | 83.78288924 | 19.10673636 | 559.205716 |
| Guam | 222.9123798 | 384.8781278 | 376.3601698 | 398.1650567 | 475.6866645 | 583.4729838 | 712.7323764 | 728.4910095 | 656.6291147 | 668.9305023 | 751.2048703 | 647.8840525 | 374.7977188 | 120.3745619 | 190.3059158 | 76.67101126 | 15.63669237 | 370.2503598 |
| Guatemala | 150.7540371 | 213.1963493 | 244.5463792 | 340.4177043 | 489.0690438 | 749.6403828 | 1094.846412 | 1309.152801 | 1408.635293 | 1255.562687 | 935.9937139 | 666.0567409 | 427.8254092 | 198.2931978 | 247.1364518 | 110.1639595 | 32.90664968 | 342.683139 |
| Guinea | 48.88844581 | 68.08930714 | 85.30835599 | 108.6817108 | 138.2950343 | 218.2652564 | 344.4514028 | 502.0403317 | 684.57296 | 834.6818027 | 962.1891822 | 869.9565404 | 606.5087146 | 257.6409833 | 24.48397456 | 8.175612068 | 4.194896008 | 116.4923253 |
| Guinea-Bissau | 60.98733614 | 90.26576329 | 112.2746049 | 138.4323561 | 174.0808279 | 263.7232644 | 403.3972388 | 572.4251852 | 762.7907285 | 883.7764456 | 942.8803013 | 813.3157947 | 561.3819551 | 262.5384255 | 338.2682571 | 135.9164287 | 27.45133602 | 130.666771 |
| Guyana | 178.7437505 | 280.2955573 | 318.8751705 | 398.1869555 | 545.4835377 | 959.3675865 | 1560.650625 | 1679.771544 | 1466.421189 | 1260.835316 | 1073.109845 | 868.2312387 | 657.4447996 | 311.1322698 | 435.0969738 | 178.4172764 | 37.91723619 | 528.5847147 |
| Haiti | 159.0544855 | 246.0647475 | 276.5000907 | 335.5623969 | 446.4356678 | 707.5477963 | 1085.105665 | 1233.150955 | 1200.858103 | 1103.616514 | 1088.28637 | 745.0580837 | 419.6960894 | 159.8928756 | 208.1027711 | 90.60956429 | 19.46136525 | 325.5060735 |
| Honduras | 163.8721522 | 229.5996184 | 246.58557 | 316.8361079 | 438.4218002 | 644.1077147 | 915.7073698 | 1097.540937 | 1199.359919 | 1054.577829 | 731.8167372 | 507.3545777 | 360.9146942 | 173.8695623 | 227.5012272 | 100.0440333 | 31.23698442 | 308.9140944 |
| Hungary | 37.68331036 | 124.3010227 | 208.5846537 | 264.5347504 | 316.5289251 | 460.8159771 | 683.1389008 | 849.3424304 | 965.355249 | 934.5401358 | 791.2397438 | 588.6613244 | 330.9709956 | 106.7893363 | 64.65376587 | 18.5259765 | 6.85095501 | 431.3521495 |
| Iceland | 81.86642931 | 139.5746504 | 136.0144917 | 151.4429488 | 189.5470594 | 280.9822778 | 421.0426739 | 561.63308 | 701.6729854 | 754.4212296 | 736.3834829 | 575.0147946 | 291.9470026 | 73.46171397 | 24.02805173 | 8.377439748 | 3.869030192 | 281.9846282 |
| India | 101.119044 | 157.3784565 | 221.2636027 | 318.5642841 | 438.3669338 | 600.7182052 | 795.4793566 | 856.5550994 | 808.3678502 | 683.8738036 | 500.5160874 | 411.9086592 | 406.9896676 | 237.094928 | 314.3263809 | 127.6617555 | 27.75873911 | 302.4778187 |
| Indonesia | 50.86315267 | 96.16016169 | 143.2276825 | 208.2230223 | 290.1175434 | 369.29811 | 444.9044011 | 531.8275301 | 628.0578834 | 708.4684052 | 773.2470729 | 704.3996781 | 527.5917941 | 263.9956106 | 111.4702514 | 48.40181386 | 13.87582879 | 243.4621945 |
| Iran (Islamic Republic of) | 57.95740528 | 129.258331 | 182.6690928 | 234.1032577 | 304.7460872 | 514.0891231 | 841.9816256 | 1045.281684 | 1136.424939 | 1009.714035 | 737.9371273 | 507.9388355 | 299.2187964 | 109.4141813 | 98.9572296 | 39.52473709 | 7.676165787 | 345.7766077 |
| Iraq | 102.856267 | 237.3295685 | 294.838864 | 318.4481786 | 386.164473 | 672.525366 | 1135.289111 | 1366.983482 | 1411.855483 | 1220.291842 | 875.4233521 | 597.0159343 | 361.195248 | 140.1898891 | 342.0282828 | 144.2561426 | 37.53640528 | 344.3155096 |
| Ireland | 59.42472001 | 109.2514467 | 112.0278288 | 133.8543952 | 177.3370889 | 271.8168971 | 412.9589158 | 563.8715272 | 718.1012849 | 816.1587301 | 864.8468627 | 696.6175337 | 349.7676209 | 92.59223303 | 341.8222864 | 133.2415105 | 20.54099259 | 288.1604601 |
| Israel | 67.83207827 | 125.3671695 | 113.4779437 | 123.6804214 | 159.2963326 | 291.6408669 | 511.7695757 | 747.7284294 | 988.5901195 | 1043.501281 | 950.0628529 | 695.2206077 | 304.4980872 | 56.91271918 | 85.41656326 | 33.97052518 | 6.311969997 | 277.3703614 |
| Italy | 60.34863759 | 108.1167942 | 131.4570183 | 163.9780955 | 209.6904853 | 354.8605053 | 584.2982339 | 860.0293899 | 1159.688812 | 1162.979817 | 953.1985199 | 665.1707809 | 307.9499301 | 66.77607811 | 343.1407687 | 144.7516687 | 37.91221813 | 449.4124022 |
| Jamaica | 130.3550467 | 190.8770037 | 218.4628572 | 281.1400707 | 392.7722505 | 658.8241771 | 1049.802729 | 1282.136751 | 1376.077749 | 1379.910341 | 1325.197856 | 1107.541345 | 785.4572133 | 294.5667189 | 497.7639221 | 201.8620776 | 40.03561179 | 477.3183111 |
| Japan | 56.79316669 | 91.97129073 | 127.5546409 | 171.2207876 | 222.298738 | 277.420425 | 335.7115715 | 394.7187889 | 454.1591967 | 460.356053 | 420.5365899 | 354.616289 | 265.8252876 | 111.456291 | 175.8038383 | 78.60167151 | 25.23207265 | 241.671157 |
| Jordan | 81.53742002 | 190.1086968 | 231.2768073 | 241.2281098 | 291.8914969 | 561.6464869 | 1011.421823 | 1300.983427 | 1453.75272 | 1311.209602 | 972.3510845 | 670.0428273 | 379.5884822 | 146.2867555 | 102.8364111 | 41.31110936 | 7.719548978 | 330.1702554 |
| Kazakhstan | 77.58377111 | 164.424714 | 216.9169954 | 259.5116965 | 327.0484788 | 490.6397633 | 734.6188337 | 879.5416544 | 941.6462658 | 803.8172759 | 498.1163568 | 296.2834741 | 189.9172434 | 81.7054049 | 24.86236049 | 8.634716014 | 3.843236158 | 311.3741691 |
| Kenya | 51.55666607 | 70.67540824 | 83.87753038 | 104.1975616 | 213.670598 | 287.7295932 | 366.9452005 | 436.0615916 | 494.6101188 | 597.3190143 | 736.5395875 | 724.1179672 | 588.633568 | 241.8489782 | 24.67508148 | 8.734117243 | 3.763544402 | 266.0505637 |
| Kiribati | 287.9900696 | 619.3328431 | 704.860275 | 795.98428 | 1018.322081 | 1185.034181 | 1306.087168 | 1266.163365 | 1110.037975 | 1074.521295 | 1096.629045 | 857.4634765 | 432.0531331 | 141.4893965 | 240.0758215 | 112.5644234 | 38.95138624 | 540.1041602 |
| Kuwait | 114.4747131 | 282.5338931 | 350.286931 | 367.4061349 | 449.2074339 | 699.7586731 | 1042.633145 | 1282.475701 | 1428.259129 | 1315.17714 | 1032.257273 | 746.9588859 | 447.1787357 | 174.4567222 | 229.0677734 | 107.7148468 | 38.47193106 | 468.6857923 |
| Kyrgyzstan | 54.80654786 | 110.7066952 | 142.61142 | 166.5113574 | 203.2753729 | 264.8652653 | 348.9152982 | 386.9393222 | 383.7617638 | 347.2708782 | 277.8191263 | 216.6098701 | 164.0039751 | 75.36408306 | 201.2587896 | 83.8052048 | 20.54275677 | 140.9167011 |
| Lao People's Democratic Republic | 75.57815854 | 145.8435755 | 193.8398917 | 248.3240218 | 320.9432817 | 441.0445786 | 602.7249013 | 768.1702028 | 929.2938926 | 1162.142127 | 1436.804337 | 1231.206913 | 690.7261492 | 252.9349446 | 302.9658518 | 124.6653863 | 28.06571714 | 272.7208227 |
| Latvia | 16.3082696 | 102.3340754 | 195.4381099 | 220.0498908 | 224.3418187 | 299.8724217 | 441.8248637 | 503.5590879 | 495.3715101 | 443.5072194 | 348.4211577 | 256.1207242 | 169.6436664 | 67.12492565 | 98.10852228 | 38.99441633 | 7.131243774 | 242.3457289 |
| Lebanon | 76.30565756 | 185.9799622 | 240.5565176 | 268.6328736 | 333.6786691 | 583.0824298 | 984.6716902 | 1220.413274 | 1314.411981 | 1183.444048 | 914.3112783 | 649.900312 | 383.6945156 | 142.4119683 | 105.4165467 | 41.75594916 | 7.761520919 | 390.8886384 |
| Lesotho | 65.76838964 | 102.6916868 | 129.6413087 | 166.2181522 | 219.618747 | 358.9337501 | 577.4077398 | 850.6771702 | 1147.610618 | 1149.226503 | 931.4418367 | 770.5473176 | 656.4626175 | 352.9389386 | 451.6224437 | 175.957256 | 26.0585012 | 229.6250217 |
| Liberia | 66.46806853 | 96.18821076 | 120.3485447 | 148.8520902 | 184.5982971 | 292.7257419 | 465.9009553 | 680.9967228 | 927.3391181 | 1081.065911 | 1149.164639 | 951.393764 | 587.4459388 | 227.0409255 | 360.6253247 | 140.8137847 | 25.89703315 | 165.2320141 |
| Libya | 81.76959343 | 218.2036282 | 293.1211437 | 337.2584998 | 429.9953085 | 768.7824638 | 1296.007373 | 1537.390884 | 1555.070311 | 1290.027982 | 855.3687611 | 544.073043 | 316.9344918 | 106.9483074 | 108.7878748 | 44.24439801 | 9.385242635 | 490.0888781 |
| Lithuania | 14.18902519 | 84.94871872 | 161.7340127 | 185.3476214 | 193.3088383 | 239.6523761 | 322.9673285 | 362.8536752 | 363.0409298 | 329.9118547 | 265.1075846 | 206.0263323 | 153.8565875 | 67.93670645 | 379.9796637 | 155.758513 | 31.74474235 | 189.2065005 |
| Luxembourg | 70.49236573 | 130.9642439 | 135.6882563 | 165.3651097 | 221.0434219 | 405.9720715 | 705.0244276 | 1062.647668 | 1431.334103 | 1394.936763 | 1092.910009 | 735.3976894 | 323.920627 | 60.36963724 | 207.9206991 | 86.27963617 | 18.95918819 | 474.2536364 |
| North Macedonia | 33.8407563 | 122.9970103 | 209.5069008 | 270.9556952 | 222.7999615 | 276.9108168 | 333.1081704 | 389.4593788 | 448.438705 | 456.7503785 | 420.4989667 | 355.1396162 | 263.900479 | 102.7412731 | 341.246021 | 141.9099884 | 32.34405871 | 237.3321934 |
| Madagascar | 59.49561111 | 81.64020523 | 92.00340814 | 110.3944454 | 583.4678352 | 737.5569373 | 824.5549728 | 1096.706256 | 1490.159977 | 1354.087192 | 869.2483851 | 493.4184115 | 183.6003776 | 15.71229565 | 346.6673788 | 139.8264064 | 29.0579999 | 503.8403403 |
| Malawi | 76.90734044 | 105.5249007 | 114.9947481 | 132.5892669 | 160.1686449 | 247.17556 | 392.3715698 | 523.623221 | 648.671901 | 748.1615146 | 812.9725953 | 725.7602038 | 539.1378377 | 247.9945419 | 342.7954275 | 132.5361051 | 18.05416991 | 122.7365021 |
| Malaysia | 82.24548234 | 158.8263763 | 199.3805738 | 240.6966076 | 303.2234044 | 429.1166141 | 610.7816629 | 773.9767366 | 915.7792265 | 1120.958234 | 1360.635538 | 1161.665905 | 655.2686581 | 235.6289213 | 458.5101886 | 178.7976836 | 27.06340567 | 332.8233223 |
| Maldives | 61.12538549 | 113.7995709 | 142.604302 | 169.9871334 | 208.8919248 | 293.2850556 | 418.8746671 | 570.8026207 | 743.7474402 | 1000.914811 | 1308.940907 | 1151.641467 | 682.9869486 | 255.4735524 | 110.7371115 | 45.3996869 | 10.45241926 | 231.5462966 |
| Mali | 49.44851558 | 70.11589995 | 87.75584678 | 110.9418134 | 139.9521154 | 221.250116 | 349.7585805 | 501.3097021 | 669.7024192 | 799.0492838 | 897.1387134 | 806.6493909 | 564.6742413 | 249.7099836 | 240.3025074 | 112.3890083 | 39.32469343 | 107.6554734 |
| Malta | 93.49975868 | 157.3895035 | 146.4409158 | 171.6901942 | 229.8328072 | 381.9145618 | 616.8511136 | 883.8288275 | 1162.100702 | 1213.666148 | 1098.020951 | 801.855531 | 362.3148302 | 75.04447352 | 28.35453449 | 9.678824128 | 4.669753118 | 469.4994623 |
| Marshall Islands | 407.5324954 | 908.7219543 | 1084.243156 | 1229.199372 | 1497.875346 | 1707.285333 | 1843.464511 | 1583.141355 | 1139.005706 | 920.1765065 | 863.0123534 | 652.3819881 | 317.0740149 | 95.36118201 | 122.105321 | 51.15133849 | 10.07776987 | 775.8231917 |
| Mauritania | 33.42765391 | 46.01533433 | 58.67855043 | 76.8607302 | 98.08593707 | 160.3988914 | 261.1263864 | 385.7057416 | 529.1062759 | 655.4233885 | 772.1540266 | 740.1724592 | 581.0940917 | 273.5203184 | 385.7570894 | 148.3852124 | 21.57778975 | 103.7063652 |
| Mauritius | 107.1343036 | 234.1130162 | 325.2577429 | 435.7008845 | 596.0297889 | 814.2512474 | 1070.209015 | 1526.279718 | 2075.973489 | 2215.033624 | 2097.184831 | 1495.000836 | 677.8954765 | 166.1868806 | 281.744018 | 113.1846673 | 27.83337152 | 811.210416 |
| Mexico | 157.6703272 | 236.2438853 | 281.6001684 | 388.1513402 | 555.9650653 | 835.6122466 | 1193.183318 | 1405.855374 | 1498.270451 | 1337.818874 | 1023.047736 | 728.7712543 | 437.8006731 | 171.1310535 | 238.6433888 | 111.8258447 | 39.45390737 | 496.3925317 |
| Republic of Moldova | 21.51854467 | 115.2331248 | 213.4240179 | 244.4932888 | 262.4854958 | 347.7386437 | 494.3608605 | 547.3757477 | 519.702872 | 448.3555409 | 335.8181378 | 244.8155554 | 175.027737 | 75.72081418 | 236.5749813 | 111.2077932 | 40.17357882 | 261.1042953 |
| Mongolia | 38.15699903 | 76.2126875 | 99.19285957 | 116.1359842 | 137.8644917 | 175.8637307 | 229.0277049 | 251.3605274 | 246.0694067 | 235.9149393 | 219.4233075 | 193.3808959 | 159.3108068 | 88.02757546 | 109.6691203 | 45.45533146 | 11.26353542 | 104.08377 |
| Montenegro | 40.49237431 | 136.791134 | 226.4405316 | 280.9059893 | 594.2633039 | 789.5456506 | 938.197438 | 1285.731933 | 1743.00979 | 1526.902619 | 899.2503083 | 471.6188117 | 174.5591747 | 14.65411996 | 242.8482608 | 113.2114043 | 39.98662522 | 550.532254 |
| Morocco | 73.10992135 | 177.13889 | 230.4834109 | 262.3330433 | 331.3097319 | 542.4224671 | 873.2595747 | 1061.529373 | 1126.739236 | 1022.520162 | 795.0601552 | 576.3509317 | 361.8761053 | 147.035738 | 284.4544756 | 113.1969332 | 20.60659679 | 359.658354 |
| Mozambique | 79.29754262 | 106.8003348 | 116.4363597 | 136.2253923 | 165.7682232 | 245.579045 | 371.5001992 | 491.4341298 | 604.8535758 | 703.7585433 | 779.9444668 | 710.4660607 | 554.4719901 | 273.5111276 | 196.3225064 | 81.29182667 | 17.40799047 | 114.2838887 |
| Myanmar | 69.14808781 | 136.6534647 | 201.0376398 | 266.6508839 | 331.7059894 | 428.7283157 | 553.3241132 | 691.0274918 | 837.711559 | 1163.825839 | 1605.506153 | 1403.032844 | 767.8846788 | 261.3938225 | 116.4154048 | 41.28169999 | 5.456207509 | 330.5289749 |
| Namibia | 59.62837948 | 85.33891265 | 100.829093 | 125.3482129 | 163.1770948 | 286.3595271 | 488.4228787 | 757.6894905 | 1067.103423 | 1083.7737 | 878.4451178 | 533.9052561 | 422.8939959 | 210.5668241 | 133.4495909 | 49.12033718 | 6.074923652 | 82.57145869 |
| Nepal | 102.6344951 | 145.4511097 | 203.9375738 | 269.0691462 | 331.3380162 | 428.9943797 | 556.0026746 | 620.2018393 | 626.1806923 | 572.5300351 | 467.7296461 | 438.6142424 | 480.6495245 | 285.6048017 | 381.7781471 | 147.1766023 | 22.75924858 | 223.3909065 |
| Netherlands | 58.81728301 | 107.0070183 | 103.0151887 | 116.1465589 | 148.779458 | 225.2930652 | 342.7413681 | 463.458884 | 583.0345151 | 646.8050673 | 663.3637922 | 530.4711614 | 265.9912851 | 68.08619195 | 105.887068 | 121.5012001 | 27.60889909 | 267.1115325 |
| New Zealand | 32.7088815 | 56.70328716 | 95.60019181 | 146.4671973 | 197.3392642 | 257.6379213 | 320.0118636 | 355.2495105 | 370.3395838 | 440.6568089 | 558.4633191 | 659.5298562 | 755.2487015 | 373.0938366 | 25.99288554 | 8.432182416 | 4.438165037 | 240.6171477 |
| Nicaragua | 141.2569032 | 195.9593074 | 216.1356787 | 296.4578471 | 431.2648269 | 651.20489 | 935.5459897 | 1126.249898 | 1236.792252 | 1081.78425 | 735.3465424 | 503.9655909 | 365.6370352 | 177.0003471 | 236.1523589 | 105.1932897 | 32.75460544 | 333.0969595 |
| Niger | 33.01011622 | 47.53229207 | 62.1647241 | 81.38578208 | 103.5218484 | 161.8495826 | 255.3096677 | 365.5006689 | 486.8699451 | 597.7462793 | 696.2343871 | 670.6685079 | 537.296941 | 273.4838532 | 363.8342102 | 141.9548651 | 23.9509173 | 71.01833217 |
| Nigeria | 31.24070576 | 44.13239505 | 58.96619144 | 79.87569941 | 103.1461094 | 162.7504776 | 257.132259 | 373.8438098 | 508.5048114 | 624.2753175 | 733.2692156 | 719.5002583 | 567.258175 | 274.0824399 | 380.7902939 | 150.7664633 | 27.85420987 | 87.21445846 |
| Democratic People's Republic of Korea | 105.2506002 | 180.716606 | 152.9497262 | 180.6245808 | 261.0325255 | 341.6569095 | 425.2733733 | 464.5060412 | 461.9918825 | 460.0580978 | 456.1068588 | 390.0458177 | 270.2091379 | 122.1391117 | 160.7291501 | 61.60437911 | 12.09480838 | 242.2193166 |
| Northern Mariana Islands | 229.9366173 | 416.7071924 | 434.7054385 | 511.3748947 | 672.2190053 | 842.7301992 | 1013.506084 | 1071.883671 | 974.1366049 | 979.0168319 | 1079.558921 | 861.0957233 | 442.5389783 | 130.2170999 | 183.4754008 | 125.8156835 | 26.79396946 | 611.248179 |
| Norway | 82.29697259 | 132.6102607 | 153.511422 | 185.9180151 | 233.4360879 | 322.2519157 | 449.6250401 | 590.8929946 | 740.5783176 | 825.8333469 | 1337.605154 | 878.4390562 | 345.3331054 | 50.82337584 | 68.43401618 | 23.42679353 | 4.372106287 | 454.5162719 |
| Oman | 67.31743086 | 155.6158329 | 196.915731 | 219.6943597 | 274.8682079 | 538.9957941 | 978.8617203 | 1376.775315 | 1684.521552 | 1510.757632 | 794.1762492 | 720.8454232 | 538.1854779 | 272.890567 | 349.8940261 | 146.9927414 | 38.06735254 | 229.8915181 |
| Pakistan | 110.9949202 | 162.7501202 | 213.4279327 | 299.7114385 | 415.4172805 | 537.5452136 | 665.1237212 | 749.5316898 | 796.2888821 | 752.4981634 | 633.1922628 | 555.1200648 | 518.6270254 | 290.7127652 | 386.5684991 | 155.4877412 | 30.46313947 | 220.89271 |
| Palestine | 63.71881038 | 155.4060122 | 203.8917602 | 238.5841609 | 309.3719985 | 604.9682328 | 1086.295579 | 1541.658832 | 1918.307816 | 1707.078752 | 1177.160531 | 766.1089459 | 418.4643142 | 142.6987209 | 176.1427609 | 78.67077226 | 25.19899925 | 313.0779468 |
| Panama | 131.7130776 | 180.2893165 | 195.5460414 | 255.3501908 | 424.2525116 | 577.1851731 | 709.8013265 | 968.5321934 | 1310.901539 | 1231.057488 | 849.4582325 | 507.2253252 | 187.07377 | 13.84213609 | 284.5640218 | 115.8557468 | 27.98916222 | 467.5020467 |
| Papua New Guinea | 253.4614589 | 506.2898045 | 576.4047522 | 627.4642667 | 727.3695341 | 819.4311505 | 903.5721438 | 895.4928909 | 817.6610038 | 823.114326 | 901.5545533 | 744.8027069 | 410.5538128 | 144.8385134 | 329.9856568 | 139.3682134 | 36.55257838 | 391.4327338 |
| Paraguay | 53.26855833 | 138.9306575 | 189.4784183 | 265.3250572 | 420.4861037 | 487.0558967 | 581.0638698 | 646.1893276 | 685.7348685 | 803.3146569 | 984.6660209 | 826.1008117 | 405.5544801 | 107.3861528 | 158.2669103 | 63.97731918 | 12.33861486 | 424.1662423 |
| Peru | 44.07449122 | 66.65965762 | 85.42717531 | 116.7585906 | 160.9356577 | 250.769652 | 382.9041712 | 501.9519411 | 606.8312677 | 666.3705795 | 686.4048399 | 617.6292475 | 473.7943677 | 203.7943733 | 315.7173289 | 125.200182 | 22.92241108 | 190.4148397 |
| Philippines | 52.92515863 | 89.53568699 | 122.1837396 | 166.0874513 | 223.592949 | 316.8105362 | 442.1913712 | 570.71332 | 699.5326727 | 857.8089971 | 1034.657094 | 931.9473262 | 608.306308 | 259.7770785 | 353.1484332 | 146.5808506 | 36.1071438 | 209.5774662 |
| Poland | 40.2338608 | 129.0580154 | 235.4343667 | 310.6340087 | 367.374382 | 461.5944262 | 588.1436914 | 731.172571 | 884.3328239 | 843.6253478 | 650.0114871 | 452.1041241 | 246.6830246 | 75.80935753 | 198.8257496 | 81.82679105 | 17.78217777 | 386.2965982 |
| Portugal | 101.1027097 | 171.3747679 | 155.5700185 | 173.345895 | 225.0045559 | 390.0389688 | 654.4285367 | 968.4303922 | 1302.245182 | 1323.955644 | 1127.17316 | 792.4391128 | 341.7682786 | 61.63505893 | 96.5249499 | 34.34724988 | 4.407603315 | 505.2859599 |
| Puerto Rico | 158.8321839 | 239.3551228 | 271.3178296 | 335.4154597 | 454.6477303 | 750.9097509 | 1181.546386 | 1362.19123 | 1352.845477 | 1250.95506 | 1091.5975 | 894.9657051 | 681.8610248 | 278.2278589 | 448.0531299 | 175.9565935 | 30.01035464 | 634.3988054 |
| Qatar | 79.7697093 | 214.0742411 | 298.8072575 | 364.1778346 | 486.5859839 | 1499.999543 | 3002.377216 | 3671.218466 | 3547.927017 | 2193.801856 | 898.7210408 | 363.141462 | 184.8386402 | 62.02981725 | 72.23212299 | 29.34316618 | 4.750053098 | 835.0190917 |
| Romania | 24.46491445 | 97.81278898 | 162.8037431 | 195.4656004 | 228.4800331 | 313.3198769 | 445.3474576 | 547.0988112 | 620.0711098 | 588.7956607 | 468.0204384 | 333.9939966 | 184.5937176 | 59.85874308 | 24.76842223 | 8.448246758 | 3.982592492 | 274.2430905 |
| Russian Federation | 23.8808527 | 67.43837687 | 115.2272716 | 144.9459476 | 165.7909262 | 212.2909243 | 283.4559069 | 352.8835505 | 419.9432434 | 414.5189959 | 341.846503 | 289.8872621 | 258.0384951 | 133.8292212 | 179.4085018 | 74.24098712 | 14.86029565 | 191.2627556 |
| Rwanda | 51.54774632 | 70.61040662 | 81.24969979 | 98.17856016 | 120.9505788 | 184.6177799 | 284.0515817 | 394.4871715 | 512.4843209 | 615.0900317 | 698.0459453 | 655.6504802 | 542.4813412 | 278.9684512 | 107.5255597 | 37.56472621 | 4.920675407 | 110.3202362 |
| Saint Lucia | 179.5469802 | 288.6350129 | 323.5759827 | 394.0931912 | 544.2045594 | 882.1651712 | 1356.277411 | 1636.071898 | 1755.939557 | 1573.496888 | 1238.007737 | 940.6431491 | 658.1991332 | 283.8805993 | 411.9670117 | 165.1020246 | 32.47373402 | 682.8126512 |
| Saint Vincent and the Grenadines | 166.3461076 | 267.635249 | 307.6545961 | 367.4083486 | 486.3251816 | 836.7425404 | 1361.826402 | 1525.961435 | 1419.967047 | 1303.082185 | 1195.875307 | 991.7388672 | 723.3383911 | 342.6952893 | 467.2476804 | 191.6880097 | 38.66960779 | 597.1927834 |
| Samoa | 229.8650624 | 442.6718332 | 503.4841779 | 603.089938 | 779.674105 | 977.4102203 | 1178.9506 | 1178.116736 | 1035.582376 | 999.7025727 | 1045.600044 | 834.8249712 | 424.5845688 | 128.1149509 | 325.923053 | 126.7734849 | 22.85751323 | 442.4769371 |
| Sao Tome and Principe | 55.61614707 | 79.88206646 | 101.2265234 | 127.5459329 | 159.50818 | 260.4112988 | 425.2140279 | 594.9807257 | 763.2624686 | 881.1786584 | 950.6478897 | 811.2299621 | 532.2450164 | 225.2681953 | 309.6798627 | 118.5763488 | 17.11425244 | 158.0239783 |
| Saudi Arabia | 108.8218002 | 255.3611396 | 322.4641714 | 360.3807433 | 452.6547766 | 738.3457902 | 1172.676305 | 1405.160673 | 1475.151736 | 1327.813902 | 1049.6746 | 755.9848072 | 450.8291206 | 163.0113229 | 240.7574947 | 95.07369164 | 13.92876241 | 473.5268677 |
| Senegal | 73.58512403 | 102.5905191 | 126.9657897 | 157.8480053 | 197.8675919 | 320.0015354 | 517.6065378 | 765.0484733 | 1041.670863 | 1188.70217 | 1228.38535 | 1006.349443 | 596.6789896 | 228.7219526 | 25.31065212 | 8.639650006 | 3.968413967 | 187.1427455 |
| Serbia | 32.52999981 | 118.3295832 | 203.7702969 | 263.4587137 | 325.2691 | 505.4478145 | 788.2211316 | 1025.188608 | 1215.743721 | 1148.817526 | 895.7920169 | 619.9194883 | 313.7719589 | 100.9645643 | 126.7298037 | 49.81778051 | 7.953204853 | 467.3389548 |
| Seychelles | 93.45725723 | 199.1899225 | 273.3018249 | 356.3057419 | 472.6319307 | 677.3989461 | 953.5883563 | 1255.255697 | 1548.528349 | 1832.056863 | 2056.402937 | 1546.194418 | 692.1822797 | 175.4640985 | 163.6139369 | 65.55274572 | 13.0458699 | 602.107816 |
| Sierra Leone | 33.88083585 | 46.79132723 | 59.21158871 | 76.87070028 | 97.81111204 | 150.7731029 | 233.5850389 | 337.9295511 | 460.5660789 | 579.4818821 | 693.205553 | 683.2871525 | 574.3387132 | 298.207028 | 401.2036034 | 155.2063349 | 23.69250113 | 86.19498838 |
| Singapore | 92.92233425 | 149.4409772 | 195.3284456 | 262.271901 | 349.4174163 | 429.447707 | 560.4898316 | 646.1444553 | 747.4499888 | 714.134747 | 584.9105081 | 433.2171987 | 263.9160381 | 95.84016743 | 317.8319186 | 121.8168583 | 17.20098925 | 359.1386849 |
| Slovakia | 25.90852843 | 100.9147189 | 170.2363788 | 209.1037052 | 246.6710893 | 347.6017752 | 505.3074775 | 649.8908094 | 780.7776606 | 751.7363867 | 591.8869032 | 417.8312687 | 223.8685142 | 66.91238113 | 98.62844084 | 39.17961982 | 7.125778504 | 324.1139404 |
| Slovenia | 28.7733399 | 106.4919387 | 174.7661307 | 211.0981898 | 247.4610933 | 330.3095512 | 454.9721171 | 606.0082821 | 777.8312665 | 793.2312944 | 683.7007501 | 510.782437 | 275.4957554 | 78.68055684 | 123.5318327 | 48.52460945 | 8.300489167 | 342.3744303 |
| Solomon Islands | 247.3840033 | 530.6279876 | 631.1233088 | 671.3731168 | 755.5998107 | 841.359398 | 924.8043386 | 924.5536926 | 858.6438213 | 927.562767 | 1094.973977 | 899.3561019 | 458.5449137 | 161.3430631 | 177.4739534 | 78.92766465 | 25.20676509 | 396.6057725 |
| Somalia | 69.84613588 | 98.19807808 | 108.7819747 | 126.5085149 | 153.7864737 | 229.7968583 | 348.1840191 | 462.0470062 | 575.6288734 | 651.0636968 | 2618.079699 | 1941.916933 | 797.4861822 | 182.3799085 | 252.5357288 | 101.8805195 | 17.44870304 | 623.2182751 |
| South Africa | 69.45219624 | 96.9149089 | 113.2808515 | 144.0995111 | 192.4826036 | 370.1399043 | 666.118819 | 1002.196365 | 1343.300304 | 1326.462529 | 740.5114383 | 636.0332341 | 453.7690958 | 216.9673207 | 233.1531032 | 109.8794353 | 39.98723229 | 149.107308 |
| Republic of Korea | 68.93301209 | 113.6327524 | 147.6991785 | 194.0682725 | 250.2662789 | 351.3673631 | 491.5663267 | 632.7794545 | 772.7051849 | 801.5184782 | 740.9244904 | 573.3421592 | 314.7401819 | 108.7360968 | 150.0750846 | 64.0434193 | 16.38534652 | 365.7035052 |
| South Sudan | 59.04234453 | 80.28481347 | 90.91326121 | 108.8973624 | 135.0294965 | 201.8422925 | 311.3633469 | 426.3961951 | 539.8502867 | 634.8703511 | 714.4070354 | 674.2843971 | 547.601152 | 263.6370174 | 179.278566 | 72.8309195 | 18.43507395 | 105.5781093 |
| Spain | 88.15803574 | 156.0501298 | 144.1448636 | 157.4401405 | 200.9920292 | 335.6071383 | 551.4366843 | 808.107607 | 1086.068681 | 1135.668646 | 1018.811731 | 736.5809614 | 314.9247242 | 50.26731257 | 84.73734943 | 26.64675751 | 6.23887594 | 425.7088272 |
| Sri Lanka | 84.89659435 | 172.6067769 | 228.9856956 | 296.0863656 | 394.6786944 | 604.4435117 | 904.7636673 | 1221.917345 | 1525.185773 | 2065.240574 | 1018.761826 | 876.8272378 | 580.5326992 | 253.1048318 | 343.6441876 | 134.2324885 | 20.81197311 | 124.6641188 |
| Sudan | 73.3699239 | 180.8548096 | 238.5519586 | 272.8472306 | 345.0341129 | 574.8584693 | 937.9460524 | 1125.32153 | 1163.125646 | 1044.26291 | 815.2639281 | 589.2880774 | 361.6714534 | 141.9414088 | 197.1095038 | 83.19513292 | 21.3462712 | 246.4823663 |
| Suriname | 175.5650553 | 278.376288 | 323.7835331 | 388.3878076 | 506.6440198 | 860.144144 | 1385.942658 | 1514.348368 | 1357.396731 | 1166.884385 | 959.1077951 | 771.3794077 | 610.5840884 | 282.8796847 | 242.6923109 | 112.9839305 | 39.63119649 | 545.5441764 |
| Eswatini | 71.24149456 | 103.8854212 | 127.7782235 | 166.7610509 | 225.1536863 | 382.5510542 | 630.4890907 | 1044.099799 | 1549.389469 | 1588.187541 | 1307.595733 | 1025.942256 | 761.0704929 | 389.9162875 | 216.5412297 | 89.35416035 | 19.61482987 | 239.0830844 |
| Sweden | 66.48097647 | 113.8848653 | 133.4330583 | 158.3105704 | 194.5125258 | 278.2484186 | 405.6808206 | 556.2921955 | 720.3348049 | 786.6796412 | 773.9452419 | 618.515798 | 340.9181612 | 103.0142178 | 205.0014033 | 84.15607625 | 18.00362898 | 304.1167055 |
| Switzerland | 64.93841907 | 119.1855718 | 118.6243378 | 136.8508708 | 176.8656124 | 275.8984028 | 429.2761376 | 602.4841495 | 787.3251385 | 851.1741236 | 818.5974446 | 628.7426713 | 302.9968844 | 66.9518965 | 132.8463198 | 51.54247246 | 7.359561827 | 328.4372045 |
| Syrian Arab Republic | 73.9076907 | 171.4201798 | 214.5700501 | 240.1183679 | 302.2064699 | 507.5311659 | 836.4357173 | 1059.910225 | 1184.277341 | 1105.096695 | 882.0102243 | 643.7638106 | 388.1113425 | 159.4899944 | 204.5912099 | 82.58306664 | 18.4653307 | 356.3647082 |
| Taiwan (Province of China) | 66.6906138 | 112.0431257 | 112.3058192 | 169.6337116 | 274.9898407 | 409.2559031 | 568.2654401 | 687.0066618 | 767.6182718 | 811.0548585 | 823.6198875 | 701.2514244 | 466.2165842 | 170.4229982 | 269.1280326 | 103.8736511 | 15.4178314 | 388.1056379 |
| Tajikistan | 73.32794594 | 146.4756071 | 191.2041549 | 241.5539174 | 319.3871886 | 511.5236096 | 799.6907354 | 869.1834149 | 758.4831666 | 605.1373255 | 412.5892007 | 276.0716448 | 188.2425702 | 85.23706169 | 345.4190764 | 134.1127089 | 21.59849465 | 230.3990762 |
| United Republic of Tanzania | 52.25881725 | 71.01737412 | 80.09827344 | 96.87417348 | 121.4356973 | 180.2994354 | 272.0351849 | 375.1529903 | 485.9659968 | 585.3641384 | 674.1641207 | 650.4001437 | 545.2313483 | 276.9217118 | 379.3231265 | 145.1447921 | 22.5362701 | 96.93225332 |
| Thailand | 57.54223566 | 110.7694602 | 145.7992842 | 184.942325 | 238.2417587 | 326.7658792 | 447.0600096 | 567.6316098 | 685.913411 | 856.1715126 | 1062.298934 | 955.7336112 | 609.2889018 | 227.5161257 | 343.3902637 | 134.7685617 | 25.96076521 | 359.8363417 |
| Bahamas | 139.6904371 | 220.6094614 | 256.656106 | 299.7220658 | 377.2672799 | 605.7489479 | 958.9745457 | 1083.466634 | 1026.154452 | 966.7253002 | 907.1090377 | 662.7181458 | 512.5807827 | 236.7640923 | 216.4953074 | 92.36975075 | 24.91137968 | 301.7913209 |
| Gambia | 47.9847048 | 68.27949211 | 87.09358688 | 112.0400385 | 142.7378403 | 228.3403423 | 363.8538144 | 520.7650693 | 693.8830944 | 829.0069712 | 916.8279612 | 812.6003711 | 558.0087657 | 262.0849544 | 110.8041931 | 44.9726118 | 8.528904174 | 118.6461537 |
| Timor-Leste | 58.27250888 | 109.8398958 | 143.2719651 | 182.338367 | 235.8544787 | 337.2545903 | 481.3362014 | 617.7629415 | 744.37496 | 955.4157179 | 1217.623745 | 1085.785642 | 665.2133509 | 266.4546858 | 359.181367 | 140.9450013 | 26.22752892 | 198.7514022 |
| Togo | 34.92524384 | 49.82551519 | 64.43664752 | 85.19790709 | 110.148016 | 170.8602964 | 264.987851 | 381.7956809 | 518.721749 | 642.0738873 | 744.4481869 | 686.2918114 | 550.8180572 | 278.6623075 | 194.010798 | 79.85597743 | 16.96304856 | 102.4879291 |
| Tonga | 199.5103577 | 378.1639245 | 426.4922984 | 524.4932858 | 704.6486114 | 942.209918 | 1207.461015 | 1230.898554 | 1089.801815 | 1069.812725 | 1143.064117 | 914.5028329 | 457.0655572 | 127.398495 | 186.5129325 | 77.49936563 | 15.39976967 | 432.324383 |
| Trinidad and Tobago | 128.6995405 | 218.5826019 | 272.4780232 | 377.4639373 | 555.5799164 | 1008.291944 | 1643.389347 | 1831.994604 | 1718.973025 | 1461.821054 | 1136.415158 | 862.4702317 | 627.9875538 | 272.9474814 | 367.4612557 | 144.678957 | 24.59992094 | 699.3211418 |
| Tunisia | 77.28868577 | 194.7484497 | 255.1467748 | 283.0199923 | 350.547713 | 612.4893636 | 1032.863494 | 1310.515426 | 1459.555692 | 1302.571865 | 952.9680073 | 656.0885423 | 390.0605259 | 145.3984383 | 160.0472435 | 65.34281124 | 15.94020637 | 475.4893785 |
| Turkey | 50.20517655 | 118.8191563 | 153.3565268 | 176.0150582 | 221.7604959 | 370.1190205 | 610.2667608 | 803.6084541 | 950.4228635 | 943.9291231 | 818.3024054 | 635.1683704 | 409.5788182 | 157.6267871 | 372.8047771 | 145.1840063 | 22.07330899 | 317.4903998 |
| Turkmenistan | 91.16128142 | 157.7376473 | 190.7617944 | 232.5926427 | 303.300137 | 417.809866 | 568.5838115 | 598.7739934 | 529.8529001 | 438.5020614 | 323.1774888 | 234.4335013 | 170.7916487 | 74.82392053 | 350.5311961 | 147.1115111 | 37.98698312 | 216.9769774 |
| Uganda | 76.39274788 | 105.7874197 | 118.1694725 | 139.9924606 | 172.2685458 | 258.6654519 | 396.5783165 | 537.0047888 | 677.7904196 | 777.1218931 | 828.3376496 | 727.7417994 | 535.2262823 | 250.6789237 | 340.8475448 | 130.2527971 | 20.68185122 | 115.0139962 |
| Ukraine | 34.94879527 | 108.2526165 | 174.6627986 | 197.5809167 | 213.4043846 | 245.1466184 | 292.0929535 | 325.1421517 | 346.574925 | 325.0134766 | 260.9288828 | 205.005862 | 159.3226944 | 81.70765388 | 107.4179919 | 144.0085247 | 37.19436983 | 190.4756291 |
| United Arab Emirates | 76.34144602 | 189.2809429 | 241.1119837 | 266.5617258 | 338.8132622 | 781.2146691 | 1509.711533 | 2288.464177 | 2929.830442 | 2267.723591 | 1134.629696 | 560.2042424 | 299.1149016 | 104.6842412 | 547.7292306 | 220.7680475 | 44.76749768 | 691.0010387 |
| United Kingdom | 175.4136268 | 358.4337973 | 403.0494167 | 393.6159141 | 430.2339242 | 515.0352398 | 641.0964853 | 732.0469903 | 792.2333401 | 894.7721184 | 1029.530214 | 707.5993321 | 329.3376064 | 74.26403082 | 342.8283132 | 140.6502059 | 31.33574688 | 377.567444 |
| United States of America | 72.38764819 | 60.52943179 | 121.1655964 | 287.1495454 | 485.437 | 656.0809345 | 796.2016052 | 1075.918654 | 1444.036758 | 1309.149169 | 834.4183176 | 469.1488482 | 174.6537632 | 14.7960453 | 23.67635248 | 7.885860281 | 4.510335616 | 477.5340672 |
| Uruguay | 24.53676857 | 70.81259753 | 83.53950835 | 105.1609031 | 409.219897 | 633.907056 | 950.4600534 | 1185.197217 | 1345.335467 | 1244.79624 | 599.8414221 | 428.7097602 | 211.5843989 | 49.47365509 | 79.61304257 | 148.3955522 | 32.27116307 | 232.9501134 |
| Uzbekistan | 68.38660585 | 133.7917745 | 170.8830879 | 215.4357235 | 289.0896796 | 489.3223228 | 796.036328 | 899.9599052 | 838.6453258 | 665.8825931 | 394.3453923 | 235.3639092 | 178.9139775 | 101.5928356 | 115.7283666 | 47.91227648 | 10.25426555 | 254.9785012 |
| Vanuatu | 213.1647113 | 419.3449989 | 492.4119241 | 584.7094697 | 738.7489997 | 894.8866051 | 1045.649617 | 1039.146107 | 916.6423457 | 893.9816298 | 952.4408017 | 777.9462383 | 413.8521523 | 150.2373099 | 185.6653014 | 76.26368421 | 15.90354316 | 387.4312746 |
| Venezuela (Bolivarian Republic of) | 136.4698774 | 180.0868957 | 185.1034269 | 239.8056904 | 340.9284464 | 540.231559 | 820.3070871 | 1066.459941 | 1272.893612 | 1178.181689 | 866.4590259 | 617.036328 | 415.0522986 | 166.3967831 | 249.7870945 | 103.7792548 | 18.86986852 | 396.8356976 |
| Viet nam | 46.57804494 | 86.43420092 | 113.6644101 | 148.7027031 | 196.9792528 | 277.8594055 | 388.4887807 | 554.0505213 | 765.9992899 | 1078.235427 | 1445.974261 | 1275.88538 | 730.5147491 | 257.7659163 | 342.7393471 | 144.5592407 | 37.76177309 | 290.9814398 |
| Virginia | 74.4793763 | 60.51984289 | 122.6478636 | 298.105858 | 510.16257 | 677.4158126 | 800.9207655 | 1068.239648 | 1430.581704 | 1298.25942 | 827.168276 | 464.9937113 | 173.4088698 | 14.79446228 | 105.9108605 | 43.90202285 | 10.34981603 | 484.9793259 |
| Yemen | 49.37885019 | 117.7427079 | 156.1363928 | 183.4995626 | 232.8862964 | 364.5283544 | 570.2561335 | 696.145437 | 749.3579586 | 727.0764511 | 643.3453741 | 518.2722836 | 356.559507 | 162.909432 | 23.70778424 | 8.525029935 | 3.668959799 | 154.0610607 |
| Zambia | 65.86447993 | 91.31640497 | 101.5481499 | 121.238231 | 151.1935655 | 230.4944834 | 360.0191862 | 481.0571221 | 595.2011777 | 704.7788108 | 798.263322 | 737.4926541 | 573.0606835 | 288.5475843 | 379.2032704 | 145.0049484 | 21.05599694 | 112.4788861 |
| Zimbabwe | 89.37855432 | 87.74864633 | 106.4755931 | 130.6884009 | 112.804201 | 173.9317465 | 270.7566368 | 351.078554 | 419.1014955 | 491.4243222 | 563.9746806 | 532.1033753 | 417.9747133 | 206.426266 | 157.7109406 | 64.69569762 | 13.02937038 | 142.6173576 |
| Monaco | 77.27565693 | 135.6462115 | 128.3867987 | 142.0027491 | 179.005376 | 272.7724967 | 414.0687004 | 543.9511978 | 660.9331716 | 705.5025471 | 695.9619837 | 546.7802587 | 274.1583025 | 64.48290085 | 107.3525011 | 44.28616304 | 10.72841041 | 330.0107098 |
| San Marino | 76.60047982 | 135.0835955 | 126.8569954 | 141.669279 | 182.6083078 | 286.0477688 | 446.7648075 | 613.3111044 | 778.4862586 | 840.6580969 | 813.7485467 | 625.3655141 | 299.8057674 | 66.35269958 | 108.9563173 | 39.86224759 | 4.830668676 | 322.1578305 |
| Saint Kitts and Nevis | 124.7449146 | 190.6440052 | 220.5792258 | 285.7591671 | 404.6669403 | 669.7830608 | 1050.432958 | 1259.918765 | 1327.229226 | 1269.785354 | 1130.452238 | 938.301282 | 717.0233154 | 333.2733316 | 160.1204799 | 67.14710715 | 16.47311583 | 540.2549091 |
| Cook Islands | 235.4601557 | 458.3172202 | 535.7785693 | 661.8313175 | 872.3133823 | 1114.445777 | 1358.652525 | 1382.917777 | 1255.640555 | 1261.155792 | 1365.570895 | 1056.81438 | 485.6351988 | 115.9685382 | 166.4789444 | 70.11741122 | 13.35567375 | 683.0805198 |
| Nauru | 229.093609 | 451.8241411 | 535.4865737 | 637.4462646 | 793.9189038 | 961.8464829 | 1133.003843 | 1137.348028 | 1017.930438 | 1011.167111 | 1081.447642 | 879.9529286 | 454.9863112 | 150.7782631 | 343.1706775 | 144.5255747 | 37.96512274 | 419.8830329 |
| Niue | 362.6416613 | 105.5893662 | 135.0852727 | 188.3539266 | 264.6982459 | 397.5847074 | 578.5180392 | 727.1568911 | 842.9724399 | 851.6617419 | 778.182145 | 672.7208763 | 544.8288466 | 254.1288852 | 377.5925557 | 56.97293446 | 10.34231234 | 303.0897274 |
| Palau | 267.1441606 | 546.073528 | 654.0916213 | 777.9907382 | 963.3365066 | 1166.763902 | 1378.377617 | 1362.872078 | 1187.186477 | 1115.855555 | 1128.563513 | 880.1570655 | 427.875372 | 119.3604597 | 167.18997 | 69.92882755 | 13.20639838 | 782.7479266 |
| Tokelau | 231.2784364 | 437.9161157 | 494.7005409 | 594.4060707 | 750.9627012 | 967.0319571 | 1120.122439 | 1148.922509 | 1021.874634 | 1000.465832 | 1050.049053 | 861.8167676 | 426.6971252 | 132.9451819 | 336.5231988 | 131.5922987 | 21.48264986 | 480.2603506 |
| Tuvalu | 235.5873776 | 460.1427528 | 537.4469657 | 643.4430587 | 813.0577032 | 1004.01684 | 1205.804995 | 1203.039712 | 1050.109668 | 1001.853182 | 1037.671857 | 825.4659154 | 421.9319315 | 131.1453706 | 178.1849508 | 73.94583521 | 14.94019976 | 514.5517995 |

**Supplementary Table 7.** Age distribution of deaths rate for type 2 diabetes in different countries in 2019.

| 2019death rate | 15 to 19 | 20 to 24 | 25 to 29 | 30 to 34 | 35 to 39 | 40 to 44 | 45 to 49 | 50 to 54 | 55 to 59 | 60 to 64 | 65 to 69 | 70 to 74 | 75 to 79 | 80 plus | 80-84 | 85-89 | 90-94 | all ages |
| --- | --- | --- | --- | --- | --- | --- | --- | --- | --- | --- | --- | --- | --- | --- | --- | --- | --- | --- |
| Afghanistan | 0.308030342 | 0.612190955 | 0.854131613 | 1.913304549 | 3.628862915 | 1.991591143 | 6.099627079 | 10.30446945 | 15.74469563 | 28.41085798 | 39.32714005 | 69.76134308 | 115.4838455 | 232.1327027 | 178.0739444 | 234.41185 | 312.5266935 | 25.4184514 |
| Albania | 0.015232897 | 0.049325375 | 0.076801279 | 0.171433372 | 0.166864899 | 0.222985487 | 0.876369617 | 1.51172465 | 2.93593088 | 7.115840009 | 13.52908781 | 25.5988537 | 40.00985774 | 59.09601378 | 56.38154248 | 54.72668572 | 75.56245138 | 5.490918243 |
| Algeria | 0.088343405 | 0.131601809 | 0.191963665 | 0.489782278 | 0.954097083 | 1.496729595 | 4.461335561 | 9.740542463 | 20.13062192 | 37.46762454 | 62.60214676 | 96.75511965 | 146.0555113 | 246.8905269 | 210.9954261 | 259.9372564 | 355.9261924 | 14.88533362 |
| American Samoa | 0.378423228 | 0.919687586 | 1.916577963 | 5.430841231 | 12.36772841 | 4.655707021 | 11.91614206 | 24.94872564 | 40.61778872 | 73.60193223 | 107.1232863 | 155.0301394 | 252.6499082 | 502.338026 | 428.6307659 | 567.2669369 | 773.8470711 | 19.0284597 |
| Andorra | 0.006287393 | 0.010910178 | 0.022911337 | 0.097099117 | 0.154781659 | 2.229452686 | 6.176599514 | 12.93272766 | 22.52947449 | 41.83460263 | 62.79767074 | 91.72022343 | 148.628743 | 332.9196693 | 225.1156092 | 533.2959047 | 688.0141266 | 11.93697788 |
| Angola | 0.285127798 | 0.529362165 | 0.594642109 | 1.735211657 | 3.493028569 | 7.936426943 | 19.91273328 | 39.77976236 | 60.48207723 | 93.11019518 | 142.0821824 | 159.7033885 | 289.6520002 | 561.7805832 | 480.0742165 | 596.7788387 | 903.4595036 | 11.82532752 |
| Antigua and Barbuda | 0.395382613 | 0.650472832 | 0.93919198 | 1.956480046 | 4.760582158 | 6.206239567 | 18.73709806 | 41.93148188 | 78.99968654 | 146.4848951 | 211.3206346 | 354.421944 | 549.4589704 | 1057.090562 | 915.6893382 | 1072.892172 | 1458.196999 | 60.81742094 |
| Argentina | 0.09157133 | 0.164606645 | 0.239362913 | 0.634795811 | 1.103192843 | 11.8625435 | 31.72499803 | 72.0082549 | 139.2596678 | 305.9695408 | 444.5583751 | 644.8766484 | 882.1848219 | 1138.377608 | 235.7747981 | 376.7117118 | 566.1883456 | 44.29779674 |
| Armenia | 0.08975778 | 0.18300363 | 0.235792766 | 0.664587357 | 1.114175847 | 1.30714564 | 6.527761361 | 14.65260969 | 32.92488809 | 66.15505324 | 131.3123109 | 233.6007808 | 427.02296 | 584.5811439 | 559.8055233 | 635.0871702 | 652.9285839 | 46.50690367 |
| Australia | 0.016907707 | 0.031823765 | 0.254493448 | 1.080273389 | 1.935368356 | 1.830633486 | 5.504533236 | 9.090782374 | 11.50244716 | 15.71988971 | 25.09956578 | 28.93287281 | 31.65183493 | 40.99138724 | 229.0587068 | 303.137357 | 393.4720942 | 3.346452098 |
| Austria | 0.019050974 | 0.040868771 | 0.061261007 | 0.21551791 | 0.360542163 | 0.286440731 | 1.009688923 | 2.224269102 | 4.569216458 | 8.486710037 | 15.50284867 | 31.64992758 | 66.65451193 | 257.4750139 | 135.4130799 | 263.1244333 | 478.4821751 | 21.75348544 |
| Azerbaijan | 0.197060233 | 0.350822985 | 0.369451447 | 0.782533968 | 1.167520923 | 1.223802406 | 3.44216805 | 7.825411849 | 15.72360876 | 31.59035279 | 60.34479886 | 119.2588636 | 185.8794429 | 419.4881671 | 1009.282536 | 1300.729653 | 1540.236302 | 8.006007551 |
| Bahrain | 0.492573675 | 0.82592704 | 1.175260266 | 1.436890788 | 2.487693902 | 6.678043143 | 18.92972639 | 42.7602902 | 95.89688909 | 202.6546438 | 413.6786036 | 836.5243899 | 1398.594276 | 2381.175172 | 2015.36979 | 3065.041139 | 4493.88 | 48.52742319 |
| Bangladesh | 0.361595285 | 0.335710115 | 0.47668649 | 0.953890818 | 1.579985205 | 19.75332506 | 39.62561876 | 78.75881282 | 140.7300626 | 205.3766198 | 244.8324508 | 328.1024787 | 432.3086465 | 552.8957182 | 478.2621973 | 611.0446226 | 790.9445559 | 30.82899704 |
| Barbados | 0.689457275 | 1.035912161 | 1.611050057 | 3.279438168 | 6.179215281 | 4.221821759 | 9.861356774 | 18.61535676 | 31.30960161 | 52.72237981 | 74.9144987 | 108.5424166 | 161.6668057 | 278.4522611 | 494.4135727 | 571.5004235 | 635.5216069 | 19.00240811 |
| Belarus | 0.01430594 | 0.027668392 | 0.080093322 | 0.251921815 | 0.343629175 | 0.355506642 | 1.369378041 | 2.18414984 | 3.919039024 | 6.607464075 | 9.965500526 | 12.12266341 | 16.37386565 | 33.20342802 | 24.49015297 | 35.92782951 | 58.63516948 | 3.762706472 |
| Belgium | 0.010473747 | 0.022167131 | 0.034217466 | 0.131271376 | 0.225378034 | 0.399346287 | 1.644058574 | 2.863461934 | 5.597040405 | 10.22103458 | 17.22395339 | 29.71193227 | 49.20751484 | 161.3377001 | 95.90212833 | 168.5437693 | 281.9481927 | 14.5548374 |
| Belize | 0.723702267 | 1.166556957 | 1.975946389 | 4.650094035 | 6.081840006 | 6.540940591 | 15.65528984 | 31.94829062 | 57.23910576 | 99.48748031 | 162.8368539 | 263.1028489 | 367.8520023 | 729.5699708 | 555.9174028 | 884.8957155 | 1474.758578 | 22.0852686 |
| Benin | 0.80549915 | 0.470959697 | 0.835175952 | 1.387364354 | 1.769700393 | 0.398646644 | 1.247986052 | 2.026197528 | 3.150694469 | 4.762813887 | 6.671134134 | 10.74332558 | 16.87087546 | 49.41099408 | 28.88502425 | 48.24448189 | 79.44080216 | 7.33868513 |
| Bermuda | 0.107488924 | 0.176239841 | 0.212828487 | 0.523358673 | 0.951163727 | 4.869148213 | 14.19004047 | 31.08402642 | 50.17886221 | 88.3309524 | 117.275844 | 203.0474749 | 292.8876832 | 495.7167845 | 432.1794202 | 537.4500609 | 775.0520589 | 11.42393504 |
| Bhutan | 0.153523542 | 0.198422781 | 0.293572999 | 0.760551529 | 1.151910156 | 0.753975172 | 2.157894413 | 3.879037675 | 6.485888318 | 10.03990969 | 13.54127367 | 22.2874882 | 38.64224834 | 82.11670859 | 466.0732136 | 592.3972722 | 761.2945583 | 11.47376757 |
| Bolivia (Plurinational State of) | 0.195683492 | 0.315839008 | 0.525828712 | 1.219224676 | 5.647826238 | 5.992193123 | 17.08449477 | 37.00236147 | 72.82623637 | 126.5857944 | 202.882033 | 320.3166444 | 464.3915476 | 619.9002184 | 586.5507682 | 638.0910394 | 744.8693341 | 30.53839537 |
| Bosnia and Herzegovina | 0.169535898 | 0.326121003 | 0.50005883 | 0.972213584 | 1.323412726 | 0.632513272 | 2.215813525 | 4.492801546 | 8.686796865 | 18.14808703 | 33.01084892 | 54.43150423 | 95.25417694 | 288.4708779 | 193.2320544 | 308.3181334 | 460.6074356 | 25.61145889 |
| Botswana | 0.865212188 | 0.931045353 | 0.958002997 | 1.610132532 | 4.056648714 | 2.362457332 | 10.34201714 | 21.99271115 | 46.96280979 | 86.72211548 | 139.169011 | 167.9579969 | 199.0994408 | 199.5190894 | 232.2615472 | 307.870835 | 368.1934019 | 19.00403334 |
| Brazil | 0.226529433 | 0.378824573 | 0.296809511 | 0.676950836 | 1.257018359 | 5.977743665 | 14.43913682 | 27.30598833 | 48.16976987 | 82.96829724 | 128.1415329 | 187.2241835 | 266.018866 | 464.1194691 | 322.9334461 | 572.4949793 | 819.9339855 | 37.66894701 |
| Brunei Darussalam | 0.3568574 | 0.732828104 | 1.13817368 | 2.891488677 | 5.233855016 | 5.838648976 | 15.70174798 | 31.90114483 | 47.48210406 | 86.97273048 | 151.0108433 | 190.3168041 | 322.3566037 | 563.2684241 | 489.5211503 | 565.3127265 | 844.5393684 | 12.2627539 |
| Bulgaria | 0.076227841 | 0.154083654 | 0.26802106 | 0.62067397 | 0.730365842 | 2.903358038 | 8.601665271 | 14.15149095 | 21.46655045 | 32.42855127 | 49.38305631 | 73.06704738 | 105.9439099 | 232.4513046 | 161.5412057 | 235.385701 | 327.2834358 | 24.66013961 |
| Burkina Faso | 0.815167791 | 0.393539072 | 0.900024686 | 1.937348865 | 2.189030629 | 2.392195112 | 7.28106894 | 15.11046705 | 31.85025506 | 68.55226393 | 136.6452655 | 273.9423795 | 445.8777066 | 671.2393284 | 648.3689724 | 701.7149485 | 731.0473943 | 66.94940701 |
| Burundi | 0.305553548 | 0.495082402 | 0.505775992 | 1.357065841 | 2.924837189 | 7.240892569 | 17.66598615 | 36.20968888 | 61.60387411 | 113.8701656 | 198.3062191 | 349.1442188 | 508.2475465 | 1100.48968 | 827.7557547 | 1235.551655 | 1960.246277 | 58.29271929 |
| Cambodia | 0.451994201 | 0.650957573 | 0.906851317 | 1.915185075 | 3.285480727 | 1.678520414 | 5.447672487 | 9.245172609 | 15.08478253 | 23.37765156 | 33.94104971 | 54.31691453 | 82.30554141 | 177.6217339 | 126.0072818 | 188.2128994 | 275.0160268 | 15.56894956 |
| Cameroon | 1.09729234 | 0.65749591 | 1.329146138 | 2.317696943 | 2.819937161 | 11.50382594 | 27.00741774 | 50.65073397 | 104.0444598 | 160.0350554 | 249.1591166 | 367.3345069 | 523.9280065 | 842.3796722 | 700.4258846 | 972.4189486 | 1198.929038 | 50.66032044 |
| Canada | 0.011399646 | 0.022394372 | 0.04745445 | 0.220145129 | 0.327497237 | 4.364850231 | 12.40269849 | 28.17928533 | 48.28515384 | 91.56970702 | 125.6002754 | 218.0999567 | 324.3557504 | 551.1859682 | 478.3642588 | 579.983842 | 816.1338349 | 11.2541833 |
| Cabo Verde | 0.524317991 | 0.265784916 | 0.430166561 | 0.74463682 | 0.882988619 | 3.292624037 | 9.591179163 | 21.60351393 | 37.06030555 | 69.80965161 | 95.40857093 | 173.3880053 | 255.5393523 | 421.6448883 | 373.8007802 | 428.7956588 | 658.5867456 | 9.445227727 |
| Central African Republic | 0.391674514 | 0.74359633 | 4.309903457 | 8.924504446 | 16.41032801 | 4.890747941 | 11.48305406 | 22.15655962 | 39.67062636 | 68.93136855 | 109.9482588 | 172.7421207 | 261.1179621 | 464.1557312 | 706.8053076 | 871.6913217 | 1130.815277 | 29.02300008 |
| Chad | 0.849609974 | 0.494500724 | 0.82993634 | 1.335283687 | 1.866180409 | 0.400986053 | 3.096429544 | 4.896289546 | 7.359280968 | 13.24464581 | 21.19870639 | 31.02303295 | 41.99285526 | 56.08049798 | 51.20738255 | 54.03268629 | 74.23697018 | 9.381714861 |
| Chile | 0.03641622 | 0.065360487 | 0.107440785 | 0.298476251 | 0.595996425 | 2.101981905 | 7.242277672 | 14.41058069 | 28.70887425 | 52.13047053 | 85.19279843 | 122.2528188 | 177.5259512 | 281.8828862 | 195.3585651 | 290.9547413 | 367.7118684 | 22.64953025 |
| China | 0.03661339 | 0.104556508 | 0.1518823 | 0.380220688 | 0.711638445 | 4.698564433 | 11.52928155 | 22.42893093 | 37.7851405 | 58.86364577 | 85.27845172 | 121.2345841 | 166.9746121 | 212.5730007 | 133.3771902 | 195.7097722 | 214.7096575 | 10.86727247 |
| Colombia | 0.165260847 | 0.282883203 | 0.462135939 | 0.941663624 | 1.415637824 | 2.442182391 | 4.972727172 | 9.748595383 | 18.06620531 | 33.03968834 | 51.14120397 | 81.54302765 | 124.18887 | 213.2433354 | 168.9294698 | 219.6317232 | 278.8433247 | 14.42499929 |
| Comoros | 0.214742604 | 0.401631974 | 0.4445793 | 1.183807527 | 2.623283929 | 5.965903494 | 16.90665379 | 36.43719421 | 60.27877318 | 108.7299474 | 138.8504665 | 224.5049228 | 315.7997657 | 518.8244749 | 459.3200399 | 564.2189485 | 796.1035199 | 11.13312772 |
| Congo | 0.286681581 | 0.540550046 | 0.767728681 | 1.910938955 | 4.46493465 | 6.261096415 | 13.67227129 | 25.3532121 | 47.46426157 | 80.65286298 | 133.2932456 | 200.3339834 | 309.5314874 | 508.6313029 | 418.7859676 | 577.7671139 | 580.231923 | 32.47545671 |
| Costa Rica | 0.093495597 | 0.159321823 | 0.344812369 | 0.694916893 | 1.048791293 | 0.996921504 | 4.592134695 | 9.245811006 | 17.84007432 | 32.24009803 | 49.40559694 | 68.34882323 | 98.36151965 | 131.2062055 | 120.8268213 | 134.4135409 | 162.9867129 | 15.55024558 |
| C么te d'Ivoire | 0.893536147 | 0.521637535 | 0.954209167 | 1.595454557 | 1.904064638 | 6.901546196 | 17.65191139 | 35.64287602 | 52.9128173 | 95.94749879 | 169.1630481 | 211.9394441 | 363.1876178 | 612.1066015 | 550.9435637 | 616.4895907 | 909.6732605 | 12.38720544 |
| Croatia | 0.008666216 | 0.029185531 | 0.049996969 | 0.175840533 | 0.256702007 | 0.43001884 | 1.938165041 | 4.341849476 | 9.986492908 | 19.92685859 | 37.19995793 | 69.93807582 | 115.6377261 | 241.5856218 | 199.2057546 | 271.7737107 | 373.2390048 | 25.5195141 |
| Cuba | 0.104130862 | 0.188010027 | 0.305717619 | 0.59541878 | 0.937659717 | 1.577536411 | 4.081924055 | 8.40013012 | 15.30641553 | 26.11308783 | 42.97527327 | 65.40079363 | 102.2699007 | 153.6261851 | 128.1353844 | 157.1752937 | 200.0277384 | 17.32195523 |
| Cyprus | 0.010246927 | 0.021546271 | 0.063820098 | 0.126386796 | 0.315755688 | 4.834774304 | 11.0873282 | 21.53407785 | 37.96890369 | 65.7991254 | 101.4788531 | 155.8325779 | 227.9315604 | 369.06034 | 315.5238174 | 410.3422282 | 456.4266356 | 23.18209329 |
| Czechia | 0.013576853 | 0.043990653 | 0.054789198 | 0.157196104 | 0.241732026 | 1.597678445 | 5.373733441 | 9.562999051 | 14.21632406 | 25.30154394 | 34.3600608 | 49.60747707 | 72.81471239 | 158.2827523 | 117.9425919 | 166.4880308 | 280.0560253 | 13.9695723 |
| Democratic Republic of the Congo | 0.259441491 | 0.50314934 | 0.522833169 | 1.551858752 | 3.223787062 | 6.594062696 | 18.69603259 | 42.34025445 | 64.56099748 | 106.3038696 | 130.2732809 | 205.134612 | 287.7119746 | 499.4456691 | 430.6198055 | 535.509293 | 829.3331423 | 11.84658725 |
| Denmark | 0.017681085 | 0.041086146 | 0.076833637 | 0.319435185 | 0.481384928 | 0.772335712 | 3.574738227 | 7.460585918 | 11.72508258 | 20.94369671 | 33.19863451 | 55.93982938 | 96.39883071 | 251.521789 | 167.4777976 | 270.2410529 | 408.1844432 | 23.28683987 |
| Djibouti | 0.243233298 | 0.46904441 | 0.481098765 | 1.394430498 | 2.929385723 | 3.911687211 | 12.34376456 | 20.13217202 | 29.71075306 | 46.05396158 | 59.09969197 | 86.38342958 | 119.4376842 | 240.2724783 | 583.3582205 | 720.9868465 | 1031.092404 | 27.3666282 |
| Dominica | 0.833019984 | 1.343024807 | 1.759381709 | 3.701858172 | 6.388075866 | 2.574548636 | 7.1082985 | 14.12450557 | 21.52702795 | 39.66518385 | 54.94800441 | 88.95219457 | 136.4511294 | 311.1581698 | 219.3183763 | 336.7951053 | 513.7010103 | 19.65436343 |
| Dominican Republic | 0.730355666 | 1.45906124 | 1.858978915 | 3.643094749 | 5.569806681 | 2.163451414 | 11.09949291 | 24.71154837 | 53.70435377 | 105.6519463 | 147.8852674 | 175.9539625 | 198.103946 | 177.2366111 | 193.610902 | 132.2763583 | 172.0641277 | 17.70906359 |
| Ecuador | 0.281282606 | 0.511231979 | 0.912918881 | 1.986732799 | 3.429391114 | 2.466999557 | 6.350161152 | 12.92358939 | 23.23547293 | 39.71480033 | 59.96020624 | 88.68262627 | 116.1146154 | 173.0318907 | 143.1658952 | 181.4539521 | 285.3301418 | 10.32837103 |
| Egypt | 0.207681268 | 0.307901043 | 0.57331847 | 1.271287584 | 2.178473884 | 5.000146689 | 16.90510386 | 41.19787322 | 72.44612662 | 125.1120206 | 172.78554 | 208.0195589 | 249.6917441 | 384.1258633 | 326.7772209 | 445.2310652 | 593.6907289 | 19.41344088 |
| El Salvador | 0.268244911 | 0.481827912 | 1.091579959 | 2.94578387 | 5.146409448 | 4.56886112 | 11.63092042 | 24.40932962 | 39.83174601 | 72.43692439 | 104.7246618 | 150.4110816 | 247.3317436 | 490.9211276 | 421.4265303 | 553.8335903 | 747.8631387 | 18.71924347 |
| Equatorial Guinea | 0.295546786 | 0.590239515 | 0.709960352 | 2.044615232 | 4.096793036 | 0.310809745 | 1.051034673 | 1.642774612 | 2.30711469 | 3.846393986 | 5.520224105 | 9.321811744 | 14.29854678 | 41.95145569 | 23.66087065 | 36.69512153 | 66.46579837 | 7.047120361 |
| Eritrea | 0.262954378 | 0.512776283 | 0.571792461 | 1.714127791 | 3.978498644 | 0.462121566 | 1.374225687 | 3.079045078 | 6.336173921 | 11.18940513 | 20.13403492 | 31.8343331 | 54.41545707 | 88.47163432 | 61.91495958 | 83.5228124 | 111.6055655 | 13.05512307 |
| Estonia | 0.048489303 | 0.033684227 | 0.047597812 | 0.129780458 | 0.341169741 | 3.7804244 | 8.548460344 | 17.07363202 | 32.74630086 | 57.44175781 | 93.14868395 | 150.871938 | 224.7427891 | 404.9429252 | 329.3930088 | 443.9079545 | 529.5687025 | 32.2517281 |
| Ethiopia | 0.19739311 | 0.340708278 | 0.354958138 | 1.016603577 | 2.115616806 | 2.95061985 | 9.562847069 | 16.3197577 | 25.7493957 | 39.66891359 | 53.19025129 | 71.22921565 | 103.5602536 | 195.7980883 | 500.702781 | 626.8658273 | 877.0766171 | 23.12609742 |
| Micronesia (Federated States of) | 0.762607978 | 2.421880848 | 5.054890606 | 13.67029869 | 4.13336266 | 55.27843872 | 134.5010442 | 265.9653216 | 395.1948786 | 70.24938628 | 111.8300444 | 175.3855183 | 265.1684722 | 469.1178125 | 374.0871046 | 517.4622167 | 617.3177475 | 29.19239327 |
| Fiji | 1.198498554 | 2.177089585 | 4.055245049 | 10.08158355 | 25.75319638 | 1.081755788 | 4.11412948 | 10.0525574 | 18.62380585 | 32.6434712 | 58.54530084 | 103.9732639 | 193.6107889 | 529.1378665 | 328.5464281 | 535.5176831 | 895.9243314 | 27.85634208 |
| Finland | 0.015096844 | 0.031201433 | 0.049012845 | 0.226636573 | 0.282638342 | 26.76657079 | 58.43259024 | 99.70193524 | 156.6513821 | 221.9468877 | 263.5003963 | 315.1144785 | 399.8395553 | 528.0190912 | 467.7223073 | 598.0017329 | 726.988494 | 37.24734662 |
| France | 0.012760317 | 0.028956158 | 0.051586755 | 0.155729019 | 0.251283071 | 30.485788 | 67.68512808 | 118.6019477 | 180.8272978 | 246.0151013 | 289.4016505 | 350.5445892 | 426.190338 | 532.7746907 | 496.7728642 | 573.6219725 | 708.1191313 | 25.27068811 |
| Gabon | 0.388403526 | 0.761662655 | 0.830385387 | 2.483002725 | 5.262414553 | 10.795095 | 30.27007238 | 68.93744621 | 113.1945079 | 195.2218412 | 257.7624211 | 400.7407843 | 558.1004764 | 959.3418432 | 800.0195928 | 1055.748755 | 1630.867334 | 33.58691402 |
| Georgia | 0.058540589 | 0.189061602 | 0.150298857 | 0.515566977 | 0.872913702 | 11.37741819 | 29.77272608 | 55.65983843 | 82.22906759 | 139.2285924 | 230.4253714 | 272.8855703 | 415.6730664 | 659.3122701 | 603.0544847 | 682.8864387 | 960.5238279 | 15.50221381 |
| Germany | 0.014582819 | 0.02582742 | 0.04480913 | 0.173968322 | 0.296373549 | 20.48906891 | 43.18482295 | 85.39932975 | 136.6241378 | 204.1120209 | 302.3649642 | 402.6841466 | 588.3614643 | 948.2479618 | 819.3748677 | 998.750823 | 1272.387901 | 60.24139059 |
| Ghana | 0.898088956 | 0.394408214 | 0.910930645 | 1.96645287 | 2.309122512 | 0.808513104 | 4.014030947 | 8.572124473 | 16.75530648 | 28.33567003 | 45.91810434 | 73.88898628 | 125.6428578 | 197.5889356 | 178.709731 | 220.3921717 | 219.4585092 | 24.7292739 |
| Greece | 0.013885733 | 0.025876752 | 0.03797543 | 0.127729761 | 0.209296845 | 4.287362495 | 11.68911807 | 17.9273796 | 27.34603124 | 41.96201804 | 58.57240133 | 89.75660285 | 137.828503 | 268.6355602 | 756.0753111 | 968.0710204 | 1179.413379 | 29.55552755 |
| Greenland | 0.016396445 | 0.044972658 | 0.098553236 | 0.630373619 | 0.952053489 | 19.52894908 | 47.95167067 | 98.08185616 | 169.559718 | 265.7238697 | 391.0082281 | 539.8817856 | 741.2446461 | 892.311833 | 918.7209501 | 888.7486084 | 764.9909601 | 73.34404994 |
| Grenada | 0.77325457 | 1.164450156 | 1.473626222 | 3.972477808 | 6.840892683 | 13.49224537 | 29.1650218 | 57.81348798 | 99.08399975 | 155.3974161 | 245.317781 | 367.7293546 | 477.8721777 | 726.5059501 | 610.7797719 | 800.0347346 | 912.2395244 | 53.67029909 |
| Guam | 0.139845848 | 0.187604044 | 0.477544371 | 1.587698764 | 1.809020389 | 8.99784491 | 16.90118114 | 37.64109489 | 52.13488956 | 536.22765 | 727.5410355 | 982.941533 | 1242.515713 | 1895.145582 | 1639.661374 | 2241.450083 | 2503.790564 | 104.8916958 |
| Guatemala | 0.521256528 | 0.931859185 | 1.874072122 | 5.313071234 | 11.10873268 | 1.197742676 | 3.887869298 | 7.037586042 | 12.33273286 | 20.97367991 | 30.99375177 | 47.83606219 | 76.75543078 | 193.8048498 | 129.1259983 | 202.6508624 | 306.0494567 | 19.81490361 |
| Guinea | 0.943188364 | 0.636273408 | 1.110397117 | 1.560296366 | 2.362582202 | 9.717667981 | 24.5961977 | 42.8062719 | 76.04828301 | 117.8087429 | 165.6127864 | 225.5735584 | 316.5733597 | 429.0200894 | 385.375446 | 454.1472814 | 465.4038763 | 34.88155314 |
| Guinea-Bissau | 1.178034286 | 0.734395692 | 1.458880822 | 2.310485471 | 3.715643875 | 5.277252777 | 10.02389799 | 18.07647379 | 28.69706155 | 51.69182724 | 86.44057278 | 129.5210233 | 165.0875316 | 228.6563538 | 208.0707396 | 195.9167316 | 296.7943321 | 26.15541066 |
| Guyana | 1.055091743 | 2.057557061 | 2.961428164 | 6.608167102 | 10.42213379 | 2.210595332 | 7.517917892 | 13.9488075 | 21.13433167 | 33.72357602 | 48.07934904 | 70.26370812 | 102.3417379 | 220.8746515 | 153.2046993 | 225.8687412 | 323.439383 | 21.91818772 |
| Haiti | 0.757037376 | 1.186946575 | 1.518777073 | 3.243490351 | 5.608688834 | 10.65106215 | 27.45636773 | 54.16377527 | 83.82572191 | 138.2698017 | 215.401262 | 313.3327049 | 428.9998498 | 834.5304867 | 717.4969103 | 737.1073554 | 894.3458782 | 40.0357556 |
| Honduras | 0.22920783 | 0.284482684 | 0.576594587 | 1.211457042 | 2.165620459 | 1.662838449 | 5.019656048 | 11.66054695 | 18.87955549 | 31.55927776 | 69.13431755 | 117.6327873 | 166.4693094 | 253.5720562 | 295.6510719 | 393.1583273 | 507.4424356 | 15.85512356 |
| Hungary | 0.023024614 | 0.040519889 | 0.092238651 | 0.298325781 | 0.432150477 | 6.097744514 | 14.44526584 | 28.15670554 | 52.40771127 | 85.26872658 | 144.8098316 | 234.5548693 | 333.1284322 | 638.3783479 | 513.590123 | 686.3581145 | 918.2424276 | 29.91708358 |
| Iceland | 0.004705332 | 0.008842136 | 0.067885377 | 0.304241984 | 0.448588305 | 4.219119492 | 9.858052452 | 18.60957295 | 31.29700332 | 52.69044755 | 74.88727297 | 108.5699036 | 161.6991798 | 278.5790981 | 147.9500028 | 251.1345496 | 393.1910556 | 19.02005763 |
| India | 0.151529482 | 0.212945604 | 0.340345845 | 0.93123955 | 1.441743879 | 1.171009413 | 5.000812851 | 9.835126531 | 23.62813922 | 47.35700713 | 83.28105599 | 120.7191341 | 169.1440726 | 187.1352113 | 200.7755706 | 145.0069545 | 186.3163068 | 13.25107269 |
| Indonesia | 0.571527163 | 0.838900871 | 1.403124048 | 3.501697458 | 7.460570993 | 17.41922069 | 33.78203175 | 62.96879696 | 118.6719195 | 211.3622258 | 297.2592966 | 446.8252194 | 691.859408 | 1068.729282 | 913.7647225 | 1136.467348 | 1616.896214 | 63.85255532 |
| Iran (Islamic Republic of) | 0.12856776 | 0.159601337 | 0.242186306 | 0.456470857 | 0.860058102 | 0.528487734 | 1.797627423 | 2.485011783 | 3.895393858 | 6.354527118 | 10.03961947 | 18.43517901 | 33.84486323 | 114.6237119 | 65.92512651 | 115.8799511 | 219.8993871 | 9.029243644 |
| Iraq | 0.481826616 | 0.836583684 | 1.12619237 | 2.053167425 | 3.885649074 | 4.222878386 | 9.865412608 | 18.62326972 | 31.32127803 | 52.72936567 | 74.95400169 | 108.6768933 | 161.8738797 | 278.7589961 | 229.2466715 | 303.3175943 | 393.6249643 | 19.03639043 |
| Ireland | 0.01041506 | 0.020721364 | 0.044489364 | 0.127472068 | 0.172626521 | 4.712245655 | 9.124823502 | 15.75947896 | 27.91283316 | 44.39091798 | 62.61795101 | 220.8354656 | 303.7208371 | 498.3557974 | 1097.149137 | 1392.227023 | 1919.385689 | 39.01835831 |
| Israel | 0.01354671 | 0.030309497 | 0.072112444 | 0.219336052 | 0.47138096 | 15.58448859 | 38.20440726 | 74.54340544 | 135.4128244 | 223.6902213 | 335.846628 | 444.1921319 | 647.4662623 | 895.4056366 | 799.175776 | 968.5557732 | 1093.806756 | 60.99854348 |
| Italy | 0.01122875 | 0.02038028 | 0.035713808 | 0.135129601 | 24.21942611 | 4.949223176 | 11.66101809 | 22.56933957 | 40.39691912 | 456.4967966 | 654.2318424 | 910.6863822 | 1248.844835 | 1623.608512 | 1466.186052 | 1881.27111 | 2303.253162 | 58.54733145 |
| Jamaica | 0.360907469 | 0.707907021 | 1.105570643 | 3.066895478 | 4.561785575 | 5.483802998 | 11.81422575 | 21.47850938 | 37.86767736 | 83.02099123 | 145.219099 | 186.3188383 | 279.6912685 | 487.5616924 | 195.5733816 | 278.8781836 | 370.5831904 | 17.09156384 |
| Japan | 0.010981926 | 0.021157347 | 1.320301136 | 3.374700326 | 7.620902082 | 17.65203508 | 41.88185342 | 73.75052885 | 125.7763141 | 172.8800079 | 218.7356545 | 286.854519 | 384.8892358 | 513.3132136 | 458.0786216 | 591.4580652 | 771.2085648 | 24.8482465 |
| Jordan | 0.130164608 | 0.156549988 | 0.267737029 | 0.605018592 | 1.073877355 | 16.47624075 | 36.22728958 | 69.29801461 | 118.6401551 | 186.5323144 | 279.03418 | 388.9682841 | 557.4526117 | 884.2378947 | 1119.665066 | 1439.033465 | 1726.145975 | 57.65642788 |
| Kazakhstan | 0.089248473 | 0.169920853 | 0.262294769 | 0.503441727 | 0.747617275 | 2.731071632 | 8.78204709 | 15.93246019 | 25.88434511 | 40.4185336 | 54.7020771 | 78.9707674 | 112.0621049 | 227.4522418 | 164.2128641 | 242.5048437 | 341.5034122 | 23.95416282 |
| Kenya | 0.181582876 | 0.347089483 | 0.37352811 | 1.125490035 | 2.457761659 | 0.529150344 | 2.023305696 | 4.637176843 | 9.472502104 | 18.55377676 | 34.10942642 | 60.69402207 | 108.3888407 | 311.9837535 | 195.8173094 | 334.8255433 | 514.3764904 | 35.01551551 |
| Kiribati | 1.311129152 | 3.929673205 | 7.405002077 | 16.780455 | 2.626440321 | 105.2635515 | 219.43893 | 380.9609839 | 542.3555381 | 695.5856096 | 964.4195178 | 1208.910583 | 1119.769881 | 1633.402673 | 1294.524378 | 2337.435154 | 2703.992131 | 112.7827363 |
| Kuwait | 0.109745216 | 0.194031841 | 0.187500575 | 0.407781718 | 0.600520933 | 42.83850349 | 84.80145248 | 159.7193928 | 244.628489 | 347.3154763 | 503.1345857 | 690.753913 | 871.7698755 | 1406.094762 | 195.6477663 | 193.829167 | 320.092498 | 93.19872354 |
| Kyrgyzstan | 0.089687377 | 0.12751295 | 0.228720152 | 0.406746757 | 0.685674952 | 6.803557333 | 15.49776052 | 32.92702468 | 57.9357137 | 46.27561915 | 60.54077779 | 86.20825622 | 116.5541905 | 226.0467887 | 536.1274668 | 753.9555258 | 810.6092495 | 27.15905984 |
| Lao People's Democratic Republic | 0.606731623 | 0.881604003 | 1.417999555 | 2.91700697 | 1.908814042 | 9.459578518 | 20.48526512 | 39.49378507 | 67.98631209 | 112.4127699 | 160.0450963 | 225.3599764 | 320.4896931 | 506.0396618 | 436.1774572 | 565.5621346 | 719.4615765 | 20.10625555 |
| Latvia | 0.098746663 | 0.189947093 | 0.650070052 | 1.576816511 | 2.596472659 | 4.59977807 | 11.26693977 | 22.26399486 | 43.4822772 | 67.72362511 | 114.9964497 | 170.0820928 | 241.3137205 | 403.0442286 | 332.6749333 | 444.8797296 | 510.2238666 | 14.4125261 |
| Lebanon | 0.080800283 | 0.121271514 | 0.182536647 | 0.388516965 | 0.699554563 | 15.53996023 | 39.20439227 | 71.86220618 | 114.1919997 | 171.0121266 | 213.5256936 | 286.4395711 | 383.3470886 | 528.9206229 | 196.0285219 | 218.7618012 | 292.1384954 | 40.09043603 |
| Lesotho | 1.101153678 | 1.200632788 | 1.283471268 | 2.186044781 | 5.586679111 | 2.819127802 | 8.909618816 | 22.43644306 | 44.9455489 | 89.8469481 | 167.0220694 | 259.6251724 | 403.3348941 | 697.066262 | 1186.453517 | 1660.648214 | 1897.073495 | 17.19111577 |
| Liberia | 0.752916786 | 0.561702307 | 0.937387361 | 1.405844035 | 1.974405713 | 1.944438456 | 5.861397652 | 10.40653662 | 16.34725772 | 27.04491653 | 38.3680524 | 59.02685703 | 85.06852789 | 173.2014687 | 125.1996117 | 170.0758774 | 231.6015971 | 18.76683798 |
| Libya | 0.07024363 | 0.128093059 | 0.576069111 | 0.531068224 | 1.069086082 | 4.456670133 | 8.228407852 | 16.63730327 | 30.16196278 | 59.56090155 | 92.26919611 | 121.3498481 | 173.3058484 | 238.5054062 | 386.2291149 | 489.6747788 | 473.3214982 | 12.38509437 |
| Lithuania | 0.025586084 | 0.041478878 | 0.122917888 | 0.313166425 | 0.509836108 | 0.585105527 | 3.336404587 | 4.219339174 | 6.283795986 | 11.84040279 | 17.91239791 | 24.40913017 | 30.93466024 | 43.17023002 | 38.73488095 | 41.99747468 | 60.25082896 | 7.785647117 |
| Luxembourg | 0.010127744 | 0.018672786 | 1.082823381 | 3.40001313 | 7.053056128 | 15.35952998 | 33.28551125 | 63.51902064 | 97.54525703 | 138.7097591 | 198.5948042 | 263.2816611 | 376.834644 | 594.9355259 | 525.4648991 | 621.7943871 | 747.0953195 | 48.31492744 |
| North Macedonia | 0.030966418 | 0.071770947 | 0.111771375 | 0.293561572 | 0.636158311 | 1.998020187 | 6.004787426 | 10.60059899 | 19.51517758 | 29.37692101 | 42.87653064 | 71.57500945 | 99.82301834 | 223.0897105 | 158.1459321 | 230.3056215 | 337.4669528 | 23.63266322 |
| Madagascar | 0.240862518 | 0.435800291 | 0.487794335 | 1.250660994 | 2.740478989 | 0.265335175 | 0.984639612 | 1.729571683 | 3.461108872 | 6.339071764 | 12.52351088 | 97.12834257 | 141.5802816 | 233.1497276 | 408.3608056 | 509.6449693 | 653.6585214 | 18.77509943 |
| Malawi | 0.263230451 | 0.485296171 | 4.840360326 | 1.378221012 | 2.758811477 | 69.3512101 | 16.69181703 | 37.29702505 | 59.67706131 | 107.1295473 | 142.590799 | 242.3018552 | 347.3760747 | 556.2855947 | 493.6133548 | 566.5216071 | 831.7182287 | 12.3914722 |
| Malaysia | 0.145749191 | 0.203120714 | 0.331714953 | 0.866890916 | 1.410150231 | 9.916148571 | 23.98487309 | 47.90450556 | 71.22142179 | 126.9721273 | 222.9288246 | 278.3823068 | 458.1214757 | 740.2854917 | 671.7046972 | 751.6958583 | 1105.440342 | 16.73661945 |
| Maldives | 0.105019048 | 0.148512964 | 0.295977504 | 0.766702819 | 1.291245114 | 2.415055728 | 5.910909305 | 12.05946802 | 22.59390238 | 43.59028073 | 71.53140106 | 115.149141 | 171.9306328 | 350.3685446 | 275.1899365 | 396.0549978 | 557.7902123 | 9.71344954 |
| Mali | 0.807473162 | 0.540269874 | 0.930960157 | 1.344099723 | 2.198342662 | 4.9611682 | 13.13729418 | 28.32724319 | 41.84551071 | 78.62645551 | 140.093278 | 187.4923332 | 257.5128131 | 444.9165425 | 112.2298245 | 201.746303 | 290.2874649 | 9.743886572 |
| Malta | 0.036811804 | 0.07183931 | 0.13071082 | 0.387983383 | 13.71106623 | 0.972173843 | 3.093098043 | 5.378787781 | 10.33548509 | 19.72665616 | 35.38097368 | 62.06521046 | 113.5154091 | 281.5273399 | 200.357494 | 280.2183964 | 454.7129928 | 27.13998795 |
| Marshall Islands | 0.538332669 | 1.282931634 | 2.920339662 | 7.809580843 | 1.714777882 | 45.11926308 | 89.92147959 | 171.3808773 | 257.2540789 | 359.2362433 | 500.9772181 | 662.2405269 | 813.6345618 | 1228.886784 | 1084.074409 | 1460.998435 | 1643.833948 | 60.86805145 |
| Mauritania | 0.53570791 | 0.408979584 | 0.650658852 | 0.987918895 | 1.479147765 | 6.671038008 | 16.18618142 | 32.52738617 | 56.91381652 | 94.56817302 | 137.8382497 | 197.2326098 | 280.4592867 | 427.544163 | 370.1229706 | 480.5807211 | 610.4334207 | 20.18697267 |
| Mauritius | 0.445472163 | 0.702255485 | 1.619241888 | 4.870140137 | 10.70428461 | 20.14702649 | 59.10807302 | 106.6265372 | 189.2245401 | 336.003322 | 507.9391216 | 730.9124392 | 984.4398227 | 1359.897475 | 1157.291144 | 1531.232976 | 1637.130181 | 142.4928279 |
| Mexico | 0.275604691 | 0.608084061 | 1.313212472 | 3.543798605 | 7.557603274 | 6.170998626 | 17.29781315 | 38.34581271 | 62.66609327 | 117.8066135 | 155.659171 | 264.4691192 | 384.9203211 | 656.8159262 | 582.3839651 | 782.3632292 | 1151.836431 | 15.9620189 |
| Republic of Moldova | 0.074119974 | 0.110590462 | 0.191669853 | 0.351528541 | 0.554624644 | 0.735228981 | 2.685677104 | 4.75682381 | 9.51947009 | 17.7802801 | 30.8852017 | 33.89509933 | 34.28309509 | 37.23715939 | 29.06406692 | 37.9948378 | 63.77229334 | 7.314043008 |
| Mongolia | 0.195993093 | 0.222338212 | 0.214457333 | 0.764201095 | 1.069912144 | 3.038767661 | 9.753792333 | 16.9140303 | 27.27719625 | 41.69192456 | 52.10471334 | 79.56996841 | 113.2257281 | 239.5915983 | 36.1900759 | 47.15878905 | 80.75996234 | 25.88943851 |
| Montenegro | 0.02982094 | 0.097198675 | 0.14683675 | 0.331903336 | 0.452193318 | 3.706412144 | 11.33523973 | 18.83815204 | 29.79995113 | 46.82291926 | 66.8936899 | 94.29888946 | 136.7031695 | 273.5735595 | 199.8071006 | 286.9031242 | 379.8584474 | 27.89909661 |
| Morocco | 0.088269446 | 0.125421378 | 0.033564432 | 0.450742689 | 0.923434141 | 0.326035617 | 1.25983831 | 2.791439162 | 5.585792563 | 12.9549075 | 23.62440205 | 42.2181699 | 83.73005574 | 150.3124996 | 267.7062575 | 334.2612134 | 437.353635 | 16.66181143 |
| Mozambique | 0.377818598 | 0.703943735 | 2.360334537 | 2.053118004 | 4.646884773 | 21.37558082 | 44.56105145 | 80.43799002 | 122.7296615 | 176.2284524 | 240.6052439 | 302.273163 | 405.846346 | 583.1241045 | 496.325848 | 638.5456553 | 790.2103742 | 29.63497177 |
| Myanmar | 0.625335981 | 1.038987942 | 1.7170289 | 5.027000599 | 7.787674748 | 11.69347603 | 31.54942115 | 56.28771439 | 100.860105 | 129.82961 | 171.9385433 | 207.0179819 | 300.9723438 | 440.3696057 | 356.5042429 | 535.2072275 | 725.8426609 | 40.37885797 |
| Namibia | 0.443733007 | 0.43568247 | 0.380919983 | 0.669184415 | 2.068737817 | 5.713856862 | 17.2955773 | 41.59485713 | 84.43813996 | 74.72324985 | 104.3787576 | 122.0605494 | 178.8752457 | 305.8565203 | 224.6824773 | 349.1521843 | 403.2639824 | 26.20683255 |
| Nepal | 0.368221236 | 0.094255671 | 0.767388571 | 1.694029857 | 3.075164856 | 0.214141336 | 0.992567084 | 1.83521121 | 3.400595283 | 6.674166781 | 11.35223417 | 20.86442989 | 38.94906445 | 99.77888982 | 369.8448444 | 495.7594842 | 655.6632878 | 7.086472719 |
| Netherlands | 0.011377048 | 0.014595301 | 1.429562552 | 3.621350312 | 7.51574242 | 8.895924112 | 22.0992296 | 41.20567735 | 72.55956135 | 125.9805279 | 206.7972987 | 351.3763702 | 601.0156023 | 1172.333927 | 372.2968147 | 446.2862925 | 553.6457341 | 100.7170749 |
| New Zealand | 0.035453117 | 0.048205522 | 0.110021326 | 0.430848834 | 0.595802423 | 20.83827563 | 43.03340326 | 76.56382839 | 124.2886028 | 195.3804464 | 289.2495094 | 412.6551115 | 608.9808472 | 1059.974558 | 880.1654747 | 1165.158168 | 1494.758449 | 84.25549702 |
| Nicaragua | 0.302215976 | 0.40990715 | 0.619344954 | 1.270939089 | 2.715268974 | 4.11624442 | 12.13520384 | 27.69147382 | 47.72985927 | 89.90473325 | 123.3077338 | 221.8399758 | 335.0442739 | 572.1706887 | 578.7752323 | 737.205844 | 811.5612978 | 11.0166337 |
| Niger | 0.687387914 | 0.421109767 | 0.744359871 | 1.161758033 | 1.598002943 | 5.40655706 | 14.09647468 | 29.45646143 | 50.02098388 | 90.79138647 | 115.5468806 | 27.80297395 | 60.69393742 | 172.0500966 | 192.026298 | 274.6826251 | 351.5644607 | 9.767941053 |
| Nigeria | 0.441510764 | 0.205246213 | 0.528256545 | 1.183275468 | 1.266378547 | 5.882378332 | 15.68944508 | 32.3184571 | 47.93403687 | 86.66382693 | 151.4863257 | 192.5897578 | 324.6625488 | 543.6016473 | 482.3045706 | 547.0055553 | 828.3591375 | 11.18069419 |
| Democratic People's Republic of Korea | 0.104271282 | 0.256117997 | 0.356669782 | 0.751254372 | 1.236423199 | 3.761841079 | 10.05446269 | 22.47569409 | 38.592042 | 70.22507981 | 101.7501977 | 147.9354876 | 242.3093282 | 436.592584 | 388.4505142 | 474.1355429 | 633.2866527 | 18.61838337 |
| Northern Mariana Islands | 0.147339096 | 0.349145297 | 0.788467067 | 2.526877747 | 5.975147179 | 1.849119659 | 4.830194791 | 8.932621616 | 15.13132678 | 25.03083324 | 37.73278813 | 58.45162214 | 82.20225562 | 128.6292016 | 112.2755803 | 146.8880522 | 162.6575382 | 10.06316255 |
| Norway | 0.027395059 | 0.039345214 | 0.042628357 | 0.134539859 | 0.19299635 | 2.703121432 | 8.654064998 | 15.16349036 | 25.05146753 | 37.65862101 | 52.69296239 | 77.63670909 | 109.2025669 | 230.5498806 | 170.6104883 | 246.0223761 | 363.9865799 | 20.58437122 |
| Oman | 0.135834283 | 0.133832945 | 1.900417835 | 4.667106305 | 10.26578404 | 21.60315916 | 50.70642482 | 97.10280025 | 159.3974669 | 244.5142626 | 303.7982144 | 392.0109862 | 515.6996354 | 659.1858037 | 577.9473036 | 756.9092748 | 959.7717463 | 63.01416144 |
| Pakistan | 0.689561113 | 1.003249651 | 0.67907192 | 3.033796232 | 4.450160685 | 9.156724288 | 25.06622344 | 55.44789901 | 91.57414743 | 151.2295176 | 195.6553832 | 306.6334666 | 437.8203358 | 744.6425502 | 632.9332134 | 851.2864893 | 1322.384791 | 20.77449513 |
| Palestine | 0.199035524 | 0.309008661 | 0.582191424 | 0.850524967 | 1.816711121 | 4.168592568 | 13.67073111 | 32.8489774 | 84.3987047 | 147.86403 | 277.0771229 | 481.1217339 | 755.6396855 | 1192.331116 | 1014.522656 | 1330.582415 | 1687.421538 | 25.36657402 |
| Panama | 0.217442195 | 0.299982753 | 0.505638561 | 1.073210869 | 1.908194666 | 5.974766218 | 15.64865372 | 32.88366399 | 48.11432883 | 89.44969696 | 157.2923237 | 178.4536041 | 295.5950001 | 470.8772916 | 383.8832976 | 483.8728095 | 715.627924 | 8.268091622 |
| Papua New Guinea | 0.873723155 | 1.864692446 | 3.566120656 | 9.829750081 | 19.96910298 | 1.715075051 | 5.102634482 | 11.41418492 | 20.13703928 | 41.01297991 | 62.93252211 | 94.1858212 | 139.9872566 | 269.9513048 | 220.2606853 | 355.0729684 | 420.7923035 | 11.03160489 |
| Paraguay | 0.253217662 | 0.430802873 | 0.769599713 | 1.724518525 | 18.88807249 | 7.210233803 | 18.81333589 | 39.8295697 | 70.76017757 | 126.555895 | 193.4857545 | 298.1767534 | 444.6111963 | 688.6036393 | 593.1267634 | 741.9173687 | 799.4115048 | 34.48808384 |
| Peru | 0.110186542 | 0.198664791 | 0.365171524 | 0.786684381 | 1.337314928 | 0.404441989 | 1.891012755 | 3.112803742 | 4.728845049 | 8.527909976 | 14.07723434 | 24.21229725 | 48.86786797 | 163.0285021 | 86.79595882 | 164.9804136 | 265.0410695 | 11.63680306 |
| Philippines | 0.55588185 | 0.919077168 | 1.504335786 | 3.16663813 | 5.369878992 | 0.495274564 | 1.672086899 | 2.42483023 | 3.548213635 | 6.54858082 | 10.07449757 | 17.74618634 | 32.54117338 | 109.9305924 | 65.10168809 | 108.3620882 | 211.0150824 | 9.742506316 |
| Poland | 0.023942042 | 0.048487531 | 0.095456546 | 0.267513103 | 0.533130282 | 0.57589952 | 2.319373076 | 4.322106468 | 8.025922432 | 15.12620504 | 25.83617442 | 43.89881879 | 77.83146814 | 219.3108585 | 150.3373573 | 206.6372007 | 277.0729728 | 21.22081197 |
| Portugal | 0.03671452 | 0.070788569 | 1.166701005 | 3.083110175 | 6.185456992 | 15.80610575 | 33.99789219 | 66.71265713 | 108.9776497 | 161.7618351 | 253.6432625 | 324.0450437 | 475.0109454 | 707.5410303 | 570.1500842 | 813.4623862 | 939.6553204 | 54.20418058 |
| Puerto Rico | 0.069762723 | 0.214274789 | 0.479785174 | 1.506426391 | 3.126696522 | 2.891291888 | 6.910321669 | 13.46371927 | 25.20673328 | 44.05629803 | 70.63226886 | 110.455978 | 170.9074266 | 344.9927279 | 255.8092798 | 389.4564171 | 544.2515052 | 21.3693747 |
| Qatar | 0.129516713 | 0.096993629 | 0.066717337 | 0.370013544 | 1.13130123 | 0.489077501 | 1.611350493 | 2.519190845 | 4.268306583 | 7.747026825 | 13.26256451 | 23.79594259 | 40.86387318 | 120.0775477 | 203.6226621 | 279.1037279 | 366.9365131 | 8.923169614 |
| Romania | 0.050352885 | 0.08999068 | 0.693749905 | 0.291773917 | 0.373055741 | 9.453970002 | 25.93923508 | 54.97554699 | 87.54332887 | 148.7335376 | 180.3196541 | 260.137234 | 341.543997 | 643.5001673 | 512.6922276 | 677.3145089 | 849.1201461 | 14.376568 |
| Russian Federation | 0.026611092 | 0.046213669 | 0.079520642 | 0.186954461 | 0.332575725 | 0.551472186 | 2.415958737 | 4.553429998 | 9.387676488 | 20.56435704 | 38.48136397 | 57.11212536 | 84.16980648 | 98.26732585 | 98.68548516 | 84.55027285 | 118.0938526 | 12.15830625 |
| Rwanda | 0.217583515 | 0.374592926 | 0.428080457 | 1.247572097 | 2.575032107 | 11.19313605 | 31.97409179 | 66.97697585 | 113.8076303 | 167.9420164 | 250.0641964 | 364.8602765 | 485.5737659 | 718.2808621 | 624.6049002 | 723.2057017 | 892.2160328 | 35.531592 |
| Saint Lucia | 0.610202828 | 1.136449851 | 1.84045815 | 4.163904928 | 0.323897669 | 12.89404367 | 29.5406988 | 49.58260478 | 88.31067612 | 146.6756983 | 227.6444853 | 347.9963811 | 503.0929815 | 1078.032655 | 778.4866235 | 1182.338804 | 1924.844523 | 71.970901 |
| Saint Vincent and the Grenadines | 0.913659285 | 1.5893867 | 2.499678695 | 5.178479598 | 8.725680568 | 3.29642321 | 8.751857674 | 15.30701061 | 19.76880743 | 37.06075026 | 50.94769667 | 35.69314525 | 59.01025365 | 132.1050367 | 745.5938491 | 826.0857037 | 747.6582221 | 13.09540136 |
| Samoa | 0.349098884 | 0.869960953 | 0.139041652 | 4.962287226 | 11.92290256 | 1.536926003 | 59.59782898 | 116.2612011 | 181.7461734 | 266.1977815 | 394.9804097 | 537.5134708 | 696.0851766 | 1130.603688 | 936.4218668 | 1326.772927 | 1504.380197 | 57.3864312 |
| Sao Tome and Principe | 0.269596411 | 0.068422004 | 1.277551517 | 0.289378823 | 0.280708633 | 13.26942584 | 6.50168147 | 13.73790271 | 22.10072807 | 36.64336206 | 69.87451293 | 87.33793574 | 137.0694995 | 227.4294391 | 190.4698244 | 243.8029573 | 369.4591536 | 6.367737822 |
| Saudi Arabia | 0.138546899 | 0.280561501 | 0.455179695 | 0.971055449 | 1.791485702 | 16.654131 | 45.93130865 | 98.09355844 | 175.3507599 | 312.1055919 | 453.4244881 | 649.5322359 | 904.2554007 | 1297.708452 | 1133.895018 | 1362.469703 | 1566.330144 | 129.0031073 |
| Senegal | 0.78352306 | 0.446953441 | 0.817252036 | 1.327771832 | 37.97799427 | 5.843953918 | 15.39733468 | 32.67213138 | 49.81194657 | 93.24669177 | 166.8676775 | 215.4047755 | 359.4396664 | 622.2728865 | 545.7472049 | 638.4250524 | 923.2909381 | 16.05667353 |
| Serbia | 0.025008332 | 0.036365333 | 0.191014546 | 0.271961719 | 0.489547858 | 2.545535683 | 4.978466772 | 11.51612255 | 22.00677925 | 43.52900682 | 86.43363949 | 127.7230316 | 222.4826792 | 416.0245463 | 169.146931 | 259.9589155 | 362.2284771 | 40.17754051 |
| Seychelles | 0.141548577 | 0.260853345 | 0.500704568 | 1.291302395 | 2.198187051 | 0.48004956 | 1.595474631 | 2.1762534 | 3.425299385 | 5.359856955 | 8.129424174 | 15.22166666 | 29.83339207 | 122.1895771 | 62.50776155 | 121.4321143 | 254.0826991 | 9.660559875 |
| Sierra Leone | 0.7781966 | 0.558907631 | 0.949727395 | 1.358863601 | 2.012712458 | 5.884676643 | 14.85662816 | 31.18791652 | 44.98021501 | 84.15092244 | 145.7051366 | 182.675555 | 305.5181165 | 511.2840545 | 466.6498857 | 502.1173296 | 755.4881167 | 12.13778725 |
| Singapore | 0.011147489 | 0.019449232 | 0.044600349 | 0.101217717 | 8.219205976 | 0.302184458 | 0.847636458 | 1.482987252 | 2.571261173 | 4.503101218 | 7.687429886 | 13.60412692 | 22.68971143 | 51.47319973 | 36.4981806 | 46.4922025 | 84.41545277 | 3.087880235 |
| Slovakia | 0.011293036 | 0.016947938 | 0.028647351 | 0.09809369 | 0.190887617 | 0.635388603 | 2.626987538 | 4.509548217 | 8.65117313 | 17.82226675 | 31.96604317 | 1639.096178 | 1954.329848 | 3169.145672 | 2727.857019 | 3985.585585 | 4273.131581 | 186.3841028 |
| Slovenia | 0.017372302 | 0.019109533 | 0.197724104 | 0.100946982 | 0.199444867 | 2.694295329 | 28.4070378 | 55.65785963 | 98.52464356 | 161.136283 | 246.9118878 | 329.9103724 | 457.8668691 | 597.3096193 | 125.1136962 | 134.2842316 | 216.1085276 | 47.05420822 |
| Solomon Islands | 1.417068996 | 3.75812078 | 8.35479169 | 21.0373293 | 44.52650691 | 2.514174822 | 7.844617951 | 13.43218553 | 21.99367804 | 36.49622652 | 53.21421291 | 82.77885978 | 119.3652396 | 250.4804981 | 177.9630762 | 260.0995561 | 358.9554332 | 27.28451596 |
| Somalia | 0.333596938 | 0.594427425 | 0.610566355 | 1.666042709 | 3.913913162 | 2.72787637 | 7.2111595 | 15.16619438 | 27.84715532 | 52.83560068 | 86.87211596 | 149.8342774 | 237.9301019 | 464.3331846 | 363.6563294 | 530.2718751 | 677.9876241 | 25.29728607 |
| South Africa | 0.324735013 | 0.745760621 | 1.220480045 | 2.064917639 | 3.603893434 | 7.79399599 | 18.27994094 | 44.29310867 | 90.84217981 | 213.0783333 | 326.97643 | 429.5635411 | 577.4180932 | 1057.907944 | 866.4594171 | 1212.370854 | 1585.703824 | 48.34177619 |
| Republic of Korea | 0.05112163 | 0.093808732 | 0.158198277 | 0.447902452 | 0.785338145 | 14.0718182 | 32.66033021 | 62.19815482 | 109.7767991 | 171.3515725 | 267.4960985 | 373.0589822 | 528.5115422 | 829.2038966 | 713.6336392 | 923.4423111 | 1060.604851 | 50.76417796 |
| South Sudan | 0.202943308 | 0.330352236 | 0.384076684 | 1.024248529 | 2.293670536 | 2.246483821 | 7.272086934 | 12.71952458 | 20.48340011 | 33.09959801 | 46.7508121 | 69.66641314 | 101.3220341 | 216.679583 | 154.6281011 | 221.5526208 | 309.9201056 | 22.01916407 |
| Spain | 0.011701185 | 0.018918121 | 0.042326231 | 0.116606013 | 0.172743677 | 0.384412795 | 1.282708383 | 2.179538525 | 3.601857808 | 5.443073043 | 7.778228188 | 11.17532628 | 16.30350166 | 42.67783795 | 25.54144182 | 38.89171845 | 66.63604177 | 6.659339395 |
| Sri Lanka | 0.274647889 | 0.549718958 | 0.953498602 | 2.064067649 | 3.568191894 | 12.81201217 | 29.77365718 | 54.05524843 | 96.57842847 | 159.5161628 | 227.5934368 | 320.7156834 | 495.7217401 | 727.8695375 | 646.6513494 | 802.2516267 | 818.4432652 | 52.26442984 |
| Sudan | 0.075056825 | 0.128543116 | 0.195637039 | 0.470961787 | 0.904043398 | 11.82447809 | 34.0669414 | 68.57926778 | 118.2697088 | 195.0917945 | 286.6280954 | 409.525167 | 552.1970845 | 742.6000622 | 187.5134831 | 220.7758865 | 265.6921805 | 30.85396231 |
| Suriname | 0.678440713 | 0.896321371 | 0.475446609 | 1.080983244 | 1.936565998 | 11.72076721 | 35.18070531 | 76.70461224 | 116.4538389 | 176.3798515 | 204.5075953 | 292.3967306 | 374.3426324 | 524.3907454 | 229.0467433 | 303.1526931 | 393.3510323 | 17.45293414 |
| Eswatini | 1.601475892 | 1.80258536 | 1.827444818 | 3.09203072 | 7.62517027 | 13.31736133 | 31.12494382 | 58.30672241 | 102.9228004 | 25.70379863 | 43.212366 | 66.18615332 | 99.71180303 | 209.4865234 | 150.9740245 | 216.1375587 | 305.8692152 | 19.75720832 |
| Sweden | 0.0163721 | 0.043146272 | 0.077492429 | 0.275672144 | 0.364496405 | 0.570604923 | 2.332243585 | 3.908323323 | 6.381451606 | 12.08152357 | 20.67690814 | 34.09690746 | 66.86974315 | 224.6157402 | 127.0022571 | 222.4619485 | 386.901557 | 19.39100656 |
| Switzerland | 0.014432816 | 0.019256959 | 0.034506193 | 0.112681951 | 0.174979881 | 0.448288417 | 2.609772466 | 5.402234843 | 11.39964105 | 24.26796236 | 44.98431454 | 82.43971764 | 138.6503506 | 344.3673483 | 254.1545158 | 388.5323861 | 527.5872039 | 30.34114344 |
| Syrian Arab Republic | 0.271781199 | 0.356043922 | 0.419630944 | 0.694245645 | 0.92256719 | 14.01784873 | 33.10400974 | 63.82341376 | 99.69266791 | 143.4906904 | 188.5241246 | 239.1294251 | 326.5534336 | 486.9457917 | 415.6173686 | 568.5270705 | 740.090989 | 31.63612625 |
| Taiwan (Province of China) | 0.038966148 | 0.068974635 | 0.112332982 | 0.56976712 | 1.177966098 | 26.11135976 | 55.46011908 | 97.26036752 | 157.9686407 | 245.9368643 | 350.0040388 | 481.8326021 | 697.706074 | 1075.416971 | 921.4477852 | 1263.795038 | 1698.004803 | 43.55244064 |
| Tajikistan | 0.498236532 | 0.564176667 | 0.480272624 | 1.220541579 | 0.369627285 | 3.589888209 | 15.61482658 | 32.73125228 | 73.91941678 | 137.0064541 | 219.3493883 | 245.151084 | 252.0031624 | 189.8232882 | 210.6704396 | 129.2670019 | 177.5992917 | 15.57518477 |
| United Republic of Tanzania | 0.261838024 | 0.445031951 | 0.499296101 | 1.392436024 | 2.867437514 | 6.48763131 | 15.70352312 | 30.05203359 | 55.25235456 | 87.22760171 | 127.4145276 | 186.3089686 | 286.3895245 | 584.380556 | 416.2598239 | 663.4424569 | 1158.12541 | 27.46298692 |
| Thailand | 0.181398401 | 0.332344871 | 0.750619812 | 1.779616477 | 3.114880252 | 15.15660075 | 33.77069469 | 59.85966436 | 92.96549728 | 135.4807579 | 180.4194141 | 227.0245993 | 302.8980722 | 456.5378031 | 377.5362638 | 528.4412737 | 726.4889744 | 40.87054543 |
| Bahamas | 0.4549078 | 0.847596677 | 1.336148287 | 3.331644356 | 2.992815542 | 8.978620005 | 21.18782263 | 40.2114735 | 56.05883526 | 100.4754638 | 165.1760452 | 224.0657403 | 347.2132576 | 566.3247593 | 484.4136074 | 568.8124529 | 794.3404889 | 36.31789244 |
| Gambia | 0.696055337 | 0.338330205 | 0.6500556 | 1.142811701 | 1.588738735 | 19.8070702 | 42.46138758 | 80.15011206 | 135.4765492 | 212.4138415 | 311.4137751 | 426.1088458 | 625.754802 | 1027.698385 | 851.6206367 | 1135.466713 | 1518.877767 | 58.00269874 |
| Timor-Leste | 0.319223914 | 0.446160205 | 0.588917142 | 1.270926362 | 2.165916131 | 38.95612469 | 84.8951554 | 144.8520289 | 223.7756189 | 300.8752869 | 351.7787602 | 419.4534936 | 527.382488 | 660.6078707 | 598.0786634 | 764.7487215 | 950.127178 | 39.97872783 |
| Togo | 0.724969665 | 0.406724805 | 0.738518374 | 1.249458052 | 1.643429518 | 2.881702629 | 14.34424198 | 38.40062834 | 81.14407892 | 150.2925695 | 241.6920617 | 314.5688481 | 511.5300786 | 910.205739 | 815.6888181 | 1066.845429 | 1351.755715 | 13.33077713 |
| Tonga | 0.313234313 | 0.76769474 | 1.443162054 | 4.210858733 | 9.729027916 | 23.91578207 | 58.0755066 | 124.1048069 | 214.6207446 | 325.192125 | 489.8941461 | 661.9982135 | 829.5816908 | 1361.943749 | 1096.662775 | 1525.983937 | 1877.695367 | 78.90042003 |
| Trinidad and Tobago | 0.319599216 | 0.580942811 | 1.113119927 | 3.193540873 | 6.209367145 | 26.76413056 | 60.02269745 | 106.2795022 | 168.7697359 | 234.4680891 | 287.9705651 | 344.089528 | 449.8774124 | 553.7448321 | 503.8208392 | 627.1342435 | 777.1769105 | 42.66766388 |
| Tunisia | 0.040269349 | 0.077352437 | 0.131349969 | 0.35897208 | 0.644662203 | 4.926912315 | 11.6185361 | 21.39481321 | 35.61199848 | 62.09320634 | 98.46722451 | 135.9365236 | 234.6255702 | 398.0167916 | 319.7549264 | 411.2251084 | 608.3630936 | 23.71455915 |
| Turkey | 0.07607293 | 0.113588516 | 0.165890571 | 0.358637955 | 0.780100918 | 14.09656229 | 29.44390212 | 56.07019182 | 97.16045151 | 144.5497331 | 201.4768579 | 272.5014395 | 402.5996638 | 633.5911224 | 526.2279376 | 687.2876013 | 825.2936123 | 45.64229559 |
| Turkmenistan | 0.284145081 | 0.374869289 | 0.404315943 | 0.945590531 | 0.605561156 | 3.219206114 | 14.41192096 | 26.63216798 | 53.40880017 | 87.87987932 | 101.4249148 | 106.0277624 | 91.72862026 | 64.48878442 | 65.34237129 | 47.14243573 | 79.74885561 | 12.98508886 |
| Uganda | 0.263602572 | 0.483408636 | 0.523427917 | 1.479677525 | 3.052998972 | 4.675343268 | 10.88870603 | 18.77619029 | 32.89526694 | 55.65159825 | 85.4172089 | 127.0333001 | 190.4325247 | 314.9389355 | 260.353934 | 338.2799037 | 385.5754751 | 24.95607999 |
| Ukraine | 0.055335549 | 0.113211258 | 0.206162685 | 0.48838011 | 0.719581944 | 1.866814544 | 5.586764033 | 14.01259772 | 28.93991567 | 55.76124639 | 89.99576909 | 141.2989522 | 233.0316419 | 365.5814359 | 313.549505 | 383.3600048 | 503.0077454 | 16.83417506 |
| United Arab Emirates | 0.092659866 | 0.100135804 | 0.097197332 | 0.433934807 | 0.920991541 | 2.286752713 | 7.862100321 | 12.96616787 | 21.62252233 | 34.94307089 | 52.50701796 | 79.01701041 | 110.9980961 | 240.7109716 | 172.612997 | 244.5339422 | 322.806049 | 27.36684326 |
| United Kingdom | 0.02042966 | 0.036533862 | 0.061770908 | 0.224598743 | 0.334694662 | 10.79566247 | 25.86642553 | 47.30574911 | 79.6361713 | 135.2751019 | 242.7915743 | 372.8649943 | 578.6070614 | 1026.726866 | 853.59077 | 1068.418843 | 1555.44149 | 80.54446178 |
| United States of America | 0.037931732 | 0.090875234 | 0.174059761 | 0.814249146 | 1.338652518 | 8.994431981 | 22.45012914 | 45.21668168 | 86.50676517 | 141.8139244 | 228.1083857 | 325.6759281 | 472.7697566 | 749.9839638 | 627.2060371 | 836.7159107 | 1006.146036 | 44.99005793 |
| Uruguay | 0.080136506 | 0.177455036 | 0.210574855 | 0.590662563 | 0.842430166 | 0.57946815 | 3.022218837 | 5.506530723 | 9.482808186 | 18.15165575 | 30.57127716 | 48.07297783 | 78.81139298 | 217.945002 | 136.6677713 | 207.4962956 | 356.6782935 | 18.77731892 |
| Uzbekistan | 0.152897092 | 0.298279648 | 0.384117668 | 0.858191276 | 1.156750428 | 2.064029583 | 6.683516595 | 13.78381105 | 23.22494466 | 38.06907666 | 60.95660305 | 98.01383874 | 155.7436158 | 327.9942041 | 248.144958 | 335.6111927 | 494.1737529 | 36.38237188 |
| Vanuatu | 0.345978972 | 0.760118199 | 1.678263702 | 4.824012037 | 11.89708221 | 29.6099502 | 60.55106658 | 116.6006048 | 174.1549786 | 245.9350062 | 351.8867516 | 457.4961829 | 562.3593939 | 833.2952854 | 742.7064333 | 1023.8676 | 1128.645327 | 41.61751376 |
| Venezuela (Bolivarian Republic of) | 0.441419279 | 0.670628875 | 0.203576502 | 1.785915903 | 3.285405512 | 2.576831061 | 16.0112214 | 32.97787448 | 64.24512598 | 111.2596799 | 175.2965262 | 260.5312318 | 371.7784021 | 507.8608537 | 483.5142497 | 484.61495 | 546.3027999 | 37.71037522 |
| Viet nam | 0.200926572 | 0.294934161 | 0.46581429 | 1.194648322 | 2.460672532 | 4.752375758 | 12.22906591 | 21.23585083 | 41.22321753 | 66.48217043 | 111.0364666 | 92.54883667 | 126.5805753 | 260.4488443 | 125.6566046 | 133.2272938 | 149.3411243 | 27.66916827 |
| Virginia | 0.03151292 | 0.071688423 | 0.149175367 | 0.697378634 | 1.180289777 | 21.52107308 | 47.11857657 | 87.52136681 | 140.1425669 | 195.2249808 | 252.0539255 | 312.1698432 | 402.4486335 | 551.0262391 | 483.2704772 | 636.7407986 | 814.155828 | 34.62699484 |
| Yemen | 0.048120419 | 0.090205439 | 0.141653334 | 0.369241271 | 0.770158756 | 2.020724147 | 6.49222694 | 10.7670988 | 18.37040933 | 29.37708893 | 44.37752147 | 70.5673002 | 99.76945696 | 231.3617502 | 162.2443688 | 236.8435056 | 335.0967111 | 21.81322297 |
| Zambia | 0.355633202 | 0.694086587 | 0.68474953 | 1.882389463 | 4.123303755 | 18.5046936 | 43.94187451 | 74.62479177 | 118.6552659 | 163.3231016 | 172.358728 | 226.1204655 | 307.6223027 | 463.4650538 | 391.4986946 | 520.8139003 | 694.3411662 | 52.81036231 |
| Zimbabwe | 1.865406097 | 1.289389197 | 0.032122711 | 0.138076136 | 0.240713108 | 16.89526277 | 41.05733306 | 72.25277532 | 125.6605754 | 179.4961377 | 214.781805 | 251.7931866 | 321.0195982 | 404.8463612 | 134.6796728 | 258.3883553 | 455.750683 | 30.74113477 |
| Monaco | 0.008951047 | 0.025619708 | 0.05482575 | 0.140011504 | 0.25995541 | 0.21126326 | 0.850335843 | 1.464166158 | 2.643524949 | 5.005738567 | 8.804875016 | 16.39521965 | 29.66316447 | 79.04806957 | 52.85761587 | 76.64802068 | 121.1085695 | 9.34640453 |
| San Marino | 0.011403826 | 0.033973183 | 0.064791335 | 0.154180496 | 0.200158506 | 4.56886112 | 11.63092042 | 24.40932962 | 39.83174601 | 72.43692439 | 104.7246618 | 150.4110816 | 247.3317436 | 490.9211276 | 421.4265303 | 553.8335903 | 747.8631387 | 18.71924347 |
| Saint Kitts and Nevis | 0.395295061 | 0.543645776 | 0.59153785 | 1.347213692 | 2.535382872 | 5.577605772 | 15.65329117 | 33.49807127 | 51.70519384 | 92.74323463 | 168.1893592 | 205.3561481 | 345.4285937 | 578.5541678 | 513.5178901 | 630.498769 | 901.4737948 | 13.682911 |
| Cook Islands | 0.276367081 | 0.911974032 | 1.864074689 | 5.499860556 | 13.02122966 | 2.480354824 | 9.167860358 | 17.17735204 | 28.92693836 | 52.95159725 | 90.38527877 | 166.4660574 | 273.0380716 | 531.7633195 | 425.8157554 | 600.7947204 | 652.2441086 | 45.00670597 |
| Nauru | 0.884935699 | 2.441610827 | 1.057761245 | 12.90914646 | 29.0135775 | 6.688810274 | 132.2196425 | 243.7818167 | 362.2234653 | 509.0554814 | 717.4323995 | 979.8345708 | 1202.975163 | 1744.786389 | 1491.269582 | 2078.907731 | 2463.861748 | 55.95204912 |
| Niue | 0.634159888 | 1.464012971 | 2.450079115 | 6.101099172 | 0.164083401 | 34.40521432 | 72.02795619 | 147.701245 | 239.8540954 | 362.9724356 | 544.517356 | 761.8772268 | 989.5653047 | 1595.517331 | 1313.413652 | 1831.653983 | 2127.16908 | 156.9692804 |
| Palau | 0.486051578 | 1.523116514 | 3.055959668 | 8.809339545 | 19.05732861 | 44.48486164 | 77.03933509 | 145.9285599 | 232.2880835 | 345.828538 | 492.5184442 | 618.0385365 | 789.0424531 | 1081.197545 | 966.6752465 | 1240.808369 | 1316.163193 | 45.59579795 |
| Tokelau | 0.399071242 | 1.117723722 | 2.174334809 | 5.994930271 | 12.66948733 | 2.091366794 | 6.515231754 | 14.825219 | 26.43953417 | 48.54863639 | 72.82156811 | 107.8008217 | 146.0657729 | 209.4901683 | 999.1840235 | 1503.602448 | 1738.6095 | 6.245683071 |
| Tuvalu | 0.512696049 | 1.360974808 | 2.864746681 | 7.845798275 | 18.03223921 | 16.96102716 | 47.15707669 | 104.0800572 | 194.3566445 | 413.0436717 | 565.6016279 | 762.9083161 | 1004.099263 | 1252.769669 | 295.7512951 | 415.1377919 | 646.765709 | 59.07535117 |

**Supplementary Table 8.** Age distribution of DALYs rate for type 2 diabetes in different countries in 2019.

| 2019daly rate | 15 to 19 | 20 to 24 | 25 to 29 | 30 to 34 | 35 to 39 | 40 to 44 | 45 to 49 | 50 to 54 | 55 to 59 | 60 to 64 | 65 to 69 | 70 to 74 | 75 to 79 | 80 plus | 80-84 | 85-89 | 90-94 | all ages |
| --- | --- | --- | --- | --- | --- | --- | --- | --- | --- | --- | --- | --- | --- | --- | --- | --- | --- | --- |
| Afghanistan | 33.05786495 | 100.5031628 | 202.7229177 | 362.044011 | 575.5522892 | 926.8991602 | 1853.976511 | 2864.591573 | 4378.569035 | 14224.33881 | 16848.7193 | 6911.157285 | 7537.504083 | 7601.241964 | 8012.933839 | 7843.885476 | 7024.717169 | 1120.2578 |
| Albania | 1.231971366 | 13.63827685 | 45.81014236 | 97.89123651 | 164.6521847 | 257.8915076 | 421.7121555 | 622.0363661 | 880.4468545 | 2647.404162 | 3202.942442 | 3675.305422 | 3923.880139 | 4029.785932 | 4063.519126 | 4058.328843 | 3969.958153 | 1018.518588 |
| Algeria | 13.12051063 | 48.97662646 | 115.7994687 | 211.8669579 | 344.896237 | 569.216809 | 1032.624878 | 1717.152389 | 2541.794222 | 3597.239293 | 4437.679746 | 5053.605975 | 5658.140604 | 6615.207402 | 5971.050989 | 7979.478374 | 7866.626102 | 787.5645286 |
| American Samoa | 84.98817757 | 290.9281825 | 610.670898 | 1134.270691 | 1970.424997 | 3436.987258 | 5524.596824 | 8411.949632 | 11108.31099 | 2688.694508 | 3274.507174 | 17299.13 | 17997.04072 | 18357.38234 | 8613.227982 | 8577.043797 | 17364.47112 | 3365.394626 |
| Andorra | 2.938314036 | 29.88806002 | 65.33098963 | 108.050944 | 165.0891997 | 248.3812086 | 424.6786615 | 643.8107734 | 934.1855705 | 1319.159287 | 1729.057955 | 2178.622942 | 2526.935277 | 2902.093791 | 2793.759503 | 2895.950093 | 3064.393166 | 640.4721723 |
| Angola | 44.54873353 | 77.49631985 | 118.4022897 | 228.3539033 | 371.5151806 | 617.3930529 | 1413.75129 | 2389.96316 | 3960.384776 | 5704.761762 | 7229.449775 | 6843.173135 | 5929.809916 | 4039.357955 | 4497.861861 | 3129.734844 | 3129.120237 | 746.979895 |
| Antigua and Barbuda | 65.72373935 | 131.4024184 | 220.9882751 | 375.0400877 | 607.9563532 | 955.627586 | 1898.354716 | 3289.734891 | 4960.523441 | 7169.237161 | 8608.883745 | 10844.66383 | 12550.73572 | 14279.24817 | 14863.94929 | 13527.26318 | 13583.45187 | 2207.609029 |
| Argentina | 6.631451996 | 26.82281557 | 62.9113161 | 129.7092932 | 224.8604651 | 380.6244886 | 776.7546043 | 1280.948156 | 2022.940568 | 2941.54515 | 3827.782369 | 4408.743765 | 4827.74004 | 4667.934986 | 4780.330922 | 4688.078022 | 4322.590948 | 877.5950269 |
| Armenia | 14.66446882 | 45.81027941 | 92.37538785 | 179.6380681 | 302.5174288 | 523.6734328 | 1097.874878 | 1800.611464 | 2969.734289 | 4384.519086 | 5850.162445 | 6968.340222 | 6829.523834 | 5189.11461 | 5890.054843 | 3919.345877 | 4237.756855 | 1465.791598 |
| Australia | 1.555372472 | 8.39144798 | 29.02719555 | 76.81426793 | 144.3766779 | 257.8985052 | 463.4609515 | 700.282179 | 987.352403 | 1357.039807 | 1841.055569 | 2398.016126 | 2977.098629 | 3910.413635 | 3626.830828 | 4083.425656 | 4367.421861 | 690.9923443 |
| Austria | 1.655309394 | 17.3427822 | 44.40779459 | 84.71018287 | 140.2844873 | 228.1003688 | 431.1512647 | 719.251199 | 1137.931774 | 1734.399849 | 2344.330493 | 2878.084086 | 3390.073872 | 4370.551477 | 4151.325637 | 4512.52022 | 4750.564729 | 927.6876535 |
| Azerbaijan | 24.59533921 | 61.69164998 | 107.5481872 | 194.4007154 | 314.6734102 | 526.382606 | 1130.913386 | 1897.803663 | 2979.56861 | 4210.233012 | 5297.594794 | 5356.643186 | 5150.734248 | 4217.64842 | 4351.644677 | 3735.412403 | 4141.071228 | 965.6159091 |
| Bahrain | 45.41333124 | 106.674455 | 200.4147032 | 301.0507289 | 477.1963608 | 890.9968741 | 1918.607066 | 3694.490899 | 6828.452549 | 11054.72328 | 16097.83706 | 22726.73377 | 27840.72849 | 31001.05951 | 29879.42696 | 33300.22214 | 36555.01393 | 2175.457225 |
| Bangladesh | 53.83849646 | 76.83569331 | 122.1452709 | 203.8175508 | 320.5528464 | 580.700643 | 1088.110425 | 1584.768149 | 1743.703853 | 2474.197551 | 3319.153232 | 3517.735508 | 4551.84145 | 9266.083631 | 8467.207837 | 10392.47523 | 11184.79143 | 688.1389668 |
| Barbados | 88.4031425 | 158.0075922 | 243.76835 | 451.6570144 | 667.6092402 | 1049.671411 | 1959.147528 | 3722.304832 | 5469.036075 | 9150.593811 | 11075.82235 | 12493.35795 | 13807.13613 | 14372.6083 | 14611.25989 | 14369.29383 | 13810.09327 | 3009.130174 |
| Belarus | 1.202143488 | 10.22021839 | 45.85935875 | 104.4730975 | 173.4230701 | 256.1530978 | 415.7468571 | 587.2672754 | 786.248039 | 1003.110088 | 1192.54409 | 1289.982937 | 1356.798564 | 1427.424731 | 1408.881128 | 1437.693476 | 1489.135651 | 442.0004288 |
| Belgium | 3.055718435 | 30.99037356 | 68.12805151 | 115.4050779 | 182.3763591 | 281.8816927 | 493.6616925 | 761.2656846 | 1136.767413 | 1597.919193 | 2062.05936 | 2509.094842 | 2811.241334 | 3370.788224 | 3163.045112 | 3449.220555 | 3740.581471 | 821.5824253 |
| Belize | 85.41983168 | 154.5187618 | 263.6314857 | 493.515009 | 674.4031024 | 1080.712554 | 2269.89403 | 3914.201348 | 5570.443095 | 6965.901892 | 8479.329639 | 9855.704654 | 10353.18113 | 9700.047435 | 10273.88945 | 9278.76104 | 8662.09431 | 1327.004978 |
| Benin | 73.4448001 | 66.29988634 | 113.0737119 | 179.4313022 | 252.7401937 | 526.196108 | 1384.339739 | 2381.43975 | 3198.890167 | 4533.813681 | 5026.574837 | 6426.311078 | 6886.941757 | 7129.869435 | 7258.541964 | 6823.040627 | 7234.470919 | 427.5739639 |
| Bermuda | 32.17288286 | 68.68033559 | 117.3723879 | 197.4636921 | 312.56229 | 500.8383256 | 925.8227228 | 1479.932593 | 2061.730097 | 2735.229763 | 3473.057766 | 4255.685116 | 4969.852082 | 5671.2491 | 5626.574056 | 5658.001799 | 5815.745664 | 1510.447347 |
| Bhutan | 32.24885326 | 61.32951614 | 107.3300617 | 196.6603408 | 315.1918508 | 560.1008248 | 1018.775008 | 1613.951142 | 2310.469455 | 3452.872456 | 4281.133063 | 5263.382339 | 6750.606571 | 7905.780267 | 7808.160316 | 8275.975289 | 7743.292205 | 732.849579 |
[truncated: 48,503 more chars]
